# Metabolomics and Multi-Omics Determination of Potential Plasma Biomarkers in PRV-1-Infected Atlantic Salmon

**DOI:** 10.3390/metabo14070375

**Published:** 2024-07-02

**Authors:** Lada Ivanova, Oscar D. Rangel-Huerta, Haitham Tartor, Maria K. Dahle, Silvio Uhlig, Christiane Kruse Fæste

**Affiliations:** Norwegian Veterinary Institute, P.O. Box 64, 1431 Ås, Norway; oscar.daniel.rangel.huerta@vetinst.no (O.D.R.-H.); haitham.tartor@vetinst.no (H.T.); maria.dahle@vetinst.no (M.K.D.); silvio.uhlig@niom.no (S.U.)

**Keywords:** Atlantic salmon, biomarkers, biostatistics, lipid metabolism, metabolomics, multi-omics, *Piscine orthoreovirus*

## Abstract

Metabolomic analysis has been explored to search for disease biomarkers in humans for some time. The application to animal species, including fish, however, is still at the beginning. In the present study, we have used targeted and untargeted metabolomics to identify metabolites in the plasma of Atlantic salmon (*Salmo salar*) challenged with *Piscine orthoreovirus* (PRV-1), aiming to find metabolites associated with the progression of PRV-1 infection into heart and skeletal muscle inflammation (HSMI). The metabolomes of control and PRV-1-infected salmon were compared at three time points during disease development by employing different biostatistical approaches. Targeted metabolomics resulted in the determination of affected metabolites and metabolic pathways, revealing a substantial impact of PRV-1 infection on lipid homeostasis, especially on several (lyso)phosphatidylcholines, ceramides, and triglycerides. Untargeted metabolomics showed a clear separation of the treatment groups at later study time points, mainly due to effects on lipid metabolism pathways. In a subsequent multi-omics approach, we combined both metabolomics datasets with previously reported proteomics data generated from the same salmon plasma samples. Data processing with DIABLO software resulted in the identification of significant metabolites and proteins that were representative of the HSMI development in the salmon.

## 1. Introduction

The recent introduction of “omics” technologies in aquaculture has opened up completely new perspectives in the search for relevant biomarkers of disease development [1,2,3]. By analyzing the transcriptome, proteome, and/or metabolome in appropriate body fluids such as plasma or mucus, variables that are discriminant for the respective conditions of fish might be detected and tested for reliability as biomarkers. The different omics technologies address subsequent steps from genotype to phenotype [4]: transcriptomics provides information about gene expression under specific biological conditions, proteomics illustrates ongoing physiological changes [5], and metabolomics creates a real-time image of the current state of an organism influenced by exogenous events [6]. Since posttranslational protein modifications and metabolite flows are decoupled from the transcriptome, momentary events in biological processes can only be discovered by proteomic and metabolomic analyses [7]. Individual metabolic processes are part of extensive physiological networks and are controlled by complex regulatory mechanisms. The metabolites are the substrates and products of cellular responses that reflect the reaction of the biological system to challenges, providing a snapshot of an evanescent biological situation.

Metabolites are generally not species-specific, so that reference kits and compound libraries can be used for their identification and annotation. Metabolic profiling is performed either in a quantitative, targeted approach using standard substances for calibration or by untargeted screening at a global level as a phenotypic read-out [6]. The application of omics-based methodologies, especially metabolomics, in aquaculture is a growing field [4,8]. So far, studies have been mainly directed at understanding host-pathogen relations, identifying resistance traits, or evaluating the effects of different dietary supplements and product quality [3,9,10,11]. The elucidation of disease etiology and molecular regulation has been recently added as interesting targets of omics-driven approaches [12]. Skin and brain metabolomes of Atlantic salmon at different life stages have been investigated by untargeted analysis showing age-dependent changes in lipid and amino acid levels [13]. Metabolomics of plasma collected from salmon infected with the gill pathogen *Neoparamoeba perurans* revealed that the parasite infestation had a significant effect on ten metabolic pathways, showing that metabolites with neuroendocrine functions had been substantially affected [14]. 

Combining results from different omics analyses, such as proteomics and metabolomics, in a multi-omics approach is expected to lead to an even better understanding of molecular mechanisms and facilitate the discovery of relevant disease biomarkers [3]. Multi-omics data integration allows biological information to be connected from different hierarchical levels (gene, transcript, protein, metabolite), aspiring to put together a more holistic picture of physiological changes [5]. Integrated omics have been used rarely in the assessment of fish health and welfare until now [15]. Recently, we investigated the consequences of chloramine treatment against parasite infestation by interweaving proteomic and metabolomic data from skin mucus to find the optimal treatment dose for river rehabilitation [16]. 

In the present study, we have applied a comparable approach with the aim of identifying potential biomarkers for infections with *Piscine orthoreovirus*-1 (PRV-1), leading to the development of heart and skeletal muscle inflammation in the plasma of Atlantic salmon (*Salmo salar*). Intensive aquaculture makes farmed fish susceptible to viral and microbial infections and diseases [17]. Transmission is facilitated by high densities and decreased resilience, leading to impaired fish welfare and economic losses in aquaculture [18,19]. The early detection of health threats is thus essential for allowing the introduction of mitigation measures. This is especially relevant in cases where preventive vaccination is not possible due to the lack of effective vaccines, such as against the non-enveloped, double-stranded RNA virus PRV-1 [19]. PRV-1 infections are one of the most common viral challenges in the Norwegian salmon industry, affecting about 90% of the fish during the marine phase. Most of them will experience some degree of HSMI. The disease develops in stages, beginning with PRV-1 replication in red blood cells and subsequent infection of cardiomyocytes [20,21]. This state can then proceed into heart inflammation with cytotoxic lymphocyte activity and often also inflammation in the red muscle tissue [22,23]. Lesions in the epi-, myo- and endocardium layers of the heart usually develop between about four and eight weeks after infection with the virus, depending on the viral dose and genotype [24]. The acute phase of HSMI can last for several weeks, leading to a mortality rate of up to 20% [19]. Salmon dying from the disease often show impaired blood circulation, including internal bleeding, fluid accumulation, and liver fibrosis. 

Although PRV-1 is ubiquitously present in Norwegian coastal areas with salmon production, this does not necessarily mean that the fish will develop serious HSMI. The genotype of the virus plays a role [23,24], and fish exposed to higher levels of stress may suffer more serious consequences of the disease [25]. PRV-1 infection is persistent in infected salmon and can be detected until slaughter, often associated with macrophages located in dark spots in the muscle filets [26]. Demonstrating the presence of PRV-1-infected fish in an aquaculture facility is, therefore, not a sufficient indicator of an emerging HSMI outbreak. For this purpose, the determination of specific, characteristic changes in the fish physiology would be a more suitable diagnostic tool.

To our knowledge, this is the first time that a combination of targeted and untargeted metabolomics has been used to discover disease indicators in fish. Through integrating proteomics data in a multi-omics approach supported by biostatistical analysis, we could show cross-connections between specific metabolites and proteins, which could be suitable for monitoring HSMI development in farmed salmon. 

## 2. Materials and Methods

### 2.1. Fish Trial and Sampling

Plasma samples obtained from a PRV-1 infection trial in Atlantic salmon [27] were used in the current work for metabolomics analysis. The fish study had been approved by the Norwegian Animal Research Authority (FOR-1996-01-15-23) and performed at the Aquaculture Research Station at Kårvika, Norway. The sample subset selected for metabolomics included 70 fish (previously injected intraperitoneally with 200 µL uninfected salmon blood cell lysate, considered as ‘control’ in the original study). After ten weeks, these fish were divided into two groups of 35 fish, of which one was exposed to PRV-1 through the addition of an equal number of PRV-1-infected shedder fish. The virus strain used for infecting shedder fish originated from the isolate PRV-1 NOR2012-V3621 [8]. The 35 unexposed fish in a parallel tank were used as negative controls. Eight fish from the PRV-1-infected (P) and control (C) groups were sampled after 2 (W2), 5 (W5) and 8 (W8) weeks. For further details, we refer to the initial publication of this trial [27]. During the experiment, no fish died in any of the treatment groups.

At each sampling point, the fish were anesthetized by bath immersion in benzocaine chloride (0.5 g/10 L water), and blood was drawn from the caudal vein into BD Medical Vacutainer heparin-coated tubes (BD Medical, Mississauga, ON, Canada). The blood samples were stored at 4 °C for a maximum of 6 h, centrifuged (3000× *g* for 5 min at 4 °C), and the separated plasma was stored at −80 °C. The collected fish were previously analyzed to confirm the PRV-1 infection (qPCR on spleen) and state of HSMI (heart histology) in the ‘P’ group [27]. Samples from fish before the virus exposure (C0), as well as from the P and C groups at W2, W5, and W8 after virus exposure, were used for the metabolomics analyses in the current work.

### 2.2. Metabolomic Analyses

#### 2.2.1. Targeted Metabolomics Using Standardized Lipid and Metabolic Profiling

Plasma samples were thawed on ice, mixed, and centrifuged with 2750× *g* for 5 min at 4 °C. Targeted metabolomic analysis was performed using the AbsoluteIDQ^®^ p400 HR kit (Biocrates Life Science AG, Innsbruck, Austria), including more than 400 metabolites [28], in accordance with the manufacturer’s instructions. In short, the included internal standard solution (10 µL) was added to a 96-well plate with the exception of the blank before plasma samples (10 µL), calibrations and the provided quality control samples, containing all metabolites at three defined levels (QC1, QC2, and QC3), were loaded. After the plate was dried for 30 min under a gentle stream of nitrogen, the amino acids in the samples were derivatized by the addition of 5% phenyl isothiocyanate solution and incubation for 25 min at room temperature (RT). The plate was dried for 1 h. Subsequently, metabolites were extracted from the samples by shaking with 300 µL 5 mM ammonium acetate in methanol (MeOH) for 30 min at 450 rpm. Samples were centrifuged through a filter plate, and 150 µL of each sample was transferred to an empty plate and diluted 1:1 with Optima LC−MS grade water (Fisher Scientific, Oslo, Norway) for ultra-high performance high-resolution mass spectrometry (UHPLC-HRMS), or added with 250 µL mobile phase for flow-injection analysis (FIA)-HRMS. All analyses were carried out with three biological replicates.

Targeted metabolomics was performed using a Vanquish Horizon UHPLC system coupled to a Q-Exactive HRMS instrument equipped with a heated electrospray interface (HESI-II) (Thermo Fisher Scientific, Bremen, Germany). The determination of the 408 metabolites included in the kit required two different chromatographic approaches. Amino acids and biogenic amines were separated on an octadecylsilane column (ODS; Biocrates) applying a water/acetonitrile (ACN) (both containing 0.2% formic acid; LC-MS grade, Fisher Scientific) gradient with 6 min run time according to the manufacturer’s instructions. Acylcarnitines, monosaccharides (hexoses), di- and triglycerides, lysophosphatidylcholines, phosphatidylcholines, sphingomyelins, ceramides, and cholesteryl esters were analyzed using FIA–HRMS within approximately 3.8 min per sample. The mobile phase was prepared by diluting FIA buffer (Biocrates) with LC−MS grade MeOH (Fisher Scientific) as instructed.

Both the UPHLC- and FIA-HRMS analyses were run using electrospray ionization in the positive ion mode in the different full scan modes in accordance with the methods provided by the manufacturer. The UHPLC-HRMS method used the following source parameters: sheath gas flow rate 60, auxiliary gas flow rate 30, sweep gas flow rate 1, spray voltage 3 kV, capillary temperature 300 °C, S-lens RF level 60, and auxiliary gas heater temperature 550 °C. Xcalibur software version 4.2 (Thermo Fisher Scientific) was used for instrument control and data acquisition. The FIA-HRMS method used the source parameters: sheath gas flow rate 15, auxiliary gas flow rate 5, spray voltage 2.7 kV, capillary temperature 300 °C, S-lens RF level 60, and auxiliary gas heater temperature 120 °C. MetIDQ software (version Oxygen; Biocrates Life Sciences AG) was used for data processing. The results were quantitated using seven-point calibration with internal standards. Instrument performance was controlled by system stability tests.

#### 2.2.2. Untargeted Metabolomics

The salmon plasma was thawed at RT, and 50 µL aliquots were transferred into new tubes. Ice-cold MeOH (150 µL) was added for protein precipitation, which was achieved by mixing for 20 s and precipitation for 20 min at −20 °C. Subsequently, the samples were centrifuged at 13,200× *g* for 10 min at 4 °C, and 130 µL aliquots were transferred to HPLC vials with fixed inserts (Thermo Fisher Scientific). The solvent was evaporated to dryness using a gentle stream of nitrogen at 40 °C. The samples were stored at −20 °C until analysis when they were reconstituted with 50 µL ACN before UHPLC-HRMS analysis.

Untargeted metabolomics was also performed by UPHLC-HRMS. Chromatographic separation was achieved using a zwitterionic SeQuant ZIC-pHILIC column (Merck, Kenilworth, NJ, USA; 150 × 4.6 mm, 5 µm) and a mobile phase consisting of 20 mM ammonium carbonate (pH 8.3; (A)) and ACN (B). After injection of 3 µL sample, metabolites were eluted at a flow rate of 0.3 mL/min, starting with 80% B for 1 min, followed by a linear gradient to 20% B over 29 min, a column flush with 8% B for 5 min, and re-equilibration using the start conditions within 9 min. The injector was flushed with 50% ACN and the seal with 75% isopropanol/25% water/0.1% formic acid.

The mass spectrometer was run in full-scan positive and negative ion modes using fast polarity switching in the mass-to-charge (*m*/*z*) ranges of 58 to 870 and 70 to 870, respectively. The mass resolution was set to 70,000 at *m*/*z* 200. The spray voltage was 2.8 and 3.0 kV (positive and negative mode, respectively), the transfer capillary temperature was 280 °C, and the sheath and auxiliary gas flow rates were 35 and 10 units, respectively. Xcalibur software version 4.2 was used for instrument control.

A pooled QC sample (5 µL of each sample) was prepared in parallel with the test samples and measured periodically (n = 6) during the HILIC-HRMS(/MS) analysis to assess system stability and produce metabolite fragmentation data by using data-dependent acquisition (DDA). The scan ranges were set to (58(70)–230) and (230–870) Da at a default resolution of 70,000, with an AGC target of 1 × 10^6^ with a maximum ion injection time set to 100 ms. HRMS/MS fragmentation spectra were acquired at 17,500 FWHM. The settings for the AGC target, maximum IT and isolation window were set to 5 × 10^5^, 64 ms and 1.4 *m*/*z*, respectively. Normalized collision energies (NCEs) of 35% and 30% were used for the positive and negative modes, respectively. A blank sample was injected repeatedly, and the metabolites detected in the first blank analysis were removed from the peak list compiled from all samples.

### 2.3. Data Processing

#### 2.3.1. Targeted Metabolomics

The raw data from the targeted metabolomic analyses were exported to MetIDQ, validated, quantified, and summarized in a result report. Only metabolites that appeared in at least 80% of the samples in one treatment group of the study were considered. Low abundance metabolites occurring with values < LOD in some samples were kept in the dataset if they occurred at levels > LOD in 70% of all study samples to not miss potentially interesting metabolites in the dataset. Missing values for some metabolites in occasional samples (relevant for 3.6% of the complete dataset) were imputed with one-fifth of the minimum positive values for the respective metabolite. Subsequently, the raw data were normalized to correct for potential technical variabilities of the analyses by using metabolite-specific correction factors that were based on the intra-plate precision of QC2 (n = 5) and generated by dividing the median QC2 concentration of each metabolite with the target concentration for this metabolite as specified in the MetIDQ database. Afterward, the measured metabolite concentrations in the study samples (>LOD) were divided by the respective metabolite-specific correction factors. Metabolites with a relative standard deviation (RSD) > 30% between the five QC2 were excluded from the curated targeted metabolomics dataset.

#### 2.3.2. Untargeted Metabolomics

The Compound Discoverer (CD) 3.3 SP2 software (Thermo Fisher Scientific, Waltham, MA, USA) was used to process the raw data of the untargeted metabolomic analyses (Table S1). Retention times were aligned with an adaptive curve algorithm followed by peak picking, considering a 5 ppm mass tolerance, 500,000 minimum peak intensity, and the following ions: [M+H]^+^, [M+NH_4_]^+^, [M+Na]^+^, [M+K]^+^, [M+ACN+H]^+^, [M+Cl]^−^, [M+MeOH+H]^+^, [M+H−H_2_O]^+^, and [M+H−NH_3_]^+^and [M−H]^−^. 

Metabolite peak areas were integrated into the extracted ion chromatograms of the respective most common ions, preferably [M+H]^+^ and [M−H]^−^, with mass tolerance set to 5 ppm and retention time tolerance to 0.2 min. Only peaks with an Original Peak Rating of at least 40% of the respective maximum values in at least 6 study samples were considered for further processing. Missing values were imputed by using the gap-filling function with the RealPeak detection algorithm from CD [29]. The variation within the QC samples was accessed and corrected by applying the Systematic Error Removal using the Random Forest (SERRF) normalization algorithm, interpolating peak values with non-linear regression [30]. The maximum QC RSD allowed before correction was set to 50% and after correction to 25%. Metabolites detected in solvent controls at signal areas exceeding 20% of the values measured in the QC were eliminated. The subsequent data processing was performed using MetaboAnalyst 5.0 (https://www.metaboanalyst.ca/; accessed on 23 February 2024) and SIMCA 16.0 (Sartorius Stedim Biotech, Umeå, Sweden). The data were normalized to the total median of peak values in each sample, log-transformed and Pareto-scaled [31], resulting in the curated untargeted metabolomics dataset.

#### 2.3.3. Statistical Analyses

The curated targeted and untargeted metabolomics datasets were evaluated in MetaboAnalyst 5.0 using univariate and multivariate statistical analyses, including *t*-test (*p* < 0.05) and Volcano plots (fold-change (FC) cut-off > 2; false discovery rate (FDR)-adjusted *p*-value ≤ 0.1) [31] with the aim of determining significantly changed metabolite levels between control and PRV-1-challenged fish. 

Multivariate modeling of the Pareto-scaled data by unsupervised principal component analysis (PCA) was performed to assess the general distribution of the metabolite contents in the study samples and to identify potential outliers. Supervised orthogonal partial least-squares discriminant models (OPLS-DA) (with 100 permutations) were built to find metabolites with discriminating power between control and PRV-1-challenged fish. The models were considered to deliver significant results when the determined scores for the total explained variance (R^2^X), goodness of fit (R^2^Y) and predictive ability (Q^2^) were sufficiently high (maximum value 1) and the difference R^2^Y−Q^2^ relative to R^2^Y (Δ%R^2^Y−Q^2^) was below 30% [32]. Cross-validation using ANOVA (CV-ANOVA) was performed to evaluate the reliability of the models, and *p*-values ≤ 0.05 were considered significant when the permutation test was valid. Metabolite enrichment analysis based on the quantitative targeted metabolomics data was performed using the enrichment analysis module in MetaboAnalyst 5.0 based on 15 libraries containing about 13,000 metabolites. The module for biological pathway analysis was used for the untargeted metabolomics data, showing the connection of metabolites substantially changed by the PRV-1 infection to specific KEGG (Kyoto Encyclopedia of Genes and Genomes; https://www.genome.jp/kegg/pathway.html; accessed on 26 February 2024) pathways established for zebrafish (*Danio rerio*). Metabolites in the salmon plasma measured by the untargeted analysis were tentatively identified by molecular mass, *m*/*z* ion, polarity and comparison to the metabolites that had been characterized in the QC samples by their MS^2^ product ion data. Data were converted into the open-source format *.mzml and imported into SIRIUS, applying CSI:FingerID for metabolite predictions [33]. When reliable annotations could not be achieved, the metabolites were processed with the automated class and ontology prediction tool CANOPUS, which assigns molecules with a chemical fingerprint and chemical class in accordance with the ClassyFire ontology [34,35].

### 2.4. Multi-Omics Analysis

#### 2.4.1. Datasets Included in the Multi-Omics Analysis

In a multi-omics approach, the targeted and untargeted metabolomics data were combined with proteomics data that had been measured in the P5, C5, P8 and C8 groups (n = 7 samples/group) of the PRV-1 challenge experiment [36]. Only corresponding metabolomic data were used. The three omics-datasets were considered as blocks in the multi-omics analysis, containing n = 263 entries in the targeted metabolomic block, n = 779 entries in the untargeted metabolomic block, and n = 646 entries in the proteomic block. 

#### 2.4.2. Multi-Block Sparse Partial Least-Squares Discriminant Analysis (sPLS-DA)

Data Integration Analysis for Biomarker discovery using Latent cOmponents (DIABLO) for omics studies, also referred to as multi-block sPLS-DA, was used for combing the metabolomic and proteomic datasets [36]. The time aspect of the experiment, week 5 versus week 8, was not considered, so the data of, respectively, C5 and C8, as well as P5 and P8, were combined (n = 14 samples for each treatment, i.e., controls and PRV-1 challenged salmon). The DIABLO algorithm relies on the identification of a limited number of correlated variables from multiple datasets to predict an outcome, here the PRV-1 infection and HSMI development [37,38,39]. The design matrix is a Q × Q matrix with values ranging from 0 to 1, representing whether and by how much each dataset is correlated in the DIABLO analysis. We started the analysis of the salmon plasma omics data by assembling pairwise sPLS (PLS2) models, in which correlation coefficients were used as reference values for the design matrix. After the design matrix was assigned, we defined a DIABLO model with two components. The model was fitted and included all variables determined in the three omics data blocks. Global performance was assessed using a 10^6^-fold cross-validation. Applying the iteration and cross-validation algorithms of the mixOmics package, the optimal number of components and an optimal number of variables were determined for each data block [38]. The optimized, final DIABLO model delivered the optimal number of components obtained by the modeling, score vectors for the included samples that allow for the plotting of their spatial distribution, and a list of selected variables (proteins, metabolites) from each data block that are associated with each component. The values of the calculated loading coefficients of the different variables illustrated their respective importance in DIABLO for the description of the optimal components [38]. The results were presented in a Circos diagram that is built on a similarity matrix and represents the correlation between variables from different data blocks [40]. A cut-off of r > 0.75 was set to focus on significant variables.

## 3. Results

The salmon plasma for the metabolomic analyses was sampled from a previous, well-described PRV-1 challenge study, which included analyses of PRV-1 levels in the spleen and heart histology [27]. Infected fish were all PRV-1 positive and developed HSMI at W8. The control group samples were all virus-negative. 

### 3.1. Targeted Analysis Using the AbsoluteIDQ^®^ p400 HR Kit

Curation of the targeted metabolomics raw data with the set quality parameters resulted in the determination of 263 ascertained metabolites (Table S2a) that were further processed with different statistical analyses. The median concentrations (µM) for each metabolite in the seven different treatment groups (C0, C2, P2, C5, P5, C8 and P8) showed that salmon plasma contains high levels of some lipids, amino acids and hexoses (Table S2b). The most abundant lipids in control fish from the beginning of the experiment (C0) were cholesteryl esters (CE (22:6), 20 mM), phosphatidylcholines (PC (34:1), 0.59 mM), sphingomyelins (SM (42:2), 0.48 mM) and triglycerides (TG (54:3, 0.45 mM) (Table 1). The highest detected levels for lysophosphatidylcholines (LPC (22:6), 92 µM), ceramides (Cer (42:2), 28 µM), diglycerides (DG (39:0) and acylcarnitines (AC (0:0), 4.0 µM) were notably lower. Glycine (Gly, 1.1 mM) and taurine (1.4 mM) were, respectively, the amino acid and biogenic amine with the highest plasma concentrations, whereas the sum of hexose sugars (H1, 4.6 mM) was also considerably high.

**Figure 1:**
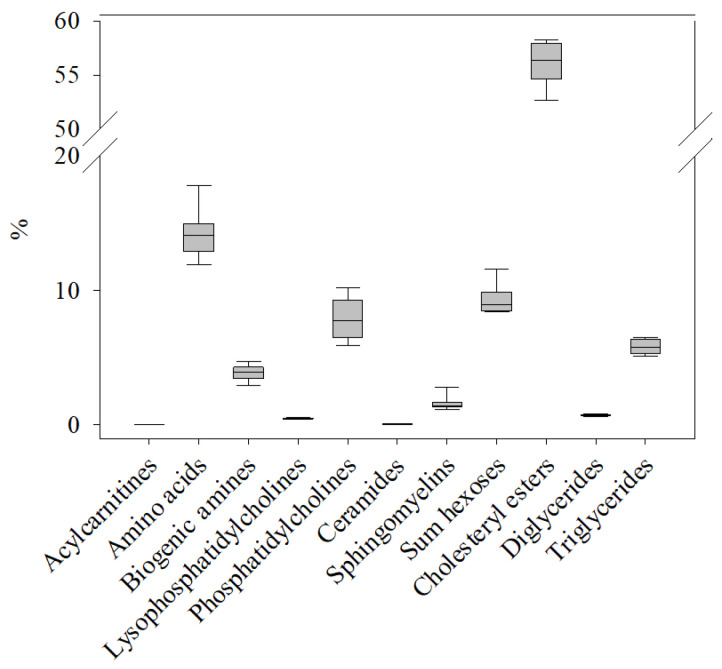
Relative occurrence of metabolites in the 11 compound classes (Table 1) included in the AbsoluteIDQ^®^ p400 HR kit in the plasma metabolome of healthy control salmon (C0). The sum of concentrations for the metabolites in each class was normalized to the total concentration of all metabolites determined in the same sample. The box plot shows the variation and median value for all metabolites within that compound class in n = 8 samples.

The occurrences of the different compound classes (Table 1) at C0 were determined by summarizing the respective metabolite concentrations (Table S2a) and expressing these sums as ratios of the total metabolite plasma concentration in each sample (Figure 1). The results showed that cholesteryl esters (56.6%) were by far the most prevalent metabolites. Second were amino acids (13.8%), followed by hexoses (9.4%). The compound classes with the lowest percentage in salmon plasma were acylcarnitines (0.02%), ceramides (0.06%) and diglycerides (0.74%). 

**Figure 2:**
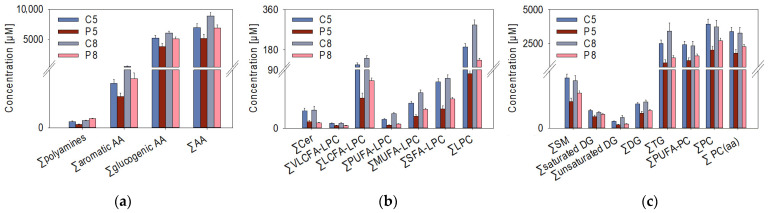
Plasma concentrations of metabolites (n = 8 samples per treatment group) that were significantly (*t*-test, *p* < 0.05) different between control (C) and PRV-1-challenged (*p*) salmon in both week 5 and 8, summarized in compound classes: (**a**) sum of polyamines, aromatic amino acids (AA), glycogenic AA, all AA; (**b**) sum of ceramides (Cer), very-long-chain fatty acid (VLCFA)-lysophosphatidylcholines (LPC), long-chain fatty acid (LCFA)-LPC, polyunsaturated fatty acid (PUFA)-LPC, monounsaturated fatty acid (MUFA)-LPC, saturated fatty acid (SFA)-LPC; (**c**) sum of sphingomyelins (SM), saturated diglycerides (DG), diglycerides (DG), triglycerides (TG), polyunsaturated fatty acid-phosphatidylcholines (PUFA-PC), phosphatidylcholines (PC), diacyl-phosphatidylcholines (PC (aa)).

While there was little difference at W2 (C2 vs. P2), only 36 of the detected metabolites showed significant concentration changes, significant differences (*t*-test, *p* < 0.05) were observed for 195 metabolites at W5 (C5 vs. P5), and for 134 metabolites at W8 (C8 vs. P8) (Table S2b). Grouping the total 96 metabolites that were significantly changed at both W5 and W8 with regard to compound classes, time- and PRV-1 infection-dependent changes in the plasma metabolite profiles became visible (Figure 2). Generally, an increase in metabolite concentrations from W5 to W8 was observable for almost all compound classes. This potential age impact was substantial, shown by the strong positive correlation (Pearson’s correlation coefficient *p* > 0.6) between the lipid compound classes, e.g., ceramides, lysophosphatidylcholines, diglycerides, triglycerides, and cholesterol esters.

Significant differences associated with the infection state of the salmon were found for several biogenic amines (spermidine, putrescine, taurine), aromatic (phenylalanine), and glucogenic amino acids (aspartate, glycine, serine, methionine), and the sum of amino acids at W5 and W8 (Figure 2a). Notably, the concentrations of several amino acids (alanine, proline, tyrosine) and branched-chain amino acids (leucine, isoleucine, and valine) were already significantly changed at W2 (data not shown). Regarding the plasma lipids, the PRV-1 infection led to a considerable decrease in the plasma concentrations of free lipid molecules at W5 and W8 (Figure 2b,c). A more detailed analysis of the lysophosphatidylcholine (LPC) subclasses revealed that very-long-chain fatty acid (VLCFA)-LPC and polyunsaturated fatty acid (PUFA)-LPC were considerably decreased both in P5 and in P8 relative to their controls. Furthermore, the virus infection had a major effect on the ceramide and unsaturated diglyceride levels at the two time points.

#### 3.1.1. Univariate Analysis of the Targeted Metabolomics Data

**Figure 3:**
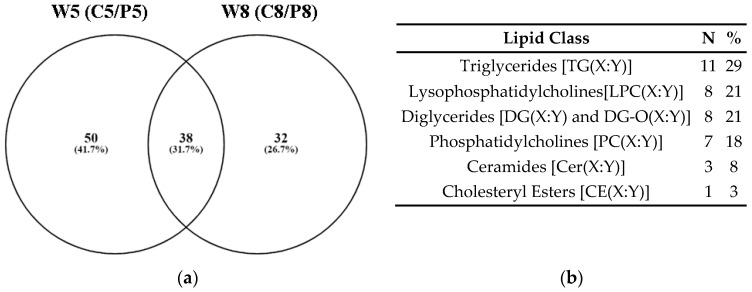
(**a**) Venn diagram showing numbers of metabolites that were significantly different in the plasma of control and PRV-1-infected salmon at W5 and W8; (**b**) The 38 metabolites that were identical at both time points (Table S3) belonged to six lipid classes. The possible combinations in the side chain lengths and saturation of fatty acids are indicated as (X:Y) (Table 1).

Progressing from the descriptive analysis of the metabolome data, univariate volcano plots were built to find metabolites that contributed significantly to the observed differences in the metabolite plasma profiles between control and PRV-1-challenged salmon at W5 and W8 (Figure S1a,b). Among the, respectively, 88 and 70 significantly different metabolites for C5/P5 and C8/P8, 38 were shared between both time points (Figure 3a). 

The 38 metabolites with significantly changed relative concentrations at both W5 and W8 were exclusively lipids (Figure 3b; Table S3), illustrating the involvement of lipid metabolism in the PRV-1-induced disease development. Out of the total 38 shared metabolites, 12 and 8 compounds belonged to the top 20 significantly changed metabolites in the volcano plot analyses of the C5/P5 and C8/P8 comparisons, respectively (Figure S1a,b). The lipids PC(32:3), LPC(20:4), DG(34:3), DG(34:1), and PC(44:10) were especially linked to differences between healthy controls and infected fish at both study time points (Table S3). 

#### 3.1.2. Multivariate Analysis of the Targeted Metabolomics Data

**Figure 4:**
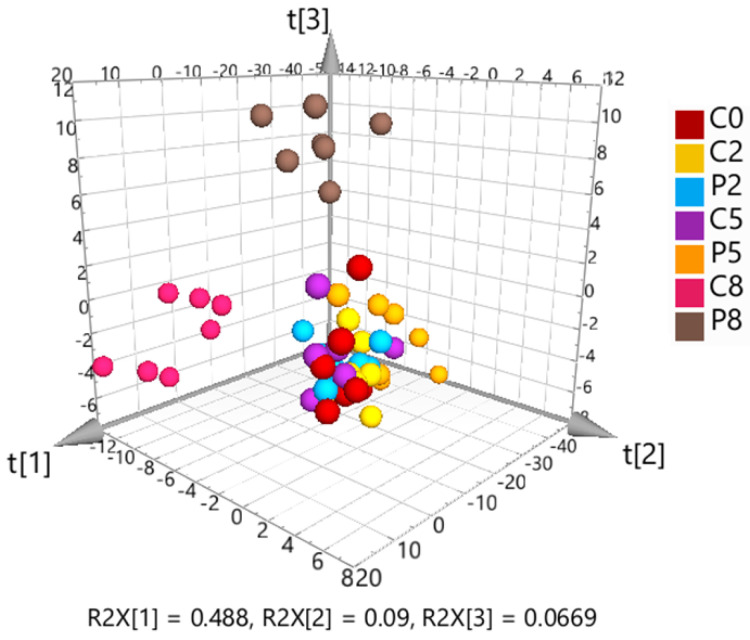
3D scores plot from unsupervised principal component analysis (PCA) of the Pareto-scaled and log-transformed targeted metabolomics data. The first three components explain 65% of the total variation.

The targeted metabolomics dataset was further analyzed by multivariate principal component analysis (PCA) to identify clustering patterns (Figure 4). The 3D PCA scores plot showed a visible separation of C5 and P5 samples, a clear separation of C8 and P8 samples, but no discernible difference between C0, C2 and P2 samples.

Subsequently, OPLS-DA models were calculated to determine the validity of pairwise combinations, which were evaluated not only based on the determined scores (R^2^X, R^2^Y, Q^2^) but also on the cross-validation of variance (CV-ANOVA) and permutation testing (Table 2).

The OPLS-DA model comparing the C2 and P2 treatment groups at W2 delivered a CV-ANOVA *p*-value at the border of significance, and the permutation test was valid. Nevertheless, considering the significance criterion Δ%R^2^Y−Q^2^ < 30%, the separation between the groups was not considered significant [32]. The OPLS-DA model was stronger for W5 (C5 vs. P5), showing a significant difference in the metabolite contents of control and PRV-1-infected fish. At W8 (C8 vs. P8), the significance was further increased, showing a clear differentiation between healthy and infected salmon. 

Enrichment analysis of the metabolite concentration data showed that discriminant plasma metabolites responsible for this separation belonged to major lipid metabolism pathways. Regarding the differences between C5 and P5, metabolites that are part of the sphingolipid metabolism and arachidonic acid metabolism were overrepresented in the metabolome at levels not expected by chance (Figure S2a). At W8, metabolites connected to phospholipid synthesis and arachidonic acid metabolism were among the most relevant lipids for the differentiation between C8 and P8 samples (Figure S2b).

### 3.2. Untargeted Metabolomics

Untargeted metabolomics of the salmon plasma using HILIC-HRMS resulted in the detection of 779 compounds in the curated dataset (Table S4). Multivariate analyses by PCA and OPLS-DA were applied to study the data structure and discern discriminant metabolites.

#### 3.2.1. Multivariate Analysis of the Untargeted Metabolomics Data

**Figure 5:**
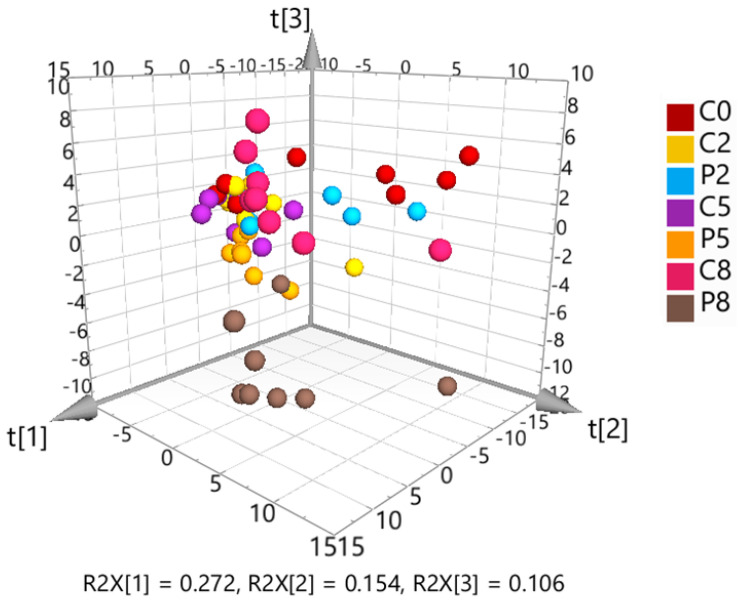
3D scores plot resulting from unsupervised principal component analysis (PCA) of the solvent control-corrected, median-normalized and Pareto-scaled untargeted metabolomics data. The first three components explain 53% of the total variation.

The initial PCA, including all samples, showed close clustering of the solvent controls and QC samples, demonstrating good data quality. Hotelling’s T-squared distribution testing identified one C2 sample as an outlier, which was removed from the subsequent data analysis. The 3D PCA scores plot (Figure 5) indicated a slight separation of controls and infected fish at W2, whereas the differentiation was clearer at W5 and W8.

The observed trends were further investigated by building supervised OPLS-DA models to compare the metabolomes of control and PRV-1-infected salmon at the three sampling time points (Table 3). 

The scores showed a result similar to that of the targeted metabolomics OPLS-DA analysis. The model comparing C2 and P2 samples returned a CV-ANOVA *p*-value below 0.05 and a valid permutation test. However, the significance criterion Δ%R^2^Y−Q^2^ < 30% was not fulfilled [32]. Therefore, it was assumed that the model lacked validity, and the separation between the treatment groups at W2 was therefore considered not significant. The corresponding model for W5 was accepted as significant but was, according to Δ%R^2^Y−Q^2^ = 25%, still not very strong, which reflected the only moderate differentiation between C5 and P5 in the PCA scores plot (Figure 5). In contrast, the distinction between C8 and P8 was significant in the OPLS-DA model (Table 3) and visible in the PCA scores plot. 

#### 3.2.2. Biological Pathway Analysis of the Untargeted Metabolomics Data

**Figure 6:**
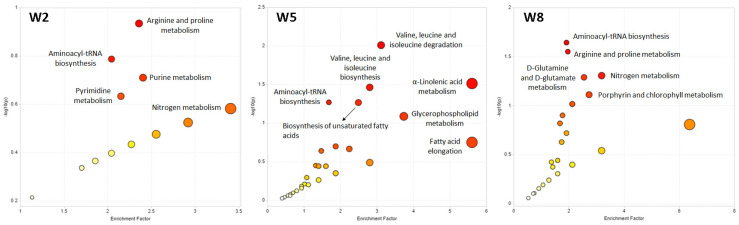
Pathway analysis of the untargeted metabolomics data, comparing metabolite profiles in the plasma of control and infected salmon at W2, W5, and W8. The pathway impact is presented as a combination of its significance (*p*-value) and enrichment factor. High impact values show the relative importance of the pathway, meaning that several of the metabolites involved have been detected in the respective salmon plasma samples. The circle size indicates the impact and the color of the significance of a pathway (a low *p*-value is represented by an intense red color).

Pathway analysis of the untargeted metabolite dataset was performed for W2, W5 and W8 based on the metabolic KEGG network for zebrafish (*Danio rerio*) to identify biological processes that were affected by the progressing PRV-1 infection and HSMI development in the virus-challenged salmon (Figure 6).

Metabolites differentiating between healthy controls and PRV-1-infected fish at W2 mainly belonged to amino acid and nucleotide pathways (Figure 6). At W5, several lipid metabolism pathways were activated, in particular, the glycerophospholipid metabolism, fatty acid elongation, α-linolenic acid metabolism, and biosynthesis of unsaturated fatty acids (Figure 6). Interestingly, the porphyrin metabolism, including metabolites such as haem, was significantly affected at W8 (Figure 6), in addition to amino acid pathways that were already noticeable at W2. 

### 3.3. Multi-Omics for the Integration of Targeted and Untargeted Metabolomics Data with Proteomics Data Available for the Same Salmon Plasma Samples

The three data blocks included in the multi-omics analysis contained, respectively, n = 263 metabolites determined by targeted metabolomics (Table S2a), n = 779 metabolites detected by untargeted metabolomics (Table S4), and n = 646 proteins identified by proteomics [36] (Table S3). Since proteomics data were only available for W5 and W8, the corresponding C5, P5, C8 and P8 samples were selected from both metabolomics datasets for the multi-omics analysis. The control (C5 + C8) and PRV-1-infected (P5 + P8) samples were considered together as two study groups, disregarding the time factor in the study, to increase the statistical power of the analysis by higher sample numbers per group. 

#### 3.3.1. Correlation Analysis between the Data Blocks Using PLS2

The extent of correlation between the measured metabolites and proteins was accessed by assembling pairwise PLS2 models considering the entire datasets, analyzing targeted vs. untargeted metabolomics data, targeted metabolomics vs. proteomics data, and untargeted metabolomics vs. proteomics data. The respective three PLS2 models had correlation values above 0.7 (data not shown), indicating a substantial correlation between the data blocks. The calculated correlation coefficients were used as reference values for generating the data matrix on which the subsequent DIABLO analysis was built.

#### 3.3.2. Determination of Relevant Components for Describing the Optimal DIABLO Model

Based on the positive correlation found in the PLS2 assessment, a full design model with an assigned value of 1 for the data matrix was chosen for the multi-block DIABLO analysis. The initial DIABLO model contained all variables from each block. It was started with two components (Latent Variables) in accordance with the recommendation to use the same number, as there are treatment groups in the study, in this case, C and P samples. Applying the Internal Functions algorithm of DIABLO, iteration and cross-validation of the data generated the final model with the optimized number of components and significant variables (metabolites, proteins) for each component.

**Figure 7:**
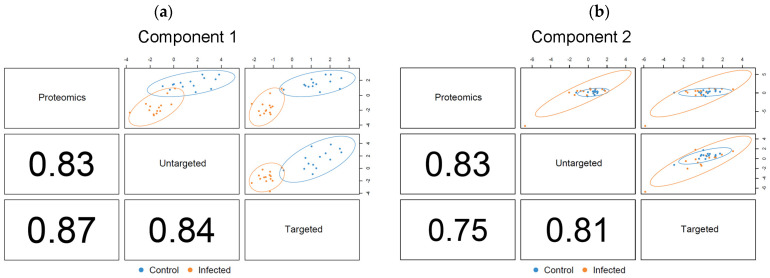
DIABLO correlation plots showing correlation factors and extent of treatment group separation for the optimized (**a**) component 1, and (**b**) component 2. Confidence ellipse plots (95%) are shown. The numbers indicate the correlation coefficients between the first or second components of each data block, respectively.

The optimized DIABLO model for the salmon metabolomics and proteomics data contained two components (Figure 7). The calculated correlation values of both component 1 and component 2 were in good agreement with the outcome of the PLS2 fitting, showing a strong correlation across the three datasets. The result also confirmed that the initial values for the DIABLO matrix were accurately derived from PLS2. The first component of the optimized model was sufficient to discriminate between the plasma contents of control and PRV-1-infected salmon (Figure 7a), matching the ideal outcome of the model. Interpretations were accordingly based on the variables identified as discriminant for component 1. Component 2 (Figure 7b) indicated variables that contributed to the separation within the two treatment groups, including the time aspect, which was confirmed as of minor importance for the differentiation between infected and healthy fish.

Each DIABLO component included five metabolites from the targeted dataset, five metabolites from the untargeted dataset, and five proteins (Table 4). The calculated overall error rate was 0.06 in component 1 and 0.08 in component 2, indicating that less than 10% of the samples were misclassified using the DIABLO model. The corresponding loading plots (Figure S3a,b) showed the relative importance and direction of change for each variable in the differentiation between the two study groups in each data block. 

Several of the variables identified as discriminant in the multi-omics analysis had already been shown to be important in the targeted metabolomic analysis, specifically PC(32:3), PC(32:4), and SDMA (Figure S1a,b). The variables of interest in the untargeted metabolomics block were tentatively annotated in silico [34] (Table 4). Among the identified metabolites were ADMA and serine, which had also been found to be decisive in the targeted metabolomics dataset. Interestingly, three of the most relevant variables determined in the proteomics block, e.g., galectin-3-binding protein, fucolectin-6, and ryanodine receptor-3, had been identified as potential biomarkers for PRV-1 infection and HSMI development in our recent proteomics study [36]. 

**Figure 8:**
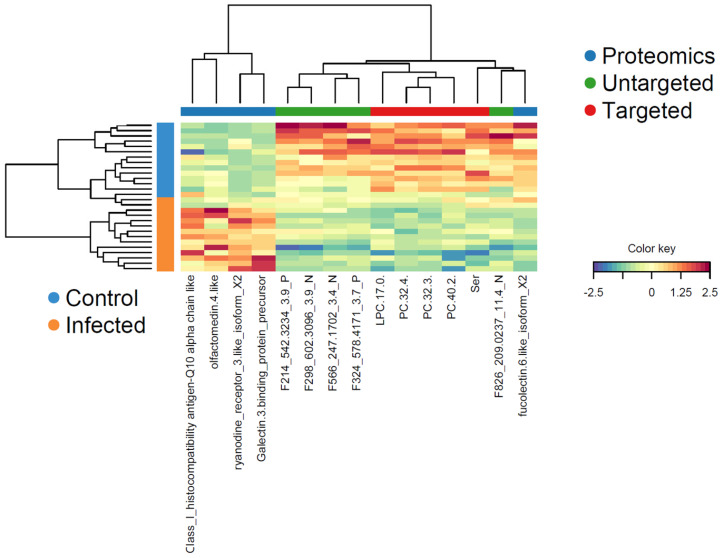
Clustered image map (heatmap) for the variables selected by multi-block DIABLO for component 1. Samples (color-coded for control and PRV-1-infected) are presented in rows, and the selected variables (with reference to the three data blocks) are in columns. The color key indicates the relative increase or decrease of a variable in a sample.

The multi-omics profiles of the significant variables in component 1 in each sample showed a clear clustering with regard to the two treatment groups (Figure 8). All proteins, with the exception of fucolectin-6, had increased plasma levels in the PRV-1-infected salmon, whereas the different lipid metabolites and serine were decreased. 

**Figure 9:**
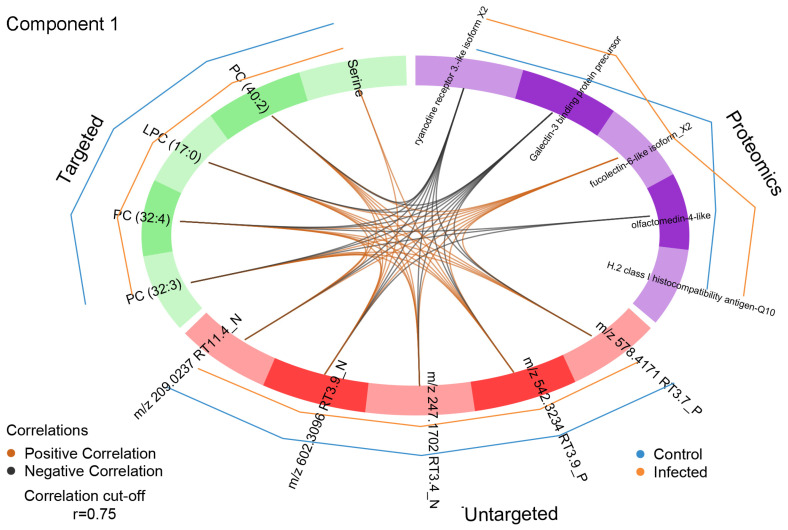
Circos plot of variables identified as a discriminant in component 1 of the proteomics, targeted and untargeted metabolomics data blocks. Only correlations above the threshold r = 0.75 are considered. The internal connecting lines show positive (orange) or negative (black) correlation between variables in the different data blocks. The outer lines indicate the relative level changes of each variable in the treatment groups.

Correlations between the variables in component 1 in the three data blocks and their directions (positive or negative) were made visible in a Circos plot (Figure 9). Using a cut-off r = 0.75 ensured that only strong correlations were shown. This resulted in the exclusion of H-2 class I histocompatibility antigen-Q10 in the proteomics block from the cross-correlation. The outer lines in the Circos plot expressing the relative occurrences of the variables in the plasma of control and PRV-1-infected fish confirmed the changes already observed in the heatmap. Apart from four proteins, the levels of all other variables were decreased in infected salmon. Consequently, galectin-3-binding protein, ryanodine receptor-3, and olfactomedin-4 were negatively correlated with the lipid metabolites, whereas fucolectin-6 was positively correlated. Serine was only correlated to lipid metabolites but not to any of the proteins, weakening its reliability as an indicator for the PRV-1-initiated physiological changes in the salmon. 

**Figure 10:**
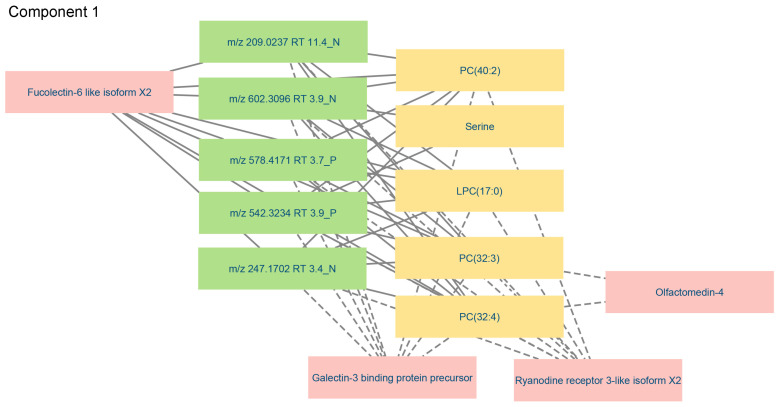
Relevance network visualizing relevant connections (cut-off > 0.75) between significant variables in component 1 of the proteomics, targeted and untargeted metabolomics data blocks. The colors of the nodes represent the different data blocks: yellow—targeted metabolomics; green—untargeted metabolomics; pink—proteomics. Solid lines indicate that plasma level changes were in the same direction, and dashed lines indicate changes in the opposite direction when comparing variable levels in the plasma of control and PRV-1-infected salmon.

The correlations between variables in component 1 in the three data blocks were visualized with more detail in a relevance network (Figure 10). Only connections above a threshold value >0.75 were shown to restrict the plot to the most relevant variables. The network analysis clearly illustrated that galectin-3-binding protein, ryanodine receptor-3 and fucolectin-6 were highly associated with PC and LPC in the targeted metabolomics data block and with all variables in the untargeted metabolomics data block. In contrast, olfactomectin-4 was only connected to the targeted metabolites PC(32:3) and PC(32:4). As previously observed, serine was only connected to two variables detected by untargeted metabolomics, of which *m*/*z* 602.3096 RT 3.9 N had been tentatively annotated as phosphatidylserine (Table 4). The loading weight for serine in the model was low, meaning that its inclusion had little influence on the outcome.

The discriminant variables in component 2 had exclusively positive connections (Figure S4). Noticeably, ADMA was connected to all proteins and all untargeted metabolites. Likewise, *m*/*z* 203.1499 RT 22.2 P, provisionally annotated as ADMA, was extensively cross-connected, i.e., to all proteins and targeted metabolites. The two histone-3 proteins were associated with the untargeted metabolites and SDMA. The network analysis thus proved both the relevance of the selected variables and their extensive connections across the three data blocks. The mutual confirmation of variables shown in the multi-omics approach increased their credibility as potential biomarkers for PRV-1 infection and HSMI development in salmon. 

## 4. Discussion

Monitoring the presence of the ubiquitous marine virus PRV-1 in salmon farms is not predictive of the risk of a disease outbreak [25]. Thus, the identification of indicators for the development of HSMI in the fish would provide better diagnostic tools. In the present study, we have therefore investigated infection-induced changes in the plasma metabolome and the inter-relationship between metabolome and proteome in samples from a controlled PRV-1 challenge/HSMI experiment in Atlantic salmon [27,36]. The time points included in our omics analyses were representative of the early stage of the infection (W2), the peak level of virus replication in red blood cells (W5), and fully developed HSMI (W8) [20,21,22,23,24]. With this study set-up, we intended to increase the chances of finding relevant biomarkers for PRV-1-driven disease development in the fish.

The targeted metabolomic analysis affirmed the presence of 263 metabolites among the compound groups included in the AbsoluteIDQ^®^ p400 HR Biocrates kit. This standardized application for broad lipid and metabolite profiling has been originally validated for human plasma, but we have applied it successfully also to salmon plasma, skin and gill mucus in a study comparing the metabolomes in different body fluids [41]. Our results confirmed the general species-independency of physiological metabolites, allowing the use of reference kits. A kit can only cover a fraction of the total metabolome (Biocrates kit contains 408 metabolites of the currently 3408 endogenous metabolites in the molecular weight range of 50 to 1500 Dalton listed in the human metabolome database HMDB version 5.0 [42,43]). Nevertheless, the targeted metabolomics results are valuable because they provide unambiguous metabolite identification, quantification, and comparison of treatment groups. The p400 kit covers a wide range of metabolic pathways, among others, those involved in inflammation, oxidative stress, fatty acid oxidation and signal transduction, which are especially relevant for PRV-1-caused HSMI development.

Lipid metabolism plays an essential role in the progression of the PRV-1 infection since we found notable differences in the plasma lipid profiles of W5 and W8. Generally, the different lipid classes, especially the cholesteryl esters, occurred at considerably high levels in the salmon plasma but were significantly reduced along with the development of HSMI. This was noticeable even against the backdrop of an overall age-dependent increase of metabolite concentrations and a high baseline lipid plasma level in farmed salmon, which was reported to result from the large proportion of vegetable oils in commercial feeds [44]. 

The importance of cellular lipids in viral infection is linked to replication mechanisms such as fusion to the host cell membrane at entry, particle maturation and viral progeny transport by attachment to lipoprotein complexes [45]. A study on the infective process with high-resolution Raman spectroscopy in time-lapse experiments on living cells revealed multiple virus interactions with the host’s cellular machinery and showed the hijacking of energy-generating metabolic pathways and an impact on lipid profiles [46]. The metabolic alterations required for virus replication and virion production involve—among others—changes in the carbon source utilization and modification of the fatty acid, sugar, and amino acid metabolisms [47]. Thus, viruses depend on and shift the host lipidome throughout the viral replication cycle [48]. Circulating lipids are crucial in the pathogenesis of viruses, and the induced changes can lead to dyslipidemia, such as decreased plasma levels of low-density (LDL) and high-density (HDL) lipoprotein cholesterols [49]. This is in accordance with our observation of a sharp decline in the plasma levels of important lipoproteins in PRV-1-challenged salmon in the preceding proteomic analysis that had been performed using P5 and P8 samples of the same fish trial [27,36]. 

The current metabolomics experiment showed that PRV-1 infection of the salmon had a particularly high impact on the plasma levels of ceramides, lysophosphatidylcholines (LPC), and triglycerides (TG). Ceramides (Cer) are lipid messengers involved in sphingolipid metabolism pathways, with a key role in regulating the physical properties of biological membranes, including the formation of membrane microdomains that enable viral entry into host cells [50]. Metabolites classed as Cer consist of a sphingosine backbone connected to various acyl chains. The addition of phosphocholine yields sphingomyelins, the most abundant eukaryotic sphingolipid and integral part of cell membranes. The results of our enrichment analysis of the targeted metabolite concentration data indicated a major impact of the virus infection on sphingolipid metabolism, suggesting that PRV-1 exploits host cell lipid pathways similarly to the mechanism observed for human immunodeficiency virus (HIV-1) and influenza virus A [50]. In accordance with our findings in the PRV-1-challenged salmon, a lipidomics study analyzing the consequences of vesicular stomatitis virus (VSV) infection on the lipid profile of mammalian cells identified LPC as an important target [51]. In that study, the incorporation of LPC in the VSV virion coincided with host cell LPC depletion. Since LPCs are vital for membrane composition and curvature and function additionally as signaling molecules in pathways regulating apoptosis, inflammation and oxidative stress, the virus-induced depletion of host LPC can lead to physiological imbalance and trigger disease development. Both the PUFA-LPC and MUFA-LPC plasma levels were substantially reduced in PRV-1-infected salmon, which was consistent with the lipid composition changes determined in HIV patients [52]. 

As observed in our study on PRV-1 in salmon, the virus-induced remodeling of the lipid metabolism can also affect the triglyceride (TG) plasma levels. In a study in transgenic mice with hepatitis C virus (HCV), the plasma secretion of TG and apolipoprotein B100 was decreased [53], which was subsequently confirmed in HCV-infected humans, indicating that the virus assembly required TG-containing lipoprotein complexes [54]. Our finding of a comparable mechanism in PRV-1-challenged salmon ([36], and present study) underlines the similarity in the virus replication machinery across different vertebrate host species and shows the applicability of proteomics and metabolomics to elucidate virus-induced physiological changes in an organism.

The untargeted metabolomic analysis of the study samples revealed a gradually increasing difference in the plasma profiles between control and PRV-1-infected salmon. Tentative annotation and affected pathway analysis established lipid metabolism and amino acid metabolism (particularly the arginine and proline pathways containing metabolites such as spermidine and putrescine) as the main drivers of the divergence. Moreover, the porphyrin metabolism, including haem, was considerably changed, especially in W8 during HSMI manifestation. Interestingly, our proteomic analysis of the same samples had shown virus-dependent effects on blood homeostasis, including a decrease of haem-binding lipocalins and the iron-storage protein ferritin [36], which was also supported by a transcriptomics study of hearts from PRV-1-infected salmon [55].

The strong correlation between the two metabolomic and the proteomic datasets in the optimized DIABLO model was based on five variables in each block that together were sufficient to define the differentiation between the plasma protein and metabolite profiles of treated and untreated fish.

Among the decisive five metabolites in the targeted dataset were phosphocholine lipids, of which PC(32:3) and PC(32:4) had already been identified in the individual analysis. Remarkably, the same PCs were reduced by hepatitis B virus infection in mice in a study showing the impact of liver disease development on the PC composition in hepatocytes [56]. Moreover, LPC(17:0) was significantly decreased in the plasma of patients with emerging chronic liver failure caused by the hepatitis B virus, showing comparable virus-related lipid deregulation [57]. Serine was the only non-lipid metabolite defined as significant in the targeted metabolomics block. It is a nonessential polar amino acid that is a precursor for the synthesis of the two serine-derived lipid classes, phosphatidylserine and sphingolipids and incorporated into cell membranes by serine incorporator (SERINC) family proteins [58]. SERINC are involved in defense mechanisms against RNA and DNA viruses by inhibiting viral replication and fusion, as has been shown for HIV. The decrease of free plasma serine in PRV-1-infected salmon could thus point to increased SERINC activity caused by the progressing infection. Supporting this hypothesis, the putative annotation of phosphatidylserine (one of the five relevant metabolites in the untargeted metabolomics dataset) corroborated the involvement of serine-containing phospholipids as part of the host response [59]. The other decisive untargeted metabolites were lipids, again underlining the importance of PRV-1-induced changes in the salmon plasma lipidome.

The proteomics dataset was narrowed down by the multi-omics analysis to five significant proteins. Of these, H-2 class I histocompatibility antigen-Q10 was subsequently removed because of the lack of correlation with the decisive metabolites determined in the Circos plot and relevance network analyses. Out of the remaining four proteins, galectin-3-binding protein (Gal-3BP), fucolectin-6 (FL-6), and ryanodine receptor 3 (RyR-3) had already been identified as potential biomarkers for developing HSMI in the preceding proteomics study [36]. Gal-3BP is a glycoprotein with multiple physiological functions, such as the activation of proinflammatory signal cascades during progressing infections and the regulation of blood lipoprotein levels [60,61]. FL-6 is a fucose-binding lectin in teleost fish that is involved in self/non-self-recognition and, thus, an interesting target in the context of PRV-1 infection [62]. Both Gal-3BP and FL-6 showed multiple connections with the significant targeted and untargeted metabolites in the Circos plot and network analysis, underpinning their relevance as indicators for HSMI development in salmon. 

RyR-3 occurs in the sarcoplasmic reticulum membrane of muscle cells and is involved in Ca^2+^ release during contractions [63], which is possibly affected by PRV-1-induced muscle lesions. The observed high degree of connection to the decisive metabolites could thus confirm the virus-induced damage to muscle cell membranes. The fourth most relevant protein, olfactomedin 4 (OLFM4), was significantly changed in the proteomics study but was not ranged among the top hits [37]. It was, therefore, interesting that the multi-omics approach revealed negative correlations of OLFM4 with the lipids PC(32:3) and PC(32:4) as well as with putatively annotated phosphatidylserine. The increasing plasma levels of OLFM4 in PRV-1-infected salmon can be interpreted as a clear signal for a declining health state since the neutrophilic glycoprotein has crucial tasks in innate immunity and inflammation. In humans, it is considered a biomarker for the severity of viral infections [64]. 

Taken together, the results of the multi-omics approach provided an in-depth analysis of central biological processes and events in salmon during PRV-1 infection and HSMI development. The interrelationship of the data obtained by targeted and untargeted metabolomics and proteomics of virus-challenged fish at critical time points in the disease progression confirmed findings from the individual analyses and allowed us to set them in a wider context. In each data block, five discriminant variables were determined that describe the differences between infected and control salmon, including the lipids PC(32:3) and PC(32:4) and the proteins Gal-3BP, FL-6 and RyR-3. While single metabolites and proteins found significant for the differentiation between infected salmon and healthy controls might be insufficient as indicators for developing HSMI, a combination of several variables might be applicable as relevant biomarkers. 

## Figures and Tables

**Table 1:** Compound classes and major metabolites in salmon plasma (C0) detected by targeted metabolomics.

Compound Class ^1^	Targeted by Kit ^2^	Detected in Salmon Plasma ^3^	Major Metabolites (Top Three) in Compound Class ^4^
Acylcarnitines [AC(X:Y)]	55	35	AC(0:0), AC(2:0), AC(18:1)
Amino acids	21	21	glycine, glutamine, alanine
Biogenic amines	21	10	taurine, trans-4-OH-proline, putrescine
Cholesteryl Esters [CE(X:Y)]	14	11	CE(22:6), CE(20:5), CE(18:2)
Diglycerides [DG(X:Y) and DG-O(X:Y)]	18	17	DG(39:0), DG(36:2), DG-O(34:1)
Triglycerides [TG(X:Y)]	42	30	TG(54:3), TG(52:2), TG(54:4)
Lysophosphatidylcholines [LPC(X:Y)]	24	17	LPC(22:6), LPC(16:0), LPC(18:1)
Phosphatidylcholines [PC(X:Y) and PC-O(X:Y)]	172	95	PC(34:1), PC(34:2), PC(36:4)
Ceramides [Cer(X:Y)]	9	4	Cer(42:2), Cer(42:1), Cer(34:1)
Sphingomyelins [SM(X:Y)]	31	22	SM(42:2), SM(38:2), SM(40:2)
Sum hexoses [including glucose]	1	1	H1

^1^ Compound classes as categorized in the AbsoluteIDQ^®^ p400 HR kit for targeted metabolomics. The possible combinations in the side chain lengths and saturation of fatty acids are indicated as (X:Y); e.g., TG(54:3) indicates a triglyceride with a total 54 carbon atoms in the side chains and three double bonds. ^2^ Number of different metabolites in a compound class targeted by the metabolomics kit. ^3^ Number of metabolites in a compound class confirmed in salmon plasma after data curation by application of quality assurance parameters. ^4^ Major metabolites with the three highest concentrations measured in salmon plasma (based on median values in C0 samples; Table S2b).

**Table 2:** OPLS-DA model characteristics for the pairwise comparison of treatment groups.

Models	R^2^X	R^2^Y	Q^2^	Δ%R^2^Y−Q^2^	CV-ANOVA	Permutation
C2 vs. P2	0.348	0.930	0.555	40%	0.047	valid
C5 vs. P5	0.722	0.606	0.522	14%	0.017	valid
C8 vs. P8	0.634	0.986	0.954	3.2%	0.0000049	valid

Total explained variance—R^2^X; goodness of fit—R^2^Y; predictive ability—Q^2^; difference R^2^Y−Q^2^ relative to R^2^Y, Δ%R^2^Y−Q^2^; CV-ANOVA, *p*-value < 0.05.

**Table 3:** OPLS-DA model characteristics for the pairwise comparison of treatment groups.

Models	R^2^X	R^2^Y	Q^2^	Δ%R^2^Y−Q^2^	CV-ANOVA	Permutation
C2 vs. P2	0.233	0.701	0.440	37%	0.0413	valid
C5 vs. P5	0.227	0.809	0.604	25%	0.0061	valid
C8 vs. P8	0.342	0.897	0.838	6.6%	0.00001	valid

Total explained variance—R^2^X; goodness of fit—R^2^Y; predictive ability—Q^2^; difference R^2^Y−Q^2^ relative to R^2^Y, Δ%R^2^Y−Q^2^; CV-ANOVA, *p*-value < 0.05.

**Table 4:** List of discriminant variables for each data block according to the optimized multiblock sPLS-DA model for components 1 and 2.

Targeted Metabolomics Block ^1^	Untargeted Metabolomics Block ^2^	Proteomics Block
	*Component 1*	
LPC (17:0)	*m*/*z* 578.4171 RT 3.7 P (LPC(22:1))	Ryanodine receptor 3-like isoform X2
PC (32:3)	*m*/*z* 542.3234 RT 3.9 P (PC(20:5))	Fucolectin-6-like isoform X2
PC (32:4)	*m*/*z* 209.0237 RT 11.4 N	Olfactomedin 4-like
PC (40:2)	*m*/*z* 247.1702 RT 3.4 N (FA(16:4))	Galectin-3-binding protein precursor
Serine	*m*/*z* 602.3096 RT 3.9 N (phosphatidylserine)	H-2 class I histocompatibility antigen-Q10 alpha chain-like
	*Component 2*	
Alanine	*m*/*z* 212.1387 RT 9.8 P (amino acid and derivatives)	Histone H3-like partial
AC (6:0)	*m*/*z* 149.0454 RT 12.6 N (D-xylose)	ATP dependent 6-phospho-fructokinase-muscle type like
AC (12:0-DC)	*m*/*z* 168.1492 RT 9.8 P	Glycine-rich RNA binding protein-like isoform X1
ADMA	*m*/*z* 203.1499 RT 22.2 P (ADMA)	Histone H3-3
SDMA	*m*/*z* 166.0144 RT 16.4 N	Barrier to autointegration factor

^1^ AC—acyl carnitine; ADMA—asymmetric dimethylarginine; DC—diglyceride; FA—fatty acid; LPC—lysophosphatidylcholine; PC—phosphocholine; SDMA—symmetric dimethylarginine. ^2^ Name in parenthesis corresponds to the tentative annotation obtained using either CSI:FingerID or CANOPUS; *m*/*z*—mass-to-ratio; N—negative ionization mode; P—positive ionization mode; RT—retention time.

## Data Availability

The original contributions presented in the study are included in the article/Supplementary Materials; further inquiries can be directed to the corresponding author/s. The raw data supporting the conclusions of this article will be made available by the authors on request.

## Supplementary Materials

The following supporting information can be downloaded at: https://www.mdpi.com/article/10.3390/metabo14070375/s1, Figure S1: Volcano blot of targeted metabolomics results (a) at W5, and (b) at W8; Figure S2: Quantitative enrichment analysis of targeted metabolomics results (a) at W5, and (b) at W8; Figure S3: Loading variables for DIABLO multi-omics analysis of (a) component 1, and (b) component 2; Figure S4: Relevance network for component 2 by multi-omics analysis; Table S1: Processing of the untargeted metabolomics data; Table S2a: Targeted metabolomics dataset; Table S2b: Curated targeted metabolomics dataset showing median concentrations and metabolites with significant differences between treatment groups; Table S3: Most-relevant metabolites identified by univariate analysis of the targeted metabolomics data; Table S4: Untargeted metabolomics dataset.
