# Supplementary material for: Metabolomics and Multi-Omics Determination of Potential Plasma Biomarkers in PRV-1-Infected Atlantic Salmon
Source: Metabolites. 2024 Jul 2;14(7):375. doi: 10.3390/metabo14070375 (PMC11279234; doi:10.3390/metabo14070375)
Supplement: Supplementary file 1 [file metabolites-14-00375-s001.zip › PRV-1 metabolomics 2024 Supplementary Figures.pdf]

metabolites

MDPI

Article

Metabolomics and Multi-Omics Determination of Potential Plasma Biomarkers in PRV-1 infected Atlantic Salmon

Lada Ivanova[1*], Oscar D. Rangel-Huerta[1], Haitham Tartor[1], Maria Krudtaa Dahle[1], Silvio Uhlig[1], Christiane Kruse Fæste[1]

[1] Norwegian Veterinary Institute, PO Box 64, 1431 Ås, Norway; lada.ivanova@vetinst.no; oscar.daniel.rangel.huerta@vetinst.no; haitham.tator@vetinst.no; maria.dahle@vetinst.no; silvio.uhlig@vetinst.no; christiane.faste@vetinst.no

* Correspondence: lada.ivanova@vetinst.no

Supplementary Figure S1a

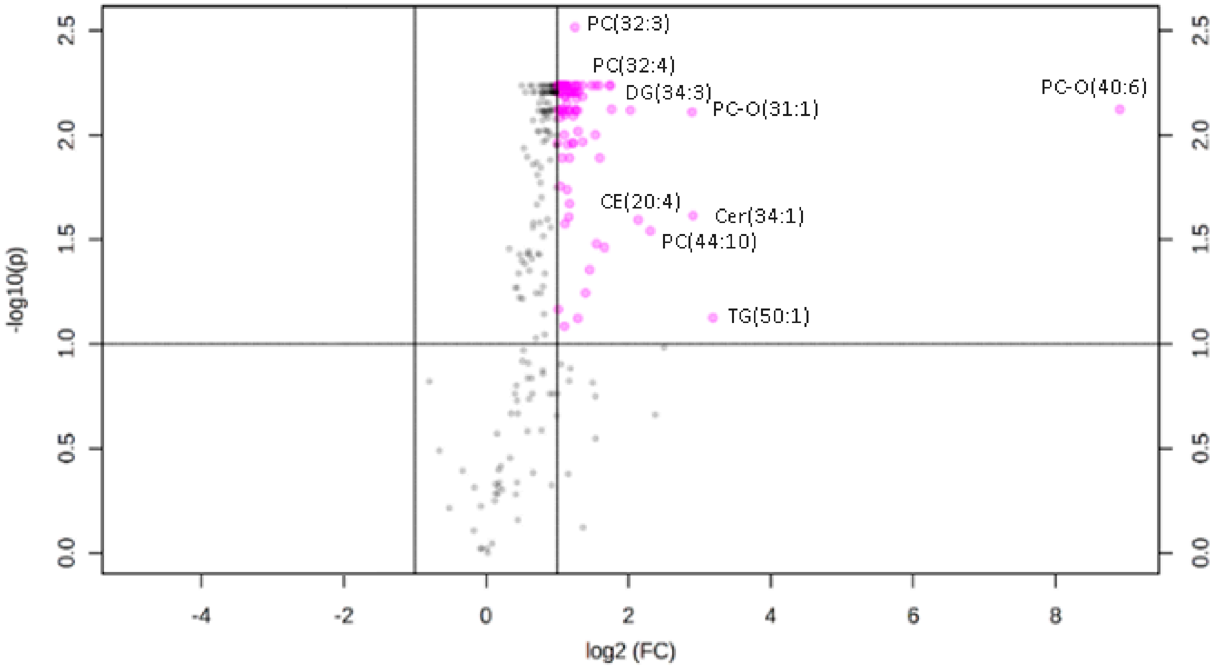

Figure S1a.

Volcano blot showing the distribution of n=88 metabolites with significant differences (-log10(p)>1) between controls (C5) and PRV-1 infected salmon (P5) in week 5 (**Table S2b**). Cut-off at fold change (FC) >2 demonstrating the concentration differences. The blot is based on n=8 replicates per treatment group. Metabolites with notable differences are indicated.

Supplementary Figure S1b

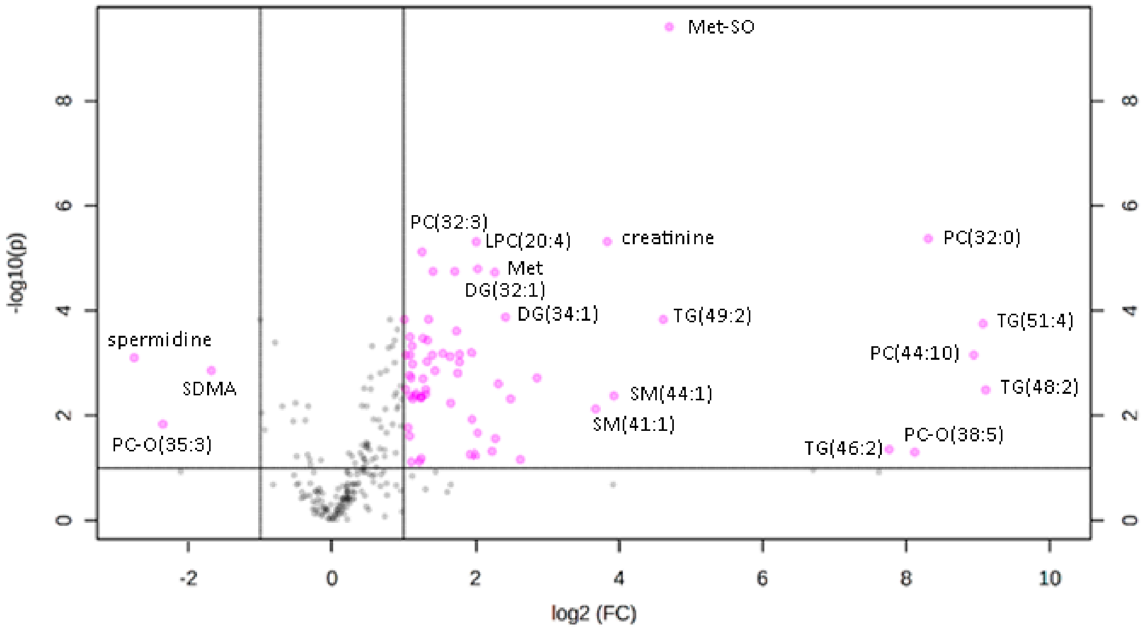

Figure S1b.

Volcano blot showing the distribution of n=70 metabolites with significant differences (-log10(p)>1) between controls (C8) and PRV-1 infected salmon (P8) in week 8 (**Table S2b**). Cut-off at fold change (FC) >2 demonstrating the concentration differences. The blot is based on n=8 replicates per treatment group. Metabolites with notable differences are indicated.

Supplementary Figure S2a

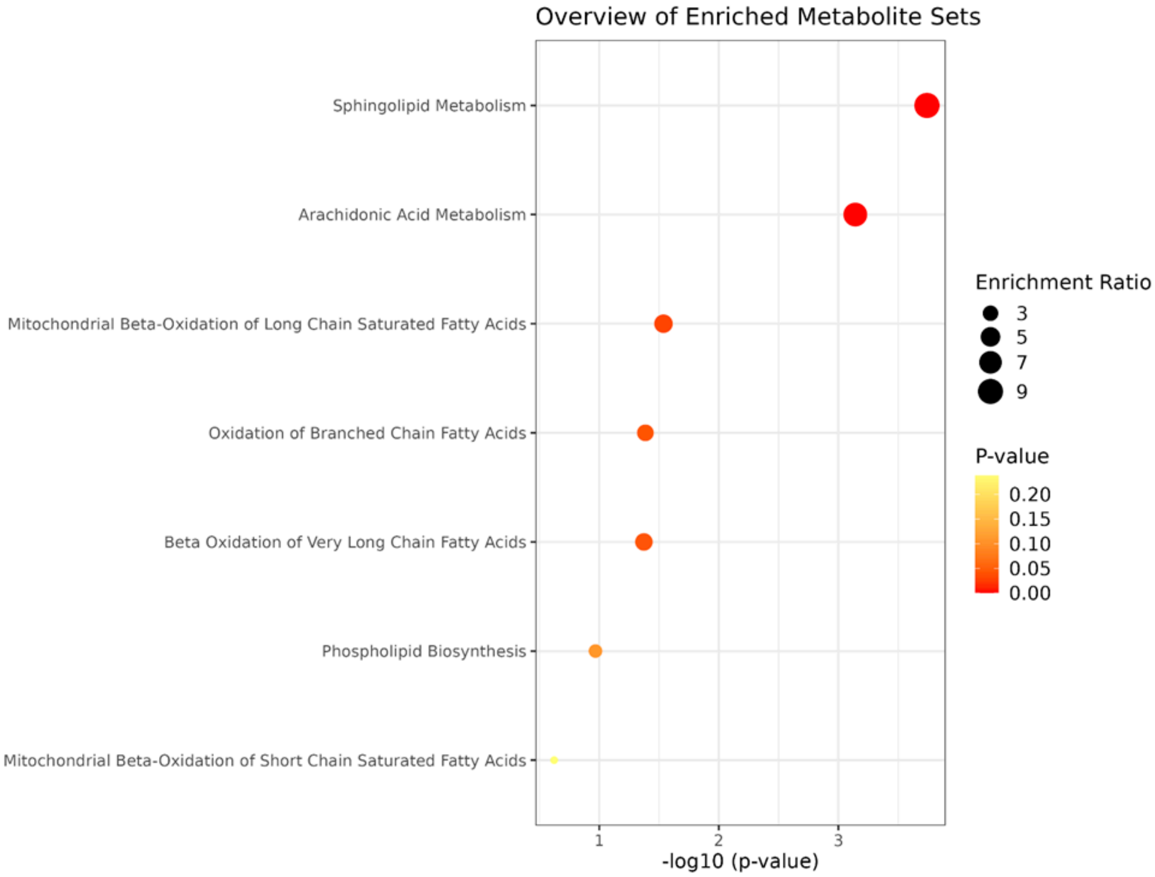

Figure S2a.

Quantitative Enrichment Analysis (QEA) based on metabolite concentrations measured at W5 in C5 and P5 samples showing lipid pathways containing discriminant metabolites for the differentiation between control and PRV-1 infected salmon.

Supplementary Figure S2b

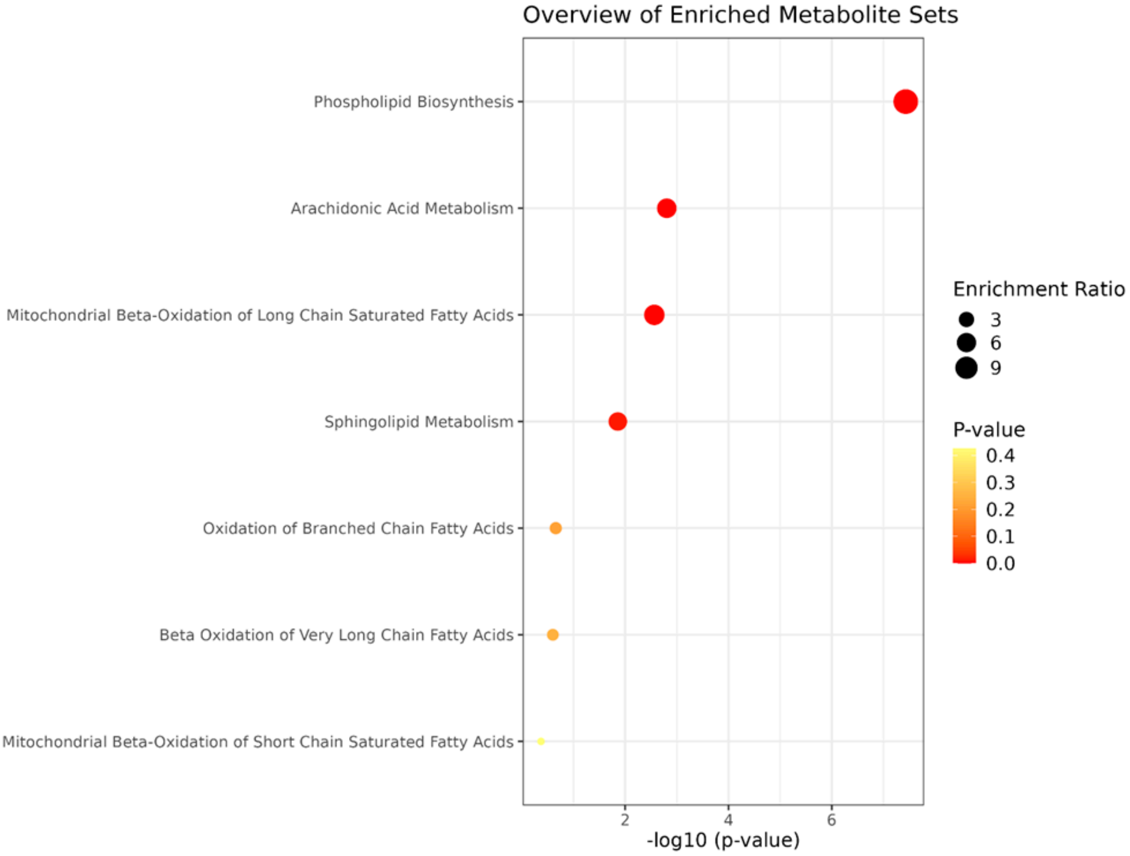

Figure S2b.
Quantitative Enrichment Analysis (QEA) based on metabolite concentrations measured at W8 in C8 and P8 samples showing lipid pathways containing discriminant metabolites for the differentiation between control and PRV-1 infected salmon.

Supplementary Figure S3a

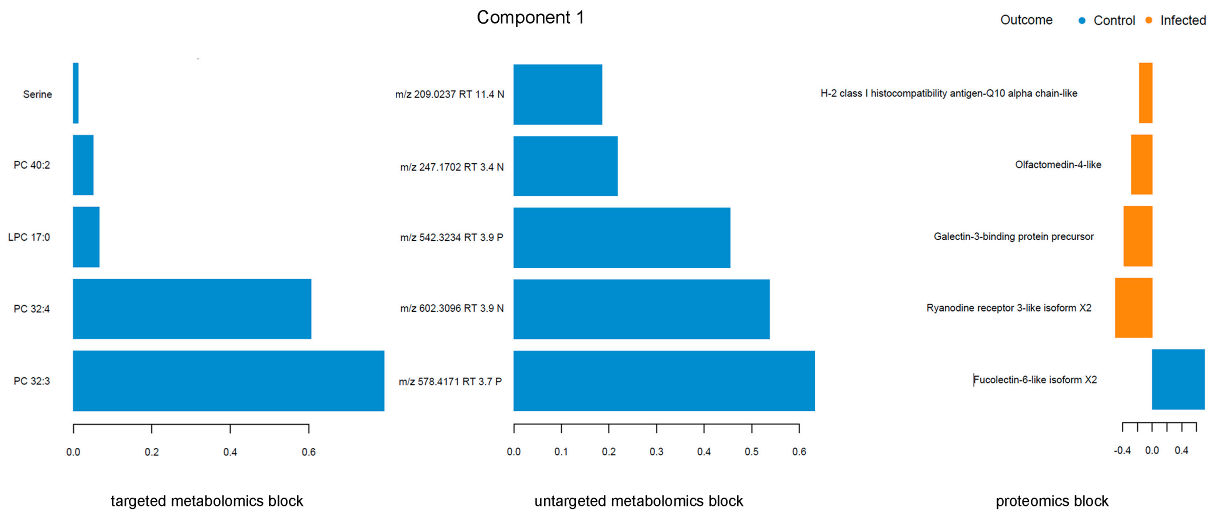

Figure S3a.
Loading variables of component 1 identified as discriminant for the separation between control and PRV-1 infected salmon plasma profiles by the multi-block DIABLO model.

Supplementary Figure S3b

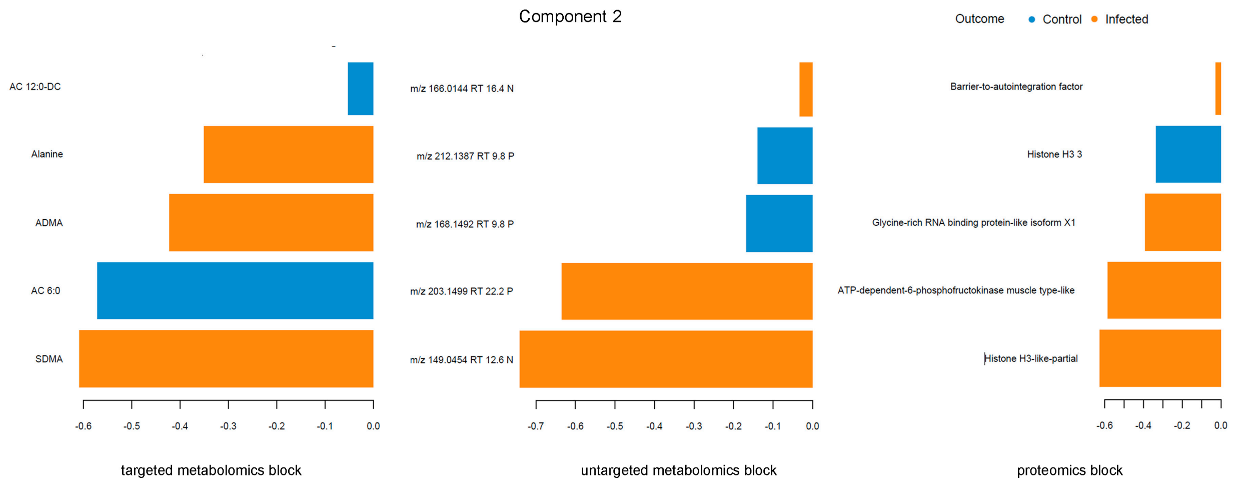

Figure S3b.
Loading variables of component 2 identified as relevant for the separation between salmon plasma profiles within the two treatment groups by the multi-block DIABLO model.

The colour code refers to the treatment group, in which the respective variable has a higher median value. Variables marked in blue had a higher median in the control group, and variables marked in orange had a higher median in the PRV-1 infected group. The most important variables (according to the absolute values of their loading coefficients; x-axis) are ordered from bottom to top.

Supplementary Figure S4

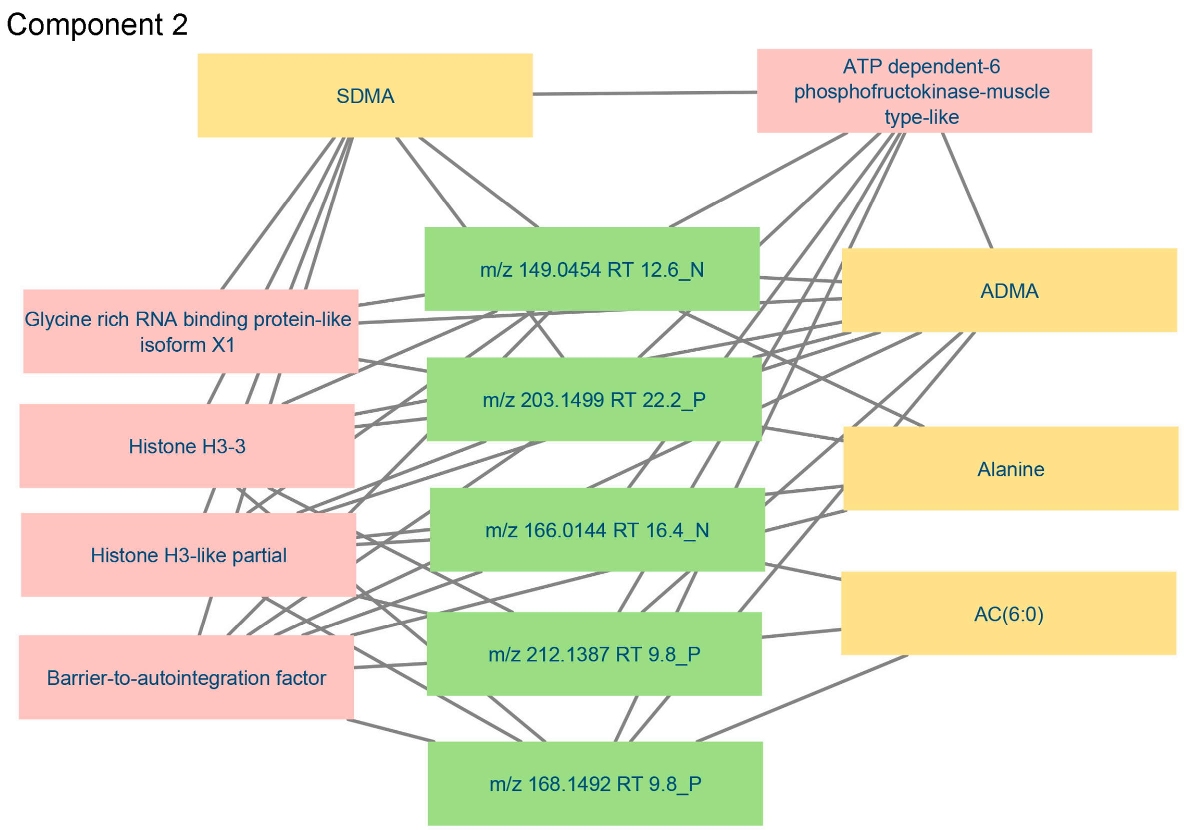

Figure S4.

Relevance network visualising relevant connections (cut-off >0.75) between significant variables in component 2 of the proteomics, targeted and untargeted metabolomics data blocks. The colours of the nodes represent the different data blocks: yellow, targeted metabolomics; green, untargeted metabolomics; pink, proteomics. Solid lines indicate that plasma level changes were in the same direction, and dashed lines indicate changes in the opposite direction, when comparing variable levels in the plasma of control and PRV-1 infected salmon.

Disclaimer/Publisher's Note: The statements, opinions and data contained in all publications are solely those of the individual author(s) and contributor(s) and not of MDPI and/or the editor(s). MDPI and/or the editor(s) disclaim responsibility for any injury to people or property resulting from any ideas, methods, instructions or products referred to in the content.
