# Supplementary material for: Metabolomics and Multi-Omics Determination of Potential Plasma Biomarkers in PRV-1-Infected Atlantic Salmon
Source: Metabolites. 2024 Jul 2;14(7):375. doi: 10.3390/metabo14070375 (PMC11279234; doi:10.3390/metabo14070375)
Supplement: Supplementary file 1 [file metabolites-14-00375-s001.zip › PRV-1 metabolomics 2024 Supplementary Tables.pdf]

Supplementary

Supplementary Table S1.

Processing of the untargeted metabolomics data.

Compound Discoverer 3.3 workflow		
**Select spectra**		
1. General Settings:		
Precursor Selection:	Use MS(n - 1) Precursor	
Provide Profile Spectra	Automatic	
2. Spectrum Properties Filter:		
Lower RT Limit [min]	0.2	
Upper RT Limit [min]	30	
First Scan	0	
Last Scan	0	
Ignore Specified Scans	not Specified	
Lowest Charge State	0	
Highest Charge State	0	
Min. Precursor Mass [Da]	58	
Max. Precursor Mass [Da]	870	
Total Intensity Threshold	0	
Minimum Peak Count	1	
3. Scan Event Filters:		
Mass Analyzer	not Specified	
MS Order	Any	
Activation Type	not Specified	
Min. Collision Energy	0	
Max. Collision Energy	1000	
Scan Type	is Full	
Polarity Mode	Any	
4. Peak Filters:		
S/N Threshold [FT-only]	1.5	
5. Replacements for Unrecognized Properties:		
Unrecognized Charge Replacements	1	
Unrecognized Mass Analyzer Replacements	ITMS	
Unrecognized MS Order Replacements	MS2	
Unrecognized Activation Type Replacements	CID	
Unrecognized Polarity Replacements	+	
Unrecognized MS Resolution@200 Replacements	60000	
Unrecognized MSn Resolution@200 Replacements	30000	
**Retention time alignment**		
1. General Settings:		
Alignment Model	Adaptive curve	
Alignment Fallback	Use Linear Model	
Maximum Shift [min]	0.2	
Shift Reference File	True	
Mass Tolerance [ppm]	5	
Remove Outlier	True	
**Detect Compounds**		
1. General Settings:		
Mass Tolerance [ppm]	5	
Intensity Tolerance [%]	30	
S/N Threshold	5	
Min. Peak Intensity	500000	
Ions	Positive	Negative
	$[2M+H]^{+1}$	$[M-2H]^{-2}$
	$[2M+K]^{+1}$	$[M-2H+K]^{-1}$
	$[2M+Na]^{+1}$	$[M-H]^{-1}$
	$[M+2H]^{+2}$	$[2M-H]^{-1}$
	$[M+H]^{+1}$	$[M+FA-H]^{-1}$
	$[M+H-H_2O]^{+1}$	$[M+H+HAc]^{-1}$
	$[M+H-NH_3]^{+1}$	$[M+H+H_2O]^{-1}$
	$[M+K]^{+1}$	
	$[M+Na]^{+1}$	
	$[M+NH_4]^{+1}$	
Base Ions	$[M+H]^{+1}$	$[M-H]^{-1}$
Min. Element Counts	C, H	
Max. Element Counts	C90H190Br3Cl4K2N10Na2O15P3S5	
2. Peak Detection:		
Filter Peaks	True	
Max. Peak Width [min]	0.5	
Remove Singlets	True	
Min. # Scans per Peak	5	
Min. # Isotopes	1	
**Group Compounds**		
1. Compound Consolidation:		
Mass Tolerance [ppm]	5	

RT Tolerance [min]	0.2	
2. Fragment Data Selection:		
Preferred Ions	$[M+H]^{+1}$	$[M-H]^{-1}$
**Fill Gaps**		
1. General Settings:		
Mass Tolerance [ppm]	5	
S/N Threshold	1.5	
Use Real Peak Detection	True	
**Peak Rating Filtering**		
Peak Rating Filtering treshold	4 (Scale of 10)	
Min number of files	6	
**Normalize Areas**		
1. QC-based Area Correction:		
Regression Model	SERRF QC correction	
Min. QC Coverage [%]	50	
Max. QC Area RSD [%] before correction	30	
Max. QC Area RSD [%] after correction	25	
Max. # Files Between QC Files	13	
2. Area Normalization:		
Normalization Type	Total sum	
Exclude Blanks	True	
3. Scaling Factor:		
Study Factor Name	not Specified	
**Mark Background Compounds**		
1. General Settings:		
Max. Sample/Blank	5	
Max. Blank/Sample	0	
Hide Background	True	

Supplementary Table S2a.

Targeted metabolomics dataset showing concentrations (μM) of quantified metabolites. Only metabolites that appeared in at least 80% of the samples in one treatment group of the study were considered. Missing values for some metabolites (NF: not found) were imputed during subsequent processing, which included also data normalisation (s. Materials and Methods).

Metabolite	C0_1	C0_2	C0_3	C0_4	C0_5	C0_6	C0_7	C0_8	C2_1	C2_2	C2_3	C2_4	C2_5	C2_6	C2_7	C2_8	P2_1	P2_2	P2_3	P2_4	P2_5	P2_6	P2_7	P2_8	C5_1	C5_2	C5_3	C5_4	C5_5	C5_7	C5_8	P5_1	P5_2	P5_3	P5_4	P5_5	P5_6	P5_7	C8_2	C8_3	C8_4	C8_5	C8_6	C8_7	C8_8	P8_1	P8_3	P8_4	P8_5	P8_6	P8_7	P8_8
AC(0:0)	2.450	3.909	4.784	4.171	2.742	4.891	3.578	4.881	3.141	3.442	5.698	4.473	3.024	4.969	4.794	5.329	5.620	3.131	4.074	3.355	3.559	4.784	4.560	2.985	3.919	4.026	3.899	6.690	8.168	4.094	5.163	1.585	5.970	3.734	3.014	3.102	2.548	3.082	7.098	4.094	6.097	6.690	7.030	5.406	5.922	11.86	8.761	8.605	7.322	6.272	9.276	9.383
AC(2:0)	0.815	0.993	2.164	1.574	1.162	3.103	1.261	1.592	1.010	1.395	1.949	1.815	0.957	2.781	2.307	2.316	2.495	0.740	1.699	0.939	1.136	1.735	1.306	1.619	2.101	1.270	1.958	2.781	3.666	1.484	1.511	0.378	2.647	1.556	1.037	0.790	0.825	1.458	2.227	1.243	1.976	4.256	2.388	2.110	2.772	5.920	2.763	2.629	2.048	2.003	2.692	3.675
AC(3:0)	0.088	0.181	0.137	0.149	0.090	0.165	0.130	0.270	0.083	0.195	0.307	0.172	0.148	0.234	0.231	0.346	0.085	0.070	0.155	0.193	0.078	0.104	0.124	0.170	0.141	0.188	0.143	0.271	0.308	0.079	0.171	0.054	0.255	0.146	0.078	0.064	0.154	0.050	0.278	0.219	0.323	0.407	0.248	0.225	0.324	0.254	0.396	0.324	0.462	0.287	0.318	0.314
AC(3:0-OH)	0.110	0.130	0.095	0.086	0.142	0.161	0.167	0.130	0.102	0.090	0.092	0.093	0.111	0.103	0.094	0.157	0.102	0.098	0.098	0.102	0.112	0.119	0.100	0.146	0.077	0.095	0.145	0.120	0.151	0.136	0.117	0.048	0.110	0.074	0.070	0.069	0.083	0.051	0.092	0.136	0.154	0.151	0.090	0.077	0.123	0.054	0.099	0.082	0.107	0.077	0.066	0.105
AC(4:0)	0.103	0.081	0.063	0.081	0.108	0.085	0.077	0.138	0.070	0.079	0.072	0.068	0.068	0.073	0.055	0.079	0.065	0.058	0.072	0.068	0.068	0.090	0.079	0.084	0.055	0.063	0.075	0.095	0.090	0.073	0.071	0.044	0.059	0.060	0.049	0.095	0.048	0.038	0.067	0.101	0.084	0.117	0.065	0.062	0.071	0.041	0.076	0.090	0.092	0.086	0.049	0.053
AC(5:0-OH)	0.022	0.022	0.020	0.019	0.024	0.034	0.020	0.024	0.016	0.020	0.023	0.023	0.017	0.020	0.020	0.024	0.025	0.019	0.017	0.024	0.025	0.017	0.023	0.016	0.020	0.018	0.023	0.029	0.026	0.020	0.017	0.014	0.021	0.018	0.016	0.018	0.017	0.017	0.017	0.029	0.034	0.041	0.021	0.023	0.020	0.018	0.017	0.017	0.023	0.021	0.016	0.026
AC(6:0)	0.025	0.027	0.029	0.021	0.025	0.040	0.021	0.025	0.019	0.022	0.020	0.023	0.015	0.021	0.021	0.034	0.044	0.013	0.025	0.017	0.018	0.021	0.022	0.019	0.017	0.025	0.021	0.026	0.042	0.027	0.021	0.009	0.027	0.016	0.011	0.020	0.016	0.015	0.024	0.023	0.031	0.042	0.029	0.031	0.023	0.029	0.025	0.022	0.021	0.022	0.018	0.050
AC(6:0-DC)	0.053	0.054	0.049	0.034	0.057	0.095	0.064	0.043	0.061	0.070	0.043	0.031	0.035	0.056	0.034	0.045	0.028	0.027	0.031	0.065	0.035	0.029	0.032	0.052	0.031	0.039	0.043	0.047	0.037	0.037	0.038	0.019	0.041	0.033	0.022	0.022	0.031	0.020	0.036	0.039	0.050	0.057	0.034	0.028	0.031	0.022	0.032	0.024	0.035	0.026	0.027	0.030
AC(6:0-OH)	0.023	0.017	0.024	0.012	0.024	0.034	0.036	0.022	0.019	0.023	0.016	0.021	0.027	0.013	0.015	0.025	0.022	0.009	0.017	0.057	0.024	0.024	0.028	0.027	0.013	0.013	0.012	0.025	0.037	0.019	0.020	0.011	0.026	0.012	0.015	0.028	0.019	0.009	0.013	0.012	0.033	0.024	0.015	0.021	0.016	0.014	0.023	0.020	0.012	0.020	0.018	0.022
AC(7:0)	0.025	0.028	0.027	0.024	0.028	0.030	0.028	0.028	0.024	0.023	0.021	0.022	0.019	0.021	0.019	0.025	0.018	0.020	0.019	0.020	0.020	0.019	0.019	0.023	0.016	0.023	0.026	0.027	0.026	0.023	0.021	0.011	0.018	0.018	0.014	0.015	0.015	0.013	0.019	0.025	0.024	0.026	0.018	0.017	0.019	0.012	0.018	0.015	0.017	0.014	0.015	0.016
AC(8:0)	0.051	0.045	0.041	0.055	0.058	0.073	0.046	0.046	0.048	0.037	0.040	0.042	0.041	0.037	0.041	0.050	0.039	0.048	0.046	0.046	0.044	0.050	0.047	0.052	0.041	0.039	0.048	0.046	0.055	0.051	0.058	0.029	0.042	0.049	0.046	0.047	0.049	0.041	0.037	0.051	0.051	0.051	0.044	0.040	0.044	0.038	0.038	0.048	0.038	0.050	0.048	0.040
AC(8:1)	0.014	0.012	0.011	0.013	0.011	0.014	0.011	0.011	0.011	0.011	0.011	0.010	0.012	0.010	0.009	0.014	0.012	0.011	0.011	0.012	0.012	0.014	0.011	0.010	0.012	0.012	0.011	0.011	0.016	0.011	0.011	0.008	0.010	0.010	0.008	0.009	0.008	0.007	0.011	0.011	0.014	0.014	0.009	0.009	0.011	0.011	0.012	0.012	0.031	0.010	0.018	0.011
AC(8:1-OH)	0.271	0.228	0.160	0.130	0.176	0.188	0.130	0.124	0.153	0.167	0.145	0.191	0.176	0.234	0.224	0.305	0.121	0.089	0.268	0.205	0.232	0.144	0.303	0.121	0.180	0.204	0.111	0.090	0.264	0.146	0.084	0.123	0.203	0.146	0.046	0.192	0.069	0.074	0.189	0.206	0.118	0.250	0.143	0.166	0.212	0.160	0.184	0.159	0.141	0.199	0.086	0.167
AC(9:0)	0.022	0.018	0.022	0.019	0.020	0.023	0.017	0.024	0.018	0.018	0.011	0.018	0.015	0.013	0.013	0.020	0.015	0.011	0.012	0.015	0.014	0.016	0.014	0.017	0.013	0.012	0.019	0.016	0.015	0.017	0.017	0.005	0.014	0.010	0.011	0.011	0.010	0.008	0.014	0.017	0.022	0.017	0.011	0.013	0.014	0.009	0.012	0.012	0.012	0.011	0.014	0.012
AC(10:0)	0.023	0.024	0.017	0.020	0.017	0.023	0.016	0.022	0.016	0.022	0.022	0.025	0.021	0.014	0.020	0.033	0.019	0.013	0.017	0.023	0.018	0.018	0.017	0.014	0.016	0.018	0.015	0.022	0.029	0.017	0.018	0.014	0.021	0.017	0.011	0.016	0.010	0.008	0.010	0.011	0.014	0.019	0.013	0.016	0.016	0.029	0.025	0.019	0.018	0.012	0.010	0.023
AC(10:1)	0.005	0.007	0.006	0.005	0.007	0.007	0.006	0.006	0.004	0.005	0.005	0.005	0.004	0.005	0.004	0.006	0.004	0.004	0.004	0.005	0.005	0.005	0.005	0.005	0.003	0.005	0.006	0.006	0.006	0.006	0.005	0.003	0.004	0.003	0.003	0.003	0.003	0.003	0.003	0.005	0.007	0.005	0.004	0.004	0.004	0.003	0.004	0.004	0.004	0.003	0.003	0.004
AC(12:0)	0.058	0.046	0.057	0.073	0.075	0.089	0.029	0.085	0.055	0.074	0.053	0.088	0.043	0.063	0.056	0.101	0.047	0.035	0.060	0.046	0.049	0.056	0.040	0.056	0.068	0.057	0.055	0.092	0.076	0.056	0.055	0.018	0.069	0.051	0.042	0.040	0.030	0.021	0.023	0.025	0.021	0.072	0.045	0.047	0.051	0.088	0.065	0.046	0.047	0.026	0.019	0.050
AC(12:0-DC)	0.018	0.026	0.041	0.028	0.028	0.037	0.025	0.030	0.018	0.023	0.025	0.033	0.025	0.026	0.024	0.037	0.027	0.018	0.032	0.033	0.029	0.031	0.027	0.029	0.018	0.027	0.030	0.028	0.039	0.021	0.027	0.019	0.044	0.024	0.021	0.031	0.016	0.013	0.042	0.043	0.050	0.060	0.053	0.043	0.067	0.027	0.038	0.031	0.047	0.049	0.034	0.069
AC(12:1)	0.010	0.015	0.016	0.019	0.028	0.044	0.019	0.038	0.021	0.032	0.028	0.042	0.014	0.026	0.032	0.042	0.026	0.014	0.021	0.023	0.019	0.021	0.007	0.020	0.030	0.023	0.024	0.038	0.027	0.023	0.024	0.010	0.027	0.012	0.006	0.019	0.009	0.002	0.012	0.004	0.009	0.035	0.016	0.012	0.015	0.038	0.023	0.012	0.009	0.012	0.008	0.015
AC(13:0)	0.021	0.021	0.024	0.023	0.021	0.035	0.021	0.032	0.024	0.022	0.024	0.028	0.027	0.026	0.024	0.032	0.027	0.021	0.021	0.024	0.023	0.027	0.020	0.026	0.023	0.021	0.020	0.030	0.031	0.024	0.022	0.009	0.024	0.024	0.017	0.022	0.015	0.015	0.022	0.023	0.021	0.020	0.022	0.024	0.026	0.028	0.022	0.026	0.020	0.018	0.019	0.026
AC(14:0)	0.085	0.103	0.111	0.099	0.123	0.143	0.097	0.143	0.096	0.095	0.087	0.109	0.070	0.084	0.085	0.131	0.070	0.074	0.080	0.076	0.076	0.085	0.084	0.117	0.081	0.101	0.098	0.137	0.118	0.084	0.089	0.029	0.077	0.062	0.048	0.058	0.041	0.035	0.059	0.085	0.086	0.129	0.087	0.099	0.102	0.095	0.095	0.074	0.076	0.057	0.044	0.080
AC(14:0-OH)	0.008	0.013	0.011	0.008	0.016	0.020	0.009	0.014	0.009	0.011	0.010	0.011	0.008	0.010	0.010	0.025	0.008	0.009	0.008	0.008	0.011	0.009	0.007	0.012	0.010	0.009	0.013	0.021	0.016	0.009	0.012	0.003	0.011	0.009	0.006	0.008	0.004	0.004	0.005	0.008	0.008	0.015	0.007	0.006	0.008	0.016	0.011	0.007	0.010	0.007	0.004	0.012
AC(14:1)	0.071	0.104	0.134	0.140	0.115	0.200	0.077	0.109	0.120	0.134	0.141	0.174	0.118	0.113	0.114	0.160	0.103	0.078	0.120	0.111	0.117	0.101	0.077	0.094	0.122	0.139	0.157	0.213	0.162	0.108	0.101	0.053	0.125	0.097	0.094	0.101	0.056	0.046	0.057	0.052	0.059	0.126	0.071	0.064	0.082	0.169	0.127	0.122	0.093	0.054	0.073	0.098
AC(14:2)	0.005	0.006	0.007	0.006	0.009	0.014	0.008	0.007	0.006	0.010	0.012	0.012	0.005	0.009	0.007	0.014	0.008	0.005	0.010	0.006	0.006	0.008	0.006	0.011	0.013	0.006	0.008	0.013	0.012	0.006	0.006	0.003	0.005	0.006	0.003	0.004	0.003	0.003	0.005	0.006	0.008	0.008	0.006	0.006	0.006	0.011	0.004	0.003	0.004	0.004	0.004	0.003
AC(14:2-OH)	0.008	0.015	0.015	0.013	0.013	0.017	0.013	0.016	0.008	0.014	0.012	0.015	0.017	0.010	0.010	0.015	0.013	0.013	0.010	0.024	0.013	0.010	0.010	0.010	0.007	0.006	0.015	0.013	0.015	0.008	0.013	0.006	0.015	0.011	0.013	0.013	0.010	0.009	0.012	0.011	0.013	0.013	0.008	0.012	0.012	0.009	0.011	0.010	0.013	0.009	0.004	0.014
AC(15:0)	0.029	0.021	0.028	0.028	0.034	0.025	0.028	0.030	0.022	0.019	0.016	0.019	0.018	0.014	0.017	0.020	0.013	0.017	0.017	0.020	0.021	0.016	0.014	0.021	0.012	0.021	0.024	0.031	0.023	0.017	0.020	0.006	0.019	0.012	0.010	0.010	0.010	0.003	0.009	0.018	0.020	0.023	0.017	0.018	0.016	0.032	0.027	0.013	0.014	0.012	0.014	0.018
AC(16:0)	0.415	0.346	0.433	0.275	0.410	0.673	0.339	0.462	0.364	0.342	0.198	0.323	0.254	0.257	0.313	0.247	0.122	0.304	0.169	0.226	0.317	0.231	0.299	0.428	0.402	0.343	0.399	0.543	0.375	0.375	0.356	0.139	0.271	0.275	0.208	0.274	0.129	0.119	0.076	0.137	0.121	0.396	0.192	0.211	0.199	0.381	0.364	0.235	0.270	0.218	0.121	0.282
AC(16:0-OH)	0.037	0.026	0.039	0.023	0.048	0.064	0.024	0.038	0.027	0.037	0.012	0.030	0.018	0.029	0.035	0.029	0.005	0.026	0.013	0.020	0.022	0.017	0.033	0.052	0.025	0.037	0.043	0.049	0.047	0.024	0.028	0.006	0.024	0.031	0.011	0.017	0.008	0.008	0.003	0.003	0.004	0.010	0.004	0.004	0.004	0.008	0.009	0.004	0.006	0.003	0.003	0.010
AC(16:1-OH)	0.029	0.025	0.025	0.106	0.034	0.057	0.029	0.024	0.027	0.031	0.023	0.047	0.029	0.043	0.032	0.050	0.019	0.028	0.030	0.021	0.034	0.026	0.033	0.045	0.031	0.035	0.046	0.161	0.049	0.024	0.024	0.034	0.045	0.033	0.025	0.029	0.020	0.013	0.015	0.015	0.016	0.024	0.019	0.019	0.021	0.031	0.024	0.016	0.020	0.016	0.014	0.029
AC(16:2)	0.004	0.005	0.006	0.005	0.006	0.008	0.005	0.006	0.005	0.005	0.005	0.006	0.004	0.005	0.004	0.006	0.004	0.004	0.004	0.004	0.004	0.004	0.004	0.006	0.004	0.005	0.005	0.006	0.006	0.005	0.005	0.002	0.004	0.003	0.003	0.003	0.002	0.002	0.004	0.005	0.005	0.008	0.005	0.006	0.006	0.006	0.004	0.003	0.004	0.003	0.003	0.004
AC(17:0)	0.004	0.004	0.007	0.004	0.023	0.033	0.009	0.026	0.007	0.005	0.006	0.007	0.003	0.003	0.004	0.033	0.006	0.005	0.003	0.006	0.005	0.006	0.006	0.030	0.008	0.024	0.015	0.013	0.013	0.008	0.012	0.002	0.008	0.005	0.004	0.005	0.004	0.004	0.007	0.008	0.011	0.016	0.010	0.009	0.010	0.016	0.021	0.007	0.016	0.006	0.006	0.012
AC(18:0)	0.081	0.080	0.090	0.063	0.104	0.107	0.052	0.079	0.046	0.048	0.040	0.063	0.064	0.030	0.046	0.060	0.031	0.032	0.035	0.058	0.032	0.035	0.050	0.072	0.046	0.055	0.047	0.078	0.082	0.074	0.079	0.037	0.059	0.067	0.037	0.057	0.030	0.029	0.023	0.040	0.044	0.117	0.036	0.053	0.051	0.117	0.128	0.119	0.103	0.083	0.067	0.101
AC(18:1)	0.961	0.890	1.139	0.646	1.038	1.743	0.916	1.028	0.859	0.762	0.494	1.006	0.574	0.587	0.792	0.697	0.338	0.743	0.597	0.538	0.826	0.605	0.820	1.250	0.953	0.774	0.917	1.179	1.119	0.950	0.855	0.354	0.697	0.818	0.530	0.712	0.312	0.352	0.411	0.609	0.455	1.270	0.954	1.098	1.058	1.633	1.310	0.897	0.850	0.795	0.530	1.018
AC(18:1-OH)	0.224	0.072	0.225	0.066	0.238	0.489	0.048	0.118	0.058	0.091	0.015	0.246	0.064	0.212	0.127	0.182	0.021	0.092	0.031	0.072	0.095	0.118	0.155	0.286	0.161	0.104	0.195	0.334	0.328	0.054	0.124	0.025	0.110	0.112	0.049	0.075	0.031	0.027	0.019	0.031	0.042	0.067	0.031	0.012	0.037	0.118	0.078	0.039	0.028	0.046	0.021	0.094
AC(18:2)	0.188	0.179	0.245	0.131	0.205	0.350	0.204	0.209	0.191	0.170	0.121	0.228	0.117	0.157	0.180	0.156	0.040	0.174	0.147	0.125	0.194	0.133	0.173	0.292	0.231	0.166	0.187	0.261	0.250	0.199	0.155	0.071	0.140	0.167	0.114	0.137	0.060	0.068	0.118	0.160	0.127	0.297	0.288	0.293	0.309	0.375	0.257	0.166	0.178	0.175	0.079	0.260
Ala	598.1	949.5	1671	686.7	652.4	798.4	764.2	937.4	757.2	508.5	780.3	778.3	536.7	882.0	681.6	686.7	803.5	961.6	1346	679.6	782.3	1122	1002	1095	610.2	760.2	876.0	866.9	799.4	603.1	1082	379.6	1027	1028	678.6	767.2	492.4	729.0	685.7	501.4	594.0	519.5	941.4	795.4	1055	696.7	952.5	1023	960.5	1041	950.5	1528
Arg	337.9	293.6	394.4	283.3	385.9	251.3	324.7	329.4	282.4	272.9	368.9	316.2	319.1	390.6	271.1	259.8	308.7	308.7	376.5	199.5	336.9	392.5	360.5	354.8	247.5	364.2	312.5	362.4	572.2	292.7	261.6	104.5	412.2	437.6	313.4	338.8	213.6	304.0	277.6	230.6	256.0	253.2	244.7	275.8	304.0	256.9	388.7	302.1	308.7	275.8	296.5	449.9
Asn	23.24	23.55	14.72	11.46	23.97	60.99	17.66	26.18	15.04	8.769	14.83	6.729	6.971	13.56	6.572	9.726	16.51	10.24	15.46	5.783	7.907	8.191	8.906	8.664	4.574	13.67	12.72	13.88	20.50	10.83	12.51	2.050	48.79	15.88	18.82	6.099	3.028	4.648	8.874	8.717	11.88	18.51	10.33	9.400	11.25	7.308	12.72	6.288	8.338	12.51	4.511	12.41
Asp	128.7	96.40	99.84	103.1	111.4	148.2	79.39	114.9	67.90	54.23	47.80	52.16	38.60	79.51	79.28	62.96	116.0	49.06	58.60	37.91	34.47	45.50	58.14	45.73	34.93	82.84	65.49	114.4	152.8	89.04	168.9	20.68	103.5	52.85	50.44	37.11	34.01	30.22	104.9	114.2	137.9	135.6	110.8	107.1	171.2	165.4	212.6	235.5	220.6	198.8	235.5	258.5
Cit	22.70	17.33	19.63	18.09	19.74	21.27	13.93	17.65	17.11	14.04	14.47	14.58	12.61	16.23	14.15	14.47	14.15	13.49	19.30	10.52	12.50	14.58	12.50	14.58	7.709	15.90	12.17	21.38	22.70	18.86	16.56	6.174	32.02	17.44	17.87	12.50	9.968	9.924	21.16	26.10	31.47	40.90	27.85	27.19	30.48	14.80	12.50	14.26	13.49	12.39	9.430	19.74
Gln	854.2	887.5	1089	608.8	813.8	1008	772.4	665.4	859.2	665.4	859.2	883.5	825.9	853.2	692.6	664.4	609.8	728.0	1050	602.8	744.1	963.2	1064	810.8	477.6	1051	743.1	903.7	1162	673.4	1028	260.5	889.5	966.3	679.5	691.6	461.4	542.2	817.8	686.6	894.6	620.9	628.0	598.7	840.0	381.7	671.4	481.6	540.2	384.7	377.6	771.4
Glu	170.7	220.6	169.5	287.9	145.1	225.2	156.7	371.5	154.4	160.2	548.0	261.2	96.8	203.2	247.3	318.1	255.4	123.1	160.2	175.3	94.6	101.0	121.9	138.1	126.5	251.9	160.2	474.8	363.4	189.2	310.0	50.5	254.2	92.8	101.9	86.3	133.5	89.7	221.7	298.4	307.6	238.0	236.8	164.9	240.3	163.7	250.8	279.8	573.5	157.9	265.9	267.0
Gly	986.8	932.1	1328	1141	1019	1222	1095	1192	890.6	657.6	1209	965.6	807.5	1412	935.2	904.8	1183	865.3	1231	774.1	802.4	1103	1166	1137	717.3	1162	898.7	1329	1726	1245	1323	400.2	1117	1023	756.9	690.0	538.0	773.1	1103	1078	1088	1250	1263	1186	1380	1249	1417	1362	1302	1229	1235	1488

His	61.04	61.44	53.66	59.17	64.68	65.77	48.83	65.47	43.12	48.64	63.99	48.83	49.92	49.33	56.61	55.33	54.64	46.67	49.82	31.60	52.87	70.00	49.52	51.89	30.52	73.84	68.92	74.82	83.19	56.41	78.37	18.51	74.63	62.22	49.42	45.19	42.83	33.57	80.83	70.30	119.1	101.4	69.51	54.54	85.95	11.42	34.85	30.23	45.19	41.65	23.83	47.95
Ile	216.0	213.0	252.0	165.0	223.0	245.0	170.0	180.0	180.0	192.0	187.0	185.0	164.0	221.0	197.0	197.0	190.0	200.0	239.0	212.0	210.0	209.0	213.0	253.0	121.0	189.0	182.0	206.0	272.0	143.0	182.0	77.00	233.0	242.0	168.0	195.0	138.0	126.0	370.0	434.0	460.0	692.0	348.0	323.0	495.0	172.0	260.0	235.0	244.0	235.0	173.0	383.0
Lys	614.8	623.7	833.5	468.6	658.3	1033	430.7	670.6	584.7	521.1	663.9	578.0	554.5	728.6	613.7	511.0	555.6	689.5	850.2	421.8	610.3	907.1	752.0	527.8	303.5	706.3	648.3	827.9	889.3	631.5	1033	147.3	763.2	819.0	552.3	725.2	320.2	369.3	353.7	387.2	446.3	483.1	386.1	353.7	509.9	534.4	717.4	458.6	590.2	593.6	518.8	738.6
Met	118.5	121.7	116.4	99.42	129.2	127.1	95.15	104.5	83.83	101.1	78.70	80.20	80.63	87.25	95.36	93.33	87.14	97.50	95.36	85.11	88.74	100.2	80.31	104.8	60.12	99.10	117.5	115.3	120.7	99.21	98.4	29.90	104.9	73.36	79.56	66.74	67.06	46.99	302.2	267.0	383.4	563.9	270.2	277.7	388.7	63.11	97.93	75.29	98.78	80.73	62.37	121.7
Orn	48.32	41.43	99.60	32.17	63.39	359.7	44.52	42.50	31.93	30.27	37.04	55.20	34.43	42.74	81.91	30.87	33.72	38.35	63.16	33.12	31.93	40.13	58.05	51.05	42.14	39.06	28.85	52.95	84.17	48.32	224.4	12.58	65.18	69.92	44.04	54.02	19.47	37.28	44.52	18.88	35.50	38.82	38.82	29.44	94.85	37.99	52.24	43.69	30.27	53.78	35.38	80.49
Phe	145.4	160.2	167.2	143.4	159.3	161.2	134.5	164.2	136.5	136.5	136.5	124.6	124.6	150.3	129.6	141.4	134.5	114.7	148.4	125.6	126.6	135.5	126.6	163.2	101.9	130.6	125.6	146.4	185.0	121.7	140.5	49.0	133.5	120.7	110.8	94.1	100.9	83.6	261.1	219.6	285.9	298.7	261.1	211.7	278.9	95.8	158.3	133.5	159.3	123.6	104.8	215.6
Pro	131.8	408.0	521.9	81.52	131.8	364.1	203.6	102.2	213.4	56.9	145.3	174.0	60.00	452.9	81.43	62.15	116.6	495.9	582.9	392.8	275.3	488.8	517.5	243.0	80.35	184.7	183.8	210.7	280.7	123.8	177.6	48.61	297.7	177.6	65.47	216.1	39.28	73.09	265.5	253.8	551.5	644.8	305.8	379.3	682.5	48.6	107.6	121.1	110.3	98.6	105.8	208.1
Ser	288.6	240.9	344.4	300.6	269.7	295.6	296.6	305.5	223.9	192.1	275.7	250.8	180.1	320.5	228.9	186.1	315.5	229.9	294.6	199.1	264.7	271.7	245.8	266.7	177.2	278.7	220.9	282.7	389.1	342.4	267.7	77.03	245.8	278.7	198.1	164.2	127.4	193.1	387.2	304.5	335.4	239.9	305.5	358.3	348.3	180.1	203.0	151.3	143.3	160.2	141.3	229.9
Thr	450.6	447.5	433.3	340.5	538.3	715.6	291.6	399.6	449.6	351.7	427.1	446.5	423.1	383.3	478.1	459.8	241.6	524.0	410.8	281.4	448.5	486.3	408.8	338.4	148.8	536.2	582.1	737.0	490.3	508.7	814.5	117.2	556.6	428.2	435.3	505.6	234.5	157.0	457.7	438.3	828.8	978.6	555.6	419.0	849.2	291.6	471.0	387.4	568.8	401.7	407.8	437.3
Trp	40.42	35.60	42.23	37.11	46.86	43.54	35.10	44.04	30.67	35.60	32.28	32.08	27.55	37.61	31.07	35.40	28.96	28.86	33.99	27.35	32.58	35.30	34.69	38.92	27.35	35.20	32.18	40.73	54.50	33.89	37.01	10.86	40.73	29.16	29.97	26.35	27.05	19.01	43.84	43.34	53.10	55.11	38.11	26.45	43.14	18.91	37.81	31.07	40.63	29.97	17.80	60.84
Tyr	104.9	87.01	140.2	93.55	114.2	91.57	68.63	97.28	73.20	98.53	103.5	80.26	74.34	131.9	93.44	90.33	84.62	68.11	77.87	85.66	78.18	77.97	69.25	95.10	86.69	75.17	82.23	89.60	164.0	93.44	77.45	32.08	89.29	62.92	78.39	59.60	69.04	56.79	234.6	182.7	177.5	159.9	263.7	305.2	254.4	97.39	130.8	108.0	116.3	109.0	80.26	205.6
Val	495.8	503.6	570.0	359.2	502.6	571.9	381.6	386.5	394.3	436.3	380.6	417.7	364.1	477.3	475.3	411.9	389.4	481.2	507.5	436.3	473.4	478.2	450.9	517.3	263.5	436.3	435.3	488.0	601.2	367.0	490.9	182.5	510.5	531.9	409.0	460.7	317.2	262.5	756.4	799.4	989.7	1402.5	726.2	641.2	931.1	392.4	587.6	529.0	537.8	554.4	397.2	888.2
xLeu	539.3	527.1	748.7	391.6	543.3	590.9	388.5	472.5	482.6	472.5	462.4	483.6	387.5	524.1	493.7	479.6	500.8	585.8	616.2	518.0	512.0	528.1	479.6	646.5	249.9	447.2	475.5	546.4	679.9	350.1	482.6	198.3	590.9	612.1	402.7	481.6	326.8	277.2	982.4	1297	1316	2664	1104	864.0	1538	432.0	621.2	573.7	626.3	692.0	449.2	1080
ADMA	1.926	1.482	2.540	1.440	1.831	1.821	1.471	2.011	1.365	1.313	1.609	1.514	1.344	1.662	1.482	1.334	1.450	1.143	1.895	1.122	1.567	1.672	1.620	1.514	0.918	1.651	1.365	1.916	2.318	1.503	1.831	0.499	2.339	1.588	1.344	1.482	0.802	0.956	0.822	1.054	1.207	1.238	1.323	1.323	1.471	2.159	2.329	2.001	2.403	2.329	2.371	3.271
Creatinine	19.18	9.666	8.729	16.67	16.35	6.255	3.999	4.250	2.300	1.860	4.686	5.525	0.379	2.340	5.666	7.900	14.28	4.827	6.168	11.22	5.307	5.013	5.775	0.934	4.392	2.440	4.326	3.062	6.070	4.915	NF	3.487	16.24	4.152	6.767	5.034	4.228	4.751	75.95	51.32	65.06	72.68	56.77	45.44	64.73	2.040	4.392	3.847	1.470	5.449	3.727	9.317
Met-SO	3.835	2.519	2.448	2.872	2.943	2.957	1.627	2.434	1.670	1.726	1.315	2.363	1.528	1.797	1.825	1.542	2.165	2.137	1.939	1.211	1.882	2.052	1.755	2.179	1.556	2.533	3.184	2.957	3.877	2.335	2.052	0.569	4.061	1.712	2.108	1.457	1.556	1.373	34.81	24.48	34.81	47.54	27.73	28.02	37.21	0.974	1.377	1.142	1.179	1.190	1.408	1.755
Putrescine	18.50	21.36	27.46	20.12	23.46	29.85	27.46	38.33	25.65	27.65	55.59	24.22	18.02	29.85	23.36	27.37	36.81	16.31	23.27	24.13	19.45	19.17	15.45	22.89	21.07	33.18	27.56	44.72	40.24	29.47	45.68	7.724	34.33	19.55	16.31	18.98	17.16	14.78	31.94	39.86	48.82	45.96	42.05	32.90	42.05	43.86	46.53	52.54	60.27	42.15	63.32	40.15
Sarcosine	4.212	3.241	5.530	4.116	4.914	4.357	3.453	4.049	2.674	3.366	3.789	3.251	2.866	4.433	4.308	4.135	3.799	3.808	4.347	3.270	3.578	5.136	3.674	4.251	2.000	3.645	3.856	6.078	4.645	4.559	5.366	0.681	5.155	3.106	3.597	3.077	2.568	1.818	3.443	4.260	7.867	10.29	4.703	4.212	6.126	3.741	6.040	4.972	6.088	5.270	4.943	5.299
SDMA	1.085	1.643	2.356	1.498	1.017	1.219	1.085	1.188	0.891	0.776	1.011	0.995	0.756	1.044	1.178	1.178	1.808	0.873	1.612	1.664	1.323	1.364	1.602	0.879	0.747	1.354	0.834	1.405	3.441	1.030	1.240	0.457	2.573	1.385	1.374	1.199	0.972	1.519	0.519	0.730	0.635	0.794	0.808	0.491	0.512	1.002	1.457	1.612	1.705	2.738	1.364	4.474
Spermidine	0.988	1.045	1.122	1.343	1.122	1.276	1.976	2.877	1.381	1.870	3.865	1.113	0.921	1.832	1.256	1.669	3.740	0.541	0.988	1.400	1.237	0.633	0.349	1.074	1.621	2.091	2.014	2.666	1.678	2.158	2.724	0.458	2.100	0.551	0.929	0.766	0.795	0.630	2.062	2.541	3.462	1.765	1.554	2.033	1.745	3.328	4.249	4.095	5.284	3.117	7.068	1.928
Spermine	0.578	0.359	0.290	0.299	NF	0.313	0.386	0.476	NF	0.389	0.521	NF	NF	0.346	0.312	NF	2.501	NF	NF	NF	NF	0.302	NF	NF	0.657	NF	0.392	0.429	0.265	0.441	NF	NF	0.567	NF	0.274	NF	NF	NF	0.687	NF	NF	NF	NF	NF	NF	0.784	0.569	1.085	0.787	0.443	1.847	0.418
t4-OH-Pro	412.4	444.3	567.3	440.5	371.0	398.3	532.6	462.1	441.5	314.7	493.1	388.9	356.0	511.0	387.0	309.0	425.5	440.5	460.3	421.8	451.8	453.7	435.8	597.4	279.0	498.8	315.6	478.1	665.0	463.1	463.1	268.6	528.8	592.7	418.0	392.6	295.9	309.0	290.2	323.1	357.9	356.9	360.7	346.6	370.1	331.6	415.2	497.8	437.7	420.8	441.5	562.6
Taurine	1075	1250	1811	1271	1071	1479	1485	1699	1187	1132	1819	1344	1070	1709	1778	1345	1460	1188	1480	1230	900.2	1113	1204	1634	1083	1378	1443	2060	2654	1728	2446	729.1	1726	1204	904.1	734.0	712.6	775.8	1731	2063	2312	2555	2471	2151	2210	2635	2609	3106	2693	2522	3127	2773
CE(16:0)	263.3	245.3	259.8	257.5	277.0	267.7	246.5	281.0	288.4	264.1	219.1	223.4	231.3	299.0	253.6	268.8	393.3	464.9	385.0	333.0	297.4	303.7	251.6	460.2	406.6	283.3	278.6	252.0	264.1	278.2	242.6	230.5	575.2	225.8	232.4	326.3	248.1	272.3	313.8	226.6	247.3	235.6	301.3	256.3	293.9	230.1	267.7	211.3	233.2	192.1	226.6	228.5
CE(16:1)	639.0	723.0	720.0	598.0	686.0	803.0	710.0	768.0	669.0	500.0	653.0	526.0	461.0	483.0	505.0	527.0	533.0	554.0	589.0	567.0	534.0	564.0	542.0	659.0	341.0	527.0	625.0	648.0	664.0	486.0	715.0	187.0	502.0	352.0	341.0	340.0	335.0	225.0	413.0	475.0	568.0	158.0	567.0	534.0	459.0	407.0	616.0	525.0	600.0	437.0	569.0	480.0
CE(17:1)	57.14	55.66	65.06	45.76	59.12	70.75	60.11	55.91	51.21	42.55	57.14	44.03	39.33	39.08	43.54	46.26	42.79	46.01	48.98	45.76	50.96	47.25	42.79	51.95	31.17	47.25	58.38	58.38	65.55	38.59	66.29	17.69	49.97	29.19	34.88	33.15	33.64	23.25	32.41	40.32	36.12	34.14	48.48	41.81	43.78	34.38	55.16	48.98	55.91	40.32	47.00	48.24
CE(17:2)	29.03	25.97	38.21	20.84	26.56	29.44	26.20	26.02	25.70	19.45	27.14	16.74	18.00	19.17	18.54	20.21	15.53	21.79	24.71	22.73	23.50	22.28	21.83	19.72	14.54	21.83	25.30	21.74	36.50	19.17	32.18	7.88	22.06	15.39	15.53	16.61	15.75	11.21	12.65	12.87	17.60	8.73	21.65	15.17	17.82	19.17	29.53	23.90	29.66	21.38	23.09	25.21
CE(18:2)	981.9	1094	1070	965.6	1187	1285	1142	1293	1016	875.3	1058	826.8	771.7	689.9	766.9	907.4	753.5	782.6	835.9	850.5	841.4	857.1	795.3	918.9	533.1	869.8	1137	1172	1190	855.3	1224	259.9	792.9	477.3	537.9	539.1	560.9	358.0	625.7	765.1	955.9	759.6	878.9	792.3	760.2	580.9	879.5	867.4	939.5	660.3	832.9	741.4
CE(18:3)	409.4	472.4	515.9	388.0	468.5	517.2	505.5	522.4	458.1	358.8	452.3	364.7	314.7	319.9	332.9	384.8	356.9	334.8	406.9	371.8	380.2	397.8	365.3	412.7	257.6	373.1	460.1	486.7	578.2	342.6	509.4	112.3	344.6	220.6	228.4	227.8	238.1	172.0	288.8	315.4	378.3	263.4	426.3	364.0	338.1	280.3	402.3	378.3	387.4	309.5	362.7	362.7
CE(19:2)	19.60	20.64	26.77	16.60	21.99	31.11	26.06	21.99	19.73	16.20	17.85	16.30	15.42	12.39	20.20	22.19	12.56	18.05	12.93	14.88	16.94	22.90	16.94	18.59	9.26	17.95	25.72	23.57	29.60	18.59	28.96	5.219	16.36	12.02	10.67	12.66	10.88	8.957	12.86	12.05	20.37	15.76	17.17	13.50	16.67	9.80	19.06	19.50	18.35	12.56	13.13	19.56
CE(19:3)	8.943	10.43	14.67	6.881	8.670	12.49	12.43	9.761	8.882	6.427	9.034	7.245	6.366	6.184	8.731	12.70	6.942	8.336	7.670	6.487	9.034	9.549	9.367	8.882	4.941	9.579	11.13	10.40	14.10	9.852	13.37	3.395	7.366	6.245	5.153	7.185	5.851	4.911	6.396	4.365	7.851	4.881	8.215	6.730	8.761	6.033	10.13	9.064	9.246	6.366	7.215	10.06
CE(20:4)	530.0	711.6	30.44	623.5	32.28	144.1	768.4	775.7	482.3	108.2	1140	1010	964.6	469.5	929.8	634.5	335.6	363.1	190.7	1073	544.7	581.3	97.38	726.2	387.0	353.9	1465	137.2	209.1	1007	1933	220.1	75.92	372.3	73.91	79.96	366.8	58.13	442.0	1366	893.1	1427	121.8	110.8	770.2	17.57	26.59	192.6	240.2	185.2	596.0	25.67
CE(20:5)	3896	4118	4781	3477	4828	5129	4478	4084	3700	3604	3875	3500	3345	2877	3114	3551	3031	3292	3864	3248	3771	4022	3764	3556	2524	3731	4940	5186	6133	3030	4660	1154	3532	2383	2521	2635	2379	1824	2640	2803	3746	2817	4212	3545	3484	2717	3844	3485	4039	3250	3193	3783
CE(22:6)	19322	19560	19505	16817	21782	21762	20373	20948	16678	15942	17721	14322	14418	12816	13676	16301	13210	14937	14766	14551	15364	15904	14152	16322	9618	15359	19258	19836	20428	14889	20682	4544	13754	9697	10678	9484	10313	6844	10735	13532	14868	12742	15728	14218	13552	10492	16462	14736	18016	12194	14986	14758
DG(32:1)	4.211	6.780	3.374	4.785	5.942	5.479	4.211	6.328	4.884	3.396	3.484	5.016	2.977	4.244	3.021	7.717	5.810	3.153	4.013	3.583	2.381	4.884	4.917	9.613	1.709	4.586	3.032	5.413	3.848	4.763	2.899	0.972	2.117	3.318	1.819	1.010	1.240	1.330	6.802	11.13	10.02	16.32	6.041	5.325	10.64	1.731	1.885	1.742	1.797	1.995	1.940	2.635
DG(32:2)	1.878	3.043	1.611	2.741	3.114	2.234	1.646	3.123	2.563	1.273	1.975	2.296	1.602	2.011	1.335	3.017	3.275	1.611	2.474	2.136	1.192	2.785	2.687	3.933	1.379	2.073	1.397	2.447	2.056	2.554	1.477	NF	1.014	0.925	0.800	NF	0.731	1.041	2.545	4.912	4.164	6.709	2.972	2.732	4.511	0.421	1.299	0.643	1.210	NF	1.424	1.984
DG(34:1)	11.96	17.45	10.06	14.71	17.75	18.97	15.39	21.79	11.35	10.29	10.13	14.86	10.82	11.13	8.763	19.13	15.39	8.077	10.29	7.597	6.744	13.26	11.28	20.35	5.334	13.11	10.52	17.53	10.36	16.38	9.982	1.989	8.991	5.776	6.530	3.848	5.242	2.492	16.31	26.59	27.89	56.16	17.91	16.31	22.94	2.995	4.160	5.570	5.037	3.299	4.892	8.458
DG(34:3)	4.157	6.766	3.157	4.717	6.063	4.872	4.872	5.801	4.193	2.359	3.562	5.920	2.871	3.824	2.466	5.194	5.789	2.752	3.669	2.907	2.025	4.836	5.313	11.054	1.382	4.681	2.597	5.682	3.073	5.682	2.299	0.114	2.323	0.129	2.025	0.786	0.192	0.616	7.278	12.03	9.744	19.893	8.326	7.135	11.91	0.857	1.429	1.334	1.703	0.089	2.823	2.299
DG(36:2)	18.08	22.41	5.40	20.70	23.23	26.10	13.25	28.39	18.57	14.40	13.58	17.18	16.44	18.49	14.64	19.80	16.12	13.99	18.08	13.82	4.982	15.54	17.51	28.71	9.162	18.49	4.810	19.55	14.97	17.59	16.93	4.336	13.09	10.06	11.62	6.111	8.589	3.837	15.95	28.30	22.74	42.70	16.52	14.48	21.43	2.634	3.305	10.80	9.980	2.241	6.561	15.95
DG(36:3)	10.81	10.67	5.706	9.021	9.308	8.234	7.876	9.522	7.138	6.215	6.881	7.733	6.888	8.090	5.835	8.663	9.809	4.661	7.876	5.799	3.816	8.090	8.162	15.25	4.031	8.735	4.725	7.804	6.902	9.021	6.966	1.675	5.062	4.196	4.038	3.236	3.186	2.212	7.589	14.10	13.10	18.83	8.878	7.947	11.89	3.329	4.582	4.267	6.558	3.136	5.434	5.549
DG(36:4)	8.502	4.387	2.174	4.608	4.978	3.446	4.355	3.738	3.109	3.057	2.460	2.934	2.921	3.433	2.239	3.271	4.530	1.876	3.115	2.311	1.357	4.108	3.362	6.685	2.155	4.245	1.707	3.953	2.778	3.894	3.239	0.544	1.856	1.564	2.103	1.376	1.142	0.785	3.154	6.244	5.724	9.086	3.232	3.738	5.134	1.363	2.090	2.181	3.141	1.058	2.616	2.220
DG(38:0)	2.292	0.932	9.582	2.055	5.861	0.461	15.21	15.18	0.252	2.213	8.595	4.281	5.532	1.788	1.037	0.488	3.112	0.397	0.453	6.092	1.475	0.522	0.584	0.542	0.317	1.452	4.511	10.11	7.804	5.203	4.577	0.240	1.185	4.808	7.277	0.394	0.489	0.550	10.24	0.488	18.93	0.205	0.463	7.244	12.32	0.371	6.289	0.472	0.554	6.257	12.09	3.919
DG(38:5)	33.98	1.961	3.391	14.431	20.33	5.241	0.309	4.624	3.700	0.149	1.843	3.726	0.138	1.279	3.267	2.991	1.220	1.830	1.738	2.211	2.683	6.822	2.499	1.351	3.096	7.675	2.926	10.10	3.149	5.556	11.02	0.682	1.935	1.909	3.234	2.742	3.221	1.305	1.614	7.543	6.822	21.187	3.680	1.509	5.077	1.614	3.522	3.975	11.55	1.522	0.296	1.850
DG(39:0)	245.5	244.6	251.8	207.3	240.2	239.3	256.2	221.5	189.5	198.4	242.9	185.9	198.4	152.1	159.2	204.6	162.8	190.4	226.9	203.7	221.5	236.6	224.2	165.5	153.9	228.6	254.4	224.2	258.0	174.4	227.7	96.1	231.3	135.2	151.2	141.4	156.6	107.6	169.9	188.6	226.9	162.8	214.4	188.6	182.4	139.7	183.3	215.3	165.5	161.0	166.4	171.7
DG(42:0)	2.296	2.845	2.475	2.660	3.562	5.216	3.250	4.210	2.897	2.834	3.123	2.915	1.989	1.671	2.296	2.591	1.972	2.192	1.845	2.203	2.041	2.429	2.307	2.897	1.157	2.440	2.868	3.140	2.839	2.481	3.007	0.758	1.885	1.290	1.267	1.365	1.365	0.937	1.839	2.446	2.996	3.296	2.134	2.157	2.036	1.544	2.342	2.088	2.683	1.787	2.279	1.839
DG(42:1)	1.175	1.379	1.140	0.559	1.034	1.633	1.705	0.775	1.183	1.100	1.420	1.356	1.016	0.677	1.042	1.270	0.968	1.031	1.031	1.218	0.904	0.939	1.025	1.235	0.634	1.201	1.731	1.402	0.651	0.786	1.506	0.337	1.232	0.328	0.685	0.622	0.648	0.360	0.619	1.106	1.230	0.369	0.317	0.985	0.438	0.579	1.014	1.123	1.209	0.671	1.088	0.579
DG(42:2)	8.063	8.322	9.812	6.606	8.484	9.456	8.938	9.164	7.416	6.153	8.355	6.995	5.861	5.278	6.347	7.448	5.505	6.250	5.991	6.088	6.898	8.711	7.351	6.703	4.436	6.865	8.873	7.772	9.553	7.027	9.812	2.445	6.509	4.696	4.275	4.048	4.469	3.562	5.764	6.671	8.258	6.671	7.578	6.865	7.383	4.436	6.671	6.347	7.027	5.278	5.958	6.768
DG(44:3)	1.281	1.203	1.682	1.294	1.734	2.170	1.486	1.808	1.503	1.194	1.473	1.490	1.333	0.697	0.985	0.967	1.050	0.867	0.819	1.089	0.854	1.473	1.394	1.473	0.597	1.103	1.556	1.743	1.455	0.937	1.756	0.330	0.911	0.628	0.584	0.715	0.654	0.545	0.889	0.972	1.316	1.207	1.503	1.233	1.299	0.872	0.906	0.906	1.347	1.116	1.150	0.981
DG-O(32:2)	2.253	2.314	1.725	1.735	2.791	2.243	2.659	3.582	1.644	1.380	1.502	1.614	1.299	1.543	1.218	2.527	1.360	1.001	1.614	1.167	0.789	1.888	1.644	2.436	0.935	2.101	2.101	2.395	1.350	2.547	1.370	0.555	1.644	0.686	1.003	0.628	1.015	0.405	2.111	4.212	4.506	5.460	1.928	2.202	2.933	0.450	0.914	1.248	0.859	0.657	1.076	1.603
DG-O(34:1)	17.15	22.78	16.07	16.15	19.63	19.55	19.05	23.61	14.75	14.41	16.48	15.91	14.66	16.40	11.10	18.31	14.41	12.01	16.98	13.42	8.62	15.91	15.49	20.05	9.526	20.79	14.17	17.23	12.67	22.20	15.74	6.163	16.65	10.27	10.93	5.91	10.60	4.830	15.99	32.47	28.25	38.35	15.49	13.92	18.56	5.62	9.94	11.10	10.85	6.73	10.35	14.50

DG-O(36:4)	5.658	6.322	5.933	7.374	13.81	14.21	6.214	8.983	4.371	7.508	4.961	8.112	5.584	3.949	5.028	5.933	5.175	4.177	4.063	4.673	4.451	4.404	4.592	5.236	5.557	5.276	10.06	9.586	7.374	6.422	7.843	2.320	5.806	3.533	3.520	2.997	4.626	2.306	4.699	6.972	7.776	12.87	7.106	4.699	6.355	2.434	3.587	3.513	4.518	3.895	3.318	3.486
TG(46:2)	0.045	0.054	0.045	0.048	0.056	0.048	0.045	0.031	0.036	0.048	0.037	0.044	0.039	0.024	0.022	0.025	0.021	0.038	0.042	0.030	0.056	0.041	0.055	0.062	0.036	0.047	0.024	0.043	0.021	0.054	0.036	0.024	0.036	0.024	0.027	0.020	0.022	0.018	0.052	19.50	10.80	0.046	0.049	4.130	0.036	0.022	0.035	0.016	0.026	0.014	0.020	0.026
TG(48:1)	34.75	34.08	27.44	24.16	39.76	42.55	35.43	40.24	28.30	26.86	24.93	23.30	23.49	21.47	19.16	30.52	23.01	18.77	22.62	19.64	17.91	27.44	27.05	29.65	13.19	29.94	24.74	38.41	28.01	33.60	22.24	6.421	25.13	13.29	17.91	9.560	14.92	8.558	29.84	50.35	65.75	74.13	26.57	26.86	41.59	11.74	14.83	13.77	20.02	15.40	18.58	22.05
TG(48:2)	0.060	0.076	0.075	NF	NF	NF	NF	0.120	0.062	0.055	0.063	0.079	0.063	0.038	0.058	0.056	0.103	0.090	28.25	0.161	0.089	0.094	0.089	0.110	0.039	0.087	0.079	0.045	0.074	0.069	0.079	0.059	0.097	0.071	0.071	0.062	0.058	0.047	24.89	53.62	56.12	56.698	0.119	0.121	33.73	0.048	0.059	0.051	0.073	0.051	0.065	0.061
TG(49:2)	0.049	9.020	2.945	0.042	0.067	0.060	0.039	0.058	4.951	0.056	4.405	5.244	2.161	5.454	0.048	2.756	4.279	0.075	5.328	0.064	0.059	0.070	0.067	6.964	0.042	0.055	0.045	11.16	5.286	9.691	5.160	0.041	2.899	0.046	2.941	0.042	0.045	0.035	4.909	7.342	9.733	11.285	5.748	4.321	5.496	0.033	1.372	0.038	0.053	0.039	0.042	0.415
TG(50:1)	0.267	0.204	0.266	0.245	0.319	0.422	0.219	0.470	0.265	0.248	0.190	0.229	0.258	0.223	0.193	0.315	0.357	0.322	0.357	5.375	0.282	0.317	0.362	0.385	0.209	0.321	0.284	0.307	9.708	0.421	2.521	0.177	0.326	0.219	0.241	0.194	0.216	0.139	18.96	2.625	1.333	182.3	0.566	0.430	0.460	0.173	0.186	0.167	8.531	4.208	0.176	0.215
TG(50:2)	122.3	194.7	0.523	89.70	238.5	178.4	146.8	220.2	143.7	145.8	105.0	109.1	145.8	133.5	96.13	165.1	134.6	90.31	156.0	95.82	67.69	132.5	93.07	165.1	38.33	145.8	128.4	179.4	145.8	166.2	127.4	0.42	111.1	0.62	83.49	48.22	49.74	0.28	117.2	230.4	247.7	289.5	149.8	115.2	152.9	0.343	82.67	52.50	81.55	47.81	75.94	137.6
TG(50:3)	69.86	87.15	73.01	60.71	83.59	71.99	74.94	99.45	68.94	50.74	58.37	58.27	46.67	59.89	42.00	73.01	61.01	48.00	70.27	44.44	32.64	64.78	55.83	91.52	28.88	73.42	53.59	86.23	63.05	84.91	57.56	10.27	54.50	27.56	32.44	21.35	26.44	11.19	67.72	137.3	151.5	133.2	77.69	66.30	90.81	22.07	41.90	27.25	46.88	25.12	35.79	49.12
TG(50:4)	13.64	6.362	6.870	9.123	11.90	10.79	12.38	11.42	8.488	9.044	8.806	9.202	7.536	9.123	9.361	16.66	8.885	14.52	10.95	12.53	12.85	13.57	10.39	9.202	12.53	9.916	10.55	7.862	15.15	38.55	8.568	12.53	13.41	8.33	10.63	11.90	9.520	10.87	20.31	69.18	74.57	82.50	15.55	11.98	37.68	11.90	13.33	11.19	13.57	10.79	11.98	7.520
TG(51:2)	0.231	0.143	0.220	0.219	18.44	0.268	0.183	0.237	0.194	0.154	0.151	0.193	54.23	45.34	17.34	65.21	35.24	0.211	42.15	31.51	0.187	51.05	0.203	0.234	0.110	61.59	29.20	87.38	49.62	62.35	33.37	0.111	0.188	0.119	26.68	8.716	13.17	8.299	35.35	69.16	95.18	91.88	31.73	37.10	56.43	0.094	43.03	0.114	30.74	0.093	0.095	0.126
TG(51:3)	34.12	38.16	35.61	36.25	42.95	39.44	37.85	43.59	29.13	27.96	25.09	25.41	27.43	29.66	23.17	34.76	27.00	16.80	24.77	19.99	15.31	29.02	25.94	33.91	15.63	35.19	24.88	44.65	30.72	42.95	32.00	5.815	26.36	15.20	18.60	9.196	15.84	5.613	24.88	44.65	50.18	55.07	29.55	21.05	30.40	9.876	17.33	10.17	21.47	9.196	16.69	23.60
TG(51:4)	0.065	NF	0.074	0.058	0.052	0.065	0.025	0.226	0.050	0.048	0.057	0.041	0.045	0.047	0.050	0.095	12.84	0.074	17.70	0.059	0.040	0.053	0.063	0.049	0.033	0.049	0.038	0.053	0.068	0.154	0.055	0.031	12.67	0.037	0.037	0.032	0.033	0.026	11.80	0.090	20.22	39.050	21.17	16.66	11.72	0.031	0.036	0.025	0.034	0.030	0.036	0.033
TG(52:2)	371.7	408.5	370.7	295.2	508.9	380.7	320.0	399.6	297.2	227.6	280.3	261.4	230.6	272.3	196.8	327.0	252.5	187.8	260.4	165.0	144.1	247.5	239.5	343.9	130.2	308.1	280.3	376.7	283.3	335.9	255.4	69.47	267.4	125.2	167.0	94.42	131.2	18.69	208.7	408.5	488.0	605.3	285.3	228.6	307.1	85.08	207.7	120.3	241.5	105.4	198.8	254.4
TG(52:3)	223.7	283.1	216.6	235.8	286.2	312.4	266.0	310.4	222.7	184.4	201.5	195.5	184.4	226.7	150.1	242.8	204.5	147.1	221.7	144.1	105.8	211.6	199.5	296.2	106.8	263.0	205.6	288.2	221.7	292.2	230.7	41.41	186.4	90.79	119.9	72.85	109.8	43.73	195.5	356.7	382.9	428.2	231.8	193.5	252.9	85.25	159.2	107.8	169.3	93.61	142.1	200.5
TG(52:4)	127.6	168.1	138.8	135.7	176.2	147.9	154.0	164.1	116.5	93.39	114.5	113.4	97.13	123.6	82.14	127.6	118.5	86.19	123.6	95.92	71.00	124.6	117.5	164.1	60.67	132.7	114.5	147.9	134.7	158.0	108.4	21.57	110.4	62.29	63.30	44.77	51.25	25.63	114.5	205.6	218.8	223.8	146.9	119.5	167.1	51.45	83.16	72.12	96.32	60.57	79.00	106.4
TG(52:5)	73.13	95.58	74.89	69.79	81.66	83.52	93.03	84.99	64.11	51.17	54.50	56.17	51.86	61.85	46.07	73.03	64.60	46.66	68.72	49.31	34.90	62.64	68.03	96.46	28.53	73.52	63.52	78.81	78.13	89.60	68.42	12.25	55.97	31.37	37.35	22.64	28.92	7.695	66.46	126.5	134.3	144.1	84.11	67.34	88.22	23.72	37.94	33.62	49.80	29.11	40.19	54.89
TG(52:6)	38.81	45.59	36.38	36.64	64.52	58.61	50.36	56.27	41.85	27.79	36.64	28.65	34.21	36.56	25.70	41.51	34.21	22.32	36.30	23.10	15.54	36.38	30.39	64.86	18.50	54.36	27.96	50.36	47.93	54.96	33.69	6.573	36.21	17.97	22.66	17.80	23.36	6.087	36.90	76.93	77.11	91.17	46.28	33.78	47.50	11.46	15.11	19.97	21.79	13.29	16.85	32.39
TG(52:7)	16.54	13.78	15.31	15.62	22.05	18.17	21.03	15.62	13.99	12.25	13.07	13.78	15.31	12.45	11.23	13.37	15.72	13.17	11.84	12.45	11.54	16.23	18.17	17.46	11.23	17.15	16.13	16.95	16.95	12.96	16.64	6.013	16.84	7.758	8.228	8.248	8.932	6.891	13.88	28.07	25.52	27.66	15.82	16.84	20.42	8.269	9.790	9.269	11.23	8.024	10.31	11.43
TG(53:3)	0.035	14.11	9.547	13.50	11.03	15.60	0.145	14.97	11.51	0.170	0.115	13.29	10.09	12.38	6.959	12.99	11.40	7.833	11.17	NF	5.088	4.476	0.047	15.142	6.732	14.34	0.174	15.53	12.82	20.46	13.78	0.146	12.08	5.403	7.501	3.270	5.630	3.585	10.88	0.168	NF	22.03	NF	10.96	NF	3.882	8.813	0.141	8.078	4.196	6.365	10.09
TG(53:4)	26.03	28.89	28.27	26.34	34.22	36.07	29.27	29.97	24.41	19.54	21.01	21.70	21.24	23.33	18.69	7.261	20.08	16.45	21.86	15.06	10.27	22.48	23.56	33.68	11.35	26.49	21.40	36.30	26.96	36.92	24.56	4.457	21.09	10.66	17.46	6.751	14.14	4.380	18.92	33.06	35.92	46.19	23.25	18.00	25.88	7.037	11.97	9.655	16.99	10.66	13.75	19.00
TG(53:5)	32.76	26.18	20.52	17.68	26.30	17.11	25.28	14.62	20.29	10.90	20.97	16.55	17.57	21.99	16.09	16.66	19.16	13.60	17.91	12.13	15.87	18.70	13.26	28.22	11.79	22.22	17.34	26.98	25.84	28.22	25.96	4.953	17.68	12.24	14.28	7.900	10.10	6.495	17.11	26.30	27.77	37.63	26.30	17.23	22.10	10.29	13.60	12.01	12.35	9.872	12.13	15.07
TG(53:6)	17.00	15.90	21.00	14.40	21.10	19.20	21.00	24.10	17.30	12.80	14.00	14.60	13.50	17.30	11.80	17.20	13.10	13.10	14.00	10.60	12.10	15.30	14.00	22.10	9.640	18.70	16.60	22.00	18.60	22.70	18.20	3.560	15.30	7.890	10.90	6.010	7.950	5.570	16.00	21.60	20.60	30.30	18.00	12.80	18.70	7.530	8.380	8.530	12.10	6.870	10.00	11.40
TG(54:2)	3.735	2.320	2.196	2.722	5.269	2.861	3.296	2.325	2.991	3.148	2.773	2.685	3.781	2.935	2.690	4.160	4.853	4.128	4.520	5.038	3.776	4.946	4.761	5.500	2.450	3.938	25.38	4.715	3.249	5.084	5.778	3.014	3.259	2.843	2.884	2.551	2.399	2.256	4.761	6.794	13.68	1872	6.933	5.084	4.899	2.131	2.746	2.639	2.935	2.145	2.635	3.125
TG(54:3)	356.6	489.5	436.8	358.6	452.4	476.8	406.5	503.2	384.0	292.1	344.9	345.9	316.6	364.4	255.0	387.9	347.8	289.2	408.4	267.7	183.7	339.0	319.5	506.1	193.5	439.7	314.6	425.0	370.3	473.9	385.9	79.05	338.1	174.9	203.2	115.3	187.6	86.18	308.8	547.2	506.1	624.4	386.9	314.6	409.4	147.5	289.2	229.6	318.5	162.2	274.6	405.5
TG(54:4)	266.8	309.0	308.0	246.6	287.9	294.9	303.0	309.0	271.8	180.2	225.5	232.5	200.3	253.7	172.1	236.6	268.8	195.3	279.8	195.3	135.9	247.6	239.6	381.5	134.9	311.1	200.3	274.8	287.9	317.1	250.7	54.96	221.5	140.9	140.9	88.69	105.7	65.53	232.5	397.6	350.3	378.5	310.0	222.5	293.9	122.8	203.3	165.1	220.5	129.9	201.3	262.7
TG(54:5)	165.3	214.9	204.8	160.2	195.7	185.5	193.6	212.9	169.3	124.7	156.1	152.1	134.8	182.5	116.6	161.2	176.4	128.8	189.6	123.7	80.40	176.4	168.3	268.7	96.9	201.7	133.8	203.8	197.7	215.9	172.3	35.28	152.1	86.07	88.71	56.57	76.04	39.23	166.3	276.8	272.7	290.0	209.9	168.3	217.0	78.77	135.9	98.95	140.9	75.53	127.7	167.3
TG(54:6)	164.8	193.3	175.4	169.0	214.5	207.1	188.1	218.7	151.1	126.8	149.0	146.9	127.8	157.4	114.1	165.9	141.6	110.9	169.0	113.0	80.72	150.0	158.5	232.4	87.2	194.4	159.5	219.8	198.6	214.5	159.5	30.32	143.7	88.53	86.63	58.00	78.39	35.29	147.9	247.2	273.6	312.7	191.2	151.1	194.4	69.83	103.4	84.31	124.7	67.62	109.9	146.9
TG(54:7)	106.8	118.8	100.0	93.1	153.0	145.3	106.0	144.4	93.14	84.25	93.99	90.57	78.53	104.2	69.38	100.0	77.07	60.67	89.72	61.69	50.84	89.72	88.01	138.4	49.30	105.1	88.87	123.0	110.2	104.2	91.43	12.48	77.33	52.89	62.21	40.25	50.76	24.61	84.08	111.9	129.9	73.49	68.79	85.45	58.45	36.23	65.11	63.40	71.01	15.89	65.11	13.67
TG(55:6)	37.30	32.10	28.40	37.40	43.20	43.80	31.40	39.50	26.80	21.80	28.70	23.50	18.50	22.70	20.90	27.40	22.10	19.60	24.20	25.30	17.10	26.10	25.20	34.50	13.10	34.80	30.00	35.30	36.30	37.00	25.40	4.980	26.60	17.70	18.30	11.60	16.00	11.70	20.10	34.60	35.70	0.311	34.30	16.80	0.082	0.076	13.70	12.40	17.80	11.30	16.80	0.074
TG(55:7)	228.1	246.0	282.5	248.1	295.3	311.3	38.42	314.9	168.5	254.3	222.9	228.1	157.2	238.8	126.4	152.0	154.1	114.0	178.7	95.02	82.70	180.3	147.9	180.8	23.17	197.8	237.8	311.3	307.7	230.1	243.0	0.079	160.3	86.80	108.9	70.37	83.21	39.34	141.8	189.0	208.0	320.5	103.8	125.8	193.6	43.8	70.37	34.41	57.53	53.93	62.15	63.18
TG(56:9)	3.527	3.628	3.857	2.945	4.899	5.560	3.893	4.698	3.355	2.658	3.333	2.952	2.622	4.059	2.212	3.204	2.816	2.277	2.981	2.521	1.803	3.663	3.060	6.400	1.810	4.181	2.916	5.043	4.511	4.727	3.886	0.713	2.722	2.126	2.040	1.422	1.681	0.634	1.487	2.873	3.944	3.491	2.773	1.983	2.981	1.315	1.379	1.990	1.595	0.884	1.631	2.076
LPC(14:0)	5.910	5.860	7.490	4.490	6.600	7.210	6.930	6.870	5.420	4.000	5.300	4.970	3.720	3.770	3.820	14.10	4.790	3.890	4.600	4.460	3.630	5.570	5.060	4.610	3.680	5.630	6.030	5.230	6.440	5.210	5.490	1.220	4.040	3.040	2.190	2.950	1.980	1.760	5.530	6.650	5.800	6.520	6.650	6.340	6.030	3.070	4.490	4.590	4.670	3.820	4.630	4.250
LPC(15:0)	1.243	1.344	1.547	0.947	1.310	1.370	1.471	1.386	0.998	0.719	1.065	1.082	0.667	0.717	0.770	3.069	1.023	0.794	1.040	0.837	0.679	1.403	1.116	1.040	0.718	1.175	1.251	1.048	1.319	1.057	1.277	0.226	0.862	0.844	0.414	0.631	0.375	0.364	1.226	1.539	1.598	1.606	1.420	1.454	1.885	0.639	0.888	0.854	0.896	0.854	0.964	1.023
LPC(16:0)	69.62	64.66	59.70	52.36	81.22	84.00	66.35	81.32	53.26	47.31	55.04	52.46	43.44	35.21	38.08	165.6	44.73	37.88	42.94	39.67	36.30	54.84	47.21	47.01	32.03	66.25	74.08	63.67	62.88	61.09	61.19	10.91	42.45	30.15	23.60	27.17	21.72	15.87	47.41	69.72	69.82	84.40	57.62	56.53	55.44	26.38	40.36	38.78	43.34	31.14	42.55	38.48
LPC(16:1)	7.211	7.121	8.118	5.280	8.324	9.608	7.758	8.692	6.016	4.499	6.007	5.594	4.167	3.852	4.238	10.24	5.289	4.499	5.100	4.696	3.933	6.160	5.666	5.307	3.592	5.433	6.995	6.061	6.456	6.178	6.447	1.212	4.535	3.224	2.227	2.999	2.074	1.697	5.873	7.570	7.597	9.788	6.941	7.229	6.941	2.945	4.723	4.247	4.939	3.951	4.993	4.247
LPC(17:0)	1.429	1.420	1.688	1.145	1.412	1.596	1.596	1.571	1.028	0.570	0.836	1.011	0.758	0.722	0.760	2.799	0.805	0.886	1.036	0.877	0.834	1.078	1.019	0.886	0.861	1.295	1.186	1.069	1.362	1.203	1.120	0.361	0.927	0.734	0.524	0.622	0.556	0.421	1.354	1.512	1.362	1.629	1.437	1.512	1.237	0.528	0.894	0.593	0.861	0.810	0.953	0.969
LPC(17:1)	2.665	2.408	3.001	1.885	2.912	3.494	2.754	3.099	2.014	1.560	2.112	2.162	1.392	1.244	1.510	2.626	1.856	1.471	1.836	1.589	1.313	2.241	1.945	1.875	1.382	2.024	2.576	2.399	2.389	2.270	2.517	0.472	1.836	1.303	0.907	1.135	0.810	0.661	2.408	3.169	3.238	3.899	2.833	2.803	3.099	1.086	1.757	1.658	1.885	1.530	1.856	1.935
LPC(18:0)	3.701	3.454	3.399	2.850	5.106	5.985	3.355	4.546	2.498	2.471	2.767	2.850	2.059	1.993	2.141	8.456	2.147	2.185	2.268	1.812	1.801	3.229	2.597	2.866	1.526	3.218	4.294	4.206	3.602	3.207	3.695	0.708	2.800	1.966	1.636	1.598	1.554	0.972	2.888	4.953	5.875	9.005	3.344	3.245	4.574	1.395	2.345	2.152	2.537	1.812	2.158	2.795
LPC(18:1)	19.69	18.41	22.71	13.14	21.33	25.88	20.82	23.42	14.99	11.81	15.29	15.55	10.59	9.922	11.61	20.71	14.17	12.02	14.01	12.12	10.43	17.13	14.58	15.91	10.69	13.40	18.67	17.70	17.80	15.96	17.08	3.918	12.43	9.718	6.598	8.337	6.342	5.268	20.97	26.60	26.60	37.08	25.83	25.16	28.39	9.104	15.60	13.45	14.78	12.63	13.86	15.24
LPC(18:2)	3.733	4.034	3.981	2.797	4.835	6.185	4.077	5.706	3.202	2.905	3.398	3.424	2.487	1.485	2.248	3.650	2.422	1.978	2.344	2.274	2.034	2.953	2.566	2.579	1.830	2.723	4.530	4.003	2.966	3.620	4.356	0.640	2.383	1.573	1.346	1.411	1.233	0.802	3.563	4.661	6.229	7.928	4.356	4.286	4.835	1.324	2.309	2.113	2.709	1.991	2.370	2.056
LPC(20:0)	0.473	0.431	0.464	0.378	0.539	0.694	0.567	0.677	0.310	0.312	0.377	0.448	0.242	0.355	0.282	0.969	0.380	0.263	0.321	0.289	0.270	0.421	0.352	0.495	0.259	0.437	0.492	0.556	0.441	0.383	0.447	0.087	0.345	0.160	0.248	0.192	0.184	0.153	0.299	0.540	0.661	0.876	0.431	0.327	0.368	0.162	0.211	0.201	0.253	0.164	0.171	0.290
LPC(20:1)	3.424	3.278	3.908	2.422	3.903	4.190	4.094	4.275	2.790	2.035	2.568	3.006	1.914	2.140	2.236	3.319	2.669	2.392	2.523	2.281	1.873	3.495	2.458	3.485	2.004	2.478	3.515	3.646	3.067	2.981	2.946	0.635	2.463	1.788	1.430	1.284	1.365	0.982	4.049	5.691	5.892	8.662	5.288	5.086	5.942	1.168	2.075	1.682	1.964	1.450	1.773	2.286
LPC(20:2)	0.938	1.215	1.087	0.692	1.239	1.407	1.134	1.458	0.915	0.692	0.955	0.972	0.638	0.614	0.726	0.891	0.715	0.661	0.712	0.695	0.580	0.908	0.709	0.938	0.601	0.672	1.164	0.999	0.834	1.114	1.080	0.213	0.857	0.446	0.402	0.523	0.446	0.299	1.343	1.782	1.846	2.707	1.579	1.654	1.782	0.422	0.837	0.682	0.877	0.668	0.786	0.719
LPC(20:4)	6.389	6.723	6.580	4.744	8.820	10.44	6.770	11.11	7.199	4.568	8.105	8.677	6.532	3.995	6.532	9.345	7.438	5.626	7.819	6.150	6.246	6.913	5.960	7.628	4.162	7.533	10.49	12.83	8.630	10.20	11.97	1.616	5.006	1.931	3.285	3.461	2.346	1.917	11.59	15.35	17.74	22.79	12.30	13.30	14.73	2.880	3.790	2.970	5.483	3.500	4.110	3.972
LPC(22:6)	88.47	83.68	95.88	63.20	96.75	104.2	87.17	108.5	64.50	53.17	65.81	67.12	47.07	40.14	51.86	64.07	60.14	50.56	55.79	54.48	44.45	71.04	61.02	67.99	44.02	57.97	79.76	80.19	64.94	77.14	80.19	15.34	54.48	42.06	28.76	33.86	26.89	21.53	106.8	145.1	141.2	187.4	118.1	126.0	134.7	33.47	56.66	50.12	60.58	47.94	57.53	54.91
LPC(24:1)	7.697	7.234	6.656	6.308	7.987	8.681	7.929	8.971	5.492	4.873	5.759	6.945	5.284	5.365	5.406	6.887	6.193	5.209	5.475	5.116	5.475	7.582	6.135	8.045	4.833	6.771	8.218	9.723	8.218	7.697	7.640	1.858	6.598	3.687	4.439	3.467	3.889	2.853	5.093	6.829	10.13	12.559	6.366	6.193	5.319	2.454	3.849	3.959	4.850	3.774	4.005	5.082
LPC-O(16:1)	1.993	2.067	2.352	1.477	2.014	2.457	2.088	2.341	1.361	0.968	1.223	1.107	0.725	0.807	0.976	2.732	0.936	0.961	0.679	0.836	0.967	0.782	0.615	1.086	0.909	1.192	1.413	1.529	1.698	1.909	1.318	0.280	0.920	0.600	0.618	0.421	0.394	0.343	1.049	1.234	1.519	2.025	1.234	1.540	0.875	0.905	1.160	0.927	1.361	1.035	1.993	0.754
LPC-O(18:1)	3.703	3.310	4.070	2.388	3.310	4.002	3.839	3.988	2.740	1.886	2.700	2.645	1.831	2.415	2.238	6.213	2.388	2.320	2.103	2.672	2.089	2.645	2.252	3.296	2.021	2.767	2.835	3.079	4.138	3.446	2.781	0.912	2.605	2.103	1.334	1.506	1.013	0.951	2.578	2.578	2.849	3.296	2.930	3.269	2.862	2.455	3.554	3.066	3.459	2.944	4.314	3.215
PC(24:0)	0.148	0.046	0.027	0.023	0.023	0.022	0.028	0.033	0.023	0.014	0.028	0.015	0.020	0.113	0.021	0.492	0.020	0.017	0.011	0.017	0.139	0.022	0.018	0.028	0.015	0.050	0.028	0.020	0.269	0.023	0.025	0.012	0.052	0.020	0.022	0.016	0.093	0.011	0.021	0.019	0.032	0.024	0.018	0.019	0.018	0.012	0.017	0.021	0.023	0.021	0.017	0.019
PC(28:1)	0.550	0.248	0.505	0.547	0.362	NF	0.606	0.274	0.411	0.322	0.342	0.371	0.182	0.314	0.370	0.534	0.276	0.238	0.264	0.349	0.417	0.389	0.268	0.440	0.274	0.384	0.634	0.544	0.382	0.453	0.455	NF	0.171	0.324	0.213	0.144	0.211	0.134	0.203	0.389	NF	0.019	0.322	0.286	0.336	0.097	0.352	0.257	0.260	0.211	0.303	0.263

PC(29:0)	1.261	0.684	0.626	1.018	1.216	0.681	0.851	0.657	0.739	0.657	0.602	0.596	0.632	0.629	0.529	0.568	0.675	1.550	0.541	1.680	0.626	0.730	0.532	0.851	1.580	0.830	1.550	0.948	0.660	0.918	0.720	0.887	0.517	1.340	0.553	1.530	1.470	1.240	1.580	0.720	0.641	1.440	0.571	0.629	0.693	1.610	0.605	0.641	0.799	1.600	0.647	0.590
PC(29:2)	NF	NF	0.258	0.192	0.301	0.319	0.284	0.249	0.246	0.190	0.224	NF	NF	0.217	0.207	0.244	0.188	0.183	0.199	0.206	0.201	0.258	0.241	0.291	0.169	0.271	0.238	0.297	0.300	0.261	0.251	0.115	0.218	0.196	0.150	0.157	0.152	0.121	0.206	0.245	0.290	0.359	0.232	0.193	0.251	0.147	0.199	0.176	0.166	0.145	0.174	0.269
PC(30:0)	8.689	8.361	9.426	7.262	10.74	10.57	11.39	10.74	8.525	7.689	7.730	7.951	6.566	7.049	6.131	7.098	6.467	7.566	6.959	8.607	7.189	7.828	8.008	11.39	6.107	7.771	7.746	8.771	7.918	8.525	7.639	3.361	6.934	5.828	5.271	5.344	4.967	3.820	6.279	9.098	8.853	9.180	7.853	7.525	8.689	6.893	9.180	7.615	8.525	6.975	8.041	7.926
PC(30:1)	15.50	15.40	18.30	13.40	20.10	17.50	20.10	19.10	16.10	15.10	14.90	14.40	13.20	14.30	12.20	14.70	13.00	14.00	13.60	15.80	13.60	15.30	15.20	20.40	11.30	16.70	17.30	18.00	16.20	16.80	17.20	6.50	14.10	11.60	10.80	11.40	10.00	7.610	10.60	15.70	15.40	17.00	13.30	12.60	15.40	12.30	16.40	14.50	15.60	13.10	14.50	15.40
PC(30:2)	1.570	1.490	1.700	0.974	2.200	1.290	1.600	1.640	1.760	1.260	1.520	1.010	0.918	1.490	1.050	1.210	1.420	1.180	1.200	1.350	1.140	1.490	1.570	1.290	0.750	1.360	1.690	1.240	1.970	1.280	1.230	0.560	0.995	0.917	0.949	0.506	0.752	0.421	0.995	1.320	1.490	1.070	1.120	0.953	1.750	0.858	1.160	1.000	1.110	1.040	1.050	1.280
PC(31:1)	6.570	6.380	7.300	5.850	8.610	8.180	8.700	8.100	6.660	6.200	6.470	6.750	5.520	6.010	5.140	5.850	5.930	6.050	6.070	6.550	5.850	6.820	6.460	9.410	5.150	7.000	7.060	7.720	6.750	7.450	6.640	3.380	6.200	5.420	4.720	4.820	4.460	3.550	5.290	6.910	7.170	8.670	5.890	5.760	6.830	5.580	7.050	6.500	6.610	5.580	6.200	6.710
PC(32:0)	5.130	7.703	NF	9.699	10.26	NF	0.529	14.59	11.78	12.02	0.035	10.98	8.176	7.415	4.305	11.86	8.898	7.928	6.196	14.91	7.182	10.50	9.218	NF	6.501	12.34	NF	5.795	3.848	11.78	4.898	0.133	8.336	6.894	6.052	NF	0.122	1.090	8.417	15.55	14.59	22.765	8.817	9.699	12.51	NF	NF	NF	0.294	NF	NF	NF
PC(32:1)	63.63	74.52	76.71	54.35	93.51	104.5	86.21	102.3	75.25	76.71	66.41	73.79	62.97	55.16	53.33	77.44	54.13	67.94	63.56	72.98	63.85	76.71	71.45	92.78	49.24	73.79	92.78	95.70	72.69	84.01	82.55	24.77	66.70	51.14	46.76	49.97	43.10	27.98	52.38	80.36	100.1	123.46	61.07	58.01	78.90	52.53	85.48	64.22	76.71	57.71	67.58	70.28
PC(32:2)	12.74	15.86	15.17	11.17	18.88	20.84	17.37	23.04	16.68	15.69	13.90	15.75	12.51	11.06	10.65	16.44	11.58	11.93	13.14	13.26	13.03	15.52	13.78	18.70	9.26	15.40	19.11	19.22	15.23	17.89	18.59	4.238	13.37	9.496	9.032	8.859	8.569	5.321	10.71	14.76	20.67	21.48	12.39	12.22	14.48	8.22	12.91	10.77	13.55	9.26	11.58	11.75
PC(32:3)	1.177	1.298	0.983	1.277	2.191	1.366	1.474	1.127	1.331	0.744	1.105	1.309	0.958	1.028	1.068	1.420	1.233	1.025	1.474	1.244	1.420	1.352	1.379	1.283	0.993	1.447	1.474	1.853	1.704	1.542	1.582	0.385	0.816	0.709	0.798	0.571	0.596	0.582	1.333	1.582	1.947	1.961	1.569	1.393	1.880	0.657	0.805	0.525	0.702	0.705	0.771	0.715
PC(32:4)	8.132	14.50	7.232	11.147	5.163	8.132	1.222	6.395	5.511	4.074	4.611	6.379	5.542	1.705	6.758	7.642	6.111	8.637	9.442	7.942	10.72	6.047	4.847	8.511	5.305	7.247	8.700	12.73	12.36	10.83	9.300	1.086	6.158	3.426	3.158	2.921	3.363	1.974	8.811	11.29	15.02	14.87	8.716	10.39	12.19	NF	3.000	4.295	3.347	3.426	3.205	NF
PC(32:5)	0.101	0.398	0.434	0.253	0.339	0.568	0.430	0.430	0.353	0.257	0.332	0.342	0.340	0.290	0.251	0.383	0.289	0.221	0.375	0.266	0.279	0.343	0.334	0.384	0.170	0.282	0.425	0.455	0.501	0.340	0.390	0.084	0.286	0.173	0.182	0.176	0.224	0.127	0.263	0.340	0.522	0.568	0.357	0.328	0.419	0.146	0.180	0.233	0.190	0.116	0.175	0.280
PC(32:6)	0.224	0.261	0.283	0.258	0.427	0.354	0.355	0.277	0.281	0.277	0.257	0.152	0.189	0.193	0.166	0.018	0.190	0.311	0.195	0.140	0.155	0.183	0.125	0.236	0.058	0.179	0.304	0.329	0.123	0.182	0.253	0.024	0.245	0.050	0.109	0.086	0.169	0.023	0.150	0.241	0.244	0.246	0.297	0.156	0.231	0.139	0.018	0.218	0.169	0.137	0.192	0.300
PC(33:0)	7.120	5.100	NF	3.860	8.520	NF	6.670	8.180	NF	8.040	NF	NF	6.250	2.600	2.270	6.190	5.010	4.820	3.630	4.920	NF	5.320	3.900	4.400	2.870	5.360	6.470	9.970	2.490	7.540	4.490	2.430	5.170	1.880	6.840	NF	3.090	2.870	1.760	7.750	4.910	8.210	3.030	7.390	6.590	1.560	NF	NF	6.350	4.450	3.740	NF
PC(33:1)	19.53	20.70	22.90	16.31	26.63	26.63	26.19	28.90	21.58	20.12	19.75	21.65	18.14	18.00	16.24	20.12	17.04	18.58	18.87	20.78	18.22	23.04	20.48	28.90	14.70	22.17	23.63	27.14	22.02	23.56	22.90	7.154	18.87	15.65	14.05	13.61	12.22	8.632	15.65	22.90	25.24	30.87	18.00	18.00	22.90	14.85	23.78	18.00	21.95	15.80	19.24	20.34
PC(33:3)	1.930	1.464	1.898	0.745	2.287	1.878	2.105	2.876	1.950	1.004	2.002	1.600	1.121	0.920	1.257	2.118	1.626	1.289	2.053	1.224	1.658	2.319	1.606	3.232	1.036	1.840	1.736	1.878	1.924	2.021	2.494	0.292	1.373	1.017	0.745	0.933	0.894	0.424	1.108	1.548	2.455	2.772	1.444	1.451	2.066	0.654	1.296	0.874	1.185	1.036	1.004	0.713
PC(33:4)	2.439	4.096	4.460	2.508	6.398	5.828	2.660	4.172	4.020	4.012	3.214	2.979	3.138	2.910	2.242	2.857	2.918	3.579	3.047	2.979	3.708	3.267	2.667	4.430	2.135	3.290	2.720	5.365	3.100	5.045	4.141	1.353	2.310	1.793	2.622	2.052	2.310	1.261	2.112	3.039	3.754	2.728	4.232	3.260	3.587	2.310	3.640	4.042	4.103	2.333	3.602	4.476
PC(33:5)	0.283	0.320	0.245	0.164	0.710	0.387	0.775	0.259	0.618	0.221	0.178	0.227	0.166	0.191	0.456	0.246	0.203	0.805	0.210	0.300	0.188	0.266	1.110	1.600	0.734	0.362	0.206	0.433	0.262	0.985	0.655	0.106	0.205	0.175	0.144	0.180	0.138	0.148	0.291	0.178	0.431	0.717	0.215	0.141	0.249	0.121	0.159	0.185	0.358	0.137	0.272	0.158
PC(34:1)	486.6	573.8	556.2	402.2	724.2	902.0	609.0	818.4	572.9	601.9	522.7	603.7	508.6	393.4	424.2	628.3	383.7	499.0	480.5	522.7	484.9	599.3	538.6	702.2	342.3	567.6	722.5	755.9	575.5	641.5	645.9	181.3	510.4	408.3	357.3	392.5	340.6	208.6	391.6	586.1	767.4	1012	447.0	418.0	601.0	389.0	643.3	480.5	601.9	440.9	491.9	513.0
PC(34:2)	445.0	623.7	404.1	422.1	773.9	935.5	505.8	882.3	508.7	619.9	473.5	534.3	520.1	275.7	347.0	686.4	312.8	329.0	365.1	378.4	394.6	431.6	379.4	512.5	242.4	522.9	825.3	798.6	438.3	659.8	693.1	113.1	449.7	300.4	307.1	279.5	299.5	146.4	306.1	501.0	827.2	1040	325.2	323.3	430.7	224.4	383.2	301.4	428.8	280.5	349.9	302.3
PC(34:3)	85.50	123.6	97.16	82.39	150.0	184.2	108.8	167.9	109.6	115.0	97.93	111.9	96.38	60.16	68.40	125.9	74.69	67.16	83.17	82.39	85.50	98.71	85.50	112.7	54.17	102.6	145.3	149.2	94.05	120.5	128.2	22.70	87.05	59.85	57.67	56.27	55.11	31.25	69.10	94.83	156.2	192.76	77.73	73.68	97.93	47.02	77.26	59.23	83.94	57.05	72.05	62.18
PC(34:4)	51.82	68.00	63.77	45.35	85.16	93.08	65.82	89.78	64.37	60.54	54.33	60.01	49.84	40.60	41.39	62.58	46.54	47.86	51.62	52.35	50.44	60.14	54.00	75.26	33.27	59.02	75.92	87.14	62.58	65.03	69.98	14.06	49.12	37.50	35.05	35.32	32.48	20.40	40.80	57.04	83.18	101.00	51.82	44.23	62.91	33.67	55.72	41.72	54.93	38.49	45.81	49.05
PC(34:5)	43.19	50.70	59.23	36.07	57.98	68.78	57.67	61.81	52.66	46.16	44.99	57.12	41.63	42.02	37.24	47.88	44.91	45.22	55.08	46.01	52.03	67.99	61.73	66.98	38.10	53.91	65.25	70.18	70.50	50.23	58.29	14.79	47.18	36.30	30.83	36.54	27.78	22.53	44.83	48.75	65.88	66.12	58.37	50.55	57.59	30.05	44.05	38.03	46.09	33.57	35.52	42.64
PC(35:0)	0.458	0.679	2.390	2.730	0.467	13.90	11.60	2.780	8.660	8.020	1.290	0.465	1.090	6.350	1.260	1.380	5.620	4.680	4.620	10.50	6.270	5.960	4.900	9.150	3.200	4.680	10.10	18.00	9.040	7.930	8.250	2.110	10.60	3.320	3.470	11.70	3.700	0.292	4.180	10.30	7.150	8.010	12.30	5.390	13.20	0.267	1.500	4.700	16.50	2.360	3.390	11.80
PC(35:1)	17.85	21.94	25.40	19.11	22.26	27.69	29.97	28.94	22.10	18.96	17.85	19.43	16.52	21.47	17.54	18.09	20.84	21.16	22.81	21.31	19.51	23.60	21.24	27.61	17.38	24.07	22.42	25.64	29.57	24.70	26.19	8.49	21.71	14.87	15.26	13.29	13.06	9.36	21.08	21.31	24.93	30.20	21.47	19.27	21.79	14.39	24.93	20.21	20.84	14.47	20.53	23.52
PC(35:2)	24.12	28.17	32.12	21.10	31.91	38.46	33.26	37.94	29.21	25.26	24.95	26.71	21.62	22.76	21.41	25.26	24.53	24.22	27.44	27.03	25.47	30.66	27.44	36.90	20.27	28.38	29.00	33.16	30.98	29.73	30.98	9.28	27.34	20.37	17.77	17.77	16.22	11.95	23.28	28.69	31.50	38.56	25.05	23.49	30.77	20.17	32.01	24.95	29.42	22.24	24.74	27.75
PC(35:3)	3.351	4.722	4.113	3.097	5.459	5.992	4.697	6.170	4.367	3.732	3.656	4.037	3.428	2.894	2.945	4.164	3.199	3.174	3.605	3.250	3.428	3.910	3.478	4.824	2.382	4.189	5.154	5.586	4.088	4.849	4.849	1.115	3.580	2.539	2.445	2.260	2.125	1.572	2.920	4.138	5.230	6.119	3.428	3.174	4.088	2.219	3.732	2.767	3.732	2.615	3.123	3.047
PC(35:4)	5.469	7.236	6.816	3.955	7.910	7.952	7.278	7.952	6.900	5.890	5.806	6.732	5.343	4.094	5.091	6.689	5.554	5.596	6.058	5.638	5.427	7.363	6.437	9.340	4.375	6.858	7.278	9.634	7.615	7.236	8.625	1.397	5.469	4.460	3.627	4.754	3.677	1.952	4.754	6.479	8.162	9.298	7.363	5.259	6.984	4.838	8.120	6.437	6.858	5.427	5.680	6.016
PC(35:5)	5.947	7.510	8.615	5.108	7.700	8.996	8.615	8.463	7.433	6.290	6.519	8.539	5.718	6.404	5.032	6.557	6.671	6.252	8.310	6.480	7.357	9.988	8.691	10.22	5.756	7.205	8.958	9.606	10.10	6.785	7.891	2.146	6.328	5.680	4.308	5.108	3.793	3.362	6.900	6.747	8.768	8.996	9.568	7.433	8.043	4.460	6.061	5.566	6.176	4.841	5.032	6.709
PC(36:0)	0.010	NF	NF	NF	NF	0.010	NF	0.022	NF	NF	NF	NF	NF	0.014	0.008	NF	NF	NF	NF	0.010	0.010	NF	0.011	NF	0.013	NF	NF	NF	NF	7.430	NF	NF	5.410	0.010	NF	NF	NF	0.006	NF	0.024	0.024	NF	NF	5.190	0.021	4.040	0.009	4.190	5.110	4.770	5.650	NF
PC(36:2)	204.0	282.1	269.9	175.8	344.1	458.9	272.7	391.2	268.0	266.1	238.8	269.9	226.6	179.6	194.6	283.0	196.5	199.3	224.7	215.3	206.9	267.0	230.4	318.8	155.1	231.3	322.5	344.1	259.5	296.2	291.5	70.80	234.1	181.5	156.1	162.7	157.0	95.0	186.2	268.9	377.1	527.5	220.0	202.2	304.7	148.6	267.0	189.0	250.1	182.4	205.9	213.4
PC(36:3)	219.0	338.7	245.6	216.3	387.5	515.1	262.4	423.8	270.4	298.8	243.8	289.0	259.8	152.5	193.3	315.6	182.6	179.1	211.0	209.2	213.7	253.6	213.7	297.9	140.1	263.3	381.3	379.5	253.6	324.5	335.2	61.89	234.1	180.0	157.8	168.5	153.4	89.6	174.7	284.6	407.0	526.7	204.8	201.3	255.4	135.7	218.1	178.2	249.1	165.8	214.6	177.3
PC(36:4)	322.2	362.3	39.0	255.5	420.3	513.0	326.2	406.3	310.9	220.2	273.5	320.2	291.5	187.5	230.8	342.2	224.8	245.5	299.5	255.5	296.9	295.5	216.8	386.9	167.5	328.9	452.3	498.4	378.9	356.9	389.6	79.39	306.2	251.5	182.1	293.5	182.8	119.4	213.5	306.2	454.3	532.4	280.9	242.8	325.6	4.196	341.6	264.9	307.6	189.5	240.8	363.6
PC(36:5)	103.6	124.3	127.6	87.98	155.0	179.2	127.4	147.1	114.4	121.4	107.5	135.8	114.1	93.17	86.53	127.7	94.74	96.55	121.1	97.52	118.4	148.2	133.6	151.1	82.43	129.1	177.5	183.6	162.4	128.8	140.6	33.79	110.6	91.60	76.88	87.02	68.91	49.60	93.17	109.5	167.4	177.1	123.2	106.2	124.4	68.31	98.36	82.07	102.8	74.47	77.6	98.8
PC(36:6)	1.645	1.930	2.266	1.350	2.199	2.246	2.246	2.390	2.028	1.687	1.749	1.909	1.490	1.707	1.511	1.775	1.697	1.785	1.945	1.956	1.733	2.282	2.106	2.665	1.397	1.925	2.214	2.370	2.302	2.142	2.152	0.647	1.821	1.433	1.169	1.350	1.050	0.911	1.723	2.287	2.297	2.390	2.044	1.930	1.945	1.190	1.733	1.609	1.899	1.345	1.552	1.599
PC(37:1)	1.303	1.972	2.111	1.442	1.845	2.007	2.226	2.468	1.765	1.407	1.338	1.453	1.269	1.707	1.499	1.407	1.511	1.788	1.592	1.903	1.626	1.880	1.799	1.995	1.280	1.926	2.030	1.995	2.434	1.730	2.007	0.784	1.822	1.063	1.113	1.110	1.104	0.795	1.719	1.615	1.857	2.099	1.546	1.626	1.522	1.052	1.799	1.557	1.430	0.978	1.626	1.626
PC(37:2)	13.86	17.47	18.43	12.54	18.24	20.56	19.37	22.77	18.27	14.47	15.03	15.19	13.55	15.26	12.85	14.94	13.83	14.33	15.50	15.05	14.77	19.65	13.18	17.89	11.44	17.28	20.82	23.68	22.89	17.94	17.19	6.331	15.78	10.11	10.50	10.18	10.13	7.597	13.04	14.68	19.63	25.79	14.23	13.79	17.21	9.379	17.35	13.90	13.53	12.85	13.93	15.31
PC(37:3)	9.210	15.00	13.90	10.10	20.10	20.20	16.00	15.70	12.70	10.30	11.10	11.50	8.94	11.30	10.30	9.150	10.70	11.80	11.20	12.70	11.90	16.10	12.20	16.90	7.70	14.10	14.80	16.90	16.60	15.30	15.40	3.920	11.40	8.330	6.320	8.510	7.950	4.880	9.570	12.50	15.40	18.00	11.30	10.90	15.10	7.390	13.10	10.10	10.70	8.160	10.60	12.40
PC(37:4)	22.10	26.30	31.80	20.80	32.50	32.50	29.80	33.50	24.30	20.40	19.20	21.00	18.50	18.40	17.60	21.30	18.90	17.70	19.50	18.40	18.80	22.80	20.70	27.30	14.60	21.80	29.20	29.10	25.90	22.80	24.50	7.260	19.70	15.20	13.40	13.10	13.80	9.020	16.40	21.20	28.30	32.80	18.40	15.40	20.20	12.30	29.20	18.30	20.80	12.90	19.00	24.20
PC(37:5)	16.98	23.30	23.07	16.67	28.59	28.21	23.92	25.76	19.89	17.86	17.25	20.70	16.86	17.98	14.83	19.28	15.98	16.63	18.90	17.71	17.63	21.85	20.05	25.11	13.30	18.44	25.72	27.14	23.57	21.58	22.65	5.289	18.21	15.71	12.04	13.91	11.15	8.164	14.72	20.54	24.57	27.83	19.47	17.25	19.97	17.32	24.30	17.52	20.85	15.37	15.25	18.78
PC(37:6)	48.39	53.71	61.52	38.81	60.34	61.52	63.89	63.89	55.13	45.31	47.03	52.35	41.94	50.52	41.94	47.50	49.81	50.16	57.56	51.29	49.16	68.03	59.75	78.68	41.88	55.13	62.70	66.85	68.03	60.93	58.80	17.33	50.46	42.95	33.30	38.04	28.51	25.50	51.17	67.44	65.66	67.44	60.93	57.79	58.21	35.91	50.70	44.43	52.53	36.85	43.48	49.75
PC(37:7)	4.709	5.066	5.976	3.995	6.495	6.528	5.878	6.885	5.683	4.742	4.709	4.871	4.157	4.189	3.995	4.871	4.709	4.319	5.424	4.579	4.677	5.878	5.099	6.820	3.280	5.131	6.138	6.755	6.300	5.846	6.008	1.367	4.644	3.475	2.910	3.140	2.786	2.121	4.287	5.878	6.950	6.755	5.716	5.326	5.326	2.575	3.995	3.572	4.514	3.114	3.637	3.800
PC(38:0)	4.221	4.756	3.442	4.361	4.663	3.652	3.349	3.373	5.629	6.478	3.105	4.687	4.884	5.931	5.303	4.326	5.001	5.268	5.605	4.954	6.396	5.861	5.256	6.303	5.570	5.524	3.989	3.780	6.268	6.012	4.059	2.954	5.850	11.62	5.454	5.187	4.419	4.140	6.350	4.035	3.814	5.291	4.756	5.012	5.163	5.233	7.268	6.396	4.663	6.850	5.850	7.268
PC(38:1)	19.30	25.60	23.40	18.70	29.50	29.30	24.70	27.40	25.50	22.00	18.60	25.20	18.00	21.50	18.30	22.90	20.40	18.80	21.70	19.40	21.60	27.10	22.80	29.50	17.40	23.40	24.90	26.30	25.40	25.70	21.90	7.84	21.80	25.20	17.80	17.30	15.60	10.80	21.80	24.60	26.60	34.40	20.70	20.80	25.30	17.10	25.50	19.40	21.20	20.70	19.50	26.00
PC(38:2)	23.04	31.14	31.35	21.23	38.58	42.46	32.67	38.30	29.88	24.02	23.97	28.18	22.11	23.04	21.18	27.52	20.85	21.01	22.98	22.22	21.94	31.79	23.47	34.09	17.67	24.90	33.54	37.48	28.29	31.24	29.11	7.82	27.14	18.06	18.22	18.11	19.04	10.40	20.57	29.71	35.24	45.58	24.51	21.50	33.38	16.20	27.69	19.04	26.05	18.82	20.52	26.54
PC(38:4)	144.3	160.3	104.7	99.97	7.27	334.8	146.2	175.4	125.4	91.10	129.2	144.3	132.0	78.94	100.9	145.2	94.31	95.25	107.5	105.6	115.1	195.2	151.8	153.7	72.43	110.3	198.1	211.3	150.9	152.8	158.4	25.56	149.0	108.5	76.30	132.0	77.81	35.74	91.20	140.5	209.4	236.7	85.73	101.9	116.0	57.43	114.1	85.26	120.7	76.01	100.9	111.3
PC(39:4)	7.566	9.549	9.989	7.110	9.475	9.696	10.43	12.27	8.374	7.272	7.022	7.257	6.728	8.227	7.316	6.207	7.419	7.786	7.859	7.566	7.712	8.447	8.227	10.577	5.957	8.594	9.108	10.43	12.05	9.255	11.02	2.718	7.786	4.929	5.120	5.259	5.391	3.364	6.743	7.117	9.108	8.080	8.227	7.566	8.006	4.943	7.492	7.198	7.419	5.413	6.912	7.786
PC(39:5)	8.875	10.43	10.64	7.227	7.745	11.94	11.21	10.90	8.522	4.591	7.557	7.769	6.521	6.545	6.686	6.592	8.287	7.651	8.169	8.546	7.510	9.393	7.392	13.231	6.215	8.098	8.522	12.24	11.39	9.276	9.982	1.817	9.841	6.427	5.744	6.286	5.014	3.814	7.063	8.805	9.417	10.64	7.934	6.874	7.439	0.039	9.228	5.721	8.993	6.592	7.016	9.464
PC(39:7)	4.759	5.167	5.881	3.841	6.663	6.969	5.813	6.799	5.303	4.759	4.419	5.065	4.181	4.283	4.011	4.895	4.283	4.453	4.963	4.521	4.419	5.677	4.963	6.765	3.467	5.235	6.187	6.629	6.051	6.255	5.643	1.513	4.895	3.807	3.399	3.433	3.110	2.250	4.113	5.949	6.323	7.343	4.997	4.725	5.303	2.971	4.725	4.079	5.201	3.501	4.045	4.351
PC(40:1)	0.961	1.652	2.045	0.033	0.848	2.599	0.063	3.424	0.901	1.430	3.455	1.974	0.850	1.259	1.561	1.410	1.370	0.313	1.239	2.729	2.357	0.147	1.239	4.462	1.299	1.551	1.440	0.182	1.934	1.692	2.367	0.104	0.030	1.158	0.975	1.561	0.929	0.690	0.821	3.455	3.878	5.539	3.011	2.327	4.129	0.076	1.813	1.410	1.783	1.692	1.692	2.196

PC(40:2)	1.626	10.31	10.13	9.085	13.10	11.98	24.62	17.84	19.51	12.35	7.599	11.80	11.98	11.80	9.568	15.51	13.19	11.24	15.61	14.49	14.49	17.93	15.33	17.37	11.98	15.42	17.37	20.90	17.28	17.00	17.37	3.799	15.98	10.31	8.481	10.40	10.59	3.177	15.61	18.95	20.16	24.90	18.67	14.86	22.94	10.03	12.54	11.24	10.96	8.806	3.771	11.98
PC(40:3)	0.382	2.090	0.248	0.221	1.720	7.750	14.40	3.270	7.400	1.650	0.979	18.00	13.30	10.50	4.820	12.20	12.00	4.230	11.90	9.470	12.60	5.980	6.920	17.00	3.070	13.40	15.60	25.40	13.80	19.00	11.90	0.042	22.30	11.50	6.630	15.80	10.80	0.044	10.20	19.10	17.70	22.00	14.20	11.00	17.80	11.10	4.470	0.516	3.220	0.123	14.90	13.80
PC(40:4)	22.86	24.30	28.37	20.49	35.34	43.48	26.27	36.91	28.24	23.65	25.88	29.56	24.83	19.57	22.07	30.35	18.52	19.05	21.02	19.44	21.68	27.72	21.68	34.02	15.37	26.67	37.44	41.77	29.95	27.98	24.30	5.622	24.83	17.87	18.26	20.23	18.52	11.05	22.20	31.53	37.18	45.85	26.54	24.83	31.13	13.14	22.46	20.76	23.25	16.68	19.31	24.04
PC(40:5)	0.042	0.338	NF	NF	0.424	0.595	0.607	0.594	0.529	0.413	0.364	0.335	0.302	0.284	0.254	0.336	0.400	0.311	0.355	0.357	0.264	0.365	0.416	0.436	0.256	0.275	0.421	0.527	0.219	0.445	0.493	0.156	0.116	0.236	0.208	0.188	0.194	0.156	0.379	0.352	0.619	0.756	0.658	0.484	0.464	0.132	0.199	0.253	0.272	0.198	0.191	0.230
PC(40:6)	NF	1.021	1.224	1.036	1.389	3.101	2.027	1.734	1.960	1.907	1.381	1.682	1.682	1.547	1.201	2.095	1.487	1.389	1.419	1.321	1.209	1.839	1.464	1.426	1.074	1.457	2.500	3.183	3.341	2.763	2.688	0.546	1.261	1.171	1.164	1.081	1.179	0.514	1.704	2.327	4.212	5.818	2.485	1.697	2.545	0.514	0.856	0.938	1.171	0.749	0.818	1.014
PC(41:5)	20.27	19.09	NF	18.35	20.57	7.298	28.23	3.516	20.79	15.19	21.16	20.57	18.13	21.08	13.12	19.76	19.09	22.85	19.46	26.17	23.22	0.752	18.65	23.51	19.98	16.95	19.90	19.53	30.44	18.13	16.95	0.759	24.47	9.288	14.67	14.23	15.85	11.94	17.69	15.92	15.48	10.69	21.08	15.77	17.10	13.05	23.66	21.52	21.45	14.89	18.72	20.05
PC(42:1)	0.047	0.027	0.020	0.025	0.034	0.027	0.025	0.044	0.022	0.024	0.017	0.028	0.016	0.013	0.014	0.023	0.042	0.020	0.019	0.025	0.015	0.029	0.015	0.034	0.015	0.019	0.027	0.037	0.030	0.032	0.026	0.008	0.015	0.015	0.015	0.014	0.012	0.009	0.382	2.590	2.890	5.320	0.847	1.090	2.240	0.072	0.258	0.452	0.944	0.032	0.017	1.390
PC(42:2)	4.335	3.243	4.724	2.551	6.346	5.470	7.210	5.362	6.140	5.978	3.697	5.459	1.438	3.784	4.378	4.681	5.167	4.832	2.249	2.724	6.054	7.362	2.303	7.394	3.416	5.243	5.005	8.162	5.254	5.438	5.416	1.967	6.940	1.243	2.951	3.751	5.546	0.926	6.227	14.49	14.27	22.16	9.351	9.232	15.46	2.346	8.540	4.216	7.718	5.124	4.497	10.58
PC(42:4)	0.904	1.365	0.775	0.976	2.109	1.491	0.855	1.706	0.891	1.419	0.980	1.061	1.527	0.877	1.155	1.352	0.958	1.048	0.851	0.886	1.079	1.791	1.124	1.298	0.689	1.330	1.482	2.538	1.160	1.316	1.683	0.369	1.124	0.833	0.842	1.343	0.940	0.300	1.204	3.134	2.659	4.052	1.746	1.553	2.610	1.254	1.562	1.160	1.598	1.254	1.267	2.100
PC(43:6)	1.545	1.456	1.417	1.238	2.115	2.635	1.828	1.907	1.694	1.570	1.263	1.516	1.263	1.521	1.342	1.402	1.590	1.501	1.654	1.288	1.471	2.065	1.283	2.169	1.412	1.322	2.046	2.026	2.234	1.540	1.788	0.555	1.793	1.134	1.144	1.218	1.105	0.877	1.382	1.902	2.100	1.758	1.763	1.595	1.639	1.149	1.431	1.352	1.833	1.213	1.402	1.605
PC(44:5)	2.313	2.401	2.409	1.456	2.821	2.688	2.361	3.034	2.222	2.009	1.946	2.020	1.674	1.624	1.754	1.993	1.828	2.260	2.105	2.079	2.127	2.928	2.244	3.087	1.757	2.302	2.874	3.673	3.087	2.901	2.821	0.791	2.151	1.570	1.408	1.882	1.453	0.809	2.020	3.487	4.072	5.589	3.220	2.640	4.072	1.469	2.901	2.172	2.326	1.900	1.778	3.247
PC(44:7)	8.452	7.785	7.859	6.228	9.861	8.601	10.01	11.20	8.452	6.881	6.977	6.591	6.584	6.792	6.258	7.489	6.636	6.717	5.546	7.177	6.458	8.452	6.265	10.677	5.561	8.156	8.971	10.97	9.046	9.935	7.637	2.469	7.355	4.597	5.279	4.004	4.760	3.188	6.310	10.08	9.342	10.973	7.711	7.044	7.288	3.900	7.058	5.932	6.836	5.294	5.464	7.859
PC(44:10)	0.256	121.5	0.143	0.192	0.245	147.2	0.264	146.6	118.9	0.264	0.212	97.71	99.00	0.289	102.9	117.0	91.93	0.272	91.93	84.86	99.64	0.253	0.203	0.301	0.172	115.7	133.1	153.6	133.1	143.4	133.1	0.077	0.244	0.172	94.50	68.79	0.134	0.094	79.71	1.794	0.482	123.4	122.1	100.3	132.4	0.094	0.163	0.168	0.209	0.149	0.165	0.192
PC(46:2)	0.184	0.337	0.168	0.092	0.357	0.218	0.079	0.068	0.180	NF	0.148	0.231	0.266	0.104	0.081	NF	0.886	0.143	NF	NF	NF	0.257	NF	0.165	NF	NF	NF	NF	0.222	NF	0.098	0.031	0.210	0.073	NF	NF	0.096	0.027	0.637	9.305	2.595	46.52	16.12	6.055	22.80	0.609	6.107	4.417	6.645	4.312	0.160	11.94
PC-O(26:0)	0.057	0.054	NF	0.199	0.116	0.093	0.173	0.110	0.109	0.139	0.134	0.153	0.173	0.064	0.061	0.521	0.087	0.114	0.093	NF	0.143	0.127	0.201	0.127	0.106	0.185	0.131	0.184	0.120	0.108	0.073	0.039	0.172	0.094	0.036	0.077	0.065	0.057	NF	0.525	0.141	0.270	0.343	0.046	0.229	0.093	NF	0.180	NF	NF	0.234	0.091
PC-O(26:1)	0.443	0.505	0.438	0.500	0.580	0.599	0.620	0.614	0.427	0.317	0.404	0.428	0.403	0.360	0.422	0.541	0.423	0.389	0.360	0.359	0.378	0.486	0.404	0.515	0.281	0.492	0.555	0.700	0.474	0.624	0.531	0.170	0.439	0.336	0.303	0.264	0.305	0.179	0.326	0.565	0.576	0.899	0.425	0.433	0.440	0.238	0.305	0.381	0.464	0.342	0.311	0.447
PC-O(28:1)	2.870	2.400	2.400	2.340	3.400	3.000	3.550	2.980	2.360	2.240	2.460	2.250	1.820	2.010	2.160	2.380	2.160	2.400	1.730	2.430	2.350	2.630	2.410	2.930	1.790	2.480	3.250	2.900	2.680	3.070	3.050	0.855	2.380	1.530	1.600	1.280	1.520	1.010	1.450	1.720	2.410	2.640	1.700	1.910	1.960	1.540	1.990	1.770	2.410	1.540	1.780	1.580
PC-O(30:1)	5.600	5.660	5.480	4.800	6.680	6.650	7.680	6.100	4.940	4.350	4.950	4.570	3.920	3.970	4.190	4.240	4.610	5.980	3.890	5.810	4.840	5.560	5.050	6.170	3.970	5.300	7.040	6.370	5.530	6.410	6.440	2.230	4.840	3.340	3.050	2.920	2.950	2.200	2.870	3.180	4.210	3.740	3.270	3.390	3.470	3.150	4.210	3.620	4.440	3.280	4.220	3.490
PC-O(30:2)	4.120	3.730	3.630	3.400	5.000	5.040	4.700	4.560	3.600	3.480	3.580	3.280	3.030	3.140	3.010	3.130	2.980	3.590	2.620	3.780	3.540	3.690	3.430	4.260	2.540	3.580	4.430	4.660	3.580	4.300	4.340	1.670	3.530	2.380	2.260	1.890	2.220	1.740	2.070	2.380	3.570	3.960	2.080	2.280	2.230	2.140	2.800	2.250	3.110	2.090	2.690	1.960
PC-O(31:1)	0.052	NF	NF	0.238	NF	0.073	NF	0.065	0.078	NF	0.197	NF	NF	NF	0.753	NF	1.710	1.020	NF	2.550	NF	2.760	2.120	0.771	1.240	0.983	2.790	1.420	NF	2.660	2.380	NF	NF	0.037	0.038	NF	1.270	0.073	1.060	NF	1.950	NF	1.120	1.350	0.869	0.162	0.094	0.133	0.693	NF	1.210	2.180
PC-O(31:3)	0.198	0.221	0.092	NF	0.320	0.163	0.124	0.062	0.346	0.114	0.138	0.184	0.111	0.124	0.148	0.079	NF	0.099	0.250	0.114	0.181	0.181	0.119	0.291	0.172	0.229	0.125	0.172	0.063	0.099	0.023	NF	0.081	0.157	NF	0.035	0.049	0.035	NF	0.120	0.304	0.112	0.058	0.089	0.096	0.058	0.120	0.114	0.178	0.075	0.110	0.133
PC-O(32:1)	NF	NF	NF	2.034	3.610	NF	NF	NF	0.644	NF	NF	4.807	1.685	1.991	1.781	3.044	2.978	4.759	2.912	4.187	3.736	4.675	4.247	4.951	2.737	3.766	7.701	6.197	4.175	4.927	4.765	2.046	4.488	2.052	1.661	2.503	3.820	NF	2.461	3.086	2.557	3.159	2.761	2.876	3.670	2.810	0.326	2.232	1.937	1.486	3.495	4.260
PC-O(32:2)	5.624	5.850	6.395	5.624	8.027	9.252	8.073	7.846	6.122	5.079	5.533	5.125	4.104	5.261	4.581	5.034	4.358	5.669	4.295	5.261	5.533	5.170	4.372	6.440	4.236	5.442	6.757	7.710	6.803	6.259	6.440	2.172	5.306	3.723	3.905	2.816	3.751	2.422	3.991	4.898	6.576	8.526	4.531	4.626	4.943	3.061	5.669	4.209	5.261	4.467	4.413	4.581
PC-O(33:2)	3.730	4.040	6.220	4.050	8.760	8.670	6.620	7.220	6.250	5.670	5.760	4.820	3.710	5.240	3.890	4.340	4.190	4.550	4.190	4.430	4.870	4.690	4.510	6.350	4.110	5.110	7.560	6.550	6.530	5.190	6.220	1.990	4.820	3.500	3.760	2.610	3.420	2.160	4.580	6.050	7.480	9.080	4.820	4.770	5.470	2.720	6.110	4.440	5.150	3.970	4.190	4.670
PC-O(33:3)	1.550	1.430	1.660	1.470	1.840	2.050	2.480	1.980	1.710	1.180	1.680	1.340	0.803	1.450	1.210	1.220	1.170	1.380	1.210	1.380	1.390	1.360	1.070	1.680	1.100	1.330	1.530	2.020	1.810	1.520	1.420	0.449	1.330	0.902	0.873	0.633	0.817	0.495	1.050	1.050	1.180	1.580	1.020	1.140	1.050	0.679	1.420	1.050	1.140	0.882	1.070	1.090
PC-O(34:1)	9.627	10.72	12.47	8.050	13.61	15.05	13.30	12.99	11.54	9.895	10.41	10.62	7.545	9.153	8.514	8.658	8.112	12.37	8.596	12.16	9.916	13.61	11.34	12.99	8.050	11.03	12.88	16.70	15.56	13.50	10.72	4.247	11.44	8.627	7.091	7.483	6.957	4.422	8.503	9.926	11.34	12.27	9.709	9.256	12.16	8.277	13.50	10.41	13.71	11.54	10.82	15.36
PC-O(34:2)	21.00	23.80	27.80	19.20	30.70	40.80	28.90	32.90	25.80	23.70	23.00	21.70	18.40	23.40	20.10	22.60	17.90	24.10	16.40	22.20	24.00	22.90	18.10	28.70	16.90	23.70	25.70	31.60	31.60	29.90	24.80	7.770	24.80	15.10	16.50	11.30	15.80	8.95	18.00	20.30	30.00	40.90	19.70	21.90	23.00	13.50	27.30	16.50	26.80	20.20	20.50	22.10
PC-O(34:4)	4.837	5.453	6.217	4.804	7.416	8.725	5.671	6.707	6.053	6.326	4.450	4.303	4.493	3.872	4.030	3.632	4.799	6.380	3.872	6.053	5.290	6.598	4.722	6.162	3.359	5.279	6.544	7.852	6.707	6.053	5.415	1.636	5.344	2.781	2.536	3.337	3.970	1.347	3.512	3.670	4.821	5.889	5.186	4.608	6.217	3.583	5.671	3.512	6.925	3.752	5.208	4.297
PC-O(35:3)	0.008	0.011	NF	NF	0.014	NF	NF	NF	NF	NF	NF	NF	NF	NF	NF	NF	1.770	1.783	1.845	NF	2.107	NF	NF	NF	1.988	NF	NF	NF	NF	NF	NF	NF	NF	NF	NF	1.346	1.552	NF	1.864	NF	NF	NF	NF	NF	NF	1.053	1.677	1.484	2.225	1.652	NF	1.907
PC-O(35:4)	2.260	3.730	2.220	2.510	0.731	5.680	4.000	7.180	3.240	2.850	3.980	5.020	0.812	3.340	2.630	5.100	3.370	3.760	2.560	3.810	3.750	4.630	2.130	4.900	2.120	1.670	5.100	5.730	3.160	2.970	3.770	0.891	4.290	2.330	2.430	1.690	2.790	1.920	0.017	2.030	9.010	*NF*	3.200	2.590	5.060	2.090	3.980	2.560	3.550	2.470	2.990	3.470
PC-O(36:2)	15.36	15.68	19.88	12.94	20.30	24.83	24.20	22.41	21.78	14.20	15.89	18.09	10.63	18.09	13.78	13.47	15.04	19.78	14.62	18.73	15.89	18.62	16.10	22.09	14.73	17.78	17.57	23.14	24.93	21.25	15.46	7.164	18.09	14.62	10.84	10.14	9.868	7.375	16.20	16.83	19.36	25.14	16.10	16.10	18.83	13.89	23.57	17.57	23.57	18.52	18.83	22.62
PC-O(36:3)	6.564	7.490	8.332	5.879	8.151	10.14	10.05	8.488	8.260	5.482	6.576	7.430	4.953	7.358	6.396	5.831	6.216	8.741	5.903	8.007	6.649	8.644	7.250	9.426	5.687	6.961	7.562	9.258	9.582	8.825	6.925	2.465	8.067	5.567	4.689	4.136	4.028	2.585	5.579	6.204	7.190	8.176	6.228	5.879	6.841	5.266	8.452	6.997	8.548	7.093	7.406	7.947
PC-O(36:4)	16.43	18.15	19.32	13.36	20.85	23.65	20.49	20.22	17.97	15.89	15.62	15.35	12.46	13.63	13.99	15.44	13.81	18.51	15.17	18.06	15.89	20.04	16.61	22.57	13.00	18.78	19.68	26.72	23.20	22.30	21.31	6.301	17.97	12.55	11.38	11.47	9.93	7.249	12.73	17.42	20.76	20.04	16.61	15.17	17.24	9.57	18.60	15.35	16.70	15.80	15.17	16.52
PC-O(36:5)	46.48	53.60	57.68	37.76	59.60	70.88	59.76	54.80	53.36	44.80	43.04	51.84	40.64	43.68	41.92	42.88	41.28	54.16	45.68	52.40	51.20	62.08	51.76	64.40	38.56	55.04	61.20	79.68	76.72	57.68	53.84	18.08	49.76	38.16	31.44	33.04	29.68	20.96	34.64	39.52	55.20	61.04	47.44	41.76	48.72	34.96	55.44	41.12	54.32	42.48	43.76	51.76
PC-O(36:6)	9.703	9.797	9.916	7.812	11.20	12.76	11.44	10.91	10.38	8.320	8.403	8.568	7.209	8.521	7.457	8.533	7.635	9.242	7.103	8.663	9.100	10.91	7.765	11.43	6.157	10.45	10.74	13.24	11.44	13.00	9.502	2.612	8.521	5.673	5.720	3.995	4.869	3.451	5.496	7.765	10.16	11.31	4.751	6.701	7.032	5.023	7.942	5.377	9.608	6.890	7.363	6.996
PC-O(37:6)	14.48	15.70	24.38	12.07	13.71	18.98	18.59	15.52	14.02	11.79	15.98	12.63	8.83	10.81	10.12	12.84	9.28	12.45	12.21	14.13	10.92	13.57	0.078	15.91	8.90	13.29	11.97	17.37	13.71	16.05	13.26	5.756	12.21	10.47	7.570	0.152	7.884	0.057	6.279	15.45	13.78	15.77	0.138	8.477	9.523	3.321	11.30	6.244	12.42	9.802	11.62	10.67
PC-O(37:7)	84.90	88.60	96.60	63.60	102.0	112.0	92.60	104.0	81.20	73.20	73.80	75.00	65.30	69.20	69.70	70.20	65.10	72.50	53.30	72.80	77.70	75.80	53.50	95.90	47.80	74.50	83.10	94.80	89.20	91.60	87.50	22.10	68.80	43.80	45.80	27.90	42.00	28.50	47.40	61.80	82.80	102.00	55.80	60.50	53.50	45.10	68.70	44.50	74.40	55.30	62.80	42.10
PC-O(38:4)	4.414	3.626	2.000	2.952	4.885	6.078	2.462	0.085	3.212	5.280	2.760	3.780	2.895	3.856	3.529	4.664	4.049	5.251	2.347	3.876	3.597	5.203	3.279	4.664	1.847	4.357	7.867	3.251	4.857	5.414	5.991	1.210	3.674	2.866	2.135	2.500	2.491	1.450	2.654	5.232	4.385	5.097	2.904	5.578	2.654	2.856	0.029	2.029	5.463	5.020	3.597	4.270
PC-O(38:5)	0.092	0.104	0.156	0.102	0.212	0.251	0.157	0.206	0.174	0.185	0.133	0.169	0.138	0.154	0.167	0.179	63.53	0.191	0.167	0.229	33.46	0.359	0.166	67.873	0.120	0.184	0.186	58.04	0.439	0.251	41.78	0.060	0.109	0.131	0.092	34.22	0.112	0.049	0.162	0.208	71.47	44.808	61.73	0.166	0.153	0.068	0.068	0.127	0.100	0.116	0.083	0.079
PC-O(38:6)	173.9	196.4	198.4	145.3	199.7	235.6	227.6	223.6	219.0	151.3	167.9	183.1	134.0	160.6	144.7	153.3	154.6	203.7	153.9	193.1	169.9	224.3	172.5	240.9	136.7	210.4	230.3	264.8	249.5	250.8	191.1	65.56	178.5	141.3	113.5	99.54	102.9	73.66	130.1	159.3	187.8	199.7	147.3	146.6	173.2	120.1	204.4	146.6	221.0	157.3	177.2	189.1
PC-O(40:3)	4.310	4.190	4.070	3.280	6.260	9.120	5.350	12.80	4.430	4.390	2.570	5.180	2.850	2.310	2.820	5.110	3.460	2.520	2.820	3.670	3.630	5.020	3.740	5.870	2.570	3.160	5.070	4.390	8.110	4.360	5.750	1.660	3.190	3.370	3.460	4.070	2.530	1.860	3.390	4.010	5.240	6.390	3.000	3.760	3.660	2.040	4.860	3.630	3.330	3.010	2.030	3.900
PC-O(40:6)	0.155	0.234	NF	0.172	0.272	0.254	0.306	0.396	0.292	0.123	0.141	0.155	0.059	0.197	0.208	0.084	0.120	0.171	45.11	0.127	0.171	0.096	NF	0.257	0.099	0.145	0.240	70.85	0.213	0.262	0.234	0.072	NF	0.038	NF	NF	NF	NF	37.29	NF	48.25	0.084	45.80	0.048	29.36	0.117	0.108	NF	0.166	0.125	0.231	0.062
PC-O(42:6)	3.102	2.809	4.033	2.482	3.907	4.578	4.225	4.745	3.471	2.297	2.616	3.194	2.297	2.624	3.010	2.733	3.102	3.018	1.853	2.658	2.649	3.370	2.817	4.184	2.021	2.892	3.890	3.454	3.865	3.504	3.949	1.333	3.177	2.591	1.803	2.238	1.744	1.383	2.188	3.152	2.926	3.664	2.213	2.004	2.331	1.534	3.186	2.574	2.767	2.037	2.725	3.018
Cer(34:1)	0.212	0.069	0.076	0.077	0.113	0.199	0.041	0.296	0.024	0.017	0.081	0.068	0.017	0.019	0.145	0.137	0.022	0.124	0.151	0.013	0.025	0.065	0.043	0.168	0.019	0.194	0.022	0.237	0.392	0.034	0.032	0.005	0.018	0.031	0.025	0.018	0.013	0.014	0.013	0.201	0.213	0.618	0.078	0.014	0.189	0.012	0.016	0.018	0.119	0.010	0.014	0.026
Cer(42:1)	2.630	2.875	1.901	2.623	8.016	0.053	4.836	0.058	2.252	1.557	1.802	2.610	2.213	1.272	1.404	3.750	1.650	1.464	0.729	1.245	1.451	2.385	1.570	3.710	0.967	2.484	2.776	4.876	2.849	6.691	2.544	0.327	1.623	1.544	1.080	0.788	1.007	0.460	1.351	4.081	6.326	7.817	1.431	1.921	2.670	0.346	0.518	0.584	0.874	0.655	0.788	1.365
Cer(42:2)	23.70	26.73	16.75	25.74	37.70	39.29	28.55	49.54	20.01	18.46	17.47	22.71	19.34	15.38	13.06	29.10	14.71	12.23	12.46	11.24	12.46	18.24	13.56	23.97	9.369	21.49	23.48	36.26	21.22	33.51	21.93	4.11	14.38	12.95	11.35	7.716	9.865	4.982	12.84	26.12	36.54	47.40	14.38	14.33	18.52	4.166	5.787	7.495	7.991	6.503	7.660	10.86
Cer(43:1)	0.017	0.018	NF	0.021	NF	NF	0.379	NF	NF	0.286	NF	0.303	0.303	0.199	0.210	0.041	NF	NF	0.020	0.321	0.265	0.026	0.211	0.334	0.105	0.318	NF	NF	0.030	0.336	0.026	0.108	NF	0.175	0.577	0.124	0.185	0.128	0.179	0.306	0.036	0.384	NF	0.205	0.026	0.263	NF	NF	NF	NF	NF	NF
SM(30:1)	0.970	0.627	0.586	0.578	NF	1.080	NF	0.399	0.571	0.743	0.455	0.768	0.172	0.577	0.662	0.597	0.726	0.201	0.570	NF	0.597	0.550	0.736	1.100	NF	0.822	0.910	0.916	0.902	0.584	NF	NF	NF	1.180	NF	0.565	0.479	NF	1.050	1.250	1.460	2.220	1.170	0.999	1.480	NF	0.921	0.639	0.462	0.405	0.588	0.819
SM(31:0)	0.045	NF	0.041	0.054	NF	NF	NF	NF	0.046	0.035	0.046	0.041	0.025	0.048	0.028	0.054	0.101	0.033	0.033	0.056	0.033	0.046	0.038	0.045	0.044	0.040	0.036	0.040	0.057	0.031	0.041	0.036	0.033	0.045	0.050	0.046	0.052	0.042	0.652	1.210	1.027	1.791	2.382	1.791	3.001	0.535	1.252	0.853	1.205	1.153	0.130	1.876
SM(31:1)	0.427	0.498	0.449	0.364	0.645	0.631	0.689	0.625	0.388	0.407	0.442	0.411	0.346	0.411	0.372	1.040	0.570	0.381	0.373	0.410	0.414	0.375	0.412	0.518	0.367	0.355	0.524	0.574	0.500	0.557	0.514	0.149	0.478	0.306	0.293	0.304	0.309	0.183	1.070	0.570	0.992	0.744	0.493	0.387	0.627	0.323	1.011	0.741	1.056	0.973	0.975	1.325

SM(32:1)	38.63	40.86	42.72	35.93	53.89	56.50	51.19	53.43	32.86	36.12	32.30	40.58	33.70	31.28	31.28	34.25	28.20	31.09	29.04	32.67	30.53	37.05	37.98	48.49	26.90	33.79	45.61	49.61	39.00	47.28	44.03	12.47	33.14	27.37	23.08	28.95	25.04	15.82	30.72	36.95	61.81	87.50	30.44	27.55	35.37	21.87	30.16	34.07	30.25	26.53	26.53	36.21
SM(33:1)	0.271	0.223	0.229	0.273	0.594	0.386	0.490	0.371	0.174	0.172	0.216	0.165	0.135	0.204	0.226	0.276	0.405	0.221	0.114	0.222	0.195	0.160	0.152	0.677	0.146	0.198	0.514	0.666	0.202	0.561	0.256	0.135	0.157	0.129	0.190	0.182	0.191	0.150	2.745	8.800	12.54	12.87	11.28	0.726	19.36	2.684	9.295	8.855	12.21	9.185	2.085	14.58
SM(33:2)	0.941	0.268	0.217	0.179	0.321	0.259	0.296	0.262	0.209	0.186	0.277	0.164	0.176	0.176	0.178	0.226	0.526	0.169	0.182	0.174	0.201	0.183	0.174	0.194	0.163	0.158	0.236	0.231	0.321	0.249	0.223	0.084	0.261	0.156	0.125	0.124	0.116	0.099	3.036	3.445	8.346	5.196	10.23	9.491	1.391	2.046	7.617	6.136	8.836	7.511	1.751	11.45
SM(34:1)	17.63	8.254	6.758	11.82	9.325	23.08	9.694	23.54	13.66	7.239	12.09	11.91	6.140	5.706	18.65	17.63	4.838	11.54	10.62	5.974	11.36	10.06	5.780	15.142	5.318	17.54	8.254	22.25	20.50	9.039	7.820	4.663	8.836	9.602	9.787	7.322	4.487	6.518	4.847	19.48	7.811	30.28	11.63	5.300	12.09	8.993	12.19	12.28	15.60	5.484	5.392	5.521
SM(34:2)	0.176	0.060	0.058	0.395	0.407	0.082	0.122	0.340	0.199	0.061	0.228	0.048	0.065	0.081	0.150	0.079	0.080	0.262	0.053	0.452	0.118	0.051	0.055	0.083	0.037	0.093	0.177	0.387	0.107	0.061	0.066	0.025	0.061	0.049	0.046	0.052	0.039	0.066	0.047	0.175	0.213	0.691	0.309	0.175	0.327	0.068	0.259	0.063	0.177	0.178	0.042	0.351
SM(35:1)	0.045	0.058	0.061	0.048	0.082	0.076	0.068	0.072	0.069	0.074	0.064	0.063	0.054	0.054	0.052	0.084	0.109	0.067	0.079	0.059	0.065	0.090	0.075	0.072	0.055	0.056	0.066	0.075	0.093	0.067	0.067	0.035	0.086	0.064	0.042	0.042	0.042	0.034	0.307	2.590	2.385	3.382	1.817	1.780	2.385	0.040	1.416	1.724	2.162	0.892	0.093	1.547
SM(36:1)	4.769	4.482	4.948	3.394	4.409	5.284	2.716	5.595	3.084	3.182	3.059	3.386	3.673	3.059	2.184	3.141	3.885	2.405	2.593	2.527	3.035	3.803	4.180	3.681	2.626	4.613	5.758	4.711	4.989	4.261	3.877	1.145	2.994	1.391	2.290	1.235	2.388	1.538	3.247	4.777	5.562	3.942	4.171	3.231	3.542	2.487	2.936	1.775	3.951	2.601	3.239	3.182
SM(36:2)	1.800	1.379	1.019	1.484	2.186	2.055	1.449	1.730	1.027	1.203	0.983	1.264	1.352	0.771	1.019	1.502	0.887	1.027	0.703	1.300	1.019	1.264	1.124	1.185	0.717	1.247	1.686	1.739	1.370	1.545	1.502	0.550	1.106	0.522	0.729	0.606	0.887	0.502	0.589	1.528	1.826	1.563	0.689	1.124	1.238	0.544	0.223	0.797	0.922	0.446	0.767	1.624
SM(37:1)	0.723	0.157	0.454	2.804	4.015	0.197	0.984	0.677	2.905	0.119	0.204	0.224	0.183	0.061	0.144	NF	3.757	0.150	0.212	0.126	0.658	0.354	0.109	0.261	NF	0.080	0.552	0.430	4.385	2.366	6.202	0.057	5.776	3.432	NF	NF	1.045	0.042	3.140	8.905	16.49	10.037	3.387	3.903	7.178	1.525	3.578	3.510	4.497	2.692	4.105	4.710
SM(38:2)	73.49	60.68	53.03	54.37	89.31	77.01	63.57	77.21	54.68	57.26	46.20	44.14	54.27	36.07	44.96	72.05	41.14	53.13	39.90	57.06	40.52	51.48	50.86	49.82	29.36	53.54	56.54	59.74	45.69	66.15	50.96	17.88	46.10	29.25	35.25	24.81	32.25	17.37	27.08	44.34	45.79	62.02	30.39	35.97	27.70	25.94	41.35	29.98	50.75	33.70	38.87	26.05
SM(39:2)	0.580	1.898	0.450	2.864	2.171	2.047	0.442	2.319	1.048	1.577	0.656	0.281	0.908	0.284	1.667	2.377	0.301	2.105	1.436	1.552	0.409	1.090	0.834	0.266	0.182	0.941	1.651	2.171	0.941	1.453	2.220	0.280	1.180	0.257	1.106	0.473	0.803	0.666	1.684	0.095	1.486	0.184	2.031	1.337	0.304	0.420	0.076	0.688	0.396	0.433	0.125	0.121
SM(40:1)	7.422	4.915	4.012	602.8	4.614	NF	3.109	3.009	3.410	NF	2.708	2.708	2.207	NF	NF	NF	NF	NF	NF	NF	NF	NF	NF	389.2	NF	3.711	5.015	NF	16.25	7.222	2.909	1.003	6.419	1.906	1.505	2.808	NF	NF	1.906	3.210	NF	NF	NF	5.015	4.814	NF	NF	NF	NF	NF	NF	NF
SM(40:2)	34.96	42.48	36.35	35.65	52.55	55.33	54.52	60.65	39.47	46.30	41.21	35.42	37.16	13.43	34.49	44.57	38.66	36.93	31.37	42.83	34.15	40.51	38.55	47.69	31.25	52.09	51.16	53.48	41.09	57.18	52.09	10.68	34.61	25.58	28.13	24.54	25.23	13.77	30.21	45.26	46.65	51.16	35.65	40.75	33.68	26.62	44.33	31.60	42.25	36.00	37.04	26.51
SM(41:1)	3.570	0.092	4.340	0.023	4.481	0.039	2.376	3.722	5.881	0.013	0.091	0.052	3.103	2.203	0.025	5.620	3.038	0.733	0.154	0.028	0.028	4.025	3.635	0.023	0.013	1.345	0.435	4.166	0.072	3.125	1.920	NF	0.044	2.224	1.139	0.022	3.505	0.009	2.365	5.251	8.647	5.382	5.533	0.039	3.743	0.018	0.029	0.021	0.035	2.181	NF	0.070
SM(41:2)	14.63	14.96	11.00	10.47	18.87	21.11	18.98	18.87	12.28	14.07	13.29	10.17	11.17	11.84	10.53	12.17	9.693	12.06	8.386	12.95	11.50	13.18	10.37	14.85	9.023	13.74	13.40	15.63	13.74	15.86	12.51	3.674	13.29	7.493	9.056	6.689	7.772	5.036	9.190	10.53	18.98	15.19	8.029	7.929	9.112	7.638	10.73	9.235	12.73	8.219	10.81	9.235
SM(42:2)	407.9	467.2	407.9	352.8	530.8	530.8	491.6	603.9	406.8	401.5	363.4	382.5	368.7	328.4	310.4	383.5	331.6	348.6	297.7	369.7	331.6	416.4	344.3	459.8	282.9	415.3	502.2	535.0	412.1	544.5	470.4	138.8	371.9	273.3	257.4	220.4	261.7	151.5	294.5	464.0	526.5	542.4	321.0	352.8	360.2	222.5	356.0	276.5	357.0	306.2	324.2	285.0
SM(43:2)	5.181	5.484	3.610	4.764	7.890	3.757	7.743	6.695	3.814	3.552	3.692	2.865	4.878	2.726	2.783	3.970	2.848	2.406	3.094	6.057	3.315	3.552	3.266	5.836	2.791	3.258	3.724	4.084	5.607	7.383	3.913	1.588	3.520	2.079	2.398	2.529	2.218	1.588	3.462	4.003	3.724	4.117	2.447	2.750	3.176	2.014	3.282	2.447	2.783	2.677	2.693	3.053
SM(44:1)	7.990	0.039	0.035	6.835	16.28	8.786	0.040	5.057	6.246	2.021	0.034	4.907	9.514	6.777	4.884	8.590	0.048	3.822	10.17	0.048	4.491	0.117	0.042	0.073	0.458	6.685	0.207	6.500	5.923	9.410	8.475	0.708	0.037	0.032	4.537	2.032	4.156	1.478	8.001	0.066	21.59	17.549	4.445	5.415	8.913	0.020	0.027	0.024	0.034	0.021	0.025	4.180
SM(44:2)	2.638	3.670	2.922	2.808	3.851	4.398	3.459	4.749	2.633	2.958	2.416	2.421	2.251	2.261	2.122	3.293	2.018	2.344	2.720	2.628	2.478	3.252	2.555	3.748	2.044	2.695	3.526	4.037	2.927	4.424	3.108	0.867	2.111	2.204	1.776	1.605	1.889	0.940	2.411	4.811	5.157	6.143	2.849	2.628	3.139	1.833	2.349	0.934	2.375	1.838	1.946	2.658
H1	4027	4571	5934	3481	4540	4642	4904	4407	4388	3693	4734	4338	4095	5916	4987	3661	4574	4588	6014	4429	5249	6814	8513	5939	3574	7473	4049	4876	8503	4044	5555	1990	4310	5498	4706	4574	3490	4151	3116	3327	3530	4583	4926	4534	4645	3320	4231	3642	4615	3170	3887	4585

Supplementary Table S2b

Curated targeted metabolomics dataset showing the median concentrations (µM) of n=8 replicates per treatment group, and metabolites with significant differences (control vs. PRV-1 infected) as determined by t-test ($p<0.05$) and Volcano plot (adjusted $p<0.1$).

	Median concentrations (µM) (n=8 replicates)								Based on individual concentrations			
									t-test p-values		Volcano plot, adjusted p	
**Metabolite**	**C0**	**C2**	**P2**	**C5**	**P5**	**C8**	**W8**	**% sum**	**C5/P5**	**C8/P8**	**C5/P5**	**C8/P8**
AC(0:0)	4.04	4.63	3.82	4.09	3.08	6.10	8.76		0.0272	0.0042		
AC(2:0)	1.42	1.88	1.46	1.96	1.04	2.23	2.69		0.0423			
AC(3:0)	0.14	0.21	0.11	0.17	0.08	0.28	0.32					
AC(3:0-OH)	0.13	0.10	0.10	0.12	0.07	0.12	0.08		0.0030	0.0405		
AC(4:0)	0.08	0.07	0.07	0.07	0.05	0.07	0.08		0.0393			
AC(5:0-OH)	0.02	0.02	0.02	0.02	0.02	0.02	0.02		0.0234			
AC(6:0)	0.03	0.02	0.02	0.03	0.02	0.03	0.02		0.0179			
AC(6:0-DC)	0.05	0.04	0.03	0.04	0.02	0.04	0.03		0.0064	0.0158		
AC(6:0-OH)	0.02	0.02	0.02	0.02	0.02	0.02	0.02					
AC(7:0)	0.03	0.02	0.02	0.02	0.02	0.02	0.02		0.0005	0.0019		
AC(8:0)	0.05	0.04	0.05	0.05	0.05	0.04	0.04					
AC(8:1)	0.01	0.01	0.01	0.01	0.01	0.01	0.01		0.0003			
AC(8:1-OH)	0.17	0.18	0.17	0.15	0.12	0.19	0.16					
AC(9:0)	0.02	0.02	0.01	0.02	0.01	0.01	0.01		0.0041	0.0218		
AC(10:0)	0.02	0.02	0.02	0.02	0.01	0.01	0.02		0.0447			
AC(10:1)	0.01	0.00	0.00	0.01	0.00	0.00	0.00		0.0004			
AC(12:0)	0.07	0.06	0.05	0.06	0.04	0.05	0.05		0.0100			
AC(12:0-DC)	0.03	0.03	0.03	0.03	0.02	0.05	0.04					
AC(12:1)	0.02	0.03	0.02	0.02	0.01	0.01	0.01		0.0073		0.0131	
AC(13:0)	0.02	0.03	0.02	0.02	0.02	0.02	0.02		0.0390			
AC(14:0)	0.11	0.09	0.08	0.10	0.05	0.09	0.08		0.0003		0.0058	
AC(14:0-OH)	0.01	0.01	0.01	0.01	0.01	0.01	0.01		0.0052			
AC(14:1)	0.11	0.13	0.10	0.14	0.09	0.06	0.10		0.0057			
AC(14:2)	0.01	0.01	0.01	0.01	0.00	0.01	0.00		0.0013		0.0062	
AC(14:2-OH)	0.01	0.01	0.01	0.01	0.01	0.01	0.01					
AC(15:0)	0.03	0.02	0.02	0.02	0.01	0.02	0.01		0.0051		0.0101	
AC(16:0)	0.41	0.28	0.26	0.38	0.21	0.19	0.27		0.0005			
AC(16:0-OH)	0.04	0.03	0.02	0.04	0.01	0.00	0.01		0.0022		0.0067	
AC(16:1-OH)	0.03	0.03	0.03	0.04	0.03	0.02	0.02					
AC(16:2)	0.01	0.00	0.00	0.00	0.00	0.01	0.00		0.0003	0.0184		
AC(17:0)	0.01	0.01	0.01	0.01	0.00	0.01	0.01		0.0003		0.0058	
AC(18:0)	0.08	0.05	0.04	0.07	0.04	0.04	0.10		0.0290	0.0027		
AC(18:1)	0.99	0.73	0.67	0.95	0.53	0.95	0.90		0.0018			
AC(18:1-OH)	0.17	0.11	0.09	0.16	0.05	0.03	0.05		0.0073		0.0131	
AC(18:2)	0.20	0.16	0.16	0.20	0.11	0.29	0.18	0.02	0.0017			
Ala	781	722	982	799	729	686	961			0.0268		
Arg	327	299	346	312	313	256	302			0.0327		
Asn	23.4	9.25	8.79	12.7	6.10	10.33	8.34					
Asp	107	58.6	47.4	89.0	37.1	114	221		0.0171	0.0000	0.0266	
Cit	18.9	14.5	13.8	16.6	12.5	27.9	13.5			0.0000		0.0003
Gln	834	840	777	904	680	687	482			0.0127		
Glu	196	225	131	252	93	238	266		0.0059		0.0111	
Gly	1118	920	1120	1245	757	1186	1302		0.0168	0.0341		
His	61.2	49.6	50.9	73.8	45.2	80.8	34.9			0.0007		0.0032
Ile	215	190	211	182	168	434	235			0.0010		
Lys	641	581	650	706	552	387	590			0.0016		
Met	117	85.5	92.1	99.2	67.1	302.2	80.7		0.0242	0.0000		0.0000
Orn	46.4	35.7	39.2	48.3	44.0	38.8	43.7					
Phe	160	137	131	131	101	261	134		0.0330	0.0001		
Pro	168	113	441	184	73	379	108			0.0001		0.0006
Ser	296	226	266	279	193	335	160		0.0317	0.0000		
Thr	440	437	410	536	428	556	408			0.0292		
Trp	41.3	32.2	33.3	35.2	27.1	43.3	31.1					
Tyr	95.4	91.9	78.1	86.7	62.9	235	109			0.0008		
Val	499	415	476	436	409	799	538			0.0058		
xLeu	533	481	523	476	403	1297	621	13.82		0.0012		0.0049
ADMA	1.83	1.42	1.54	1.65	1.34	1.24	2.33			0.0000		
Creatinine	9.20	3.51	5.54	4.33	4.75	64.7	3.85			0.0000		0.0000
Met-SO	2.70	1.70	2.00	2.53	1.56	34.8	1.19			0.0000		0.0000
Putrescine	25.5	26.5	21.2	33.2	17.2	42.1	46.5		0.0051	0.0437		
Sarcosine	4.16	3.58	3.80	4.56	3.08	4.70	5.27					
SDMA	1.20	1.00	1.48	1.24	1.37	0.63	1.61			0.0002		0.0014
Spermidine	1.20	1.52	1.03	2.09	0.77	2.03	4.10		0.0004	0.0066	0.0058	0.0010
Spermine	0.34	0.18	0.05	0.39	0.05	0.05	0.78			0.0001		
t4-OH-Pro	442	388	446	463	393	357	438			0.0040		
Taurine	1375	1345	1217	1728	776	2210	2693	3.83	0.0034	0.0019		
CE(16:0)	262	259	359	278	248	256	229			0.0223		
CE(16:1)	715	516	559	625	340	475	525		0.0029			
CE(17:1)	58.1	43.8	46.6	58.4	33.1	40.3	48.2		0.0091			
CE(17:2)	26.4	19.3	22.1	21.8	15.5	15.2	23.9		0.0133	0.0019		
CE(18:2)	1118	851	839	1137	538	765	833		0.0019			
CE(18:3)	489	362	376	460	228	338	363		0.0015			
CE(19:2)	22.0	17.1	16.9	23.6	10.9	15.8	18.4		0.0058		0.0111	
CE(19:3)	10.1	7.99	8.61	10.4	5.85	6.73	9.06		0.0039			
CE(20:4)	577	782	454	387	80	770	185		0.0161	0.0254	0.0255	0.0591
CE(20:5)	4298	3526	3660	4660	2383	3484	3485		0.0048			
CE(22:6)	19967	15180	14851	19258	9697	13552	14758	56.64	0.0032			
DG(32:1)	5.13	3.86	4.45	3.85	1.33	10.0	1.89		0.0031	0.0000	0.0076	0.0000
DG(32:2)	2.49	1.99	2.58	2.06	0.80	4.16	1.21		0.0088	0.0021	0.0151	0.0071
DG(34:1)	16.4	11.0	10.8	10.5	5.24	22.9	4.89		0.0033	0.0000	0.0077	0.0001
DG(34:3)	4.87	3.69	4.25	3.07	0.62	9.74	1.43		0.0031	0.0004	0.0076	0.0019
DG(36:2)	21.6	16.8	15.8	16.9	8.59	21.4	6.56			0.0016		0.0059
DG(36:3)	9.16	7.01	7.98	6.97	3.24	11.9	4.58		0.0019	0.0001	0.0062	0.0010
DG(36:4)	4.37	3.00	3.24	3.24	1.38	5.13	2.18		0.0018	0.0009	0.0062	0.0039
DG(38:0)	4.08	2.00	0.56	4.58	0.55	7.24	3.92					

DG(38:5)	4.93	2.42	2.02	5.56	1.94	5.08	1.85		0.0051		0.0101	
DG(39:0)	242	194	213	228	141	189	166		0.0071			
DG(42:0)	3.05	2.71	2.20	2.84	1.29	2.16	2.09		0.0015		0.0062	
DG(42:1)	1.16	1.14	1.03	1.20	0.62	0.62	1.01		0.0180			
DG(42:2)	8.71	6.67	6.48	7.77	4.27	6.87	6.35		0.0019			
DG(44:3)	1.58	1.26	1.07	1.46	0.63	1.23	0.98		0.0027		0.0074	
DG-O(32:2)	2.28	1.52	1.49	2.10	0.69	2.93	0.91		0.0037	0.0001	0.0081	0.0010
DG-O(34:1)	19.3	15.3	15.0	15.7	10.3	18.6	10.4		0.0121	0.0012		0.0046
DG-O(36:4)	6.85	5.31	4.52	7.37	3.52	6.97	3.51	0.74	0.0005	0.0007	0.0058	0.0031
TG(46:2)	0.05	0.04	0.04	0.04	0.02	0.05	0.02		0.0258	0.0177		0.0444
TG(48:1)	35.1	24.2	22.8	28.0	13.3	41.6	15.4		0.0061	0.0002		0.0014
TG(48:2)	0.04	0.06	0.10	0.07	0.06	33.73	0.06			0.0007		0.0032
TG(49:2)	0.06	3.58	0.07	5.16	0.05	5.75	0.04			0.0000		0.0001
TG(50:1)	0.27	0.24	0.36	0.32	0.22	1.33	0.19				0.0746	
TG(50:2)	163	139	114	146	48.2	153	76		0.0230		0.0347	
TG(50:3)	74.0	58.3	58.4	63.0	26.4	90.8	35.8		0.0030	0.0001	0.0076	0.0007
TG(50:4)	11.1	9.08	11.7	10.6	10.9	37.7	11.9			0.0037		0.0118
TG(51:2)	0.23	8.77	15.9	49.6	8.30	56.4	0.11			0.0013		0.0049
TG(51:3)	38.0	27.7	25.4	32.0	15.2	30.4	16.7		0.0037	0.0011	0.0082	0.0045
TG(51:4)	0.06	0.05	0.06	0.05	0.03	16.7	0.03			0.0000		0.0002
TG(52:2)	376	267	244	283	125	307	199		0.0131	0.0059	0.0213	0.0169
TG(52:3)	275	199	202	231	91	253	142		0.0019	0.0010	0.0062	0.0042
TG(52:4)	151	114	118	133	51.3	167	79.0		0.0029	0.0002	0.0076	0.0010
TG(52:5)	82.6	55.3	63.6	73.5	28.9	88.2	37.9		0.0047	0.0001	0.0097	0.0007
TG(52:6)	48.0	35.4	32.3	47.9	18.0	47.5	16.8		0.0110	0.0001	0.0184	0.0008
TG(52:7)	16.1	13.2	14.4	16.6	8.23	20.4	9.8		0.0013	0.0000		0.0005
TG(53:3)	12.3	10.8	6.46	13.8	5.40	0.17	6.36					
TG(53:4)	29.1	21.1	21.0	26.5	10.7	25.9	12.0		0.0059	0.0009	0.0111	0.0038
TG(53:5)	22.9	17.1	16.9	25.8	10.1	26.3	12.1		0.0021	0.0001	0.0065	0.0007
TG(53:6)	20.1	14.3	13.6	18.6	7.89	18.7	8.5		0.0015	0.0001	0.0062	0.0007
TG(54:2)	2.79	2.96	4.81	4.71	2.84	6.79	2.64		0.0421		0.0571	
TG(54:3)	445	345	329	386	175	409	275		0.0024	0.0075	0.0070	
TG(54:4)	299	229	244	275	106	310	201		0.0022	0.0023	0.0065	
TG(54:5)	195	154	172	198	76	217	128		0.0015	0.0005	0.0062	
TG(54:6)	191	148	146	194	78	194	103		0.0020	0.0003	0.0062	0.0019
TG(54:7)	113	91.9	82.5	104	50.8	84.1	63.4		0.0073	0.0216	0.0131	
TG(55:6)	37.4	23.1	24.7	34.8	16.0	20.1	12.4		0.0107			
TG(55:7)	265	196	151	238	83.2	189	57.5			0.0000		0.0002
TG(56:9)	3.88	3.08	2.90	4.18	1.68	2.87	1.59	5,97	0.0028	0.0049	0.0075	
LPC(14:0)	6.74	4.49	4.61	5.49	2.19	6.34	4.49		0.0003	0.0000	0.0058	
LPC(15:0)	1.36	0.88	1.03	1.18	0.41	1.54	0.89		0.0015	0.0000	0.0062	
LPC(16:0)	68.0	49.9	43.8	62.9	23.6	57.6	38.8		0.0004	0.0002	0.0058	
LPC(16:1)	7.94	5.05	5.19	6.18	2.23	7.23	4.25		0.0004	0.0001	0.0058	
LPC(17:0)	1.50	0.80	0.89	1.19	0.56	1.44	0.86		0.0002	0.0000		
LPC(17:1)	2.83	1.79	1.85	2.39	0.91	3.10	1.76		0.0007	0.0000	0.0058	
LPC(18:0)	3.58	2.48	2.23	3.60	1.60	4.57	2.16		0.0034	0.0010	0.0078	0.0042
LPC(18:1)	21.1	13.4	14.1	17.1	6.6	26.6	13.9		0.0004	0.0000	0.0058	0.0001
LPC(18:2)	4.06	3.05	2.38	3.62	1.35	4.66	2.11		0.0004	0.0000	0.0058	0.0003
LPC(20:0)	0.51	0.33	0.34	0.44	0.18	0.43	0.20		0.0007	0.0004	0.0058	0.0020
LPC(20:1)	3.91	2.40	2.49	2.98	1.36	5.69	1.77		0.0009	0.0000	0.0058	0.0000
LPC(20:2)	1.17	0.81	0.71	1.00	0.45	1.78	0.72		0.0019	0.0000	0.0062	0.0001
LPC(20:4)	6.75	6.87	6.58	10.2	2.35	14.7	3.79		0.0001	0.0000	0.0058	0.0000
LPC(22:6)	92.2	58.6	58.0	77.1	28.8	135	54.9		0.0006	0.0000	0.0058	0.0000
LPC(24:1)	7.81	5.45	5.80	7.70	3.69	6.37	3.96		0.0010	0.0022		
LPC-O(16:1)	2.08	1.04	0.89	1.41	0.42	1.23	1.03		0.0001		0.0058	
LPC-O(18:1)	3.77	2.53	2.35	2.84	1.33	2.86	3.22	0.48	0.0010		0.0058	
PC(24:0)	0.03	0.02	0.02	0.03	0.02	0.02	0.02					
PC(28:1)	0.43	0.36	0.31	0.45	0.17	0.29	0.26		0.0347		0.0483	
PC(29:0)	0.77	0.62	0.70	0.92	1.24	0.69	0.65					
PC(29:2)	0.25	0.21	0.20	0.26	0.15	0.25	0.17		0.0011	0.0121		
PC(30:0)	10.0	7.39	7.70	7.77	5.27	8.69	7.93		0.0010			
PC(30:1)	17.9	14.6	14.6	16.8	10.8	15.4	14.5		0.0016			
PC(30:2)	1.59	1.24	1.32	1.28	0.75	1.12	1.05		0.0032			
PC(31:1)	7.70	6.11	6.27	7.00	4.72	6.83	6.50		0.0014			
PC(32:0)	6.42	9.58	8.41	5.80	1.09	12.50	0.15			0.0000		0.0000
PC(32:1)	81.5	70.1	69.7	82.6	46.8	78.9	67.6		0.0024			
PC(32:2)	16.6	14.8	13.2	17.9	8.86	14.5	11.6		0.0018	0.0313		
PC(32:3)	1.29	1.09	1.32	1.54	0.60	1.58	0.70		0.0000	0.0000	0.0030	0.0000
PC(32:4)	7.68	5.53	8.23	9.30	3.16	11.3	3.21		0.0003	0.0288	0.0058	0.0637
PC(32:5)	0.41	0.34	0.31	0.39	0.18	0.36	0.18		0.0038	0.0003	0.0083	0.0017
PC(32:6)	0.28	0.19	0.19	0.18	0.09	0.24	0.17				0.0682	
PC(33:0)	5.89	2.44	4.61	5.36	2.87	6.59	1.56			0.0331		0.0692
PC(33:1)	24.5	19.9	19.7	22.9	13.6	22.9	19.2		0.0019			
PC(33:3)	1.91	1.43	1.64	1.88	0.89	1.55	1.00		0.0019	0.0019	0.0062	
PC(33:4)	4.13	3.06	3.16	3.29	2.05	3.26	3.64		0.0028			
PC(33:5)	0.30	0.22	0.28	0.43	0.15	0.25	0.16		0.0005		0.0058	
PC(34:1)	591	548	511	642	357	586	492		0.0046			
PC(34:2)	565	514	379	660	299	431	302		0.0060		0.0113	
PC(34:3)	116	104	84.3	120	56.3	94.8	62.2		0.0031	0.0183	0.0076	
PC(34:4)	66.9	57.2	52.0	65.0	35.1	57.0	45.8		0.0028		0.0076	
PC(34:5)	57.8	45.6	53.6	58.3	30.8	57.6	38.0		0.0016	0.0007		
PC(35:0)	2.56	1.34	5.79	8.25	3.47	8.01	3.39					
PC(35:1)	23.8	18.5	21.3	24.7	13.3	21.5	20.5		0.0008			
PC(35:2)	32.0	25.1	27.2	29.7	17.8	28.7	24.9		0.0025			
PC(35:3)	4.71	3.69	3.45	4.85	2.26	4.09	3.05		0.0017	0.0329		
PC(35:4)	7.26	5.85	5.85	7.28	3.68	6.98	6.02		0.0031		0.0076	
PC(35:5)	8.08	6.46	7.83	7.89	4.31	8.04	5.57		0.0016	0.0003		
PC(36:0)	0.16	0.16	0.16	0.16	0.16	0.16	4.19					
PC(36:2)	277	252	220	291	157	269	206		0.0055			
PC(36:3)	301	265	212	325	158	255	178		0.0062	0.0496		
PC(36:4)	344	283	276	379	183	306	265		0.0159			
PC(36:5)	128	114	120	141	77.0	123	82.1		0.0027	0.0037		
PC(36:6)	2.22	1.73	1.95	2.15	1.17	2.04	1.60		0.0017	0.0018		
PC(37:1)	1.99	1.43	1.79	2.00	1.10	1.63	1.56		0.0010			
PC(37:2)	18.3	15.0	14.9	17.9	10.1	14.7	13.9		0.0008			

PC(37:3)	15.4	10.7	12.1	15.3	8.0	12.5	10.6		0.0015			
PC(37:4)	30.8	19.8	19.2	24.5	13.4	20.2	19.0		0.0015			
PC(37:5)	23.6	17.9	18.3	22.7	12.0	20.0	17.5		0.0047			
PC(37:6)	60.9	47.3	54.4	60.9	33.3	60.9	44.4		0.0016	0.0006		
PC(37:7)	5.93	4.73	4.90	6.01	2.91	5.72	3.64		0.0017	0.0003		
PC(38:0)	3.94	5.09	5.44	5.52	5.19	5.01	6.40			0.0244		
PC(38:1)	25.2	21.8	21.7	24.9	17.3	24.6	20.7		0.0279			
PC(38:2)	32.0	24.0	22.6	29.1	18.1	29.7	20.5		0.0084			
PC(38:4)	145	127	111	153	78	116	101		0.0384			
PC(39:4)	9.62	7.26	7.82	9.25	5.12	8.01	7.20		0.0009			
PC(39:5)	10.5	6.64	8.23	9.28	5.74	7.93	7.02		0.0167			
PC(39:7)	5.85	4.59	4.74	6.05	3.40	5.30	4.08		0.0035	0.0140		
PC(40:1)	1.31	1.42	1.30	1.55	0.93	3.45	1.69					
PC(40:2)	11.1	11.9	14.9	17.3	10.3	19.0	11.0		0.0076	0.0013		
PC(40:3)	1.91	8.95	10.7	13.8	10.8	17.7	4.47			0.0366		0.0752
PC(40:4)	27.3	25.4	21.3	28.0	18.3	31.1	20.8		0.0200	0.0039		
PC(40:5)	0.38	0.34	0.36	0.42	0.19	0.48	0.20		0.0008	0.0000	0.0058	0.0004
PC(40:6)	1.31	1.68	1.42	2.69	1.16	2.49	0.86		0.0016	0.0001	0.0062	0.0007
PC(41:5)	18.7	20.2	21.2	19.5	14.2	15.9	20.1					
PC(42:1)	0.03	0.02	0.02	0.03	0.01	2.24	0.26		0.0005	0.0100	0.0058	0.0278
PC(42:2)	5.04	4.53	5.00	5.25	2.95	14.27	5.12		0.0419	0.0089		0.0249
PC(42:4)	1.17	1.11	1.06	1.33	0.84	2.61	1.27		0.0330	0.0271		
PC(43:6)	1.69	1.46	1.55	1.79	1.13	1.76	1.40		0.0080	0.0263		
PC(44:5)	2.40	1.97	2.19	2.87	1.45	3.49	2.17		0.0016	0.0169		
PC(44:7)	8.53	6.84	6.68	8.97	4.60	7.71	5.93		0.0010	0.0154		
PC(44:10)	0.26	98.4	42.6	133	0.17	100	0.16		0.0188	0.0001	0.0287	0.0007
PC(46:2)	0.18	0.13	0.08	0.01	0.03	9.30	4.42					
PC-O(26:0)	0.10	0.14	0.12	0.12	0.07	0.23	0.09		0.0257			
PC-O(26:1)	0.54	0.41	0.40	0.53	0.30	0.44	0.34		0.0033	0.0331		
PC-O(28:1)	2.93	2.25	2.41	2.90	1.52	1.91	1.77		0.0007			
PC-O(30:1)	5.88	4.30	5.31	6.37	2.95	3.39	3.62		0.0002			
PC-O(30:2)	4.34	3.21	3.57	4.30	2.22	2.28	2.25		0.0006			
PC-O(31:1)	0.07	0.07	1.37	1.42	0.07	1.06	0.16		0.0114		0.0189	
PC-O(31:3)	0.14	0.13	0.15	0.13	0.04	0.10	0.11				0.0692	
PC-O(32:1)	0.03	1.73	4.22	4.76	2.05	2.88	2.23				0.0886	
PC-O(32:2)	7.12	5.10	5.22	6.44	3.72	4.90	4.47		0.0008			
PC-O(33:2)	6.42	5.03	4.53	6.22	3.42	5.47	4.44		0.0007	0.0495		
PC-O(33:3)	1.75	1.28	1.37	1.52	0.82	1.05	1.07		0.0008			
PC-O(34:1)	12.7	9.52	11.8	12.9	7.09	9.93	11.5		0.0035			
PC-O(34:2)	28.4	22.8	22.6	25.7	15.1	21.9	20.5		0.0025			
PC-O(34:4)	5.94	4.38	5.67	6.05	2.78	4.82	4.30		0.0040			
PC-O(35:3)	0.03	0.03	0.90	0.03	0.03	0.03	1.65			0.0045		0.0136
PC-O(35:4)	3.12	3.29	3.76	3.16	2.33	2.59	2.99					
PC-O(36:2)	20.1	15.0	17.4	17.8	10.1	16.8	18.8		0.0021			
PC-O(36:3)	8.24	6.49	7.63	7.56	4.14	6.23	7.41		0.0040			
PC-O(36:4)	19.8	15.4	17.3	21.3	11.4	17.2	15.8		0.0012			
PC-O(36:5)	56.2	43.4	52.1	57.7	31.4	47.4	43.8		0.0012			
PC-O(36:6)	10.4	8.46	8.88	10.7	4.87	7.03	7.00		0.0007		0.0058	
PC-O(37:6)	15.6	12.2	12.3	13.3	7.6	9.5	10.7				0.0823	
PC-O(37:7)	94.6	71.7	72.7	87.5	42.0	60.5	55.3		0.0008		0.0058	
PC-O(38:4)	3.29	3.65	3.96	4.86	2.49	4.39	3.60		0.0105		0.0177	
PC-O(38:5)	0.16	0.17	0.29	0.25	0.11	0.21	0.08			0.0208		0.0505
PC-O(38:6)	199	157	183	230	103	159	177		0.0007			
PC-O(40:3)	4.83	3.62	3.65	4.39	3.19	3.76	3.33		0.0251			
PC-O(40:6)	0.24	0.15	0.15	0.23	0.01	29.4	0.12		0.0027		0.0074	
PC-O(42:6)	3.97	2.68	2.92	3.50	1.80	2.33	2.72	7.62	0.0047			
Cer(34:1)	0.10	0.05	0.05	0.03	0.02	0.19	0.02		0.0151	0.0326	0.0243	0.0692
Cer(42:1)	2.63	2.01	1.52	2.78	1.01	2.67	0.65		0.0027	0.0005	0.0075	0.0025
Cer(42:2)	27.6	18.9	13.0	21.9	9.86	18.5	7.50		0.0022	0.0003	0.0065	0.0016
Cer(43:1)	0.01	0.20	0.12	0.03	0.13	0.18	0.01	0.06		0.0141		0.0370
SM(30:1)	0.58	0.59	0.58	0.82	0.34	1.25	0.59			0.0261		0.0601
SM(31:0)	0.02	0.04	0.04	0.04	0.04	1.79	1.15					
SM(31:1)	0.56	0.41	0.41	0.51	0.30	0.63	0.98		0.0048			
SM(32:1)	47.0	33.3	31.9	44.0	25.0	35.4	30.2		0.0031			
SM(33:1)	0.32	0.19	0.21	0.26	0.16	11.3	9.19		0.0154		0.0246	
SM(33:2)	0.27	0.18	0.18	0.23	0.12	5.20	7.51		0.0078			
SM(34:1)	10.8	12.0	10.3	9.0	7.32	11.6	8.99					
SM(34:2)	0.15	0.08	0.08	0.09	0.05	0.21	0.18		0.0312		0.0444	
SM(35:1)	0.06	0.06	0.07	0.07	0.04	2.39	1.42		0.0257			
SM(36:1)	4.63	3.11	3.36	4.61	1.54	3.94	2.94		0.0002	0.0139	0.0058	
SM(36:2)	1.61	1.12	1.08	1.50	0.61	1.24	0.77		0.0009		0.0058	
SM(37:1)	0.70	0.16	0.24	0.55	0.06	7.18	3.58			0.0375		0.0764
SM(38:2)	68.5	50.2	50.3	53.5	29.3	36.0	33.7		0.0036			
SM(39:2)	1.97	0.98	0.96	1.45	0.67	1.34	0.40					
SM(40:1)	4.31	1.10	0.00	3.71	1.50	1.91	0.00			0.0170		0.0430
SM(40:2)	47.5	38.3	38.6	52.1	25.2	40.7	36.0		0.0009		0.0058	
SM(41:1)	2.97	1.15	0.44	1.35	0.12	5.25	0.04			0.0021		0.0071
SM(41:2)	16.9	12.00	11.8	13.7	7.49	9.19	9.24		0.0032			
SM(42:2)	479	376	346	470	257	360	306		0.0011	0.0266		
SM(43:2)	5.33	3.62	3.29	3.91	2.22	3.46	2.69		0.0019	0.0403		
SM(44:1)	5.95	5.58	0.09	6.50	1.48	8.00	0.03			0.0010		0.0042
SM(44:2)	3.56	2.42	2.59	3.11	1.78	3.14	1.95	1.45	0.0014	0.0053		
H1	4556	4363	5594	4876	4310	4534	3887	9.37				
sum	48624							100				

Supplementary Table S3

Univariate analysis of the targeted metabolomics data by volcano plot: 38 metabolites were significantly different at both W5 and W8 (**Figure 3**). Among these were 5 metabolites (highlighted with yellow) that belong to the 20 most relevant metabolites describing the differences in W5 (**Figure S1a**) and W8 (**Figure S1b**). Most-relevant metabolites identified only for W5 or W8 are marked, respectively, in red or blue. They were present among the 38 shared, 50 exclusively in W5 or 32 exclusively in W8 determined metabolites.

W5/W8 38 shared metabolites		W5 50 exclusive metabolites	W8 32 exclusive metabolites
**ID**	**name**	**ID**	**ID**
PC(32:3)	PC(14:0/18:3(6Z,9Z,12Z))	Cer(42:1)	Met-SO
LPC(20:4)	LysoPC(20:4(5Z,8Z,11Z,14Z))	AC(17:0)	PC(32:0)
PC(32:4)	PC(14:0/18:4(6Z,9Z,12Z,15Z))	LPC-O(16:1)	Creatinine
LPC(18:2)	LysoPC(18:2(9Z,12Z))	LPC(16:0)	Met
LPC(20:0)	LysoPC(20:0)	Spermidine	TG(49:2)
LPC(22:6)	LysoPC(22:6(4Z,7Z,10Z,13Z,16Z,19Z))	SM(36:1)	TG(51:4)
LPC(18:1)	LysoPC(18:1(9Z))	LPC(16:1)	TG(55:7)
PC(42:1)	PC(18:0/24:1(15Z))	LPC(14:0)	Cit
PC(40:5)	PC(18:0/22:5(4Z,7Z,10Z,13Z,16Z))	LPC(17:1)	TG(52:7)
DG-O(36:4)	1-(hexadecanoyl)-2-(8--ladderane-octanyl)-sn-glycerol	PC-O(36:6)	Pro
LPC(20:1)	LysoPC(20:1(11Z))	SM(40:2)	Spermine
PC(40:6)	PC(18:0/22:6(4Z,7Z,10Z,13Z,16Z,19Z))	PC-O(37:7)	SDMA
TG(52:3)	TG(16:0/18:1(9Z)/18:2(9Z,12Z))	LPC-O(18:1)	TG(48:1)
TG(54:6)	TG(16:0/18:2(9Z,12Z)/20:4(5Z,8Z,11Z,14Z))	AC(14:0)	His
DG(36:4)	DG(14:0/22:4(7Z,10Z,13Z,16Z)/0:0)	SM(36:2)	TG(48:2)
TG(53:6)	TG(15:0/16:1(9Z)/22:5(4Z,7Z,10Z,13Z,16Z))	TG(54:5)	SM(44:1)
DG(36:3)	DG(14:1(9Z)/22:2(13Z,16Z)/0:0)	PC(33:3)	DG-O(34:1)
LPC(20:2)	LysoPC(20:2(11Z,14Z))	AC(14:2)	TG(51:2)
Cer(42:2)	Ceramide (d18:1/24:1(15Z))	LPC(15:0)	xLeu
TG(53:5)	TG(16:1(9Z)/15:0/22:4(7Z,10Z,13Z,16Z))	DG(42:0)	DG(36:2)
Cer(42:1)	Ceramide (d18:1/24:0)	TG(54:4)	SM(41:1)
DG(34:3)	DG(14:0/20:3(5Z,8Z,11Z)/0:0)	AC(16:0-OH)	TG(50:4)
TG(50:3)	TG(16:0/16:1(9Z)/18:2(9Z,12Z))	TG(54:3)	PC-O(35:3)
TG(52:4)	TG(16:0/16:0/20:4(5Z,8Z,11Z,14Z))	PC-O(40:6)	PC(42:2)
DG(32:1)	DG(14:0/18:1(11Z)/0:0)	DG(44:3)	Cer(43:1)
DG(34:1)	DG(14:0/20:1(11Z)/0:0)	TG(56:9)	SM(40:1)
LPC(18:0)	LysoPC(18:0)	PC(34:4)	TG(46:2)
DG-O(32:2)	DG(P-14:0/18:1(9Z))	PC(34:3)	PC-O(38:5)
TG(51:3)	TG(15:0/16:0/20:3(8Z,11Z,14Z))	PC(35:4)	SM(30:1)
PC(32:5)	PC(14:1(9Z)/18:4(6Z,9Z,12Z,15Z))	DG(38:5)	PC(33:0)
TG(52:5)	TG(16:0/16:1(9Z)/20:4(5Z,8Z,11Z,14Z))	AC(15:0)	PC(40:3)
TG(53:4)	TG(15:0/16:0/22:4(7Z,10Z,13Z,16Z))	Glu	SM(37:1)
DG(32:2)	DG(14:0/18:2(9Z,12Z)/0:0)	CE(19:2)	
TG(52:6)	TG(16:1(9Z)/16:1(9Z)/20:4(5Z,8Z,11Z,14Z))	PC(34:2)	
TG(52:2)	TG(16:0/18:0/18:2(9Z,12Z))	AC(18:1-OH)	
Cer(34:1)	Ceramide (d18:1/16:0)	AC(12:1)	
CE(20:4)	CE(20:4(8Z,11Z,14Z,17Z))	TG(54:7)	
PC(44:10)	PC(22:4(7Z,10Z,13Z,16Z)/22:6(4Z,7Z,10Z,13Z,16Z,19Z))	PC-O(38:4)	
		PC-O(31:1)	
		SM(33:1)	
		Asp	
		TG(50:2)	
		SM(34:2)	
		PC(28:1)	
		TG(54:2)	
		PC(32:6)	
		PC-O(31:3)	
		TG(50:1)	
		PC-O(37:6)	
		PC-O(32:1)	

Supplementary Table S4.

Untargeted metabolomics dataset. Normalised peak areas retrieved from Compound Discoverer (version 3.3).
The dataset was subsequently processed (s. Materials and Methods) for use in the statistical analyses.

Label	C0_3	C0_5	C0_2	C0_1	C0_8	C0_4	C0_6	C0_7	C2_1	C2_3	C2_6	C2_8	C2_5	C2_4	C2_2	C2_7	P2_4	P2_5	P2_3	P2_8	P2_2	P2_6	P2_7	C5_8	C5_2	C5_3	C5_4	C5_7	C5_5	C5_1	P5_5	P5_4	P5_3	P5_7	*P5_8*	P5_2	P5_6	P5_1	C8_3	C8_4	C8_2	C8_6	C8_7	C8_5	*C8_1*	C8_8	P8_4	P8_7	P8_3	P8_6	P8_8	P8_1	P8_5
F1_116.0704_12.9_P	70.32	21.07	52.12	24.59	14.69	16.92	49.15	36.37	41.17	27.53	59.78	14.87	17.18	35.07	16.20	19.95	65.95	53.60	89.16	38.41	83.17	67.83	72.64	22.35	34.15	31.92	29.10	31.24	36.26	29.86	54.86	19.14	42.22	29.96	33.26	49.13	16.95	34.14	33.40	47.65	38.71	43.49	48.85	49.72	43.30	62.48	26.90	23.34	25.00	24.33	33.61	17.68	22.15
F2_147.0761_15.4_P	47.92	30.08	42.66	42.61	36.00	36.77	29.21	45.52	54.74	43.50	46.73	40.77	54.30	36.21	43.80	41.69	45.96	52.38	48.46	47.92	54.09	51.18	53.39	37.35	54.01	38.42	43.21	48.95	44.28	41.37	58.96	55.70	63.44	58.31	55.06	46.77	48.35	52.37	30.83	32.46	34.57	31.28	32.53	22.82	31.48	32.39	31.89	27.53	40.12	31.14	41.13	35.01	27.76
F3_118.0859_12.6_P	17.91	13.26	16.46	19.06	14.13	17.81	11.88	20.00	18.11	14.46	18.85	17.43	22.34	10.36	23.80	25.79	24.59	21.80	17.05	19.91	26.47	23.01	21.19	15.50	19.61	18.10	16.76	16.42	21.71	20.85	28.79	27.19	28.31	23.20	31.59	25.50	26.74	29.30	26.00	24.45	26.60	21.23	23.65	30.58	28.05	29.72	23.65	18.88	26.31	28.88	30.99	26.12	21.48
F4_130.086_11_P	0.515	0.701	0.931	0.927	0.760	0.769	0.671	1.002	1.070	0.780	0.908	0.939	1.057	0.256	0.875	0.646	0.834	1.012	0.661	0.848	0.970	0.728	0.954	0.785	0.913	0.758	0.658	0.721	0.488	0.657	0.865	0.905	0.736	0.951	0.755	0.800	0.943	0.900	0.458	0.445	0.460	0.444	0.426	0.437	0.399	0.464	0.457	0.465	0.705	0.637	0.484	0.513	0.518
F5_179.056_15.4_N	0.919	0.490	0.928	0.965	0.935	0.856	1.380	1.223	1.215	0.817	1.266	0.795	1.243	1.853	1.112	1.361	1.281	1.156	1.549	1.099	1.502	2.034	2.003	0.920	1.843	0.932	0.802	0.928	1.395	1.308	1.748	1.550	1.668	2.568	1.491	0.942	1.524	1.189	0.565	0.481	0.547	0.745	0.870	0.614	0.657	0.676	0.797	0.678	0.819	0.628	0.694	0.926	0.914
F6_179.0559_14.9_N	0.475	0.307	0.228	0.256	0.370	0.272	0.371	0.300	0.399	0.329	0.415	0.341	0.424	1.437	0.402	0.325	0.411	0.442	0.421	0.500	0.584	0.497	0.576	0.369	0.608	0.341	0.272	0.404	0.667	0.323	0.685	0.507	0.779	0.365	0.566	0.344	0.506	0.332	0.184	0.152	0.194	0.272	0.341	0.202	0.275	0.317	0.359	0.250	0.303	0.262	0.259	0.424	0.302
F7_139.05_11.6_P	0.222	0.230	0.193	0.466	0.070	0.119	0.591	0.054	0.158	0.200	0.136	0.161	0.132	0.908	0.036	0.268	0.539	0.225	0.109	0.102	0.167	0.163	0.162	0.158	0.156	0.055	0.109	0.530	0.083	0.189	0.315	0.218	0.426	0.117	0.177	0.236	0.101	0.387	0.104	0.066	0.132	0.148	0.054	0.068	0.198	0.065	0.225	0.049	0.723	0.067	0.195	0.065	0.267
F9_136.0402_11.5_N	0.162	0.151	0.232	0.201	0.270	0.254	0.085	0.455	0.233	0.065	0.143	0.387	0.217	0.161	0.196	0.153	0.101	0.091	0.079	0.104	0.157	0.160	0.113	0.118	0.103	0.132	0.097	0.072	0.206	0.132	0.184	0.170	0.206	0.177	0.206	0.175	0.263	0.171	0.081	0.084	0.104	0.106	0.174	0.071	0.160	0.112	0.282	0.250	0.168	0.210	0.142	0.083	0.086
F10_76.0755_10.7_P	28.99	28.13	32.10	27.49	56.30	66.00	27.65	41.80	37.89	46.94	50.59	57.21	50.29	20.69	46.00	42.38	38.73	31.52	32.99	25.11	24.69	28.48	33.77	50.79	52.27	43.93	64.64	42.37	57.68	36.43	32.95	28.16	27.22	44.13	19.79	37.53	48.42	21.83	76.77	54.76	49.36	24.24	41.70	43.57	37.10	28.52	18.49	25.68	17.27	21.37	16.21	21.36	21.54
F12_135.0299_13.7_N	0.563	1.049	0.477	0.713	0.886	0.901	0.781	0.953	0.490	0.648	0.806	0.473	0.701	0.726	1.087	0.670	0.723	0.643	0.393	0.371	0.445	0.640	0.479	1.045	0.409	0.770	0.755	0.677	0.855	0.918	0.916	0.812	0.573	0.532	0.377	0.766	0.660	0.350	0.765	0.846	1.210	0.846	1.118	0.652	0.980	0.823	1.411	1.772	1.079	1.095	0.902	1.372	0.973
F13_118.086_11.2_P	103.7	151.4	102.4	113.8	126.8	129.6	89.3	149.6	126.5	129.0	107.4	143.3	138.5	76.0	132.3	132.6	128.8	123.9	85.2	133.4	109.8	106.4	100.2	126.4	149.5	113.2	143.5	148.5	128.4	135.2	105.9	146.7	101.5	106.9	106.0	126.5	127.8	134.5	98.62	91.13	93.29	107.4	91.12	101.6	91.64	98.62	137.3	126.8	114.5	112.1	77.40	137.8	131.0
F14_130.086_12.4_P	0.183	1.230	0.238	0.150	1.576	0.272	1.627	2.100	0.265	0.162	2.081	0.210	0.100	1.051	2.239	2.305	0.298	0.155	0.200	0.151	2.371	0.216	0.186	0.252	0.171	0.202	0.180	0.274	0.215	2.210	0.563	0.187	0.110	0.197	0.146	1.014	0.166	0.346	0.152	1.647	1.943	1.510	2.271	0.287	0.374	0.419	0.240	0.260	0.261	2.267	0.172	1.780	0.074
F15_130.0861_14.2_P	0.029	0.006	0.027	0.016	0.053	0.041	0.018	0.071	0.032	0.068	0.063	0.056	0.031	0.246	0.032	0.057	0.033	0.023	0.023	0.027	0.030	0.025	0.061	0.031	0.063	0.035	0.048	0.054	0.141	0.050	0.029	0.029	0.042	0.064	0.084	0.059	0.032	0.030	0.086	0.073	0.070	0.049	0.072	0.054	0.063	0.065	0.072	0.070	0.085	0.058	0.071	0.064	0.051
F16_148.06_15.3_P	1.935	1.121	2.362	1.853	4.445	3.480	1.150	2.624	2.234	7.414	3.189	3.916	1.986	2.474	2.844	3.620	3.470	1.667	2.218	1.980	2.222	1.911	2.008	2.708	3.785	2.564	4.434	3.175	4.222	3.539	2.050	2.156	1.922	3.188	2.473	3.470	4.032	2.309	2.777	2.852	2.940	2.461	2.632	1.767	2.492	2.543	3.889	4.522	3.243	2.493	2.284	3.097	4.516
F17_132.0765_14.9_P	64.08	45.31	89.46	84.17	96.18	68.88	39.86	56.87	49.09	121.3	86.26	83.89	41.75	47.68	52.91	62.01	63.00	91.04	66.05	54.70	45.06	85.09	79.99	98.50	53.73	121.9	57.64	41.82	63.71	54.18	55.72	100.5	57.76	114.5	107.9	61.04	84.62	39.10	50.71	68.95	76.94	48.72	37.61	40.49	60.74	50.62	79.95	122.9	52.05	72.98	73.06	66.42	62.60
F18_166.0858_10_P	10.31	7.527	14.64	10.39	18.80	18.02	6.086	18.43	15.84	10.28	16.90	15.03	19.18	6.046	19.51	17.90	16.96	12.08	9.946	15.19	18.29	18.21	14.82	13.93	14.58	15.27	16.62	13.28	17.66	17.92	16.32	19.59	19.43	17.52	19.16	15.66	21.29	14.94	20.70	24.12	28.56	16.35	23.51	21.10	25.60	26.26	14.90	13.15	16.34	16.19	23.63	16.01	9.349
F19_117.0193_16.3_N	0.104	0.091	0.088	0.126	0.176	0.216	0.103	0.085	0.088	0.189	0.119	0.208	0.084	0.437	0.172	0.145	0.202	0.111	0.096	0.103	0.097	0.070	0.087	0.128	0.186	0.114	0.317	0.159	0.291	0.151	0.122	0.123	0.180	0.143	0.096	0.234	0.188	0.337	0.285	0.295	0.176	0.159	0.272	0.174	0.185	0.276	0.301	0.266	0.251	0.285	0.272	0.299	0.294
F20_135.0299_13.3_N	0.707	0.978	0.595	0.638	0.544	0.773	0.600	0.690	0.556	0.439	0.502	0.623	0.680	0.617	0.677	0.670	0.760	0.784	0.748	0.554	0.525	0.550	0.617	0.697	0.488	0.562	0.738	0.463	0.849	0.687	0.615	0.768	0.436	0.621	0.578	0.766	0.952	0.810	0.633	0.646	0.785	0.637	0.857	0.696	0.606	0.681	0.604	0.661	0.551	0.745	0.791	0.638	0.776
F21_104.0704_15.7_P	2.377	1.272	1.033	0.833	1.885	1.660	1.046	1.489	1.510	1.215	1.647	1.672	1.491	12.22	2.006	1.595	1.158	0.866	1.153	1.160	1.088	2.161	1.500	1.332	1.746	1.693	2.521	3.010	2.511	2.546	1.368	1.702	1.789	2.190	1.653	2.992	1.753	2.050	1.681	2.910	2.611	2.434	2.199	2.476	2.474	2.868	3.877	3.762	3.908	3.580	3.304	2.687	4.015
F22_132.1016_10.7_P	21.80	15.92	20.96	20.81	17.66	17.40	14.26	21.39	22.64	16.77	21.73	19.32	23.65	14.21	25.40	23.74	29.02	25.61	21.50	28.43	35.61	27.10	24.16	15.64	19.24	21.72	18.64	19.23	21.69	22.23	34.37	30.64	37.34	25.74	35.56	28.53	27.47	28.43	33.45	29.55	36.75	30.78	35.23	45.27	38.35	42.02	29.47	25.69	32.25	33.93	41.96	29.37	20.53
F23_244.0923_12.1_P	0.766	0.472	0.641	0.623	1.137	1.164	0.200	0.858	0.842	1.134	1.174	1.215	0.943	0.624	1.319	1.016	1.162	0.756	0.547	0.817	0.554	0.686	0.711	0.832	1.111	0.830	1.303	0.535	1.417	1.686	0.927	1.092	0.938	1.083	1.197	1.685	1.373	1.213	1.488	1.339	1.166	0.932	1.307	1.168	2.042	1.537	2.364	1.677	2.559	2.118	2.777	2.143	1.966
F24_116.0704_7.7_P	0.038	0.030	0.059	0.038	0.085	0.097	0.026	0.092	0.071	0.034	0.074	0.067	0.085	0.182	0.087	0.077	0.068	0.056	0.035	0.052	0.052	0.072	0.061	0.071	0.068	0.076	0.092	0.050	0.103	0.067	0.074	0.078	0.058	0.081	0.066	0.086	0.122	0.057	0.074	0.081	0.078	0.043	0.082	0.084	0.060	0.086	0.046	0.045	0.040	0.060	0.078	0.045	0.023
F25_193.0351_17.1_N	0.063	0.078	0.042	0.059	0.024	0.036	0.056	0.031	0.042	0.049	0.046	0.044	0.031	0.121	0.036	0.032	0.051	0.062	0.069	0.044	0.022	0.037	0.035	0.039	0.056	0.035	0.020	0.050	0.034	0.035	0.063	0.050	0.050	0.054	0.053	0.043	0.039	0.041	0.018	0.008	0.016	0.051	0.002	0.016	0.013	0.007	0.043	0.024	0.034	0.032	0.025	0.025	0.047
F26_180.0652_8.7_P	0.680	0.377	0.661	0.468	1.352	1.024	0.454	1.374	0.618	0.561	0.663	0.809	0.715	0.313	0.774	1.035	0.815	0.550	0.646	0.680	0.887	0.822	0.635	1.201	0.952	1.125	1.609	1.156	1.143	1.172	0.745	1.102	0.962	1.162	1.202	1.214	1.812	0.805	1.530	1.472	1.720	1.482	2.167	1.077	1.615	1.699	2.042	2.816	2.620	2.318	2.004	3.682	1.736
F27_146.0459_11.3_N	0.097	0.076	0.072	0.080	0.093	0.081	0.075	0.065	0.063	0.080	0.072	0.080	0.050	0.031	0.076	0.064	0.060	0.080	0.050	0.057	0.039	0.064	0.054	0.078	0.065	0.068	0.073	0.071	0.069	0.057	0.062	0.060	0.059	0.049	0.060	0.084	0.059	0.047	0.054	0.049	0.071	0.077	0.071	0.077	0.072	0.061	0.133	0.239	0.108	0.111	0.121	0.119	0.094
F28_120.0653_14.8_P	3.267	4.751	3.643	4.105	3.634	3.876	5.765	3.964	5.812	3.048	3.738	4.573	6.781	5.227	5.312	5.820	4.116	5.097	3.812	3.458	7.674	5.565	4.879	4.829	6.595	5.114	7.080	7.240	4.397	3.211	9.064	6.855	5.950	2.932	3.051	6.382	5.138	4.385	4.089	4.654	4.591	4.318	4.787	6.883	4.971	7.393	4.193	3.634	5.889	4.951	4.154	4.393	5.730
F29_126.0217_15.9_P	9.637	7.991	8.657	8.138	12.41	12.37	10.20	13.68	9.472	11.53	13.15	10.39	11.55	16.49	10.91	15.18	11.75	8.122	10.75	11.88	11.64	10.37	10.42	12.58	11.01	11.14	14.22	15.13	17.02	14.53	8.639	10.33	11.52	11.26	10.96	13.83	10.94	15.67	12.51	12.88	14.00	12.46	14.82	11.60	14.11	13.21	22.47	24.26	18.64	20.74	18.23	25.54	18.59
F30_385.1284_13.4_P	0.036	0.016	0.021	0.024	0.023	0.028	0.014	0.030	0.019	0.018	0.023	0.034	0.024	0.044	0.037	0.029	0.039	0.024	0.024	0.017	0.023	0.027	0.021	0.038	0.029	0.043	0.037	0.020	0.054	0.034	0.032	0.040	0.040	0.032	0.024	0.031	0.039	0.033	0.019	0.028	0.027	0.028	0.024	0.025	0.029	0.025	0.029	0.023	0.042	0.079	0.027	0.027	0.023
F31_189.1593_22.7_P	0.260	0.140	0.314	0.177	0.348	0.341	0.072	0.192	0.332	0.250	0.303	0.198	0.322	0.089	0.293	0.340	0.452	0.351	0.193	0.244	0.320	0.408	0.349	0.436	0.325	0.270	0.322	0.336	0.409	0.293	0.626	0.446	0.341	0.474	0.466	0.494	0.350	0.284	0.271	0.281	0.431	0.259	0.303	0.170	0.496	0.328	0.659	0.992	0.563	0.611	1.059	0.695	0.280
F32_191.0197_17.1_N	0.389	0.390	0.169	0.320	0.215	0.196	0.230	0.278	0.594	0.291	0.218	0.284	0.287	0.260	0.332	0.196	0.430	0.341	0.292	0.255	0.355	0.246	0.183	0.514	0.289	0.187	0.199	0.529	0.172	0.265	0.326	0.298	0.330	0.333	0.349	0.183	0.333	0.486	0.148	0.061	0.260	0.211	0.018	0.109	0.102	0.023	0.224	0.107	0.221	0.056	0.075	0.095	0.213
F33_132.1016_11.2_P	11.44	8.880	8.871	10.77	8.154	9.482	8.409	9.849	11.77	8.367	11.50	8.631	11.60	8.745	13.64	11.34	15.18	13.24	13.47	12.83	17.16	14.68	12.88	6.759	8.676	9.619	8.716	7.296	10.85	9.259	19.01	13.84	20.34	13.31	16.87	14.14	15.97	16.22	17.27	18.32	20.64	14.35	14.31	23.48	18.69	21.22	11.79	9.704	14.30	16.55	21.59	12.95	12.97
F34_167.0208_13.6_N	0.004	0.326	0.397	0.308	1.070	0.880	0.005	0.670	0.623	0.919	0.826	1.373	0.591	1.181	0.958	0.027	0.866	0.474	0.580	0.579	0.635	0.407	0.371	0.288	0.782	0.427	0.932	0.002	1.047	0.732	0.625	0.907	0.597	0.970	0.850	0.721	1.557	0.574	0.444	0.478	0.255	0.214	0.112	0.073	0.253	0.201	0.624	0.631	0.733	0.373	0.861	0.426	1.126
F35_195.051_16.1_N	0.061	0.063	0.088	0.065	0.141	0.106	0.062	0.102	0.079	0.121	0.178	0.090	0.088	0.259	0.091	0.133	0.169	0.102	0.105	0.046	0.037	0.209	0.081	0.169	0.115	0.200	0.121	0.067	0.238	0.168	0.081	0.095	0.057	0.073	0.065	0.083	0.094	0.059	0.082	0.096	0.153	0.061	0.134	0.069	0.111	0.079	0.089	0.316	0.074	0.102	0.160	0.156	0.050
F36_112.0503_11.4_P	0.053	0.026	0.046	0.043	0.057	0.104	0.054	0.070	0.044	0.045	0.058	0.068	0.073	0.034	0.058	0.096	0.106	0.068	0.051	0.039	0.049	0.061	0.064	0.043	0.051	0.038	0.066	0.035	0.072	0.063	0.063	0.069	0.047	0.092	0.081	0.102	0.124	0.076	0.085	0.074	0.094	0.072	0.065	0.085	0.078	0.076	0.105	0.079	0.092	0.166	0.174	0.086	0.074
F37_400.3414_4_P	0.212	0.128	0.169	0.244	0.212	0.141	0.214	0.145	0.314	0.096	0.122	0.146	0.131	0.074	0.193	0.193	0.079	0.298	0.091	0.312	0.185	0.057	0.107	0.192	0.190	0.198	0.214	0.223	0.145	0.394	0.193	0.170	0.184	0.143	0.209	0.147	0.076	0.268	0.041	0.019	0.026	0.056	0.073	0.111	0.073	0.061	0.155	0.048	0.242	0.153	0.140	0.263	0.118
F38_429.3733_3.3_N	0.260	0.249	0.367	0.570	0.554	0.707	0.368	0.711	0.428	0.409	0.523	0.364	0.510	0.640	0.554	0.534	0.761	0.372	0.209	0.351	0.640	0.508	0.602	0.611	0.443	0.633	0.781	0.493	0.496	0.383	0.690	0.528	0.643	0.491	0.456	0.565	0.793	0.639	0.469	0.420	0.377	0.213	0.392	0.256	0.194	0.164	0.459	0.343	0.401	0.391	0.417	0.155	0.419
F39_286.1027_8.1_P	0.003	0.004	0.004	0.002	0.006	0.007	0.005	0.004	0.003	0.004	0.003	0.008	0.005	0.023	0.005	0.006	0.005	0.003	0.002	0.002	0.001	0.005	0.005	0.005	0.003	0.004	0.007	0.004	0.013	0.005	0.007	0.010	0.003	0.005	0.007	0.011	0.007	0.003	0.015	0.005	0.002	0.001	0.001	0.003	0.006	0.001	0.005	0.001	0.005	0.011	0.023	0.007	0.004
F40_103.04_10.2_N	0.075	0.057	0.093	0.069	0.108	0.156	0.052	0.081	0.062	0.039	0.121	0.123	0.132	0.534	0.123	0.140	0.060	0.054	0.047	0.071	0.074	0.106	0.087	0.074	0.071	0.100	0.126	0.059	0.152	0.085	0.105	0.111	0.112	0.059	0.109	0.092	0.113	0.042	0.283	0.298	0.254	0.125	0.266	0.418	0.218	0.397	0.096	0.118	0.111	0.141	0.169	0.102	0.039
F41_172.0978_4.5_N	0.000	0.000	0.003	0.000	0.002	0.003	0.001	0.002	0.000	0.001	0.004	0.005	0.003	0.002	0.004	0.003	0.003	0.000	0.000	0.003	0.003	0.003	0.003	0.003	0.003	0.003	0.003	0.000	0.003	0.003	0.000	0.003	0.003	0.002	0.003	0.003	0.004	0.003	0.005	0.005	0.007	0.000	0.004	0.010	0.010	0.008	0.004	0.004	0.003	0.005	0.005	0.004	0.000
F42_145.0142_17_N	0.104	0.194	0.254	0.204	0.426	0.496	0.177	0.254	0.215	0.038	0.222	0.470	0.214	0.112	0.268	0.411	0.182	0.104	0.109	0.189	0.210	0.087	0.226	0.209	0.421	0.092	0.588	0.283	0.429	0.155	0.218	0.239	0.212	0.237	0.212	0.277	0.305	0.197	0.339	0.342	0.280	0.134	0.170	0.346	0.198	0.248	0.320	0.058	0.319	0.219	0.238	0.242	0.401
F43_198.1275_5_P	0.005	0.007	0.008	0.003	0.005	0.009	0.006	0.012	0.007	0.003	0.002	0.005	0.003	0.022	0.006	0.004	0.003	0.004	0.004	0.003	0.003	0.002	0.010	0.010	0.004	0.016	0.005	0.025	0.003	0.002	0.003	0.003	0.003	0.004	0.002	0.003	0.129	0.046	0.002	0.002	0.005	0.003	0.002	0.001	0.002	0.023	0.003	0.003	0.203	0.012	0.002	0.018	0.005
F44_132.0301_15.8_N	0.209	0.208	0.216	0.292	0.296	0.296	0.166	0.301	0.201	0.202	0.229	0.143	0.220	0.122	0.216	0.202	0.206	0.169	0.169	0.142	0.197	0.193	0.197	0.329	0.220	0.256	0.255	0.295	0.332	0.260	0.308	0.335	0.381	0.363	0.341	0.349	0.276	0.232	0.285	0.260	0.390	0.292	0.417	0.210	0.388	0.295	0.705	0.854	0.687	0.815	0.638	0.695	0.486
F45_179.0559_17.8_N	0.426	0.730	0.461	0.922	0.250	0.312	0.367	0.417	0.549	0.578	0.356	0.449	0.460	0.246	0.389	0.184	0.464	0.645	0.388	0.458	0.475	0.349	0.336	0.396	0.596	0.331	0.219	0.499	0.147	0.225	0.415	0.547	0.210	0.461	0.478	0.396	0.459	0.446	0.111	0.106	0.073	0.344	0.092	0.113	0.046	0.061	0.286	0.130	0.365	0.149	0.121	0.107	0.546

F46_90.0548_15.7_P	4.275	2.062	3.528	2.858	3.941	3.068	2.571	2.398	2.834	4.731	5.873	2.367	3.572	3.267	2.718	4.489	4.274	2.377	5.738	3.365	3.093	3.503	3.747	3.642	2.741	5.031	3.098	2.533	5.226	3.928	3.320	3.182	4.296	4.917	5.902	4.369	2.972	4.458	1.523	1.657	4.820	4.479	4.353	1.206	3.519	2.897	7.252	6.606	7.391	6.356	7.499	8.960	5.182
F47_170.0921_13.1_P	1.549	0.718	2.354	2.369	3.560	1.730	0.601	1.198	0.768	5.478	2.475	0.970	0.841	1.216	1.739	4.292	2.197	1.108	1.661	0.813	0.293	3.867	1.009	6.278	0.959	10.63	1.485	1.624	4.219	0.915	0.665	2.168	0.771	3.356	2.076	2.572	1.999	0.622	0.857	1.382	3.881	0.885	1.156	0.830	1.401	2.428	4.350	10.37	1.018	2.404	6.654	2.533	1.772
F48_90.0548_15.1_P	9.300	4.778	6.924	5.001	6.182	6.076	5.047	7.174	7.025	6.061	6.903	5.831	6.257	6.542	5.509	5.741	7.450	8.038	10.66	8.937	9.188	8.308	8.103	5.008	6.616	6.538	5.919	7.258	5.331	8.236	9.337	7.806	9.879	10.51	12.58	8.235	8.082	9.347	3.786	3.530	5.634	6.338	6.233	2.898	5.697	5.811	8.642	7.167	8.657	9.377	9.133	6.907	7.770
F49_151.061_13.3_N	0.044	0.075	0.043	0.058	0.048	0.057	0.064	0.059	0.044	0.043	0.033	0.050	0.055	0.045	0.050	0.042	0.048	0.057	0.050	0.047	0.036	0.031	0.036	0.053	0.043	0.048	0.060	0.049	0.047	0.044	0.041	0.054	0.028	0.042	0.040	0.052	0.066	0.057	0.047	0.049	0.043	0.049	0.043	0.046	0.039	0.037	0.043	0.042	0.051	0.056	0.049	0.042	0.068
F50_112.0503_9_P	0.007	0.005	0.006	0.009	0.014	0.015	0.027	0.011	0.008	0.008	0.008	0.010	0.011	0.096	0.014	0.012	0.009	0.008	0.010	0.007	0.007	0.009	0.009	0.012	0.008	0.009	0.014	0.010	0.016	0.012	0.008	0.010	0.008	0.010	0.010	0.012	0.013	0.008	0.012	0.013	0.010	0.007	0.012	0.011	0.011	0.014	0.023	0.021	0.024	0.023	0.042	0.027	0.015
F51_137.0455_9.5_P	0.209	0.134	0.142	0.247	0.331	0.334	0.148	0.316	0.094	0.127	0.102	0.130	0.154	0.103	0.155	0.155	0.146	0.136	0.065	0.099	0.133	0.143	0.132	0.310	0.134	0.302	0.159	0.355	0.222	0.209	0.185	0.201	0.136	0.157	0.154	0.314	0.201	0.173	0.175	0.238	0.281	0.238	0.279	0.144	0.234	0.264	0.717	0.976	0.411	0.522	0.422	0.628	0.309
F52_252.1086_7.8_P	0.101	0.135	0.040	0.096	0.075	0.115	0.077	0.096	0.026	0.022	0.018	0.071	0.129	0.092	0.088	0.082	0.043	0.053	0.013	0.010	0.027	0.053	0.049	0.190	0.023	0.163	0.049	0.200	0.096	0.120	0.063	0.103	0.049	0.047	0.049	0.170	0.144	0.113	0.153	0.182	0.191	0.140	0.234	0.253	0.090	0.168	0.185	0.172	0.120	0.207	0.058	0.195	0.080
F53_150.058_11.6_P	7.453	6.489	9.655	8.655	10.92	11.78	4.985	12.81	9.790	6.327	9.435	9.684	11.99	4.261	12.50	12.70	11.70	8.965	6.176	9.768	14.12	11.99	9.179	11.21	9.898	12.69	12.00	9.290	10.57	10.21	11.61	12.77	11.28	9.684	9.824	11.17	14.08	9.809	19.94	25.18	25.18	15.78	22.31	28.10	22.07	27.91	7.968	7.671	10.10	10.29	12.20	10.13	6.339
F54_157.0606_11.7_P	0.003	0.008	0.002	0.004	0.002	0.006	0.009	0.004	0.002	0.003	0.002	0.197	0.002	0.047	0.002	0.005	0.002	0.003	0.004	0.003	0.002	0.006	0.002	0.003	0.002	0.002	0.004	0.002	0.004	0.006	0.003	0.002	0.002	0.003	0.004	0.012	0.003	0.003	0.003	0.004	0.006	0.004	0.003	0.007	0.005	0.008	0.003	0.002	0.003	0.012	0.009	0.003	0.007
F55_167.9971_13.4_N	0.020	0.020	0.017	0.014	0.019	0.031	0.020	0.022	0.013	0.009	0.016	0.021	0.021	0.012	0.024	0.025	0.025	0.022	0.022	0.016	0.011	0.022	0.022	0.024	0.013	0.018	0.023	0.013	0.037	0.030	0.017	0.023	0.009	0.018	0.018	0.031	0.029	0.019	0.043	0.042	0.066	0.035	0.076	0.041	0.033	0.065	0.015	0.015	0.014	0.024	0.035	0.015	0.014
F56_181.0504_9.7_N	0.017	0.006	0.015	0.010	0.018	0.022	0.006	0.016	0.019	0.020	0.044	0.021	0.016	0.018	0.021	0.022	0.026	0.019	0.015	0.025	0.014	0.027	0.020	0.020	0.028	0.013	0.023	0.010	0.047	0.027	0.025	0.029	0.019	0.029	0.028	0.023	0.028	0.028	0.016	0.028	0.030	0.017	0.029	0.014	0.020	0.035	0.021	0.017	0.018	0.031	0.066	0.029	0.011
F57_222.0967_11.9_P	0.044	0.023	0.046	0.036	0.061	0.054	0.045	0.080	0.063	0.042	0.059	0.061	0.069	0.137	0.078	0.097	0.067	0.051	0.027	0.051	0.048	0.062	0.076	0.062	0.058	0.050	0.058	0.070	0.082	0.075	0.061	0.063	0.044	0.068	0.073	0.082	0.101	0.070	0.057	0.040	0.056	0.046	0.069	0.047	0.065	0.072	0.062	0.065	0.067	0.065	0.058	0.087	0.033
F59_146.1172_13.2_P	4.825	3.036	6.093	3.652	7.186	8.524	4.939	8.998	7.254	9.353	13.18	12.25	7.043	4.868	11.01	15.96	7.119	7.012	4.876	7.035	10.31	10.26	9.036	8.346	7.152	12.17	13.82	9.036	11.22	18.45	8.346	10.34	10.95	7.369	8.395	14.88	9.058	8.242	6.583	10.42	16.29	10.61	16.81	20.18	8.67	17.12	15.49	14.64	16.11	11.26	13.76	35.38	6.838
F60_179.056_12.6_N	0.087	0.646	0.150	0.043	0.122	0.262	0.190	0.361	0.287	0.052	0.111	0.207	0.496	0.083	0.287	0.273	0.099	0.077	0.136	0.052	0.048	0.062	0.125	0.223	0.067	0.044	0.157	0.070	0.083	0.138	0.096	0.222	0.131	0.099	0.445	0.075	0.352	0.068	0.147	0.034	0.137	0.088	0.050	0.055	0.220	0.172	0.094	0.052	0.679	0.325	0.217	0.455	0.090
F61_114.0659_9.7_P	2.314	1.163	1.755	1.486	1.465	9.146	1.724	1.528	0.464	0.442	0.298	4.005	0.372	0.722	0.699	3.755	7.914	1.445	2.105	1.019	0.553	1.526	3.346	1.178	0.338	1.621	1.553	0.475	5.600	2.294	0.470	0.480	0.293	0.615	0.526	3.065	0.981	0.454	22.92	22.41	30.95	19.77	23.83	22.50	32.42	26.69	0.307	0.664	0.306	1.741	5.150	0.352	0.204
F62_128.0354_10.7_N	1.605	1.374	1.888	1.803	2.605	2.592	1.486	2.509	1.787	1.403	2.369	1.936	2.501	1.434	2.455	2.483	2.301	1.480	1.210	1.598	2.068	2.276	2.161	2.251	2.181	1.953	2.634	1.927	2.985	2.026	2.612	2.293	2.814	1.955	2.001	2.661	2.290	2.282	2.756	2.771	3.155	1.458	2.427	2.308	2.973	3.130	2.001	1.948	2.451	2.421	3.117	2.265	1.360
F63_111.02_10_N	0.030	0.022	0.017	0.019	0.053	0.070	0.017	0.026	0.044	0.096	0.058	0.116	0.066	0.063	0.091	0.068	0.085	0.043	0.047	0.046	0.040	0.030	0.023	0.032	0.084	0.040	0.071	0.055	0.041	0.081	0.048	0.058	0.039	0.039	0.044	0.074	0.079	0.068	0.062	0.049	0.031	0.027	0.028	0.027	0.038	0.058	0.048	0.078	0.077	0.043	0.024	0.063	0.040
F64_189.123_12.7_P	0.108	0.101	0.100	0.096	0.150	0.131	0.081	0.123	0.077	0.122	0.102	0.124	0.221	0.087	0.219	0.156	0.110	0.164	0.080	0.104	0.097	0.173	0.133	0.220	0.093	0.181	0.172	0.169	0.189	0.170	0.142	0.209	0.120	0.128	0.126	0.203	0.189	0.099	0.277	0.281	0.566	0.325	0.359	0.273	0.400	0.363	0.270	0.570	0.210	0.266	0.180	0.339	0.170
F65_162.112_13.2_P	3.679	1.519	4.201	2.777	5.198	5.505	2.625	5.027	3.873	6.190	6.857	7.175	4.957	2.792	5.690	7.820	5.688	4.169	3.921	3.166	5.480	6.639	6.043	4.712	5.569	4.730	7.877	5.545	9.169	8.191	6.161	5.967	7.187	7.340	5.875	8.982	5.584	4.622	3.943	5.080	8.468	6.014	6.906	4.840	4.629	6.328	11.65	13.74	12.00	9.776	12.45	23.22	5.270
F66_175.1185_27.1_P	9.647	7.369	9.484	11.28	11.47	11.03	4.000	12.75	12.12	10.83	14.01	10.15	15.07	8.18	11.71	11.49	11.31	13.61	10.45	14.09	15.17	14.89	12.31	10.41	12.76	10.63	12.29	7.052	16.07	14.51	17.31	18.64	22.39	20.47	20.45	16.24	15.33	13.48	7.877	7.196	6.229	7.131	9.058	5.941	9.128	9.560	12.09	11.60	13.40	13.86	17.17	13.59	10.63
F67_305.2484_3.3_N	0.247	0.435	0.624	0.475	0.635	0.652	0.314	0.487	0.509	0.298	0.590	0.479	0.778	0.158	0.611	0.508	0.357	0.231	0.196	0.428	0.457	0.499	0.404	0.704	0.552	0.629	0.476	0.423	0.441	0.358	0.500	0.306	0.403	0.293	0.208	0.369	0.226	0.328	0.331	0.294	0.160	0.112	0.333	0.432	0.229	0.282	0.242	0.289	0.230	0.298	0.245	0.247	0.186
F68_141.0656_9.5_P	0.093	0.060	0.083	0.035	0.082	0.300	0.047	0.075	0.047	0.033	0.097	0.377	0.049	0.030	0.072	0.107	0.244	0.055	0.072	0.090	0.041	0.072	0.168	0.048	0.042	0.049	0.063	0.047	0.355	0.066	0.057	0.084	0.049	0.071	0.063	0.150	0.075	0.077	0.389	0.534	0.419	0.154	0.372	0.478	0.658	0.454	0.101	0.033	0.026	0.114	0.265	0.039	0.027
F69_279.2329_3.4_N	1.933	2.912	4.514	3.082	4.893	5.502	1.872	3.845	3.975	2.579	4.473	3.761	5.425	1.547	4.691	3.620	2.749	1.946	1.647	3.772	3.327	4.425	4.017	4.813	4.020	3.709	3.156	2.710	3.736	2.987	2.915	2.359	2.942	2.442	1.756	2.834	1.701	2.546	2.291	2.312	1.524	1.119	2.490	2.734	2.192	2.925	1.782	1.805	1.955	2.600	2.517	2.442	1.386
F70_133.0605_12.7_P	0.103	0.096	0.053	0.069	0.104	0.138	0.018	0.083	0.113	0.264	0.111	0.300	0.102	0.202	0.153	0.121	0.118	0.141	0.155	0.134	0.051	0.059	0.092	0.060	0.140	0.065	0.147	0.006	0.158	0.280	0.135	0.119	0.096	0.279	0.288	0.205	0.228	0.219	0.080	0.040	0.072	0.122	0.033	0.031	0.073	0.027	0.361	0.157	0.307	0.283	0.155	0.237	0.477
F71_137.0455_11.1_P	11.99	12.24	11.21	11.97	13.10	10.88	12.12	11.27	10.85	8.878	10.17	10.68	10.54	9.857	8.746	9.339	13.35	10.27	11.25	13.21	11.76	8.764	9.602	11.17	10.87	11.18	12.90	12.32	12.52	14.80	12.71	10.29	13.05	11.31	11.01	11.56	14.20	17.17	16.31	14.78	14.87	19.62	19.12	12.62	20.18	15.28	16.30	16.12	18.22	18.24	15.77	15.15	18.33
F72_133.0604_15.7_P	0.023	0.009	0.025	0.021	0.039	0.042	0.009	0.060	0.027	0.024	0.036	0.026	0.044	0.025	0.033	0.041	0.038	0.033	0.012	0.022	0.036	0.039	0.045	0.048	0.039	0.027	0.030	0.029	0.034	0.040	0.033	0.038	0.051	0.037	0.036	0.040	0.037	0.034	0.028	0.030	0.027	0.018	0.033	0.036	0.025	0.030	0.025	0.026	0.032	0.042	0.029	0.038	0.015
F73_337.311_3.2_N	2.252	3.007	5.475	3.260	5.478	5.546	1.954	4.485	5.024	3.534	4.798	4.003	6.149	2.236	5.464	4.831	4.080	2.207	2.428	4.924	4.158	6.594	5.053	5.679	4.694	4.123	3.961	3.074	5.127	3.331	3.720	3.143	4.239	2.484	1.926	3.666	2.024	3.298	2.495	2.517	1.784	0.800	3.105	3.232	2.026	3.159	3.112	2.428	3.805	3.489	3.804	3.840	1.853
F74_435.2509_3.9_N	0.104	0.094	0.074	0.096	0.060	0.067	0.076	0.071	0.115	0.061	0.062	0.066	0.063	0.019	0.077	0.041	0.060	0.076	0.065	0.084	0.068	0.057	0.072	0.081	0.073	0.088	0.055	0.082	0.061	0.050	0.069	0.050	0.060	0.032	0.059	0.060	0.036	0.044	0.075	0.056	0.073	0.109	0.070	0.052	0.071	0.064	0.086	0.065	0.052	0.066	0.050	0.058	0.037
F76_141.0656_9.8_P	0.076	0.027	0.039	0.018	0.078	0.089	0.062	0.008	0.026	0.049	0.035	0.153	0.014	0.025	0.024	0.107	0.296	0.045	0.130	0.046	0.007	0.036	0.099	0.015	0.023	0.009	0.030	0.027	0.130	0.018	0.025	0.015	0.011	0.027	0.048	0.041	0.011	0.072	0.068	0.015	0.055	0.042	0.030	0.046	0.146	0.041	0.062	0.032	0.062	0.063	0.124	0.045	0.050
F77_137.0454_10.4_P	5.473	3.867	6.794	5.491	8.212	8.168	4.321	7.493	6.154	6.031	4.450	5.044	3.422	2.840	4.316	5.814	5.458	3.927	4.681	4.844	4.537	3.369	3.901	6.322	5.577	6.188	6.514	7.332	5.491	6.111	5.567	5.266	4.367	5.227	7.061	8.197	7.293	7.313	12.76	11.49	11.04	15.22	15.73	10.29	15.16	13.72	13.82	12.66	19.76	19.39	16.84	10.88	17.21
F78_111.0199_11_N	0.004	0.022	0.006	0.007	0.015	0.011	0.020	0.012	0.005	0.006	0.008	0.013	0.006	0.103	0.010	0.016	0.013	0.009	0.024	0.008	0.005	0.012	0.022	0.014	0.009	0.008	0.010	0.010	0.019	0.028	0.024	0.069	0.025	0.073	0.032	0.044	0.021	0.024	0.017	0.020	0.028	0.018	0.040	0.027	0.033	0.056	0.024	0.056	0.022	0.061	0.047	0.064	0.017
F80_138.0547_9.9_P	0.046	0.031	0.051	0.021	0.074	0.124	0.023	0.050	0.040	0.061	0.037	0.181	0.024	0.027	0.046	0.079	0.066	0.025	0.060	0.065	0.034	0.043	0.044	0.066	0.046	0.050	0.065	0.033	0.115	0.051	0.028	0.039	0.022	0.043	0.022	0.048	0.029	0.048	0.410	0.925	0.824	0.239	0.370	0.194	0.875	0.409	0.037	0.039	0.019	0.042	0.040	0.025	0.015
F81_106.0498_16.3_P	1.442	1.025	0.992	1.301	1.297	1.512	0.822	1.427	1.320	1.264	1.636	0.845	1.108	1.027	1.008	1.092	1.211	1.552	1.278	1.292	1.469	1.317	1.094	1.652	1.444	0.974	1.282	1.115	1.416	1.238	1.287	1.359	1.869	1.812	1.368	1.216	0.949	1.059	1.133	1.063	1.575	1.123	1.480	0.551	1.383	1.163	0.826	0.755	1.008	1.012	1.028	1.215	0.614
F84_189.1232_10.5_P	0.183	0.065	0.126	0.117	0.129	0.141	0.041	0.165	0.108	0.184	0.156	0.111	0.140	0.088	0.121	0.168	0.114	0.127	0.100	0.173	0.124	0.222	0.173	0.156	0.144	0.194	0.222	0.282	0.235	0.273	0.176	0.145	0.132	0.156	0.143	0.259	0.148	0.208	0.109	0.168	0.241	0.265	0.258	0.176	0.301	0.225	0.625	0.617	0.439	0.475	0.401	0.395	0.314
F85_111.02_8.6_N	0.293	0.110	0.181	0.146	0.589	0.507	0.175	0.295	0.198	0.463	0.339	0.598	0.184	0.152	0.331	0.478	0.403	0.106	0.238	0.185	0.117	0.090	0.093	0.197	0.462	0.198	0.752	0.488	0.580	0.511	0.287	0.290	0.254	0.292	0.401	0.593	0.593	0.379	0.764	0.825	0.612	0.485	0.497	0.533	0.717	0.669	1.073	0.770	1.074	0.698	0.823	0.650	1.108
F87_191.1024_14.3_P	0.021	0.016	0.041	0.028	0.049	0.064	0.015	0.055	0.037	0.022	0.027	0.038	0.050	0.028	0.044	0.043	0.035	0.028	0.015	0.028	0.033	0.038	0.028	0.054	0.040	0.045	0.070	0.041	0.048	0.047	0.037	0.045	0.032	0.035	0.027	0.059	0.060	0.035	0.134	0.126	0.121	0.065	0.133	0.128	0.095	0.101	0.039	0.038	0.029	0.038	0.051	0.035	0.015
F88_150.1122_9_P	0.121	0.243	0.055	0.328	0.068	0.033	0.086	0.030	0.198	0.293	0.124	0.140	0.105	0.354	0.018	0.107	0.743	0.157	0.068	0.069	0.111	0.047	0.050	0.117	0.054	0.035	0.052	0.266	0.039	0.110	0.615	0.343	0.305	0.092	0.131	0.365	0.064	0.212	0.042	0.012	0.086	0.069	0.078	0.053	0.080	0.140	0.197	0.017	0.797	0.035	0.072	0.045	0.078
F91_163.0611_12.3_N	0.031	0.051	0.054	0.064	0.049	0.057	0.039	0.084	0.065	0.041	0.039	0.054	0.074	0.027	0.058	0.059	0.062	0.071	0.058	0.060	0.062	0.040	0.056	0.058	0.054	0.043	0.064	0.041	0.062	0.049	0.051	0.069	0.036	0.058	0.059	0.070	0.099	0.060	0.048	0.034	0.039	0.037	0.045	0.037	0.040	0.046	0.041	0.045	0.055	0.051	0.044	0.042	0.053
F92_105.0193_12.8_N	0.207	0.130	0.241	0.242	0.292	0.225	0.351	0.281	0.237	0.193	0.378	0.208	0.240	0.241	0.350	0.293	0.292	0.221	0.417	0.226	0.276	0.239	0.250	0.336	0.186	0.405	0.334	0.182	0.364	0.294	1.042	0.314	0.428	0.298	0.286	0.354	0.425	0.390	0.144	0.099	0.285	0.125	0.234	0.178	0.205	0.173	0.238	0.267	0.222	0.180	0.301	0.290	0.166
F98_180.0335_7.6_N	0.010	0.006	0.008	0.005	0.009	0.011	0.016	0.006	0.006	0.004	0.005	0.012	0.006	0.049	0.009	0.011	0.007	0.007	0.008	0.006	0.005	0.010	0.009	0.008	0.005	0.008	0.009	0.006	0.018	0.008	0.006	0.006	0.007	0.006	0.007	0.009	0.008	0.003	0.011	0.016	0.023	0.020	0.016	0.017	0.014	0.021	0.004	0.005	0.005	0.009	0.016	0.006	0.011
F99_110.0268_15.4_P	0.020	0.017	0.045	0.026	0.061	0.093	0.019	0.056	0.031	0.038	0.036	0.084	0.046	0.024	0.054	0.070	0.043	0.022	0.014	0.024	0.032	0.027	0.030	0.088	0.051	0.056	0.086	0.085	0.071	0.036	0.021	0.052	0.041	0.024	0.014	0.051	0.057	0.041	0.080	0.080	0.036	0.028	0.057	0.154	0.047	0.029	0.071	0.114	0.043	0.079	0.056	0.068	0.049
F100_335.0493_13.5_P	0.004	0.326	0.397	0.308	0.006	0.880	0.005	0.670	0.623	0.919	0.826	1.373	0.591	1.183	0.958	0.027	0.866	0.474	0.580	0.579	0.635	0.407	0.371	0.288	0.782	0.427	0.932	0.001	1.047	0.732	0.625	0.907	0.597	0.970	0.850	0.721	1.557	0.574	0.444	0.478	0.255	0.214	0.112	0.073	0.253	0.201	0.624	0.631	0.733	0.373	0.861	0.426	1.126
F101_209.0304_17.1_N	0.435	0.380	0.224	0.431	0.246	0.253	0.264	0.360	0.692	0.352	0.261	0.325	0.398	0.271	0.423	0.231	0.480	0.458	0.351	0.342	0.425	0.293	0.186	0.589	0.290	0.245	0.257	0.616	0.217	0.310	0.411	0.383	0.418	0.401	0.410	0.233	0.365	0.586	0.231	0.178	0.309	0.241	0.020	0.149	0.137	0.020	0.276	0.133	0.252	0.092	0.118	0.123	0.179
F102_126.0661_9.2_P	0.024	0.029	0.022	0.045	0.050	0.056	0.020	0.057	0.018	0.021	0.021	0.021	0.028	0.029	0.028	0.021	0.024	0.017	0.010	0.013	0.023	0.025	0.026	0.046	0.020	0.056	0.028	0.023	0.043	0.039	0.028	0.032	0.022	0.025	0.028	0.060	0.031	0.024	0.042	0.046	0.053	0.046	0.064	0.036	0.047	0.059	0.127	0.187	0.075	0.095	0.083	0.110	0.058
F104_287.0519_13.3_N	0.007	0.007	0.008	0.006	0.008	0.008	0.010	0.004	0.006	0.008	0.006	0.009	0.006	0.012	0.010	0.008	0.010	0.006	0.013	0.007	0.003	0.007	0.008	0.006	0.007	0.006	0.007	0.006	0.016	0.007	0.007	0.009	0.007	0.008	0.008	0.010	0.007	0.009	0.006	0.006	0.008	0.007	0.009	0.006	0.007	0.008	0.008	0.010	0.009	0.031	0.015	0.011	0.008
F105_152.0563_13_P	0.068	0.062	0.086	0.075	0.236	0.182	0.040	0.139	0.080	0.087	0.100	0.154	0.127	0.060	0.184	0.133	0.237	0.084	0.086	0.109	0.146	0.104	0.086	0.132	0.189	0.123	0.319	0.228	0.258	0.274	0.141	0.199	0.140	0.141	0.312	0.224	0.212	0.228	0.407	0.348	0.328	0.317	0.486	0.270	0.533	0.431	0.440	0.380	0.350	0.594	0.364	0.695	0.278
F106_218.1383_9.3_P	0.191	0.061	0.191	0.069	0.388	0.321	0.085	0.187	0.166	0.406	0.340	0.477	0.152	0.164	0.318	0.336	0.281	0.083	0.182	0.133	0.141	0.132	0.126	0.119	0.355	0.205	0.600	0.310	0.439	0.443	0.184	0.136	0.261	0.140	0.182	0.455	0.366	0.151	0.374	0.670	0.561	0.269	0.454	0.724	0.313	0.387	0.708	0.676	0.583	0.422	0.511	0.973	0.464
F107_243.062_10_N	0.165	0.107	0.106	0.091	0.220	0.266	0.125	0.135	0.234	0.452	0.281	0.580	0.321	0.353	0.470	0.413	0.381	0.234	0.236	0.221	0.190	0.154	0.151	0.151	0.381	0.198	0.385	0.308	0.264	0.376	0.217	0.273	0.215	0.231	0.270	0.340	0.453	0.293	0.216	0.225	0.191	0.153	0.180	0.184	0.241	0.214	0.259	0.244	0.295	0.159	0.164	0.228	0.240
F109_186.087_10.4_P	0.075	0.013	0.030	0.021	0.059	0.063	0.006	0.038	0.029	0.069	0.035	0.255	0.038	0.039	0.068	0.103	0.075	0.036	0.039	0.042	0.017	0.042	0.055	0.028	0.044	0.028	0.089	0.064	0.128	0.087	0.035	0.026	0.022	0.041	0.062	0.086	0.072	0.039	0.062	0.121	0.051	0.046	0.033	0.056	0.066	0.049	0.031	0.044	0.048	0.034	0.052	0.027	0.049
F110_316.2478_4.5_P	0.003	0.004	0.009	0.005	0.012	0.010	0.005	0.009	0.012	0.009	0.012	0.020	0.010	0.015	0.019	0.014	0.012	0.002	0.006	0.006	0.013	0.010	0.011	0.009	0.015	0.010	0.013	0.010	0.014	0.018	0.011	0.011	0.012	0.007	0.010	0.017	0.007	0.012	0.004	0.003	0.004	0.006	0.008	0.009	0.003	0.007	0.017	0.005	0.019	0.013	0.018	0.041	0.003

F111_176.1027_16.2_P	0.980	0.585	1.439	1.113	1.664	1.927	0.693	1.851	1.585	0.943	1.863	1.307	1.825	0.595	1.636	1.719	1.410	1.067	1.118	1.245	1.774	1.653	1.384	1.959	1.646	1.409	2.289	1.327	2.491	1.467	1.633	2.269	2.554	2.040	2.301	1.980	1.737	1.378	2.278	2.438	2.500	1.393	2.853	2.282	2.065	2.511	1.603	1.261	1.261	1.584	2.394	2.355	0.723
F112_147.0661_9.1_N	0.027	0.022	0.037	0.024	0.032	0.034	0.017	0.025	0.020	0.012	0.019	0.038	0.016	0.014	0.025	0.033	0.027	0.020	0.022	0.020	0.010	0.036	0.029	0.037	0.019	0.037	0.040	0.020	0.065	0.021	0.015	0.016	0.013	0.017	0.018	0.041	0.027	0.016	0.058	0.058	0.055	0.047	0.045	0.062	0.053	0.088	0.012	0.010	0.010	0.024	0.033	0.010	0.004
F113_165.0403_12.9_N	0.068	0.066	0.062	0.051	0.047	0.070	0.049	0.067	0.055	0.040	0.076	0.060	0.059	0.118	0.066	0.069	0.096	0.063	0.051	0.049	0.074	0.086	0.079	0.076	0.053	0.047	0.065	0.059	0.105	0.068	0.081	0.078	0.051	0.060	0.044	0.105	0.087	0.073	0.068	0.074	0.085	0.048	0.079	0.088	0.068	0.089	0.057	0.062	0.059	0.084	0.105	0.059	0.046
F114_160.1329_8.7_P	1.138	0.395	0.727	0.633	1.769	1.833	0.585	1.360	0.793	1.421	1.498	1.181	1.407	0.624	1.392	1.867	0.907	0.888	0.552	1.398	1.114	1.817	1.402	1.429	1.487	1.542	3.172	2.265	4.118	1.908	1.489	1.065	1.015	1.349	1.179	2.891	1.318	1.537	2.107	3.186	3.022	1.897	3.161	3.166	2.946	3.505	3.674	4.224	2.581	3.857	4.793	3.408	1.537
F115_271.2279_3.4_N	2.015	2.972	4.121	2.969	4.853	4.368	1.903	4.006	3.771	2.330	3.975	3.487	4.942	1.302	4.360	3.639	2.899	1.988	1.428	3.059	3.494	3.644	3.599	4.462	3.806	3.684	3.270	3.007	3.016	2.949	2.958	2.440	2.890	2.496	1.765	2.961	2.199	2.848	2.188	2.061	1.550	1.010	2.274	2.544	1.972	2.339	2.153	2.206	2.044	2.733	2.392	2.737	1.435
F116_195.051_14.4_N	0.104	0.179	0.081	0.123	0.098	0.129	0.165	0.129	0.084	0.089	0.073	0.106	0.121	0.131	0.157	0.154	0.117	0.129	0.147	0.080	0.066	0.076	0.104	0.083	0.065	0.077	0.126	0.075	0.156	0.086	0.155	0.144	0.062	0.128	0.096	0.106	0.163	0.103	0.073	0.045	0.068	0.088	0.093	0.085	0.078	0.092	0.094	0.063	0.115	0.131	0.132	0.114	0.121
F117_136.0616_9.3_P	0.054	0.044	0.043	0.046	0.175	0.135	0.090	0.160	0.068	3.135	0.163	2.246	0.075	0.421	0.129	0.837	0.367	0.083	0.075	0.043	0.044	0.086	0.039	0.048	0.217	0.053	1.165	0.108	0.129	0.543	0.045	0.076	0.114	0.049	0.058	0.300	0.309	0.056	1.141	0.945	0.575	0.974	1.026	0.727	1.059	1.310	0.811	0.502	1.994	1.961	1.887	1.027	1.288
F118_301.217_3.4_N	2.915	2.698	3.767	3.767	5.398	4.532	2.230	5.696	4.096	2.460	4.178	3.638	5.159	1.154	4.857	3.627	3.312	2.087	1.632	3.004	4.206	3.376	3.418	4.769	4.082	5.051	4.078	3.177	4.503	3.962	3.882	2.687	3.031	2.692	2.282	3.726	2.153	3.498	3.115	3.417	2.948	2.509	4.733	3.113	3.807	4.171	2.657	2.967	2.469	3.559	3.113	2.708	1.594
F119_182.0457_4.5_N	0.003	0.004	0.006	0.002	0.004	0.006	0.003	0.003	0.002	0.004	0.001	0.017	0.004	0.022	0.005	0.008	0.005	0.002	0.003	0.004	0.003	0.003	0.004	0.004	0.007	0.004	0.008	0.016	0.005	0.005	0.008	0.006	0.003	0.005	0.005	0.005	0.004	0.005	0.004	0.007	0.003	0.002	0.003	0.004	0.003	0.006	0.007	0.004	0.007	0.009	0.016	0.004	0.004
F120_144.1016_10.5_P	3.794	3.287	5.374	5.039	6.496	5.932	3.945	7.704	6.106	5.339	7.112	5.888	7.305	2.464	5.887	5.812	5.581	6.204	4.465	5.500	7.400	6.009	9.059	5.339	6.089	5.227	6.152	5.096	6.026	6.300	6.277	6.841	7.809	6.677	5.521	6.552	5.988	6.153	4.010	3.673	5.325	4.285	6.292	4.400	4.470	5.491	6.501	6.373	7.490	8.739	6.245	7.981	4.084
F121_253.0928_9.5_P	0.063	0.031	0.042	0.081	0.094	0.094	0.035	0.092	0.026	0.037	0.028	0.037	0.046	0.060	0.044	0.045	0.038	0.039	0.014	0.023	0.041	0.039	0.038	0.090	0.038	0.086	0.044	0.111	0.061	0.060	0.051	0.058	0.037	0.047	0.046	0.092	0.061	0.054	0.053	0.070	0.083	0.071	0.081	0.042	0.068	0.071	0.225	0.279	0.127	0.153	0.118	0.175	0.102
F122_268.1035_8.8_P	0.114	0.207	0.153	0.091	0.114	0.104	0.083	0.271	0.061	0.068	0.059	0.161	0.122	0.042	0.174	0.165	0.063	0.069	0.062	0.035	0.055	0.104	0.052	0.212	0.052	0.214	0.118	0.141	0.131	0.171	0.067	0.099	0.119	0.044	0.066	0.146	0.134	0.081	0.153	0.154	0.137	0.104	0.211	0.225	0.110	0.171	0.091	0.087	0.093	0.159	0.080	0.105	0.083
F123_253.0925_8.6_P	0.002	0.005	0.001	0.002	0.002	0.001	0.005	0.001	0.001	0.002	0.001	0.001	0.001	0.025	0.001	0.001	0.001	0.002	0.003	0.001	0.001	0.001	0.001	0.001	0.001	0.001	0.002	0.001	0.001	0.001	0.004	0.007	0.003	0.006	0.007	0.009	0.005	0.003	0.003	0.003	0.007	0.002	0.006	0.002	0.002	0.004	0.022	0.023	0.034	0.035	0.043	0.026	0.014
F124_160.1329_13.1_P	0.052	0.027	0.070	0.037	0.082	0.096	0.037	0.089	0.046	0.063	0.081	0.089	0.065	0.037	0.074	0.123	0.075	0.055	0.043	0.041	0.060	0.067	0.079	0.054	0.048	0.071	0.080	0.058	0.096	0.093	0.053	0.058	0.056	0.073	0.075	0.101	0.073	0.049	0.063	0.081	0.099	0.072	0.094	0.086	0.063	0.092	0.090	0.092	0.088	0.098	0.100	0.183	0.038
F127_269.0876_11.1_P	17.78	19.46	18.09	19.07	21.98	19.43	18.92	20.08	17.49	13.80	15.83	18.03	18.09	15.88	16.44	16.08	21.67	15.64	17.21	21.42	19.03	14.16	15.60	19.68	18.10	18.53	23.28	20.60	21.87	24.79	20.05	17.12	20.80	19.14	18.52	19.93	23.97	26.30	29.02	27.02	25.91	29.86	33.18	21.13	33.59	28.84	26.37	24.49	28.65	29.41	26.22	26.16	28.93
F128_206.0821_4.5_N	0.000	0.001	0.000	0.000	0.002	0.002	0.001	0.002	0.004	0.002	0.000	0.004	0.002	0.003	0.003	0.002	0.002	0.001	0.001	0.001	0.005	0.002	0.002	0.002	0.002	0.001	0.002	0.001	0.003	0.004	0.003	0.002	0.002	0.002	0.004	0.002	0.003	0.001	0.003	0.003	0.018	0.001	0.003	0.004	0.012	0.005	0.003	0.002	0.004	0.004	0.003	0.003	0.001
F129_204.1227_10.6_P	2.111	0.950	1.180	0.923	1.855	1.856	1.884	1.877	1.599	1.995	2.674	2.944	1.339	1.648	2.584	3.526	1.709	1.500	1.883	2.011	1.519	2.652	1.657	1.357	1.467	2.256	2.837	1.808	2.921	3.917	1.805	1.835	3.607	3.421	3.583	3.819	2.202	1.916	0.800	0.860	2.314	2.651	1.704	2.403	1.143	1.926	3.957	4.724	4.316	3.415	4.293	12.67	2.114
F130_111.0199_7.9_N	0.005	0.010	0.003	0.005	0.005	0.005	0.016	0.006	0.004	0.006	0.003	0.004	0.004	0.059	0.004	0.004	0.005	0.006	0.008	0.003	0.005	0.004	0.003	0.004	0.004	0.003	0.008	0.005	0.008	0.007	0.007	0.012	0.009	0.009	0.010	0.014	0.014	0.013	0.014	0.014	0.031	0.014	0.026	0.007	0.015	0.016	0.054	0.042	0.071	0.078	0.083	0.056	0.035
F131_174.1234_25.3_P	0.017	0.007	0.017	0.017	0.027	0.030	0.006	0.024	0.020	0.012	0.020	0.020	0.026	0.034	0.025	0.023	0.017	0.023	0.012	0.009	0.016	0.030	0.023	0.024	0.030	0.020	0.029	0.018	0.043	0.022	0.030	0.048	0.032	0.044	0.039	0.043	0.041	0.015	0.009	0.010	0.012	0.008	0.010	0.009	0.008	0.010	0.022	0.015	0.017	0.035	0.045	0.015	0.006
F132_178.0528_8.5_P	0.029	0.008	0.015	0.010	0.016	0.023	0.006	0.023	0.021	0.011	0.026	0.035	0.020	0.028	0.024	0.039	0.028	0.029	0.017	0.029	0.055	0.053	0.027	0.017	0.028	0.011	0.023	0.013	0.028	0.026	0.037	0.038	0.037	0.021	0.062	0.034	0.021	0.041	0.003	0.003	0.002	0.002	0.001	0.007	0.006	0.002	0.025	0.015	0.024	0.045	0.034	0.017	0.006
F133_176.0915_13.7_P	0.017	0.011	0.012	0.010	0.027	0.023	0.007	0.040	0.009	0.019	0.018	0.061	0.019	0.108	0.018	0.056	0.062	0.016	0.016	0.009	0.026	0.022	0.042	0.012	0.021	0.008	0.037	0.017	0.056	0.043	0.017	0.026	0.024	0.026	0.041	0.038	0.069	0.020	0.025	0.030	0.030	0.013	0.019	0.027	0.043	0.021	0.029	0.030	0.046	0.046	0.044	0.025	0.036
F134_232.154_4_P	0.007	0.011	0.006	0.010	0.006	0.006	0.017	0.007	0.007	0.008	0.005	0.006	0.007	0.031	0.007	0.007	0.006	0.008	0.009	0.008	0.007	0.007	0.007	0.005	0.006	0.006	0.005	0.007	0.005	0.009	0.006	0.006	0.007	0.007	0.007	0.007	0.008	0.009	0.006	0.005	0.005	0.008	0.006	0.006	0.007	0.006	0.005	0.005	0.006	0.005	0.005	0.006	0.013
F136_191.0731_13.3_P	0.016	0.007	0.013	0.010	0.015	0.022	0.009	0.019	0.013	0.016	0.023	0.021	0.021	0.038	0.017	0.026	0.018	0.021	0.011	0.015	0.016	0.021	0.027	0.018	0.016	0.012	0.017	0.014	0.031	0.017	0.024	0.033	0.026	0.029	0.029	0.033	0.026	0.025	0.015	0.022	0.023	0.013	0.018	0.020	0.021	0.027	0.015	0.020	0.017	0.033	0.048	0.020	0.014
F137_212.0236_13_N	0.016	0.012	0.017	0.009	0.012	0.015	0.013	0.015	0.011	0.008	0.017	0.024	0.015	0.010	0.020	0.014	0.018	0.014	0.014	0.010	0.015	0.018	0.016	0.019	0.014	0.015	0.018	0.013	0.019	0.018	0.021	0.014	0.014	0.011	0.011	0.019	0.015	0.011	0.020	0.029	0.022	0.015	0.024	0.039	0.021	0.034	0.009	0.007	0.012	0.013	0.013	0.012	0.006
F138_372.3104_4.1_P	0.063	0.038	0.049	0.023	0.058	0.050	0.018	0.035	0.054	0.042	0.045	0.073	0.037	0.030	0.050	0.039	0.053	0.055	0.038	0.069	0.046	0.034	0.038	0.040	0.063	0.044	0.063	0.038	0.043	0.061	0.067	0.040	0.040	0.050	0.069	0.044	0.039	0.054	0.025	0.010	0.021	0.041	0.042	0.030	0.032	0.019	0.077	0.029	0.093	0.044	0.027	0.121	0.042
F139_540.33_4_N	0.074	0.075	0.082	0.081	0.082	0.068	0.054	0.077	0.079	0.049	0.049	0.064	0.060	0.027	0.061	0.035	0.053	0.051	0.036	0.065	0.054	0.061	0.058	0.071	0.063	0.077	0.060	0.063	0.068	0.052	0.041	0.041	0.034	0.035	0.042	0.051	0.043	0.044	0.045	0.032	0.042	0.056	0.055	0.039	0.061	0.042	0.047	0.066	0.051	0.043	0.027	0.051	0.030
F140_286.1476_9.8_P	0.057	0.033	0.081	0.058	0.088	0.074	0.040	0.120	0.076	0.057	0.094	0.103	0.111	0.081	0.124	0.121	0.110	0.066	0.071	0.101	0.152	0.142	0.110	0.143	0.119	0.123	0.137	0.117	0.118	0.125	0.154	0.126	0.152	0.099	0.143	0.139	0.144	0.137	0.089	0.070	0.108	0.088	0.141	0.097	0.129	0.149	0.119	0.142	0.130	0.128	0.181	0.177	0.067
F141_195.1129_4.5_P	0.002	0.011	0.061	0.070	0.007	0.019	0.005	0.012	0.060	0.063	0.049	0.051	0.013	0.054	0.016	0.014	0.037	0.002	0.002	0.066	0.011	0.014	0.041	0.022	0.046	0.025	0.032	0.045	0.016	0.026	0.025	0.019	0.011	0.016	0.035	0.060	0.033	0.039	0.034	0.047	0.013	0.002	0.007	0.038	0.034	0.009	0.047	0.021	0.038	0.049	0.018	0.014	0.006
F142_374.253_4.5_P	0.002	0.004	0.014	0.003	0.013	0.017	0.006	0.017	0.010	0.003	0.015	0.028	0.017	0.015	0.017	0.021	0.025	0.003	0.003	0.002	0.015	0.022	0.021	0.013	0.015	0.016	0.015	0.015	0.019	0.019	0.033	0.016	0.020	0.018	0.022	0.029	0.022	0.026	0.020	0.022	0.021	0.003	0.027	0.025	0.034	0.036	0.021	0.023	0.027	0.039	0.045	0.035	0.004
F143_262.1646_10.1_P	0.019	0.018	0.036	0.031	0.033	0.055	0.028	0.036	0.032	0.023	0.021	0.056	0.038	0.107	0.050	0.034	0.036	0.036	0.027	0.028	0.019	0.025	0.036	0.046	0.034	0.029	0.058	0.040	0.028	0.026	0.037	0.040	0.030	0.030	0.034	0.032	0.073	0.021	0.056	0.048	0.038	0.028	0.033	0.073	0.046	0.022	0.036	0.037	0.049	0.064	0.058	0.039	0.033
F144_492.3083_4.1_P	0.078	0.065	0.063	0.047	0.060	0.042	0.059	0.046	0.064	0.035	0.040	0.051	0.011	0.018	0.033	0.027	0.043	0.041	0.042	0.049	0.043	0.038	0.053	0.052	0.049	0.049	0.032	0.033	0.044	0.026	0.032	0.023	0.024	0.027	0.035	0.037	0.024	0.027	0.052	0.043	0.036	0.063	0.048	0.041	0.039	0.043	0.027	0.041	0.028	0.037	0.016	0.025	0.018
F146_301.1386_8.4_P	0.028	0.026	0.062	0.033	0.047	0.050	0.041	0.071	0.044	0.020	0.040	0.030	0.038	0.034	0.036	0.057	0.048	0.022	0.044	0.052	0.059	0.055	0.033	0.047	0.034	0.038	0.049	0.048	0.042	0.042	0.033	0.016	0.051	0.037	0.037	0.029	0.036	0.038	0.022	0.017	0.052	0.031	0.045	0.020	0.041	0.030	0.037	0.032	0.037	0.026	0.042	0.059	0.022
F147_166.0143_16.5_N	0.006	0.002	0.006	0.006	0.011	0.011	0.005	0.009	0.006	0.006	0.007	0.007	0.006	0.015	0.010	0.008	0.012	0.006	0.006	0.007	0.007	0.007	0.006	0.010	0.009	0.006	0.008	0.006	0.016	0.009	0.012	0.010	0.010	0.007	0.010	0.013	0.012	0.012	0.008	0.009	0.014	0.008	0.010	0.009	0.018	0.017	0.017	0.014	0.016	0.025	0.036	0.022	0.012
F148_237.1328_8.5_P	0.029	0.008	0.016	0.013	0.014	0.026	0.008	0.010	0.015	0.010	0.006	0.061	0.008	0.036	0.016	0.014	0.009	0.014	0.014	0.017	0.004	0.006	0.010	0.011	0.011	0.010	0.014	0.015	0.019	0.012	0.008	0.008	0.008	0.007	0.007	0.015	0.020	0.006	0.012	0.018	0.008	0.012	0.010	0.016	0.014	0.010	0.007	0.003	0.008	0.013	0.017	0.004	0.006
F149_559.473_3.1_N	0.014	0.006	0.017	0.018	0.022	0.016	0.006	0.011	0.027	0.018	0.016	0.035	0.018	0.009	0.029	0.020	0.017	0.017	0.011	0.011	0.012	0.020	0.016	0.028	0.039	0.024	0.045	0.031	0.033	0.015	0.016	0.038	0.020	0.018	0.013	0.026	0.029	0.014	0.018	0.023	0.011	0.006	0.017	0.013	0.012	0.011	0.012	0.017	0.008	0.013	0.014	0.015	0.011
F150_413.1676_10_N	0.059	0.051	0.028	0.033	0.023	0.029	0.052	0.018	0.052	0.119	0.041	0.059	0.041	0.125	0.050	0.037	0.038	0.072	0.087	0.050	0.027	0.020	0.022	0.015	0.046	0.023	0.026	0.042	0.018	0.047	0.035	0.038	0.027	0.039	0.055	0.037	0.047	0.054	0.025	0.021	0.022	0.047	0.015	0.018	0.024	0.015	0.039	0.024	0.051	0.024	0.012	0.020	0.088
F152_525.3249_3.1_N	0.012	0.007	0.019	0.030	0.016	0.014	0.007	0.030	0.024	0.023	0.021	0.013	0.034	0.010	0.019	0.048	0.023	0.019	0.015	0.033	0.061	0.016	0.034	0.043	0.024	0.019	0.024	0.028	0.013	0.025	0.029	0.025	0.043	0.027	0.031	0.018	0.038	0.041	0.012	0.014	0.025	0.018	0.016	0.034	0.017	0.024	0.034	0.022	0.037	0.020	0.017	0.019	0.010
F153_313.2383_3.6_N	0.017	0.017	0.017	0.023	0.035	0.034	0.017	0.037	0.028	0.020	0.031	0.023	0.030	0.017	0.025	0.028	0.022	0.020	0.016	0.023	0.027	0.028	0.027	0.039	0.024	0.026	0.027	0.023	0.033	0.019	0.028	0.020	0.015	0.014	0.015	0.022	0.022	0.022	0.029	0.037	0.031	0.028	0.042	0.038	0.043	0.062	0.027	0.030	0.020	0.022	0.019	0.026	0.019
F154_529.2972_4.1_N	0.044	0.021	0.040	0.045	0.051	0.028	0.012	0.043	0.047	0.027	0.033	0.032	0.030	0.014	0.038	0.020	0.039	0.027	0.031	0.040	0.040	0.038	0.038	0.033	0.037	0.041	0.025	0.030	0.036	0.027	0.033	0.024	0.027	0.022	0.027	0.027	0.019	0.022	0.037	0.018	0.029	0.039	0.038	0.023	0.033	0.027	0.034	0.033	0.032	0.026	0.026	0.031	0.015
F155_147.0661_7.4_N	0.021	0.021	0.027	0.018	0.024	0.044	0.016	0.030	0.020	0.013	0.016	0.034	0.021	0.014	0.021	0.030	0.039	0.022	0.014	0.021	0.014	0.016	0.018	0.036	0.016	0.021	0.019	0.021	0.045	0.021	0.023	0.026	0.017	0.019	0.015	0.033	0.031	0.020	0.030	0.040	0.041	0.028	0.038	0.032	0.023	0.039	0.016	0.015	0.015	0.028	0.026	0.012	0.009
F157_246.1082_12.4_P	0.038	0.029	0.039	0.048	0.029	0.061	0.026	0.052	0.041	0.031	0.028	0.043	0.051	0.052	0.052	0.057	0.064	0.057	0.030	0.035	0.028	0.037	0.054	0.043	0.035	0.020	0.029	0.032	0.045	0.064	0.036	0.065	0.026	0.044	0.042	0.068	0.081	0.091	0.040	0.020	0.028	0.036	0.034	0.028	0.043	0.022	0.050	0.036	0.039	0.057	0.041	0.044	0.045
F158_192.1226_11.4_P	0.019	0.021	0.025	0.022	0.020	0.038	0.017	0.032	0.016	0.009	0.017	0.030	0.022	0.041	0.022	0.028	0.027	0.025	0.025	0.022	0.017	0.024	0.028	0.028	0.016	0.024	0.022	0.016	0.033	0.029	0.020	0.028	0.015	0.022	0.027	0.040	0.032	0.022	0.028	0.041	0.041	0.038	0.048	0.040	0.026	0.035	0.023	0.022	0.022	0.035	0.043	0.015	0.012
F159_285.1258_4.1_N	0.025	0.012	0.029	0.022	0.028	0.041	0.008	0.038	0.036	0.024	0.038	0.036	0.045	0.011	0.031	0.038	0.053	0.030	0.036	0.032	0.053	0.059	0.039	0.032	0.033	0.030	0.040	0.027	0.039	0.063	0.059	0.038	0.046	0.071	0.039	0.028	0.053	0.078	0.041	0.030	0.036	0.021	0.030	0.023	0.038	0.031	0.042	0.047	0.042	0.039	0.029	0.039	0.017
F160_245.0957_12.1_P	0.052	0.030	0.045	0.044	0.084	0.089	0.011	0.059	0.059	0.083	0.087	0.083	0.071	0.068	0.097	0.083	0.080	0.053	0.035	0.054	0.041	0.055	0.050	0.060	0.078	0.060	0.095	0.039	0.110	0.121	0.069	0.075	0.069	0.080	0.087	0.120	0.101	0.090	0.106	0.096	0.082	0.073	0.092	0.089	0.154	0.113	0.151	0.112	0.188	0.134	0.195	0.157	0.144
F162_110.9757_9.4_N	0.016	0.018	0.020	0.021	0.038	0.029	0.049	0.053	0.041	0.025	0.019	0.017	0.063	0.014	0.035	0.020	0.026	0.026	0.014	0.022	0.033	0.043	0.024	0.039	0.020	0.036	0.025	0.027	0.020	0.032	0.022	0.021	0.023	0.017	0.023	0.028	0.026	0.021	0.012	0.016	0.019	0.015	0.023	0.013	0.021	0.033	0.030	0.045	0.020	0.020	0.020	0.033	0.011
F163_254.1593_8.6_P	0.038	0.017	0.025	0.017	0.029	0.055	0.014	0.021	0.023	0.016	0.012	0.077	0.016	0.020	0.031	0.026	0.019	0.020	0.019	0.024	0.009	0.015	0.018	0.021	0.023	0.020	0.034	0.024	0.040	0.021	0.016	0.021	0.014	0.012	0.011	0.027	0.032	0.010	0.023	0.036	0.017	0.018	0.018	0.026	0.020	0.019	0.012	0.008	0.014	0.018	0.035	0.010	0.008
F164_542.3094_4.1_N	0.030	0.016	0.037	0.030	0.033	0.027	0.012	0.035	0.041	0.024	0.029	0.028	0.027	0.014	0.022	0.022	0.026	0.036	0.029	0.040	0.033	0.035	0.033	0.031	0.032	0.032	0.025	0.017	0.037	0.028	0.024	0.022	0.025	0.023	0.032	0.023	0.020	0.020	0.028	0.020	0.029	0.039	0.036	0.020	0.022	0.020	0.029	0.025	0.047	0.028	0.021	0.027	0.015
F165_812.5069_3.4_N	0.089	0.145	0.030	0.077	0.007	0.025	0.140	0.031	0.050	0.084	0.049	0.071	0.050	0.288	0.039	0.040	0.063	0.120	0.119	0.077	0.061	0.027	0.080	0.027	0.056	0.049	0.042	0.066	0.038	0.078	0.088	0.061	0.057	0.058	0.087	0.026	0.065	0.091	0.041	0.054	0.064	0.082	0.030	0.031	0.043	0.044	0.056	0.047	0.061	0.044	0.042	0.048	0.050
F167_289.1754_9.5_P	0.009	0.005	0.009	0.006	0.010	0.020	0.005	0.011	0.008	0.007	0.009	0.020	0.007	0.021	0.007	0.023	0.049	0.011	0.021	0.016	0.004	0.009	0.022	0.011	0.005	0.007	0.010	0.010	0.044	0.010	0.015	0.008	0.004	0.006	0.003	0.018	0.011	0.012	0.017	0.015	0.021	0.009	0.022	0.020	0.012	0.013	0.012	0.008	0.011	0.037	0.032	0.010	0.005
F168_276.191_14.3_P	0.012	0.008	0.021	0.009	0.020	0.027	0.007	0.039	0.022	0.017	0.019	0.024	0.033	0.032	0.032	0.031	0.043	0.025	0.010	0.019	0.023	0.029	0.029	0.032	0.020	0.024	0.031	0.027	0.034	0.046	0.038	0.033	0.024	0.027	0.029	0.035	0.041	0.041	0.029	0.017	0.036	0.022	0.028	0.017	0.052	0.028	0.057	0.053	0.055	0.044	0.042	0.058	0.021

F169_502.157_10.9_P	0.214	0.635	0.061	0.231	0.022	0.026	0.006	0.023	0.076	0.179	0.041	0.060	0.038	0.018	0.044	0.028	0.042	0.172	0.110	0.087	0.037	0.029	0.035	0.020	0.041	0.024	0.019	0.050	0.016	0.044	0.052	0.042	0.047	0.064	0.084	0.038	0.051	0.091	0.029	0.021	0.023	0.145	0.028	0.029	0.038	0.018	0.054	0.026	0.068	0.037	0.023	0.029	0.130
F170_140.0815_7.4_P	0.001	0.003	0.006	0.005	0.006	0.013	0.006	0.007	0.003	0.002	0.003	0.007	0.004	0.011	0.005	0.005	0.008	0.004	0.002	0.004	0.003	0.005	0.007	0.006	0.004	0.005	0.007	0.004	0.008	0.005	0.008	0.005	0.004	0.006	0.004	0.008	0.007	0.004	0.023	0.022	0.028	0.003	0.023	0.030	0.039	0.028	0.004	0.004	0.004	0.008	0.011	0.005	0.002
F171_124.9913_7.3_N	0.015	0.008	0.024	0.018	0.041	0.036	0.010	0.069	0.031	0.036	0.024	0.021	0.037	0.007	0.038	0.027	0.040	0.031	0.015	0.027	0.043	0.043	0.034	0.046	0.026	0.034	0.031	0.033	0.026	0.033	0.030	0.030	0.031	0.027	0.023	0.039	0.044	0.031	0.018	0.018	0.018	0.014	0.022	0.017	0.022	0.022	0.031	0.040	0.019	0.022	0.020	0.032	0.010
F172_304.0704_16.1_N	0.003	0.007	0.033	0.005	0.027	0.030	0.024	0.039	0.042	0.002	0.056	0.032	0.037	0.011	0.028	0.044	0.042	0.002	0.052	0.055	0.053	0.058	0.075	0.029	0.067	0.031	0.034	0.047	0.044	0.042	0.070	0.051	0.064	0.069	0.048	0.039	0.035	0.005	0.021	0.018	0.021	0.001	0.028	0.017	0.026	0.027	0.038	0.036	0.040	0.030	0.029	0.046	0.005
F174_417.1331_11.5_P	0.041	0.054	0.067	0.040	0.014	0.027	0.029	0.018	0.029	0.063	0.064	0.059	0.032	0.025	0.056	0.036	0.065	0.039	0.029	0.027	0.034	0.021	0.042	0.031	0.039	0.047	0.019	0.068	0.017	0.025	0.031	0.019	0.018	0.033	0.019	0.032	0.024	0.046	0.019	0.020	0.024	0.024	0.024	0.024	0.024	0.020	0.029	0.019	0.022	0.022	0.015	0.015	0.018
F175_340.1033_8.3_N	0.008	0.003	0.006	0.001	0.019	0.018	0.003	0.015	0.010	0.028	0.008	0.030	0.008	0.015	0.020	0.023	0.032	0.001	0.008	0.007	0.013	0.002	0.003	0.008	0.020	0.017	0.040	0.024	0.051	0.035	0.005	0.002	0.005	0.004	0.009	0.019	0.018	0.012	0.011	0.015	0.014	0.007	0.004	0.006	0.014	0.006	0.025	0.014	0.016	0.007	0.005	0.008	0.010
F176_192.1225_9.8_P	0.020	0.021	0.022	0.016	0.027	0.032	0.009	0.025	0.017	0.022	0.023	0.020	0.030	0.050	0.023	0.028	0.019	0.018	0.015	0.026	0.020	0.028	0.021	0.025	0.025	0.025	0.071	0.033	0.039	0.026	0.018	0.024	0.016	0.018	0.018	0.040	0.028	0.027	0.042	0.043	0.043	0.032	0.053	0.045	0.041	0.054	0.053	0.055	0.042	0.049	0.044	0.033	0.031
F178_140.9862_12.9_N	0.026	0.040	0.014	0.004	0.004	0.004	0.034	0.018	0.010	0.010	0.045	0.005	0.008	0.065	0.006	0.009	0.042	0.017	0.013	0.010	0.054	0.032	0.053	0.011	0.011	0.009	0.010	0.007	0.023	0.013	0.024	0.010	0.027	0.019	0.010	0.019	0.008	0.007	0.010	0.010	0.007	0.003	0.011	0.013	0.011	0.016	0.008	0.009	0.024	0.004	0.012	0.005	0.008
F180_326.1089_15.3_N	0.041	0.020	0.027	0.034	0.033	0.022	0.017	0.019	0.029	0.123	0.035	0.037	0.018	0.012	0.024	0.032	0.031	0.030	0.050	0.023	0.020	0.020	0.028	0.020	0.050	0.020	0.031	0.040	0.046	0.042	0.026	0.022	0.022	0.045	0.031	0.031	0.026	0.026	0.016	0.018	0.025	0.037	0.015	0.012	0.017	0.018	0.031	0.032	0.027	0.015	0.016	0.021	0.074
F181_576.1929_16.4_P	0.012	0.005	0.019	0.018	0.023	0.021	0.010	0.012	0.014	0.042	0.020	0.018	0.009	0.025	0.015	0.023	0.017	0.013	0.009	0.011	0.007	0.019	0.020	0.043	0.012	0.060	0.015	0.040	0.010	0.011	0.010	0.018	0.009	0.011	0.008	0.014	0.011	0.010	0.013	0.008	0.030	0.016	0.008	0.008	0.008	0.010	0.024	0.044	0.011	0.006	0.009	0.011	0.011
F182_155.0924_9.7_P	0.006	0.008	0.004	0.003	0.003	0.024	0.007	0.003	0.004	0.002	0.001	0.011	0.002	0.033	0.001	0.010	0.023	0.002	0.003	0.002	0.001	0.004	0.009	0.003	0.001	0.004	0.004	0.002	0.016	0.006	0.002	0.002	0.001	0.002	0.002	0.008	0.002	0.002	0.055	0.057	0.079	0.050	0.058	0.053	0.081	0.066	0.002	0.002	0.002	0.004	0.017	0.002	0.005
F183_364.0919_14.5_N	0.017	0.003	0.017	0.018	0.012	0.012	0.005	0.013	0.014	0.014	0.013	0.010	0.006	0.015	0.010	0.009	0.011	0.012	0.013	0.013	0.005	0.009	0.011	0.023	0.015	0.022	0.007	0.025	0.007	0.012	0.017	0.013	0.009	0.011	0.015	0.013	0.009	0.013	0.009	0.007	0.020	0.020	0.008	0.008	0.023	0.012	0.032	0.062	0.022	0.011	0.010	0.016	0.027
F184_559.1505_11_P	0.214	0.220	0.081	0.207	0.035	0.037	0.005	0.044	0.096	0.006	0.068	0.094	0.059	0.019	0.064	0.044	0.062	0.020	0.197	0.130	0.047	0.048	0.051	0.042	0.062	0.037	0.026	0.079	0.034	0.069	0.055	0.062	0.058	0.123	0.083	0.051	0.077	0.128	0.032	0.030	0.029	0.310	0.035	0.037	0.053	0.024	0.072	0.034	0.074	0.052	0.039	0.042	0.019
F185_277.1443_4_N	0.020	0.010	0.018	0.010	0.014	0.014	0.012	0.022	0.023	0.015	0.020	0.024	0.031	0.012	0.024	0.039	0.029	0.020	0.021	0.015	0.033	0.023	0.022	0.027	0.020	0.024	0.024	0.027	0.038	0.022	0.048	0.028	0.068	0.040	0.053	0.041	0.053	0.063	0.026	0.033	0.024	0.021	0.034	0.033	0.028	0.014	0.042	0.050	0.072	0.062	0.045	0.025	0.044
F186_262.128_13_P	0.016	0.012	0.021	0.017	0.020	0.029	0.015	0.026	0.016	0.031	0.017	0.039	0.021	0.033	0.033	0.026	0.019	0.028	0.015	0.015	0.008	0.020	0.016	0.036	0.022	0.023	0.022	0.027	0.030	0.050	0.018	0.035	0.017	0.023	0.023	0.030	0.043	0.023	0.028	0.028	0.038	0.028	0.036	0.029	0.030	0.023	0.052	0.080	0.044	0.049	0.039	0.065	0.034
F188_146.1172_9.5_P	0.018	0.026	0.024	0.028	0.027	0.051	0.014	0.030	0.032	0.020	0.036	0.042	0.029	0.036	0.029	0.025	0.021	0.025	0.027	0.022	0.031	0.028	0.037	0.025	0.020	0.025	0.056	0.034	0.040	0.015	0.030	0.039	0.021	0.021	0.023	0.034	0.046	0.057	0.038	0.027	0.035	0.025	0.026	0.033	0.034	0.028	0.036	0.017	0.044	0.027	0.026	0.013	0.024
F189_249.1859_3.5_N	0.029	0.036	0.054	0.039	0.079	0.061	0.017	0.052	0.042	0.031	0.054	0.050	0.043	0.015	0.047	0.036	0.028	0.021	0.012	0.029	0.029	0.035	0.043	0.056	0.044	0.039	0.037	0.030	0.035	0.035	0.025	0.022	0.019	0.023	0.013	0.028	0.015	0.023	0.039	0.038	0.031	0.018	0.046	0.042	0.039	0.051	0.018	0.025	0.018	0.027	0.021	0.032	0.011
F191_320.0791_9_P	0.019	0.007	0.025	0.016	0.019	0.022	0.007	0.015	0.017	0.008	0.011	0.020	0.012	0.022	0.013	0.020	0.017	0.018	0.014	0.020	0.007	0.011	0.016	0.019	0.007	0.016	0.012	0.014	0.017	0.016	0.011	0.015	0.008	0.011	0.009	0.020	0.020	0.011	0.022	0.027	0.023	0.017	0.029	0.041	0.010	0.022	0.009	0.007	0.005	0.015	0.010	0.005	0.004
F192_319.2279_3.4_N	0.032	0.046	0.033	0.043	0.038	0.038	0.032	0.040	0.042	0.030	0.027	0.034	0.036	0.074	0.032	0.023	0.025	0.030	0.025	0.024	0.023	0.021	0.021	0.037	0.032	0.038	0.030	0.031	0.030	0.025	0.024	0.020	0.024	0.021	0.015	0.029	0.019	0.026	0.088	0.097	0.058	0.112	0.077	0.113	0.056	0.060	0.040	0.056	0.035	0.034	0.014	0.031	0.059
F193_217.1179_11.7_P	0.030	0.023	0.048	0.027	0.015	0.028	0.014	0.021	0.033	0.014	0.043	0.031	0.047	0.062	0.055	0.033	0.032	0.037	0.037	0.026	0.045	0.042	0.047	0.032	0.039	0.024	0.032	0.031	0.081	0.039	0.070	0.046	0.064	0.040	0.066	0.071	0.047	0.040	0.019	0.013	0.022	0.019	0.016	0.029	0.018	0.029	0.020	0.007	0.038	0.045	0.040	0.016	0.009
F194_360.1046_13_N	0.015	0.004	0.025	0.017	0.014	0.007	0.004	0.007	0.010	0.029	0.024	0.009	0.004	0.018	0.009	0.028	0.028	0.010	0.016	0.012	0.006	0.036	0.013	0.046	0.008	0.062	0.006	0.007	0.010	0.007	0.006	0.017	0.008	0.029	0.021	0.018	0.015	0.007	0.004	0.006	0.019	0.005	0.005	0.002	0.004	0.014	0.020	0.031	0.010	0.006	0.028	0.013	0.004
F196_185.0124_9.1_N	0.001	0.002	0.000	0.001	0.000	0.000	0.002	0.000	0.002	0.001	0.000	0.000	0.000	0.011	0.000	0.000	0.000	0.001	0.001	0.000	0.000	0.000	0.000	0.000	0.000	0.000	0.001	0.001	0.000	0.000	0.000	0.000	0.001	0.001	0.001	0.000	0.001	0.001	0.000	0.000	0.000	0.001	0.000	0.000	0.000	0.000	0.000	0.000	0.001	0.001	0.000	0.000	0.002
F197_558.3311_4_N	0.059	0.072	0.086	0.087	0.082	0.059	0.086	0.062	0.087	0.053	0.046	0.072	0.059	0.023	0.037	0.042	0.060	0.036	0.041	0.073	0.059	0.056	0.048	0.073	0.065	0.063	0.067	0.070	0.065	0.041	0.049	0.046	0.050	0.038	0.047	0.051	0.042	0.034	0.065	0.057	0.047	0.061	0.053	0.046	0.050	0.044	0.051	0.054	0.053	0.047	0.041	0.043	0.039
F198_610.4307_3.1_P	0.027	0.053	0.022	0.048	0.025	0.027	0.085	0.029	0.029	0.033	0.018	0.034	0.035	0.023	0.036	0.028	0.023	0.036	0.016	0.031	0.021	0.017	0.021	0.054	0.019	0.027	0.019	0.027	0.015	0.036	0.035	0.024	0.023	0.012	0.019	0.017	0.035	0.024	0.019	0.031	0.015	0.012	0.016	0.057	0.013	0.016	0.018	0.018	0.020	0.015	0.015	0.013	0.036
F199_273.186_3.4_N	0.025	0.028	0.043	0.034	0.062	0.047	0.016	0.043	0.039	0.022	0.031	0.060	0.045	0.016	0.041	0.027	0.023	0.020	0.011	0.021	0.029	0.028	0.027	0.047	0.034	0.052	0.041	0.031	0.036	0.031	0.031	0.035	0.023	0.021	0.013	0.029	0.022	0.020	0.038	0.041	0.029	0.022	0.040	0.044	0.026	0.033	0.020	0.023	0.017	0.025	0.021	0.025	0.008
F201_242.0992_8.8_P	0.037	0.012	0.033	0.023	0.021	0.018	0.017	0.017	0.025	0.043	0.027	0.056	0.020	0.018	0.025	0.025	0.020	0.031	0.021	0.035	0.009	0.010	0.018	0.023	0.028	0.024	0.020	0.054	0.039	0.024	0.020	0.019	0.013	0.027	0.043	0.037	0.028	0.020	0.042	0.041	0.037	0.060	0.040	0.059	0.050	0.042	0.055	0.047	0.079	0.066	0.066	0.044	0.038
F202_453.2816_3.9_N	0.079	0.110	0.110	0.148	0.008	0.079	0.058	0.120	0.097	0.006	0.050	0.102	0.083	0.026	0.046	0.036	0.066	0.009	0.060	0.092	0.071	0.094	0.075	0.076	0.081	0.098	0.055	0.061	0.052	0.049	0.038	0.049	0.048	0.048	0.033	0.059	0.035	0.047	0.091	0.044	0.044	0.011	0.062	0.054	0.065	0.051	0.045	0.067	0.057	0.045	0.034	0.053	0.064
F203_459.348_3.2_N	0.024	0.015	0.013	0.031	0.026	0.018	0.014	0.046	0.039	0.018	0.022	0.019	0.028	0.012	0.035	0.058	0.028	0.025	0.011	0.008	0.045	0.014	0.012	0.055	0.023	0.032	0.029	0.050	0.030	0.024	0.031	0.048	0.026	0.015	0.030	0.025	0.041	0.039	0.016	0.017	0.010	0.008	0.015	0.012	0.006	0.005	0.026	0.026	0.049	0.025	0.025	0.013	0.022
F205_226.9859_15.4_N	0.050	0.013	0.063	0.046	0.043	0.054	0.009	0.063	0.089	0.051	0.060	0.054	0.086	0.014	0.069	0.051	0.062	0.053	0.039	0.057	0.072	0.071	0.054	0.059	0.066	0.063	0.060	0.061	0.036	0.059	0.083	0.078	0.082	0.062	0.075	0.079	0.068	0.070	0.051	0.052	0.059	0.030	0.038	0.028	0.049	0.046	0.047	0.033	0.060	0.042	0.056	0.042	0.020
F206_176.0914_9_P	0.048	0.019	0.019	0.010	0.009	0.049	0.015	0.015	0.007	0.012	0.019	0.106	0.011	0.032	0.008	0.037	0.067	0.027	0.070	0.035	0.003	0.022	0.059	0.013	0.009	0.011	0.016	0.021	0.065	0.020	0.021	0.009	0.006	0.019	0.018	0.023	0.015	0.014	0.036	0.035	0.042	0.022	0.024	0.014	0.032	0.043	0.016	0.009	0.010	0.043	0.050	0.007	0.007
F207_330.2516_3.3_N	0.063	0.084	0.097	0.085	0.121	0.103	0.055	0.091	0.094	0.062	0.124	0.088	0.129	0.016	0.101	0.092	0.071	0.045	0.038	0.077	0.087	0.080	0.066	0.126	0.111	0.111	0.096	0.065	0.105	0.072	0.110	0.040	0.081	0.056	0.025	0.065	0.047	0.050	0.073	0.072	0.055	0.039	0.084	0.078	0.061	0.087	0.050	0.061	0.052	0.076	0.067	0.050	0.025
F208_87.0451_7.5_N	0.007	0.079	0.011	0.006	0.021	0.030	0.087	0.048	0.016	0.016	0.020	0.022	0.038	0.388	0.021	0.029	0.025	0.011	0.006	0.018	0.017	0.019	0.016	0.026	0.017	0.026	0.023	0.009	0.017	0.023	0.036	0.024	0.018	0.037	0.044	0.013	0.032	0.025	0.027	0.052	0.041	0.022	0.067	0.026	0.040	0.035	0.045	0.052	0.060	0.046	0.037	0.045	0.023
F209_200.054_10.3_P	0.056	0.042	0.063	0.058	0.055	0.057	0.061	0.048	0.057	0.061	0.039	0.049	0.027	0.079	0.031	0.045	0.043	0.043	0.060	0.048	0.033	0.024	0.032	0.044	0.049	0.041	0.042	0.067	0.036	0.051	0.042	0.034	0.038	0.048	0.071	0.059	0.061	0.067	0.095	0.071	0.075	0.143	0.105	0.078	0.118	0.091	0.097	0.082	0.166	0.144	0.093	0.073	0.183
F210_261.1074_15.9_P	0.036	0.026	0.049	0.033	0.047	0.036	0.022	0.057	0.043	0.031	0.056	0.061	0.057	0.027	0.058	0.065	0.074	0.036	0.038	0.054	0.064	0.078	0.055	0.070	0.051	0.057	0.068	0.044	0.061	0.058	0.077	0.061	0.072	0.045	0.060	0.062	0.074	0.057	0.047	0.046	0.054	0.041	0.075	0.051	0.044	0.054	0.043	0.042	0.055	0.055	0.067	0.057	0.025
F211_287.1267_11.4_P	0.053	0.100	0.096	0.066	0.028	0.037	0.045	0.034	0.041	0.099	0.077	0.074	0.045	0.030	0.088	0.046	0.100	0.053	0.040	0.041	0.040	0.025	0.072	0.048	0.047	0.085	0.035	0.123	0.040	0.042	0.033	0.019	0.018	0.046	0.022	0.046	0.033	0.091	0.008	0.006	0.009	0.027	0.015	0.006	0.016	0.008	0.048	0.030	0.022	0.023	0.012	0.021	0.031
F212_134.0805_12.7_P	0.094	0.083	0.076	0.114	0.037	0.061	0.113	0.098	0.083	0.095	0.062	0.075	0.067	0.091	0.049	0.104	0.069	0.143	0.101	0.112	0.048	0.067	0.114	0.079	0.065	0.052	0.047	0.072	0.059	0.138	0.102	0.097	0.047	0.101	0.083	0.076	0.094	0.171	0.019	0.025	0.042	0.058	0.027	0.024	0.035	0.033	0.067	0.033	0.092	0.111	0.039	0.084	0.091
F214_346.2218_8.5_P	0.019	0.020	0.030	0.012	0.027	0.040	0.007	0.031	0.025	0.019	0.020	0.055	0.028	0.042	0.039	0.042	0.043	0.023	0.022	0.025	0.018	0.025	0.023	0.027	0.023	0.019	0.034	0.032	0.028	0.028	0.043	0.028	0.018	0.025	0.019	0.037	0.027	0.043	0.029	0.025	0.032	0.021	0.028	0.025	0.043	0.036	0.050	0.017	0.049	0.055	0.070	0.055	0.024
F215_250.0928_7.4_P	0.001	0.004	0.009	0.013	0.026	0.016	0.031	0.017	0.006	0.051	0.010	0.039	0.006	0.020	0.011	0.035	0.011	0.010	0.002	0.004	0.002	0.007	0.008	0.021	0.010	0.023	0.026	0.096	0.015	0.019	0.010	0.009	0.009	0.009	0.009	0.021	0.017	0.006	0.046	0.047	0.029	0.002	0.057	0.049	0.052	0.065	0.032	0.032	0.049	0.084	0.062	0.030	0.003
F216_176.1278_13.8_P	0.030	0.007	0.035	0.015	0.030	0.048	0.011	0.034	0.017	0.023	0.033	0.043	0.037	0.031	0.031	0.054	0.048	0.026	0.033	0.018	0.023	0.035	0.044	0.018	0.026	0.028	0.049	0.025	0.066	0.039	0.032	0.031	0.033	0.055	0.054	0.044	0.045	0.024	0.047	0.064	0.068	0.044	0.052	0.040	0.029	0.044	0.041	0.044	0.040	0.060	0.072	0.046	0.026
F218_59.073_10.7_P	0.111	0.122	0.107	0.106	0.141	0.189	0.110	0.113	0.113	0.160	0.150	0.152	0.146	0.041	0.141	0.138	0.126	0.113	0.130	0.094	0.097	0.074	0.098	0.132	0.149	0.135	0.156	0.122	0.100	0.141	0.103	0.090	0.088	0.123	0.068	0.121	0.153	0.083	0.176	0.149	0.122	0.092	0.122	0.109	0.103	0.080	0.064	0.090	0.061	0.069	0.064	0.061	0.105
F219_499.0508_15.9_N	0.055	0.007	0.051	0.034	0.057	0.062	0.008	0.059	0.062	0.072	0.058	0.054	0.053	0.034	0.058	0.065	0.064	0.043	0.048	0.067	0.059	0.042	0.044	0.056	0.056	0.055	0.063	0.077	0.081	0.069	0.050	0.058	0.064	0.066	0.056	0.058	0.051	0.076	0.063	0.059	0.063	0.072	0.058	0.046	0.060	0.050	0.117	0.133	0.084	0.108	0.089	0.133	0.072
F220_549.2987_14.1_P	0.004	0.007	0.033	0.006	0.026	0.018	0.008	0.034	0.027	0.008	0.013	0.003	0.072	0.036	0.033	0.004	0.038	0.029	0.004	0.030	0.024	0.015	0.047	0.078	0.020	0.088	0.037	0.008	0.014	0.008	0.021	0.023	0.013	0.017	0.016	0.007	0.034	0.017	0.028	0.017	0.024	0.003	0.033	0.027	0.059	0.035	0.004	0.015	0.005	0.002	0.017	0.029	0.006
F221_343.1887_11.4_P	0.188	0.402	0.082	0.206	0.037	0.048	0.417	0.049	0.097	0.132	0.058	0.062	0.057	0.065	0.064	0.048	0.063	0.170	0.309	0.096	0.057	0.059	0.060	0.036	0.058	0.041	0.035	0.064	0.035	0.070	0.107	0.074	0.076	0.095	0.136	0.071	0.089	0.225	0.049	0.058	0.073	0.200	0.050	0.048	0.073	0.045	0.101	0.065	0.107	0.075	0.055	0.057	0.495
F222_627.5346_3.1_P	0.097	0.098	0.058	0.120	0.030	0.063	0.106	0.044	0.080	0.167	0.068	0.059	0.044	0.100	0.036	0.046	0.103	0.116	0.101	0.081	0.062	0.040	0.062	0.047	0.071	0.055	0.039	0.075	0.035	0.082	0.104	0.086	0.071	0.083	0.090	0.068	0.080	0.118	0.050	0.037	0.066	0.079	0.033	0.020	0.050	0.028	0.092	0.065	0.092	0.069	0.035	0.048	0.142
F224_855.5025_3.1_N	0.028	0.064	0.047	0.068	0.033	0.044	0.051	0.061	0.058	0.053	0.029	0.058	0.041	0.059	0.042	0.027	0.067	0.057	0.035	0.040	0.038	0.028	0.037	0.075	0.044	0.069	0.055	0.041	0.029	0.041	0.059	0.048	0.029	0.030	0.038	0.057	0.063	0.045	0.032	0.046	0.032	0.033	0.026	0.039	0.026	0.022	0.033	0.035	0.039	0.038	0.020	0.020	0.037
F225_290.0977_16.1_P	0.006	0.006	0.009	0.002	0.030	0.030	0.007	0.008	0.006	0.043	0.023	0.062	0.005	0.030	0.035	0.048	0.024	0.005	0.007	0.003	0.003	0.008	0.011	0.008	0.021	0.011	0.060	0.023	0.071	0.021	0.012	0.007	0.008	0.014	0.016	0.039	0.020	0.010	0.017	0.050	0.016	0.006	0.006	0.008	0.002	0.005	0.009	0.016	0.012	0.011	0.014	0.006	0.027
F226_146.0822_12.2_N	0.053	0.060	0.038	0.043	0.024	0.048	0.097	0.043	0.063	0.046	0.053	0.031	0.042	0.048	0.043	0.046	0.047	0.087	0.072	0.043	0.037	0.050	0.048	0.034	0.045	0.025	0.039	0.029	0.061	0.072	0.069	0.052	0.059	0.076	0.089	0.085	0.047	0.084	0.035	0.030	0.046	0.078	0.045	0.048	0.053	0.045	0.040	0.028	0.040	0.067	0.093	0.039	0.075
F227_492.3233_3.1_P	0.045	0.031	0.071	0.068	0.054	0.086	0.023	0.086	0.075	0.067	0.064	0.051	0.092	0.044	0.087	0.068	0.089	0.051	0.046	0.053	0.060	0.100	0.068	0.060	0.054	0.053	0.050	0.035	0.045	0.153	0.106	0.091	0.079	0.091	0.086	0.066	0.111	0.196	0.041	0.034	0.059	0.040	0.065	0.036	0.065	0.083	0.062	0.059	0.054	0.052	0.050	0.060	0.033
F228_330.0688_11.2_N	0.063	0.064	0.041	0.057	0.050	0.035	0.056	0.036	0.041	0.040	0.045	0.034	0.040	0.022	0.034	0.027	0.049	0.052	0.063	0.053	0.034	0.031	0.033	0.023	0.040	0.055	0.030	0.040	0.047	0.052	0.063	0.049	0.068	0.055	0.052	0.042	0.055	0.071	0.034	0.047	0.017	0.100	0.039	0.027	0.068	0.047	0.040	0.041	0.059	0.060	0.060	0.057	0.099
F229_300.1798_7.4_P	0.003	0.009	0.020	0.022	0.021	0.027	0.011	0.023	0.018	0.020	0.018	0.041	0.025	0.020	0.025	0.045	0.045	0.026	0.008	0.040	0.017	0.032	0.058	0.027	0.019	0.016	0.028	0.029	0.056	0.030	0.060	0.034	0.045	0.034	0.029	0.037	0.035	0.041	0.022	0.035	0.036	0.002	0.028	0.065	0.052	0.056	0.039	0.029	0.038	0.070	0.076	0.050	0.005

F231_241.1809_3.5_N	0.025	0.018	0.028	0.024	0.061	0.050	0.023	0.035	0.039	0.040	0.042	0.048	0.038	0.024	0.044	0.037	0.036	0.021	0.018	0.032	0.033	0.036	0.037	0.060	0.050	0.037	0.063	0.031	0.032	0.053	0.041	0.029	0.035	0.024	0.018	0.047	0.024	0.033	0.043	0.040	0.034	0.026	0.059	0.056	0.031	0.058	0.044	0.042	0.050	0.047	0.046	0.087	0.030
F232_344.279_4.3_P	0.024	0.019	0.024	0.018	0.039	0.032	0.011	0.025	0.044	0.029	0.031	0.043	0.029	0.023	0.053	0.036	0.032	0.038	0.025	0.039	0.034	0.026	0.020	0.028	0.040	0.033	0.031	0.031	0.039	0.075	0.033	0.037	0.043	0.025	0.031	0.036	0.032	0.040	0.007	0.007	0.008	0.019	0.022	0.020	0.006	0.019	0.021	0.010	0.047	0.018	0.022	0.104	0.007
F233_367.3579_3.2_N	0.030	0.027	0.070	0.049	0.066	0.084	0.021	0.060	0.077	0.059	0.055	0.064	0.058	0.038	0.067	0.062	0.097	0.045	0.034	0.075	0.067	0.085	0.077	0.094	0.077	0.067	0.087	0.054	0.094	0.061	0.085	0.048	0.072	0.048	0.039	0.077	0.054	0.060	0.062	0.050	0.058	0.030	0.090	0.069	0.043	0.068	0.090	0.073	0.074	0.078	0.084	0.094	0.034
F234_191.0844_11.6_P	0.017	0.008	0.025	0.021	0.031	0.034	0.014	0.036	0.025	0.014	0.026	0.026	0.035	0.043	0.037	0.038	0.030	0.023	0.014	0.026	0.037	0.038	0.026	0.030	0.031	0.033	0.031	0.025	0.028	0.033	0.030	0.039	0.028	0.029	0.024	0.027	0.041	0.026	0.053	0.071	0.071	0.047	0.066	0.077	0.062	0.082	0.021	0.024	0.026	0.029	0.035	0.026	0.006
F235_605.4056_3.1_N	0.035	0.055	0.047	0.071	0.060	0.054	0.063	0.109	0.062	0.037	0.035	0.048	0.054	0.056	0.063	0.071	0.086	0.052	0.020	0.042	0.088	0.027	0.040	0.093	0.032	0.054	0.039	0.066	0.029	0.081	0.075	0.061	0.045	0.025	0.027	0.038	0.064	0.058	0.044	0.051	0.052	0.025	0.041	0.057	0.029	0.022	0.046	0.041	0.034	0.034	0.029	0.042	0.040
F236_284.1601_10.3_P	0.008	0.010	0.002	0.003	0.002	0.015	0.007	0.014	0.014	0.003	0.005	0.036	0.002	0.035	0.015	0.036	0.120	0.012	0.022	0.020	0.002	0.017	0.065	0.008	0.003	0.006	0.005	0.011	0.030	0.014	0.021	0.006	0.002	0.002	0.002	0.019	0.007	0.032	0.002	0.005	0.003	0.004	0.003	0.002	0.011	0.002	0.011	0.006	0.012	0.073	0.030	0.026	0.006
F237_439.1852_10.4_P	0.055	0.053	0.023	0.057	0.013	0.035	0.021	0.039	0.015	0.030	0.011	0.018	0.024	0.037	0.025	0.013	0.023	0.035	0.004	0.009	0.005	0.017	0.015	0.045	0.013	0.045	0.015	0.029	0.023	0.036	0.020	0.023	0.007	0.016	0.018	0.042	0.023	0.029	0.036	0.035	0.052	0.061	0.037	0.034	0.044	0.037	0.106	0.101	0.061	0.090	0.095	0.077	0.133
F238_347.149_9.3_P	0.003	0.005	0.001	0.002	0.007	0.006	0.010	0.007	0.003	0.191	0.008	0.134	0.001	0.028	0.006	0.043	0.018	0.002	0.002	0.001	0.001	0.004	0.003	0.001	0.011	0.001	0.053	0.004	0.004	0.030	0.001	0.003	0.004	0.001	0.001	0.013	0.017	0.002	0.061	0.044	0.026	0.059	0.040	0.034	0.059	0.057	0.044	0.024	0.115	0.116	0.079	0.051	0.085
F239_638.3301_3.7_P	0.026	0.031	0.020	0.018	0.013	0.008	0.050	0.008	0.019	0.018	0.009	0.011	0.011	0.054	0.009	0.011	0.011	0.018	0.025	0.016	0.009	0.007	0.010	0.009	0.019	0.017	0.010	0.015	0.007	0.017	0.012	0.012	0.010	0.011	0.010	0.017	0.018	0.012	0.015	0.029	0.028	0.040	0.018	0.055	0.023	0.019	0.013	0.010	0.011	0.009	0.007	0.009	0.025
F240_676.5474_3.7_P	0.301	0.523	0.293	0.511	0.179	0.142	0.456	0.274	0.252	0.225	0.182	0.173	0.241	0.734	0.164	0.191	0.134	0.239	0.320	0.310	0.130	0.160	0.209	0.136	0.129	0.178	0.132	0.201	0.093	0.293	0.187	0.202	0.152	0.181	0.185	0.156	0.217	0.134	0.101	0.165	0.164	0.179	0.108	0.299	0.139	0.184	0.166	0.114	0.250	0.286	0.118	0.161	0.384
F241_257.1466_11.3_P	0.103	0.050	0.065	0.096	0.028	0.047	0.007	0.057	0.117	0.145	0.061	0.108	0.074	0.016	0.074	0.059	0.067	0.138	0.041	0.114	0.042	0.054	0.053	0.046	0.109	0.047	0.041	0.108	0.021	0.043	0.052	0.071	0.034	0.053	0.064	0.050	0.051	0.055	0.013	0.005	0.016	0.053	0.009	0.023	0.012	0.007	0.061	0.028	0.046	0.021	0.006	0.024	0.028
F242_130.0609_12.3_P	0.046	0.030	0.064	0.058	0.045	0.107	0.041	0.076	0.048	0.030	0.040	0.067	0.049	0.047	0.061	0.088	0.089	0.065	0.039	0.051	0.039	0.057	0.082	0.074	0.036	0.049	0.057	0.043	0.085	0.077	0.046	0.066	0.028	0.043	0.034	0.111	0.094	0.038	0.076	0.068	0.070	0.060	0.071	0.082	0.047	0.047	0.015	0.022	0.012	0.088	0.101	0.012	0.007
F243_131.0825_27.1_N	0.029	0.015	0.035	0.034	0.047	0.045	0.004	0.054	0.048	0.036	0.058	0.032	0.064	0.027	0.048	0.047	0.036	0.040	0.031	0.045	0.063	0.068	0.046	0.044	0.048	0.042	0.056	0.025	0.071	0.055	0.078	0.073	0.097	0.082	0.084	0.066	0.054	0.045	0.027	0.029	0.038	0.020	0.038	0.021	0.038	0.040	0.044	0.049	0.053	0.057	0.078	0.062	0.025
F244_223.0391_10.2_N	0.031	0.017	0.032	0.019	0.042	0.055	0.015	0.038	0.023	0.014	0.023	0.040	0.038	0.015	0.037	0.045	0.042	0.024	0.026	0.022	0.019	0.040	0.043	0.043	0.022	0.034	0.041	0.031	0.059	0.037	0.032	0.034	0.028	0.029	0.028	0.040	0.040	0.029	0.043	0.056	0.069	0.031	0.075	0.077	0.049	0.075	0.026	0.025	0.027	0.056	0.059	0.032	0.016
F245_730.5731_3.5_P	0.169	0.288	0.082	0.191	0.074	0.083	0.382	0.093	0.106	0.223	0.119	0.046	0.074	0.215	0.109	0.080	0.108	0.173	0.232	0.131	0.106	0.071	0.075	0.057	0.101	0.090	0.125	0.103	0.061	0.173	0.068	0.164	0.112	0.141	0.054	0.081	0.093	0.187	0.055	0.051	0.058	0.207	0.060	0.079	0.058	0.047	0.111	0.081	0.174	0.102	0.079	0.097	0.303
F246_215.1387_12_P	0.054	0.014	0.064	0.032	0.057	0.085	0.024	0.043	0.038	0.044	0.058	0.092	0.058	0.054	0.072	0.108	0.095	0.047	0.040	0.033	0.055	0.069	0.078	0.038	0.058	0.030	0.069	0.046	0.073	0.080	0.056	0.083	0.061	0.100	0.094	0.094	0.094	0.063	0.094	0.071	0.076	0.059	0.062	0.054	0.057	0.045	0.082	0.079	0.069	0.100	0.097	0.085	0.070
F248_252.0774_15.2_P	0.046	0.014	0.034	0.031	0.021	0.038	0.014	0.036	0.039	0.047	0.043	0.043	0.035	0.021	0.036	0.041	0.032	0.037	0.033	0.053	0.038	0.047	0.050	0.045	0.049	0.052	0.068	0.098	0.043	0.061	0.058	0.045	0.037	0.037	0.043	0.071	0.036	0.054	0.040	0.046	0.054	0.088	0.050	0.058	0.066	0.056	0.199	0.132	0.148	0.136	0.084	0.084	0.104
F250_339.2527_3.1_P	0.037	0.053	0.023	0.038	0.041	0.039	0.027	0.041	0.058	0.058	0.055	0.045	0.093	0.059	0.084	0.089	0.040	0.055	0.038	0.031	0.054	0.061	0.045	0.060	0.053	0.073	0.088	0.061	0.081	0.072	0.060	0.052	0.057	0.048	0.050	0.060	0.079	0.039	0.037	0.056	0.037	0.055	0.072	0.061	0.039	0.060	0.068	0.052	0.081	0.074	0.053	0.079	0.044
F251_177.0767_9.1_N	0.027	0.020	0.034	0.023	0.037	0.075	0.020	0.040	0.028	0.017	0.027	0.040	0.035	0.018	0.038	0.042	0.053	0.030	0.023	0.031	0.021	0.034	0.036	0.043	0.032	0.036	0.052	0.029	0.065	0.042	0.030	0.037	0.019	0.027	0.024	0.050	0.047	0.034	0.072	0.062	0.074	0.041	0.074	0.071	0.042	0.053	0.028	0.027	0.024	0.056	0.058	0.020	0.015
F252_579.4205_3.8_P	0.215	0.310	0.136	0.241	0.129	0.113	0.158	0.151	0.213	0.111	0.105	0.293	0.134	0.085	0.090	0.090	0.115	0.155	0.177	0.266	0.155	0.158	0.140	0.158	0.099	0.185	0.109	0.148	0.084	0.161	0.125	0.104	0.120	0.079	0.111	0.118	0.104	0.099	0.240	0.250	0.201	0.317	0.176	0.172	0.117	0.118	0.050	0.049	0.062	0.064	0.039	0.032	0.079
F253_166.0721_9.6_P	0.031	0.017	0.027	0.024	0.046	0.037	0.014	0.033	0.029	0.031	0.033	0.049	0.034	0.034	0.046	0.039	0.032	0.025	0.019	0.023	0.023	0.033	0.031	0.030	0.032	0.026	0.041	0.035	0.041	0.041	0.038	0.038	0.042	0.050	0.043	0.057	0.074	0.038	0.025	0.029	0.027	0.025	0.035	0.027	0.033	0.030	0.098	0.088	0.071	0.097	0.079	0.081	0.051
F254_437.1695_9.8_P	0.002	0.006	0.002	0.002	0.001	0.002	0.007	0.002	0.001	0.002	0.002	0.002	0.002	0.034	0.002	0.002	0.001	0.002	0.005	0.002	0.001	0.002	0.002	0.002	0.003	0.001	0.002	0.003	0.001	0.002	0.006	0.011	0.006	0.009	0.011	0.017	0.011	0.018	0.005	0.003	0.006	0.010	0.009	0.002	0.003	0.003	0.067	0.045	0.105	0.117	0.078	0.075	0.097
F255_152.9958_12.3_N	0.021	0.006	0.040	0.021	0.025	0.020	0.013	0.021	0.013	0.016	0.020	0.015	0.016	0.015	0.017	0.026	0.021	0.013	0.021	0.012	0.018	0.022	0.023	0.020	0.012	0.019	0.019	0.015	0.011	0.024	0.020	0.010	0.011	0.019	0.016	0.014	0.013	0.012	0.038	0.054	0.079	0.014	0.076	0.037	0.049	0.032	0.020	0.070	0.043	0.020	0.026	0.025	0.019
F256_142.0508_9.5_N	0.004	0.017	0.009	0.003	0.005	0.008	0.004	0.003	0.005	0.002	0.003	0.006	0.009	0.049	0.005	0.008	0.003	0.005	0.004	0.003	0.002	0.006	0.002	0.012	0.004	0.006	0.008	0.006	0.011	0.003	0.001	0.005	0.005	0.003	0.004	0.003	0.004	0.001	0.032	0.034	0.040	0.018	0.044	0.043	0.053	0.036	0.004	0.003	0.006	0.005	0.004	0.005	0.012
F257_148.0037_15.9_P	0.070	0.105	0.046	0.092	0.047	0.047	0.129	0.051	0.036	0.057	0.048	0.048	0.045	0.183	0.044	0.051	0.054	0.075	0.092	0.063	0.045	0.041	0.042	0.036	0.048	0.030	0.048	0.074	0.051	0.072	0.037	0.056	0.041	0.068	0.048	0.047	0.049	0.074	0.045	0.036	0.045	0.049	0.045	0.035	0.054	0.035	0.092	0.082	0.088	0.063	0.030	0.069	0.157
F258_619.0933_14.3_N	0.011	0.002	0.012	0.003	0.021	0.021	0.003	0.020	0.016	0.016	0.024	0.020	0.021	0.014	0.017	0.021	0.019	0.007	0.003	0.015	0.017	0.017	0.015	0.028	0.020	0.025	0.033	0.034	0.028	0.036	0.022	0.023	0.024	0.014	0.015	0.030	0.022	0.023	0.025	0.029	0.032	0.025	0.029	0.029	0.032	0.025	0.052	0.081	0.050	0.048	0.043	0.059	0.025
F259_177.0767_13.8_N	0.037	0.050	0.047	0.045	0.060	0.053	0.042	0.066	0.049	0.041	0.040	0.052	0.066	0.073	0.064	0.055	0.064	0.048	0.045	0.043	0.073	0.055	0.056	0.054	0.052	0.055	0.065	0.048	0.049	0.053	0.060	0.058	0.050	0.056	0.044	0.062	0.074	0.066	0.041	0.040	0.039	0.034	0.041	0.035	0.040	0.047	0.044	0.044	0.049	0.051	0.042	0.051	0.056
F261_131.0825_16.2_N	0.024	0.015	0.032	0.024	0.034	0.042	0.014	0.038	0.035	0.023	0.041	0.026	0.041	0.011	0.036	0.033	0.030	0.027	0.026	0.026	0.040	0.037	0.028	0.042	0.037	0.030	0.048	0.026	0.048	0.030	0.040	0.052	0.058	0.050	0.052	0.042	0.039	0.028	0.050	0.052	0.054	0.032	0.058	0.048	0.043	0.055	0.029	0.020	0.026	0.031	0.047	0.044	0.016
F262_227.1036_7.3_N	0.013	0.001	0.016	0.015	0.009	0.012	0.006	0.011	0.007	0.009	0.009	0.011	0.016	0.005	0.017	0.007	0.018	0.011	0.016	0.008	0.016	0.014	0.022	0.032	0.015	0.022	0.016	0.029	0.054	0.014	0.035	0.019	0.036	0.019	0.023	0.029	0.017	0.023	0.013	0.010	0.023	0.000	0.016	0.009	0.030	0.015	0.041	0.028	0.034	0.037	0.032	0.039	0.014
F263_164.1162_13.2_P	0.016	0.007	0.017	0.009	0.023	0.025	0.007	0.022	0.017	0.029	0.034	0.032	0.022	0.031	0.024	0.038	0.027	0.017	0.013	0.014	0.025	0.029	0.025	0.021	0.027	0.019	0.033	0.027	0.040	0.035	0.030	0.027	0.029	0.032	0.026	0.039	0.023	0.019	0.017	0.024	0.037	0.024	0.029	0.021	0.020	0.028	0.049	0.067	0.057	0.051	0.058	0.109	0.015
F264_183.1389_3.6_N	0.019	0.032	0.042	0.055	0.061	0.053	0.053	0.074	0.075	0.028	0.036	0.040	0.039	0.025	0.046	0.049	0.057	0.047	0.028	0.036	0.057	0.040	0.032	0.072	0.048	0.048	0.067	0.053	0.042	0.037	0.011	0.042	0.030	0.016	0.011	0.038	0.036	0.022	0.034	0.031	0.037	0.030	0.041	0.053	0.027	0.040	0.014	0.028	0.025	0.016	0.018	0.021	0.012
F265_197.1005_27.1_P	0.043	0.031	0.032	0.054	0.062	0.049	0.007	0.073	0.067	0.050	0.063	0.038	0.062	0.031	0.054	0.041	0.053	0.072	0.045	0.069	0.068	0.068	0.043	0.049	0.062	0.063	0.056	0.031	0.110	0.061	0.099	0.103	0.105	0.094	0.090	0.062	0.063	0.043	0.037	0.038	0.050	0.023	0.042	0.024	0.038	0.044	0.060	0.063	0.053	0.065	0.048	0.063	0.046
F267_618.3098_7.8_N	0.002	0.004	0.005	0.003	0.008	0.004	0.004	0.008	0.005	0.003	0.002	0.001	0.019	0.020	0.009	0.001	0.010	0.005	0.002	0.004	0.023	0.004	0.013	0.068	0.009	0.056	0.013	0.017	0.022	0.010	0.011	0.019	0.013	0.007	0.001	0.005	0.013	0.020	0.009	0.003	0.015	0.007	0.019	0.007	0.028	0.010	0.007	0.008	0.002	0.001	0.008	0.011	0.003
F268_197.1281_8.9_P	0.022	0.039	0.024	0.017	0.016	0.027	0.033	0.031	0.010	0.008	0.007	0.004	0.014	0.068	0.011	0.016	0.005	0.011	0.011	0.006	0.007	0.010	0.009	0.006	0.006	0.005	0.007	0.008	0.013	0.007	0.008	0.011	0.011	0.006	0.012	0.011	0.006	0.005	0.022	0.020	0.036	0.023	0.025	0.016	0.016	0.029	0.006	0.005	0.006	0.013	0.018	0.006	0.009
F270_516.2799_4.1_N	0.177	0.001	0.017	0.002	0.000	0.000	0.026	0.000	0.000	0.000	0.035	0.000	0.000	0.022	0.000	0.071	0.000	0.000	0.002	0.014	0.000	0.000	0.020	0.036	0.000	0.002	0.078	0.070	0.001	0.034	0.118	0.000	0.062	0.000	0.000	0.000	0.000	0.000	0.008	0.062	0.066	0.006	0.009	0.055	0.025	0.056	0.000	0.000	0.000	0.000	0.061	0.000	0.001
F271_260.0234_4.2_N	0.016	0.008	0.023	0.010	0.023	0.014	0.005	0.023	0.036	0.025	0.013	0.024	0.019	0.012	0.031	0.014	0.029	0.004	0.011	0.020	0.030	0.005	0.011	0.037	0.043	0.049	0.063	0.042	0.051	0.039	0.013	0.007	0.012	0.011	0.028	0.026	0.037	0.013	0.018	0.019	0.026	0.019	0.013	0.017	0.027	0.011	0.036	0.039	0.041	0.019	0.017	0.014	0.020
F274_638.1347_14.3_P	0.007	0.011	0.009	0.009	0.018	0.018	0.012	0.018	0.011	0.013	0.020	0.014	0.018	0.055	0.014	0.021	0.015	0.007	0.005	0.011	0.016	0.016	0.012	0.020	0.018	0.022	0.024	0.027	0.027	0.026	0.016	0.020	0.023	0.010	0.012	0.029	0.018	0.022	0.021	0.029	0.029	0.024	0.028	0.028	0.029	0.027	0.052	0.071	0.054	0.045	0.044	0.051	0.023
F275_351.1577_13.9_P	0.008	0.010	0.020	0.015	0.019	0.036	0.012	0.023	0.013	0.006	0.010	0.032	0.025	0.036	0.022	0.032	0.030	0.018	0.012	0.012	0.010	0.016	0.018	0.028	0.012	0.021	0.036	0.015	0.034	0.018	0.018	0.020	0.007	0.014	0.009	0.031	0.038	0.014	0.042	0.041	0.045	0.021	0.042	0.054	0.031	0.040	0.025	0.022	0.024	0.037	0.026	0.023	0.006
F276_242.1133_8.5_P	0.012	0.005	0.015	0.013	0.023	0.011	0.005	0.006	0.003	0.029	0.011	0.006	0.002	0.024	0.004	0.016	0.007	0.003	0.006	0.004	0.001	0.012	0.005	0.034	0.006	0.073	0.013	0.018	0.022	0.005	0.004	0.009	0.002	0.022	0.012	0.015	0.013	0.001	0.004	0.008	0.013	0.006	0.004	0.004	0.004	0.010	0.024	0.068	0.006	0.012	0.028	0.010	0.009
F278_418.2997_3.3_N	0.003	0.005	0.005	0.005	0.062	0.005	0.005	0.008	0.004	0.003	0.005	0.005	0.006	0.016	0.005	0.004	0.004	0.003	0.003	0.004	0.005	0.005	0.005	0.006	0.005	0.008	0.008	0.005	0.004	0.004	0.004	0.004	0.026	0.003	0.003	0.004	0.009	0.004	0.008	0.009	0.027	0.002	0.004	0.004	0.003	0.004	0.003	0.003	0.003	0.004	0.003	0.018	0.004
F279_454.3886_3.9_P	0.096	0.049	0.066	0.091	0.069	0.049	0.074	0.063	0.058	0.041	0.036	0.094	0.053	0.067	0.027	0.067	0.051	0.066	0.049	0.164	0.042	0.048	0.091	0.067	0.063	0.047	0.043	0.058	0.044	0.070	0.084	0.065	0.112	0.053	0.076	0.103	0.050	0.137	0.049	0.022	0.033	0.137	0.073	0.062	0.062	0.085	0.118	0.051	0.154	0.086	0.063	0.204	0.068
F280_176.0913_13.3_P	0.015	0.011	0.024	0.019	0.020	0.043	0.039	0.027	0.016	0.012	0.014	0.021	0.027	0.114	0.017	0.024	0.028	0.020	0.021	0.017	0.013	0.017	0.024	0.033	0.015	0.016	0.028	0.018	0.046	0.023	0.018	0.020	0.010	0.018	0.018	0.028	0.035	0.017	0.054	0.045	0.056	0.040	0.057	0.046	0.047	0.052	0.015	0.019	0.015	0.025	0.027	0.014	0.012
F281_559.15_11.2_P	0.216	0.039	0.045	0.229	0.008	0.047	0.146	0.039	0.100	0.183	0.043	0.049	0.044	0.026	0.032	0.031	0.044	0.236	0.212	0.206	0.058	0.037	0.028	0.032	0.048	0.048	0.033	0.056	0.029	0.059	0.074	0.046	0.059	0.049	0.079	0.056	0.068	0.227	0.051	0.038	0.054	0.366	0.050	0.037	0.057	0.027	0.057	0.047	0.069	0.067	0.038	0.045	0.355
F282_179.056_17.2_N	0.059	0.099	0.092	0.096	0.045	0.054	0.068	0.065	0.076	0.082	0.057	0.076	0.079	0.042	0.049	0.028	0.060	0.109	0.084	0.098	0.062	0.043	0.041	0.059	0.053	0.050	0.040	0.049	0.027	0.039	0.071	0.126	0.051	0.113	0.073	0.069	0.073	0.091	0.040	0.018	0.021	0.115	0.014	0.015	0.007	0.011	0.059	0.018	0.075	0.023	0.011	0.013	0.122
F283_129.1021_25.3_P	0.021	0.057	0.036	0.022	0.051	0.037	0.057	0.039	0.037	0.025	0.067	0.032	0.068	0.261	0.055	0.062	0.034	0.034	0.029	0.027	0.082	0.080	0.068	0.047	0.057	0.047	0.057	0.059	0.074	0.044	0.093	0.076	0.103	0.071	0.058	0.066	0.050	0.033	0.023	0.021	0.031	0.010	0.027	0.022	0.028	0.034	0.032	0.043	0.057	0.057	0.060	0.062	0.008
F284_302.1343_9.7_P	0.003	0.008	0.003	0.003	0.003	0.002	0.008	0.004	0.002	0.007	0.002	0.002	0.003	0.037	0.004	0.002	0.003	0.009	0.004	0.002	0.002	0.004	0.002	0.009	0.001	0.005	0.004	0.003	0.002	0.003	0.004	0.005	0.002	0.003	0.002	0.005	0.004	0.002	0.005	0.005	0.007	0.010	0.006	0.004	0.007	0.004	0.026	0.072	0.019	0.013	0.004	0.029	0.009
F285_845.5746_3.4_N	0.177	0.187	0.158	0.203	0.141	0.137	0.337	0.140	0.193	0.189	0.148	0.151	0.064	0.671	0.146	0.106	0.191	0.168	0.117	0.168	0.115	0.074	0.047	0.172	0.153	0.171	0.135	0.157	0.089	0.121	0.173	0.132	0.140	0.127	0.102	0.111	0.160	0.137	0.117	0.134	0.103	0.113	0.082	0.098	0.092	0.073	0.138	0.155	0.103	0.146	0.087	0.104	0.182
F288_359.1191_15.5_N	0.043	0.005	0.034	0.029	0.025	0.024	0.270	0.043	0.045	0.036	0.056	0.020	0.045	0.200	0.036	0.057	0.047	0.124	0.076	0.051	0.056	0.089	0.109	0.029	0.086	0.032	0.029	0.042	0.057	0.051	0.083	0.074	0.062	0.141	0.063	0.029	0.063	0.088	0.016	0.010	0.016	0.079	0.028	0.019	0.018	0.023	0.022	0.026	0.031	0.020	0.016	0.030	0.062
F289_219.0973_10.5_P	0.046	0.014	0.038	0.037	0.052	0.047	0.013	0.055	0.049	0.030	0.045	0.034	0.041	0.055	0.031	0.038	0.032	0.029	0.031	0.034	0.042	0.043	0.044	0.046	0.092	0.057	0.102	0.087	0.068	0.051	0.066	0.071	0.064	0.081	0.061	0.062	0.083	0.031	0.033	0.033	0.026	0.017	0.018	0.011	0.021	0.026	0.037	0.040	0.055	0.036	0.050	0.039	0.022
F290_474.2619_4_N	0.131	0.135	0.133	0.116	0.102	0.075	0.037	0.123	0.093	0.106	0.075	0.112	0.077	0.073	0.101	0.052	0.104	0.083	0.112	0.137	0.117	0.148	0.145	0.101	0.089	0.107	0.058	0.087	0.069	0.082	0.056	0.048	0.054	0.074	0.067	0.084	0.048	0.058	0.103	0.072	0.070	0.113	0.100	0.048	0.100	0.077	0.056	0.063	0.079	0.063	0.022	0.067	0.036

F291_626.08_15.9_P	0.040	0.006	0.036	0.018	0.040	0.042	0.007	0.046	0.043	0.055	0.052	0.041	0.041	0.030	0.038	0.046	0.051	0.027	0.027	0.053	0.044	0.034	0.035	0.042	0.045	0.043	0.048	0.059	0.050	0.047	0.038	0.045	0.048	0.047	0.039	0.048	0.039	0.054	0.045	0.041	0.054	0.048	0.042	0.034	0.044	0.036	0.087	0.078	0.073	0.064	0.055	0.076	0.043
F292_252.1437_13.9_P	0.030	0.029	0.036	0.018	0.038	0.056	0.026	0.031	0.020	0.029	0.022	0.040	0.030	0.032	0.028	0.047	0.044	0.034	0.039	0.026	0.025	0.027	0.044	0.021	0.019	0.021	0.040	0.024	0.063	0.037	0.029	0.030	0.015	0.042	0.039	0.047	0.047	0.030	0.068	0.057	0.056	0.043	0.047	0.045	0.037	0.046	0.035	0.028	0.027	0.042	0.045	0.026	0.030
F293_320.2059_9.8_P	0.013	0.007	0.017	0.004	0.014	0.022	0.008	0.021	0.017	0.012	0.013	0.029	0.014	0.037	0.023	0.032	0.022	0.011	0.009	0.013	0.013	0.011	0.010	0.017	0.018	0.014	0.024	0.017	0.014	0.019	0.022	0.020	0.011	0.019	0.013	0.025	0.024	0.032	0.021	0.015	0.018	0.011	0.017	0.017	0.028	0.019	0.047	0.013	0.041	0.042	0.069	0.033	0.010
F294_502.1569_11.4_P	0.139	0.009	0.089	0.143	0.022	0.037	0.585	0.033	0.101	0.086	0.066	0.067	0.048	0.028	0.051	0.041	0.072	0.065	0.011	0.118	0.055	0.045	0.065	0.030	0.063	0.044	0.030	0.051	0.022	0.070	0.081	0.060	0.064	0.080	0.084	0.059	0.070	0.131	0.060	0.035	0.034	0.191	0.053	0.050	0.045	0.038	0.087	0.048	0.097	0.070	0.036	0.046	0.022
F295_791.5408_3.2_P	0.133	0.231	0.054	0.101	0.059	0.053	0.158	0.050	0.108	0.165	0.114	0.078	0.101	0.600	0.063	0.089	0.091	0.106	0.165	0.069	0.073	0.069	0.066	0.068	0.060	0.054	0.081	0.060	0.071	0.086	0.067	0.058	0.080	0.092	0.077	0.068	0.066	0.144	0.042	0.061	0.065	0.134	0.049	0.039	0.082	0.065	0.075	0.029	0.058	0.073	0.033	0.048	0.256
F296_289.1405_9.6_N	0.041	0.012	0.028	0.032	0.028	0.038	0.015	0.025	0.036	0.021	0.033	0.036	0.029	0.010	0.030	0.026	0.040	0.038	0.029	0.041	0.032	0.039	0.020	0.024	0.042	0.039	0.032	0.033	0.042	0.023	0.042	0.040	0.040	0.034	0.033	0.033	0.036	0.016	0.061	0.038	0.035	0.046	0.034	0.032	0.035	0.019	0.031	0.029	0.035	0.039	0.043	0.020	0.018
F297_157.097_10.5_P	0.030	0.013	0.033	0.023	0.039	0.034	0.051	0.031	0.027	0.036	0.082	0.054	0.024	0.029	0.056	0.058	0.032	0.020	0.019	0.025	0.038	0.041	0.031	0.035	0.054	0.038	0.042	0.044	0.060	0.017	0.035	0.028	0.024	0.038	0.022	0.035	0.027	0.025	0.020	0.027	0.027	0.022	0.032	0.027	0.034	0.018	0.021	0.014	0.019	0.023	0.026	0.018	0.048
F298_525.2817_3.8_N	0.109	0.125	0.157	0.127	0.098	0.107	0.086	0.142	0.135	0.127	0.075	0.131	0.120	0.071	0.125	0.069	0.093	0.107	0.098	0.189	0.101	0.166	0.136	0.090	0.089	0.108	0.088	0.125	0.069	0.098	0.064	0.067	0.080	0.087	0.080	0.104	0.070	0.083	0.238	0.160	0.147	0.262	0.277	0.201	0.225	0.181	0.055	0.074	0.073	0.077	0.039	0.074	0.064
F299_568.3251_4_N	0.052	0.039	0.055	0.064	0.042	0.039	0.037	0.048	0.056	0.035	0.038	0.046	0.044	0.022	0.047	0.031	0.032	0.036	0.027	0.053	0.039	0.045	0.047	0.046	0.045	0.054	0.034	0.051	0.044	0.034	0.032	0.034	0.037	0.029	0.038	0.038	0.019	0.029	0.050	0.035	0.045	0.058	0.045	0.033	0.036	0.038	0.038	0.042	0.042	0.040	0.026	0.039	0.024
F300_146.1172_9.2_P	0.031	0.025	0.044	0.034	0.056	0.049	0.026	0.067	0.040	0.033	0.052	0.037	0.039	0.040	0.030	0.053	0.054	0.037	0.031	0.045	0.068	0.058	0.090	0.052	0.050	0.045	0.048	0.046	0.070	0.044	0.060	0.036	0.058	0.061	0.034	0.044	0.041	0.042	0.020	0.021	0.026	0.020	0.057	0.023	0.027	0.038	0.034	0.031	0.028	0.046	0.051	0.035	0.025
F301_167.013_14_N	0.040	0.023	0.019	0.020	0.014	0.026	0.019	0.020	0.015	0.022	0.009	0.027	0.017	0.026	0.029	0.025	0.042	0.026	0.050	0.026	0.016	0.023	0.029	0.018	0.015	0.018	0.026	0.016	0.039	0.049	0.026	0.015	0.014	0.020	0.045	0.028	0.022	0.026	0.042	0.046	0.068	0.111	0.044	0.021	0.047	0.046	0.024	0.017	0.025	0.023	0.038	0.019	0.032
F304_192.1227_13.9_P	0.017	0.015	0.027	0.020	0.031	0.048	0.015	0.032	0.017	0.019	0.015	0.034	0.026	0.033	0.023	0.036	0.035	0.023	0.025	0.021	0.018	0.024	0.031	0.019	0.012	0.018	0.031	0.017	0.044	0.031	0.017	0.025	0.012	0.030	0.027	0.034	0.039	0.021	0.064	0.056	0.056	0.040	0.054	0.048	0.042	0.051	0.020	0.020	0.016	0.028	0.030	0.017	0.013
F305_328.0339_15.7_N	0.008	0.004	0.008	0.009	0.013	0.011	0.012	0.012	0.021	0.010	0.025	0.008	0.019	0.016	0.009	0.017	0.015	0.008	0.013	0.019	0.015	0.017	0.015	0.036	0.031	0.043	0.062	0.055	0.065	0.056	0.022	0.020	0.037	0.019	0.021	0.031	0.043	0.054	0.016	0.022	0.024	0.020	0.022	0.025	0.017	0.017	0.033	0.043	0.023	0.031	0.016	0.030	0.045
F306_367.2845_3.5_N	0.019	0.034	0.040	0.054	0.059	0.055	0.051	0.071	0.075	0.027	0.040	0.051	0.038	0.029	0.044	0.048	0.057	0.046	0.028	0.035	0.059	0.038	0.034	0.072	0.048	0.047	0.066	0.052	0.041	0.035	0.013	0.041	0.029	0.017	0.011	0.039	0.035	0.023	0.034	0.030	0.036	0.030	0.040	0.052	0.026	0.038	0.016	0.028	0.024	0.024	0.018	0.021	0.013
F307_206.1382_13.4_P	0.015	0.021	0.016	0.016	0.019	0.069	0.009	0.022	0.009	0.013	0.016	0.025	0.032	0.045	0.013	0.085	0.055	0.031	0.016	0.026	0.018	0.016	0.051	0.016	0.010	0.010	0.017	0.013	0.060	0.023	0.031	0.026	0.007	0.049	0.022	0.032	0.037	0.056	0.031	0.012	0.027	0.013	0.017	0.013	0.029	0.018	0.026	0.028	0.014	0.074	0.112	0.028	0.017
F308_176.1279_12.8_P	0.028	0.010	0.028	0.023	0.029	0.067	0.012	0.032	0.015	0.022	0.022	0.059	0.029	0.063	0.031	0.048	0.029	0.026	0.036	0.016	0.009	0.030	0.041	0.015	0.024	0.021	0.036	0.019	0.066	0.047	0.031	0.031	0.025	0.062	0.063	0.069	0.042	0.029	0.053	0.046	0.045	0.042	0.038	0.028	0.030	0.043	0.060	0.061	0.052	0.102	0.088	0.062	0.038
F309_208.1327_7.7_P	0.005	0.004	0.004	0.007	0.004	0.006	0.005	0.006	0.004	0.011	0.008	0.008	0.008	0.021	0.007	0.010	0.005	0.003	0.002	0.007	0.001	0.007	0.007	0.010	0.007	0.009	0.015	0.016	0.018	0.014	0.006	0.006	0.004	0.006	0.005	0.018	0.010	0.011	0.013	0.023	0.021	0.017	0.021	0.028	0.022	0.029	0.033	0.039	0.030	0.037	0.041	0.034	0.021
F310_133.0969_8.2_P	0.015	0.013	0.028	0.014	0.031	0.027	0.011	0.032	0.021	0.013	0.021	0.020	0.029	0.019	0.036	0.040	0.021	0.012	0.010	0.016	0.018	0.018	0.018	0.036	0.019	0.023	0.031	0.023	0.036	0.020	0.019	0.032	0.021	0.021	0.020	0.028	0.043	0.024	0.027	0.024	0.021	0.014	0.024	0.028	0.017	0.019	0.016	0.018	0.018	0.025	0.026	0.017	0.012
F311_334.0814_14.5_N	0.063	0.042	0.044	0.051	0.021	0.020	0.040	0.030	0.049	0.070	0.044	0.037	0.033	0.017	0.029	0.035	0.037	0.067	0.063	0.067	0.034	0.048	0.061	0.029	0.057	0.024	0.024	0.057	0.035	0.041	0.058	0.043	0.054	0.049	0.067	0.039	0.036	0.067	0.012	0.001	0.015	0.048	0.015	0.017	0.024	0.021	0.045	0.053	0.057	0.030	0.027	0.032	0.112
F312_866.5683_3.2_P	0.039	0.068	0.018	0.054	0.024	0.020	0.016	0.029	0.033	0.034	0.028	0.018	0.026	0.038	0.025	0.021	0.030	0.034	0.049	0.045	0.024	0.023	0.023	0.015	0.017	0.015	0.015	0.018	0.019	0.028	0.021	0.038	0.019	0.023	0.026	0.021	0.029	0.035	0.022	0.013	0.025	0.029	0.023	0.010	0.020	0.015	0.024	0.010	0.025	0.015	0.016	0.017	0.050
F314_221.0916_8.7_P	0.007	0.005	0.007	0.006	0.011	0.006	0.004	0.009	0.006	0.005	0.005	0.006	0.005	0.031	0.005	0.003	0.006	0.005	0.009	0.006	0.005	0.006	0.005	0.010	0.008	0.009	0.005	0.011	0.006	0.009	0.005	0.010	0.006	0.009	0.009	0.008	0.008	0.006	0.011	0.010	0.015	0.018	0.017	0.011	0.015	0.014	0.022	0.025	0.029	0.022	0.014	0.023	0.017
F315_345.1683_16.6_P	0.007	0.005	0.006	0.002	0.010	0.011	0.005	0.008	0.006	0.010	0.012	0.015	0.009	0.022	0.008	0.013	0.011	0.006	0.002	0.006	0.008	0.009	0.010	0.010	0.010	0.007	0.015	0.011	0.020	0.010	0.012	0.011	0.010	0.013	0.012	0.012	0.012	0.006	0.013	0.019	0.017	0.009	0.012	0.011	0.010	0.013	0.014	0.011	0.012	0.014	0.018	0.015	0.004
F316_153.0418_8_N	0.008	0.006	0.012	0.008	0.017	0.020	0.004	0.017	0.011	0.006	0.012	0.013	0.017	0.011	0.014	0.015	0.013	0.009	0.007	0.010	0.010	0.013	0.011	0.016	0.012	0.013	0.020	0.011	0.019	0.011	0.011	0.015	0.010	0.012	0.009	0.018	0.019	0.008	0.016	0.018	0.015	0.009	0.018	0.016	0.010	0.013	0.008	0.008	0.008	0.012	0.012	0.007	0.003
F318_371.0825_13.6_N	0.001	0.003	0.020	0.014	0.004	0.031	0.003	0.017	0.027	0.024	0.033	0.046	0.029	0.013	0.037	0.002	0.037	0.015	0.012	0.022	0.025	0.020	0.017	0.009	0.036	0.023	0.038	0.001	0.030	0.030	0.026	0.039	0.030	0.036	0.032	0.031	0.055	0.030	0.014	0.018	0.009	0.007	0.006	0.004	0.010	0.008	0.028	0.021	0.029	0.018	0.028	0.015	0.024
F319_208.0648_4.5_N	0.000	0.001	0.005	0.002	0.003	0.004	0.001	0.002	0.002	0.002	0.005	0.010	0.002	0.003	0.004	0.010	0.004	0.000	0.000	0.000	0.003	0.004	0.005	0.003	0.003	0.006	0.003	0.006	0.015	0.004	0.005	0.004	0.006	0.005	0.007	0.005	0.004	0.005	0.013	0.009	0.017	0.000	0.011	0.014	0.009	0.015	0.006	0.003	0.004	0.006	0.011	0.005	0.000
F320_226.1282_9.8_P	0.009	0.015	0.010	0.003	0.012	0.016	0.005	0.009	0.009	0.005	0.006	0.030	0.007	0.023	0.013	0.013	0.010	0.006	0.006	0.009	0.005	0.008	0.009	0.009	0.011	0.009	0.013	0.008	0.012	0.013	0.009	0.009	0.005	0.005	0.005	0.012	0.012	0.008	0.004	0.004	0.003	0.002	0.003	0.003	0.004	0.002	0.010	0.004	0.006	0.011	0.010	0.004	0.004
F321_372.1513_11.4_P	0.055	0.013	0.029	0.007	0.021	0.020	0.479	0.023	0.047	0.009	0.017	0.023	0.033	0.035	0.014	0.010	0.023	0.041	0.005	0.056	0.018	0.012	0.028	0.021	0.022	0.026	0.018	0.029	0.011	0.038	0.023	0.015	0.021	0.024	0.033	0.023	0.038	0.067	0.017	0.010	0.016	0.005	0.020	0.005	0.032	0.010	0.038	0.028	0.038	0.031	0.021	0.027	0.014
F322_337.1094_15.9_P	0.019	0.012	0.016	0.014	0.013	0.016	0.010	0.016	0.016	0.020	0.017	0.016	0.015	0.034	0.016	0.017	0.018	0.016	0.017	0.021	0.014	0.013	0.013	0.019	0.015	0.015	0.015	0.021	0.017	0.018	0.016	0.014	0.015	0.016	0.015	0.017	0.014	0.023	0.017	0.014	0.016	0.021	0.016	0.013	0.019	0.011	0.032	0.024	0.025	0.027	0.019	0.024	0.025
F323_248.1122_13.3_P	0.007	0.007	0.007	0.005	0.010	0.015	0.007	0.009	0.007	0.015	0.007	0.021	0.007	0.030	0.009	0.015	0.020	0.011	0.008	0.008	0.009	0.010	0.016	0.009	0.014	0.010	0.015	0.012	0.019	0.014	0.011	0.013	0.007	0.019	0.016	0.019	0.026	0.011	0.020	0.014	0.015	0.011	0.007	0.011	0.024	0.012	0.020	0.017	0.022	0.020	0.018	0.013	0.020
F326_650.2287_16.2_P	0.029	0.004	0.007	0.012	0.004	0.004	0.004	0.005	0.011	0.007	0.008	0.019	0.016	0.016	0.015	0.016	0.012	0.026	0.007	0.006	0.007	0.022	0.020	0.004	0.014	0.003	0.007	0.023	0.005	0.022	0.023	0.021	0.021	0.018	0.032	0.016	0.022	0.012	0.002	0.001	0.002	0.001	0.001	0.003	0.003	0.001	0.004	0.003	0.010	0.014	0.005	0.006	0.008
F327_271.0038_15.9_N	0.074	0.142	0.022	0.084	0.011	0.014	0.138	0.017	0.028	0.069	0.018	0.017	0.017	0.089	0.015	0.013	0.021	0.078	0.124	0.030	0.016	0.015	0.017	0.013	0.017	0.016	0.015	0.021	0.012	0.023	0.021	0.021	0.020	0.023	0.022	0.017	0.020	0.028	0.016	0.014	0.016	0.043	0.015	0.011	0.017	0.018	0.026	0.022	0.028	0.021	0.017	0.023	0.122
F329_131.0713_8.3_N	0.012	0.009	0.019	0.012	0.013	0.016	0.009	0.010	0.013	0.010	0.010	0.021	0.012	0.037	0.010	0.008	0.010	0.011	0.008	0.010	0.005	0.006	0.005	0.014	0.007	0.010	0.020	0.008	0.017	0.007	0.005	0.007	0.004	0.004	0.004	0.010	0.008	0.005	0.010	0.018	0.014	0.018	0.015	0.012	0.008	0.012	0.004	0.005	0.004	0.006	0.005	0.003	0.011
F330_379.3215_3.2_N	0.008	0.009	0.012	0.013	0.013	0.010	0.010	0.017	0.015	0.012	0.016	0.013	0.029	0.009	0.018	0.018	0.011	0.012	0.009	0.017	0.014	0.014	0.012	0.017	0.012	0.012	0.012	0.014	0.013	0.012	0.012	0.013	0.015	0.009	0.007	0.013	0.014	0.012	0.002	0.001	0.001	0.001	0.002	0.001	0.001	0.003	0.013	0.012	0.013	0.013	0.009	0.011	0.011
F331_258.2423_7.2_P	0.007	0.023	0.004	0.007	0.014	0.019	0.003	0.016	0.028	0.006	0.018	0.027	0.022	0.130	0.022	0.013	0.023	0.006	0.009	0.005	0.023	0.019	0.022	0.016	0.021	0.019	0.010	0.004	0.013	0.029	0.023	0.027	0.018	0.038	0.030	0.023	0.033	0.055	0.016	0.009	0.016	0.006	0.014	0.006	0.021	0.014	0.026	0.017	0.028	0.021	0.007	0.019	0.002
F332_151.007_10.6_N	0.006	0.002	0.004	0.003	0.005	0.010	0.003	0.004	0.004	0.005	0.010	0.021	0.007	0.012	0.007	0.018	0.026	0.006	0.008	0.007	0.003	0.005	0.021	0.005	0.003	0.003	0.006	0.004	0.014	0.004	0.006	0.004	0.003	0.004	0.006	0.006	0.005	0.011	0.005	0.005	0.005	0.002	0.006	0.005	0.009	0.008	0.008	0.006	0.007	0.017	0.014	0.009	0.004
F333_557.4575_3.1_N	0.005	0.003	0.007	0.007	0.011	0.008	0.002	0.007	0.012	0.008	0.008	0.017	0.010	0.011	0.015	0.011	0.008	0.008	0.004	0.005	0.008	0.012	0.010	0.015	0.015	0.013	0.020	0.016	0.013	0.010	0.009	0.018	0.010	0.008	0.008	0.012	0.013	0.007	0.009	0.011	0.007	0.004	0.009	0.008	0.005	0.006	0.005	0.009	0.005	0.008	0.007	0.008	0.002
F336_607.4731_3.1_N	0.007	0.003	0.009	0.013	0.014	0.013	0.002	0.009	0.013	0.009	0.009	0.014	0.012	0.008	0.014	0.010	0.007	0.008	0.003	0.006	0.007	0.011	0.007	0.015	0.016	0.014	0.019	0.012	0.014	0.008	0.008	0.015	0.007	0.007	0.007	0.010	0.014	0.005	0.010	0.013	0.006	0.004	0.009	0.007	0.003	0.005	0.006	0.008	0.004	0.007	0.007	0.006	0.003
F337_225.0616_17.8_N	0.019	0.031	0.020	0.039	0.015	0.019	0.015	0.030	0.025	0.024	0.018	0.021	0.025	0.016	0.026	0.013	0.025	0.029	0.012	0.021	0.027	0.023	0.019	0.025	0.030	0.017	0.013	0.024	0.009	0.017	0.023	0.027	0.012	0.022	0.028	0.023	0.036	0.031	0.006	0.006	0.004	0.015	0.005	0.007	0.005	0.005	0.017	0.008	0.020	0.008	0.007	0.005	0.023
F338_500.2798_3.8_N	0.032	0.028	0.027	0.019	0.022	0.023	0.006	0.029	0.032	0.029	0.012	0.024	0.021	0.011	0.021	0.023	0.021	0.020	0.025	0.023	0.021	0.028	0.022	0.023	0.023	0.035	0.023	0.026	0.014	0.015	0.015	0.010	0.006	0.015	0.014	0.014	0.011	0.013	0.030	0.017	0.018	0.034	0.026	0.025	0.024	0.017	0.014	0.016	0.011	0.010	0.012	0.012	0.005
F339_181.0176_8.6_N	0.004	0.002	0.001	0.001	0.002	0.011	0.003	0.001	0.001	0.002	0.007	0.040	0.002	0.012	0.003	0.013	0.012	0.003	0.005	0.003	0.001	0.004	0.008	0.002	0.001	0.001	0.003	0.003	0.019	0.003	0.003	0.001	0.001	0.003	0.003	0.003	0.002	0.004	0.006	0.006	0.010	0.005	0.007	0.005	0.012	0.009	0.003	0.002	0.002	0.008	0.007	0.002	0.002
F340_421.2263_3.1_N	0.004	0.007	0.002	0.003	0.004	0.003	0.008	0.002	0.002	0.004	0.003	0.003	0.002	0.042	0.007	0.002	0.002	0.002	0.004	0.006	0.001	0.001	0.003	0.002	0.001	0.001	0.001	0.002	0.002	0.002	0.003	0.001	0.002	0.015	0.001	0.002	0.004	0.007	0.002	0.001	0.029	0.002	0.003	0.001	0.001	0.003	0.030	0.001	0.009	0.002	0.001	0.002	0.004
F341_414.226_14.7_P	0.010	0.007	0.009	0.004	0.007	0.012	0.006	0.008	0.005	0.005	0.005	0.006	0.006	0.024	0.005	0.012	0.009	0.008	0.004	0.006	0.004	0.006	0.006	0.007	0.006	0.007	0.007	0.006	0.021	0.015	0.006	0.012	0.004	0.007	0.011	0.024	0.013	0.008	0.026	0.030	0.043	0.029	0.041	0.043	0.040	0.028	0.006	0.004	0.008	0.015	0.014	0.004	0.006
F342_236.0563_3.9_N	0.019	0.015	0.019	0.019	0.025	0.017	0.012	0.025	0.024	0.007	0.008	0.017	0.016	0.010	0.016	0.010	0.011	0.013	0.010	0.011	0.017	0.007	0.010	0.017	0.009	0.019	0.013	0.013	0.015	0.012	0.010	0.013	0.011	0.009	0.013	0.013	0.016	0.015	0.008	0.008	0.007	0.008	0.013	0.011	0.008	0.008	0.011	0.012	0.010	0.012	0.009	0.007	0.006
F344_430.2214_14.4_P	0.009	0.005	0.007	0.008	0.006	0.008	0.009	0.003	0.011	0.013	0.018	0.016	0.014	0.027	0.012	0.018	0.013	0.016	0.015	0.014	0.018	0.019	0.014	0.006	0.022	0.013	0.015	0.020	0.013	0.011	0.037	0.019	0.018	0.035	0.022	0.014	0.031	0.005	0.001	0.002	0.001	0.002	0.002	0.001	0.002	0.001	0.005	0.002	0.013	0.011	0.003	0.004	0.006
F345_649.5187_3.2_P	0.028	0.032	0.028	0.042	0.016	0.026	0.027	0.019	0.036	0.050	0.026	0.032	0.019	0.023	0.016	0.018	0.034	0.040	0.034	0.031	0.025	0.015	0.022	0.026	0.029	0.028	0.021	0.025	0.017	0.029	0.026	0.035	0.018	0.027	0.023	0.025	0.028	0.038	0.025	0.020	0.025	0.030	0.019	0.014	0.016	0.009	0.022	0.018	0.021	0.019	0.012	0.013	0.028
F346_814.5223_3.4_N	0.063	0.173	0.041	0.057	0.019	0.035	0.115	0.028	0.054	0.050	0.014	0.053	0.046	0.237	0.033	0.028	0.041	0.074	0.058	0.057	0.033	0.021	0.029	0.014	0.022	0.039	0.013	0.053	0.020	0.039	0.020	0.027	0.024	0.036	0.035	0.020	0.039	0.056	0.021	0.027	0.028	0.055	0.026	0.043	0.037	0.024	0.027	0.031	0.037	0.020	0.022	0.031	0.055
F349_161.0819_7.7_N	0.014	0.010	0.026	0.006	0.023	0.017	0.012	0.006	0.009	0.005	0.004	0.028	0.006	0.014	0.011	0.006	0.008	0.003	0.015	0.007	0.002	0.007	0.010	0.008	0.005	0.011	0.009	0.006	0.018	0.006	0.007	0.002	0.004	0.006	0.003	0.008	0.004	0.003	0.002	0.003	0.004	0.002	0.003	0.001	0.001	0.004	0.003	0.001	0.001	0.003	0.004	0.002	0.002
F351_229.1655_11.2_P	0.041	0.021	0.024	0.031	0.016	0.016	0.011	0.019	0.043	0.043	0.030	0.031	0.031	0.027	0.022	0.015	0.025	0.043	0.017	0.038	0.024	0.021	0.020	0.024	0.047	0.037	0.023	0.030	0.017	0.026	0.028	0.032	0.022	0.024	0.026	0.026	0.026	0.024	0.013	0.018	0.015	0.030	0.015	0.008	0.019	0.011	0.045	0.041	0.032	0.023	0.011	0.030	0.018
F352_224.1126_12.1_P	0.015	0.008	0.019	0.015	0.018	0.029	0.010	0.024	0.014	0.009	0.014	0.020	0.018	0.044	0.024	0.030	0.026	0.013	0.006	0.014	0.018	0.015	0.018	0.024	0.013	0.014	0.013	0.019	0.026	0.034	0.020	0.032	0.017	0.022	0.017	0.035	0.032	0.033	0.025	0.018	0.036	0.019	0.024	0.018	0.026	0.020	0.034	0.029	0.029	0.043	0.033	0.024	0.009
F353_313.1128_15.4_N	0.044	0.028	0.017	0.038	0.004	0.007	0.022	0.010	0.031	0.019	0.018	0.012	0.020	0.010	0.014	0.010	0.017	0.050	0.054	0.024	0.018	0.018	0.026	0.009	0.024	0.014	0.006	0.017	0.004	0.016	0.034	0.027	0.026	0.042	0.031	0.012	0.017	0.032	0.005	0.004	0.008	0.013	0.004	0.002	0.006	0.003	0.005	0.003	0.009	0.004	0.002	0.003	0.011

F354_853.5798_3.5_N	0.040	0.013	0.039	0.076	0.022	0.043	0.070	0.054	0.070	0.056	0.006	0.017	0.031	0.187	0.043	0.049	0.078	0.058	0.042	0.072	0.090	0.026	0.049	0.054	0.067	0.056	0.058	0.055	0.050	0.067	0.056	0.062	0.066	0.046	0.043	0.054	0.062	0.061	0.029	0.033	0.044	0.037	0.018	0.024	0.058	0.032	0.069	0.072	0.089	0.085	0.063	0.060	0.094
F356_301.1424_14.3_P	0.028	0.026	0.017	0.020	0.014	0.023	0.013	0.018	0.019	0.023	0.030	0.025	0.022	0.032	0.024	0.025	0.027	0.028	0.024	0.028	0.024	0.029	0.023	0.022	0.023	0.021	0.022	0.026	0.015	0.020	0.030	0.029	0.019	0.032	0.028	0.024	0.033	0.020	0.015	0.018	0.014	0.023	0.015	0.025	0.019	0.015	0.027	0.019	0.036	0.028	0.018	0.018	0.032
F357_439.3577_3.2_N	0.007	0.006	0.017	0.010	0.018	0.012	0.003	0.016	0.017	0.014	0.014	0.014	0.017	0.013	0.018	0.014	0.017	0.011	0.010	0.019	0.011	0.024	0.021	0.020	0.018	0.015	0.014	0.010	0.016	0.012	0.011	0.010	0.015	0.011	0.009	0.014	0.010	0.015	0.017	0.015	0.014	0.012	0.024	0.024	0.012	0.019	0.014	0.013	0.010	0.012	0.011	0.021	0.006
F358_561.4888_3.1_N	0.009	0.003	0.012	0.013	0.011	0.012	0.004	0.008	0.017	0.013	0.014	0.023	0.012	0.010	0.020	0.012	0.010	0.011	0.006	0.009	0.009	0.013	0.012	0.014	0.023	0.014	0.024	0.021	0.020	0.011	0.012	0.026	0.012	0.011	0.011	0.015	0.018	0.012	0.011	0.013	0.008	0.004	0.011	0.008	0.008	0.007	0.008	0.012	0.007	0.012	0.009	0.011	0.007
F359_244.0495_11.5_N	0.005	0.009	0.018	0.007	0.010	0.011	0.003	0.012	0.006	0.013	0.018	0.014	0.013	0.011	0.027	0.015	0.025	0.006	0.004	0.006	0.013	0.010	0.018	0.015	0.010	0.028	0.013	0.026	0.020	0.009	0.007	0.005	0.005	0.009	0.004	0.011	0.007	0.012	0.004	0.004	0.005	0.004	0.007	0.005	0.006	0.007	0.008	0.006	0.004	0.006	0.006	0.006	0.002
F360_231.0595_16.4_N	0.031	0.019	0.017	0.038	0.009	0.012	0.012	0.011	0.024	0.033	0.016	0.010	0.011	0.009	0.010	0.006	0.015	0.043	0.032	0.024	0.015	0.010	0.011	0.013	0.016	0.009	0.008	0.011	0.007	0.013	0.018	0.018	0.021	0.032	0.018	0.010	0.009	0.015	0.008	0.005	0.011	0.023	0.008	0.003	0.012	0.005	0.006	0.003	0.010	0.008	0.005	0.006	0.007
F361_164.0023_9.4_N	0.006	0.005	0.008	0.014	0.012	0.013	0.003	0.015	0.019	0.010	0.020	0.014	0.017	0.013	0.008	0.021	0.028	0.016	0.010	0.008	0.017	0.018	0.019	0.015	0.023	0.024	0.022	0.014	0.017	0.005	0.026	0.026	0.027	0.038	0.007	0.014	0.021	0.041	0.002	0.006	0.005	0.004	0.004	0.005	0.007	0.008	0.011	0.012	0.010	0.009	0.011	0.010	0.003
F362_145.0869_4.3_N	0.007	0.004	0.008	0.005	0.016	0.014	0.008	0.013	0.010	0.004	0.010	0.011	0.014	0.024	0.018	0.014	0.013	0.006	0.006	0.011	0.012	0.014	0.010	0.014	0.011	0.013	0.013	0.008	0.014	0.016	0.014	0.012	0.013	0.014	0.008	0.011	0.016	0.020	0.021	0.019	0.015	0.010	0.018	0.018	0.014	0.019	0.007	0.010	0.008	0.006	0.007	0.007	0.003
F363_570.179_16.1_P	0.059	0.004	0.028	0.065	0.006	0.011	0.162	0.010	0.030	0.061	0.024	0.025	0.014	0.017	0.014	0.010	0.023	0.051	0.080	0.039	0.015	0.010	0.015	0.012	0.022	0.014	0.009	0.027	0.006	0.021	0.022	0.018	0.016	0.030	0.033	0.018	0.014	0.040	0.012	0.004	0.011	0.067	0.008	0.007	0.016	0.005	0.033	0.022	0.044	0.027	0.009	0.019	0.124
F364_788.5222_3.2_P	0.033	0.036	0.024	0.023	0.008	0.020	0.032	0.013	0.029	0.057	0.018	0.022	0.012	0.096	0.018	0.010	0.031	0.040	0.052	0.050	0.020	0.018	0.023	0.019	0.024	0.014	0.011	0.011	0.010	0.020	0.030	0.017	0.021	0.044	0.026	0.019	0.017	0.033	0.018	0.012	0.022	0.034	0.014	0.011	0.020	0.013	0.022	0.013	0.021	0.012	0.004	0.018	0.064
F365_202.0818_13.1_P	0.022	0.007	0.014	0.013	0.021	0.021	0.008	0.016	0.014	0.008	0.008	0.028	0.025	0.032	0.032	0.036	0.015	0.023	0.007	0.007	0.009	0.023	0.017	0.023	0.018	0.006	0.025	0.024	0.024	0.027	0.017	0.037	0.021	0.020	0.023	0.025	0.039	0.018	0.016	0.011	0.007	0.007	0.009	0.011	0.010	0.003	0.011	0.010	0.013	0.027	0.019	0.014	0.011
F366_862.5537_3.1_N	0.011	0.013	0.011	0.022	0.006	0.017	0.025	0.011	0.016	0.024	0.017	0.014	0.013	0.032	0.013	0.009	0.019	0.020	0.012	0.015	0.005	0.012	0.019	0.014	0.019	0.013	0.013	0.012	0.010	0.024	0.014	0.017	0.013	0.019	0.021	0.031	0.021	0.024	0.014	0.006	0.015	0.017	0.008	0.008	0.010	0.008	0.013	0.010	0.011	0.014	0.011	0.008	0.020
F369_189.1132_4.5_N	0.000	0.003	0.005	0.000	0.007	0.006	0.002	0.007	0.008	0.003	0.008	0.006	0.010	0.006	0.006	0.010	0.016	0.007	0.001	0.005	0.009	0.008	0.011	0.010	0.012	0.011	0.009	0.011	0.009	0.006	0.013	0.013	0.012	0.020	0.003	0.010	0.008	0.023	0.002	0.003	0.003	0.001	0.002	0.003	0.002	0.004	0.003	0.003	0.004	0.002	0.003	0.003	0.001
F390_330.2269_4.5_P	0.002	0.003	0.007	0.002	0.005	0.008	0.005	0.006	0.006	0.003	0.010	0.010	0.006	0.011	0.008	0.007	0.008	0.002	0.002	0.002	0.005	0.007	0.009	0.007	0.009	0.004	0.005	0.006	0.010	0.010	0.010	0.008	0.009	0.007	0.009	0.012	0.006	0.008	0.005	0.006	0.006	0.002	0.006	0.007	0.008	0.008	0.008	0.004	0.009	0.011	0.018	0.021	0.003
F394_357.1683_13.3_P	0.015	0.007	0.013	0.015	0.010	0.016	0.008	0.014	0.019	0.013	0.012	0.014	0.017	0.034	0.015	0.018	0.019	0.020	0.014	0.016	0.013	0.011	0.018	0.013	0.015	0.008	0.012	0.018	0.016	0.017	0.017	0.019	0.012	0.016	0.015	0.017	0.022	0.022	0.014	0.010	0.013	0.017	0.012	0.012	0.023	0.010	0.019	0.018	0.023	0.024	0.015	0.014	0.009
F395_386.1177_10.5_P	0.009	0.005	0.014	0.010	0.016	0.019	0.004	0.015	0.011	0.009	0.002	0.015	0.010	0.018	0.010	0.011	0.010	0.012	0.007	0.010	0.006	0.006	0.008	0.014	0.010	0.011	0.008	0.008	0.009	0.013	0.008	0.010	0.003	0.008	0.007	0.015	0.014	0.011	0.023	0.020	0.024	0.022	0.026	0.025	0.015	0.022	0.031	0.032	0.030	0.041	0.027	0.018	0.013
F399_417.2355_4.5_N	0.000	0.001	0.004	0.000	0.003	0.002	0.001	0.004	0.002	0.003	0.002	0.001	0.006	0.004	0.004	0.001	0.006	0.000	0.000	0.000	0.006	0.004	0.007	0.017	0.006	0.016	0.004	0.013	0.011	0.008	0.009	0.010	0.011	0.007	0.003	0.007	0.006	0.007	0.005	0.004	0.008	0.000	0.006	0.003	0.014	0.005	0.012	0.019	0.006	0.004	0.005	0.012	0.001
F403_149.0466_11.7_N	0.026	0.021	0.026	0.027	0.015	0.022	0.027	0.020	0.023	0.028	0.025	0.018	0.027	0.129	0.024	0.022	0.022	0.024	0.027	0.023	0.036	0.014	0.019	0.019	0.022	0.025	0.024	0.021	0.012	0.020	0.024	0.019	0.024	0.026	0.020	0.001	0.007	0.016	0.051	0.067	0.065	0.051	0.060	0.075	0.048	0.069	0.000	0.010	0.027	0.004	0.024	0.022	0.032
F407_327.2315_3.1_P	0.009	0.009	0.010	0.013	0.011	0.012	0.008	0.010	0.014	0.013	0.009	0.019	0.012	0.018	0.017	0.012	0.009	0.014	0.006	0.007	0.008	0.012	0.010	0.015	0.017	0.015	0.017	0.014	0.012	0.014	0.011	0.017	0.008	0.007	0.009	0.012	0.016	0.009	0.009	0.011	0.007	0.006	0.009	0.009	0.006	0.006	0.007	0.008	0.006	0.008	0.005	0.007	0.007
F409_196.0979_4.3_N	0.002	0.001	0.001	0.001	0.001	0.004	0.003	0.001	0.001	0.001	0.001	0.008	0.000	0.007	0.000	0.002	0.001	0.001	0.003	0.002	0.000	0.001	0.001	0.000	0.001	0.000	0.001	0.001	0.004	0.003	0.001	0.000	0.001	0.001	0.001	0.004	0.001	0.001	0.009	0.012	0.015	0.004	0.005	0.004	0.006	0.005	0.002	0.002	0.001	0.003	0.002	0.001	0.004
F410_171.0661_4.5_N	0.005	0.015	0.015	0.006	0.009	0.008	0.017	0.009	0.016	0.012	0.012	0.013	0.010	0.072	0.011	0.011	0.012	0.005	0.007	0.004	0.011	0.010	0.021	0.009	0.014	0.013	0.011	0.015	0.008	0.014	0.012	0.012	0.010	0.017	0.014	0.012	0.016	0.023	0.008	0.006	0.008	0.004	0.008	0.007	0.009	0.006	0.012	0.009	0.016	0.010	0.008	0.008	0.011
F412_216.0873_4.5_N	0.000	0.001	0.003	0.000	0.002	0.007	0.001	0.002	0.003	0.000	0.003	0.003	0.002	0.003	0.003	0.003	0.003	0.000	0.000	0.002	0.002	0.002	0.004	0.004	0.003	0.003	0.003	0.003	0.007	0.003	0.002	0.002	0.002	0.004	0.003	0.006	0.002	0.002	0.007	0.008	0.014	0.000	0.007	0.010	0.008	0.008	0.002	0.003	0.002	0.004	0.013	0.002	0.000
F414_584.3579_3.8_N	0.010	0.012	0.062	0.028	0.010	0.011	0.003	0.010	0.007	0.005	0.009	0.019	0.014	0.014	0.004	0.009	0.001	0.003	0.010	0.013	0.007	0.010	0.012	0.010	0.008	0.064	0.019	0.016	0.003	0.006	0.009	0.010	0.012	0.008	0.002	0.075	0.011	0.006	0.014	0.037	0.008	0.012	0.008	0.008	0.002	0.011	0.019	0.006	0.006	0.003	0.009	0.002	0.008
F416_578.514_3.1_P	0.010	0.005	0.010	0.008	0.006	0.006	0.005	0.004	0.013	0.011	0.007	0.017	0.008	0.016	0.013	0.009	0.007	0.010	0.007	0.005	0.004	0.006	0.007	0.009	0.015	0.010	0.014	0.016	0.010	0.007	0.006	0.016	0.006	0.007	0.007	0.010	0.012	0.008	0.006	0.007	0.005	0.004	0.005	0.006	0.005	0.004	0.005	0.008	0.004	0.006	0.005	0.006	0.007
F418_379.3582_3.2_N	0.008	0.007	0.013	0.010	0.013	0.017	0.005	0.016	0.019	0.013	0.017	0.011	0.021	0.013	0.016	0.017	0.020	0.013	0.008	0.017	0.015	0.020	0.018	0.028	0.018	0.013	0.018	0.012	0.017	0.014	0.016	0.019	0.015	0.013	0.009	0.017	0.014	0.013	0.005	0.007	0.006	0.003	0.007	0.007	0.004	0.008	0.021	0.014	0.020	0.016	0.018	0.021	0.006
F419_321.2416_3.1_P	0.018	0.012	0.011	0.020	0.017	0.019	0.015	0.020	0.022	0.026	0.021	0.020	0.039	0.021	0.034	0.034	0.019	0.024	0.017	0.013	0.024	0.025	0.019	0.028	0.023	0.028	0.037	0.028	0.031	0.030	0.027	0.021	0.023	0.019	0.021	0.027	0.034	0.017	0.016	0.025	0.016	0.024	0.028	0.034	0.019	0.025	0.030	0.023	0.031	0.030	0.019	0.032	0.024
F420_409.1719_16.2_P	0.027	0.006	0.038	0.031	0.022	0.029	0.010	0.027	0.046	0.030	0.042	0.031	0.033	0.019	0.030	0.023	0.031	0.033	0.030	0.036	0.034	0.026	0.025	0.031	0.040	0.024	0.029	0.025	0.024	0.028	0.042	0.046	0.047	0.053	0.057	0.034	0.031	0.035	0.036	0.024	0.038	0.040	0.037	0.027	0.038	0.025	0.038	0.019	0.029	0.025	0.026	0.029	0.014
F421_461.1668_10.5_P	0.021	0.010	0.009	0.018	0.017	0.015	0.006	0.013	0.005	0.011	0.014	0.007	0.008	0.030	0.008	0.005	0.009	0.012	0.004	0.003	0.007	0.007	0.007	0.012	0.003	0.016	0.003	0.011	0.005	0.012	0.008	0.010	0.003	0.007	0.008	0.019	0.009	0.010	0.017	0.015	0.026	0.035	0.017	0.018	0.028	0.014	0.054	0.048	0.041	0.047	0.022	0.036	0.041
F422_548.2957_10_P	0.025	0.008	0.038	0.025	0.029	0.023	0.007	0.029	0.041	0.027	0.026	0.032	0.036	0.029	0.038	0.030	0.038	0.034	0.020	0.044	0.033	0.030	0.028	0.025	0.040	0.031	0.028	0.033	0.028	0.032	0.040	0.036	0.032	0.047	0.041	0.027	0.037	0.032	0.038	0.031	0.029	0.042	0.019	0.029	0.034	0.024	0.037	0.027	0.036	0.032	0.030	0.027	0.007
F423_325.2369_3.5_P	0.020	0.026	0.015	0.022	0.014	0.015	0.043	0.022	0.017	0.018	0.015	0.012	0.019	0.080	0.023	0.015	0.012	0.018	0.023	0.017	0.014	0.016	0.016	0.023	0.014	0.013	0.012	0.016	0.012	0.017	0.013	0.014	0.014	0.015	0.015	0.014	0.020	0.024	0.040	0.059	0.028	0.023	0.036	0.072	0.014	0.028	0.010	0.022	0.013	0.012	0.012	0.013	0.035
F425_295.0657_10.4_P	0.012	0.007	0.043	0.012	0.064	0.067	0.007	0.048	0.027	0.017	0.019	0.022	0.011	0.031	0.017	0.036	0.022	0.004	0.006	0.015	0.017	0.011	0.015	0.031	0.024	0.031	0.043	0.039	0.029	0.028	0.021	0.022	0.018	0.020	0.037	0.055	0.039	0.028	0.145	0.125	0.110	0.104	0.174	0.133	0.176	0.182	0.113	0.103	0.197	0.210	0.177	0.090	0.055
F429_253.0501_11_N	0.020	0.014	0.010	0.020	0.020	0.028	0.012	0.019	0.015	0.011	0.017	0.016	0.029	0.013	0.029	0.033	0.023	0.025	0.015	0.016	0.013	0.024	0.021	0.022	0.015	0.024	0.023	0.021	0.029	0.022	0.029	0.029	0.019	0.021	0.022	0.030	0.039	0.027	0.026	0.030	0.032	0.017	0.033	0.025	0.018	0.014	0.019	0.016	0.019	0.035	0.036	0.019	0.023
F430_407.1192_13.8_N	0.064	0.102	0.117	0.117	0.106	0.088	0.054	0.129	0.110	0.092	0.129	0.103	0.125	0.013	0.132	0.108	0.135	0.118	0.084	0.088	0.145	0.094	0.084	0.119	0.131	0.128	0.126	0.117	0.070	0.093	0.096	0.117	0.128	0.096	0.090	0.096	0.122	0.162	0.101	0.061	0.048	0.059	0.081	0.073	0.061	0.055	0.102	0.060	0.101	0.073	0.059	0.070	0.100
F431_514.311_10.7_P	0.046	0.034	0.043	0.060	0.010	0.014	0.021	0.028	0.057	0.049	0.018	0.031	0.045	0.030	0.033	0.028	0.052	0.078	0.071	0.070	0.052	0.037	0.039	0.018	0.031	0.029	0.010	0.038	0.018	0.041	0.066	0.047	0.059	0.056	0.066	0.034	0.048	0.081	0.008	0.005	0.025	0.087	0.027	0.027	0.044	0.018	0.054	0.037	0.064	0.044	0.034	0.035	0.052
F432_718.5365_3.5_P	0.088	0.173	0.057	0.097	0.038	0.055	0.092	0.068	0.078	0.118	0.056	0.065	0.056	0.120	0.040	0.039	0.050	0.106	0.105	0.123	0.066	0.040	0.050	0.038	0.069	0.039	0.061	0.062	0.040	0.072	0.113	0.069	0.056	0.098	0.108	0.050	0.069	0.102	0.034	0.032	0.033	0.069	0.031	0.032	0.067	0.035	0.060	0.066	0.109	0.063	0.066	0.048	0.127
F433_346.1187_10.4_N	0.015	0.007	0.020	0.014	0.021	0.021	0.007	0.017	0.019	0.017	0.011	0.013	0.008	0.014	0.010	0.014	0.014	0.009	0.013	0.012	0.011	0.007	0.009	0.016	0.014	0.016	0.015	0.021	0.013	0.014	0.015	0.013	0.010	0.014	0.019	0.020	0.015	0.017	0.033	0.030	0.028	0.044	0.032	0.023	0.033	0.030	0.035	0.032	0.052	0.044	0.038	0.025	0.043
F434_128.0353_15.3_N	0.067	0.033	0.059	0.049	0.111	0.084	0.042	0.094	0.069	0.159	0.082	0.106	0.079	0.046	0.073	0.089	0.080	0.057	0.070	0.055	0.085	0.077	0.080	0.067	0.149	0.066	0.160	0.119	0.120	0.083	0.066	0.081	0.082	0.099	0.072	0.087	0.094	0.043	0.063	0.076	0.074	0.045	0.074	0.050	0.061	0.086	0.074	0.123	0.072	0.061	0.100	0.082	0.073
F435_625.5189_3.1_P	0.037	0.051	0.034	0.060	0.030	0.041	0.053	0.034	0.044	0.077	0.031	0.046	0.033	0.053	0.042	0.029	0.034	0.047	0.039	0.033	0.029	0.027	0.028	0.037	0.047	0.041	0.042	0.040	0.029	0.035	0.036	0.043	0.027	0.037	0.036	0.037	0.047	0.045	0.031	0.018	0.026	0.027	0.021	0.025	0.020	0.016	0.028	0.026	0.043	0.029	0.020	0.020	0.060
F437_429.1007_13.8_N	0.043	0.054	0.033	0.067	0.011	0.013	0.023	0.018	0.034	0.037	0.022	0.019	0.020	0.008	0.019	0.023	0.023	0.045	0.036	0.030	0.024	0.016	0.020	0.022	0.027	0.025	0.014	0.047	0.006	0.018	0.020	0.023	0.026	0.022	0.020	0.015	0.017	0.044	0.016	0.009	0.009	0.026	0.013	0.016	0.014	0.007	0.023	0.013	0.024	0.016	0.006	0.012	0.038
F438_167.0925_7.3_P	0.001	0.002	0.002	0.001	0.002	0.003	0.001	0.001	0.001	0.001	0.002	0.002	0.001	0.007	0.002	0.001	0.003	0.001	0.001	0.000	0.000	0.001	0.002	0.001	0.000	0.001	0.001	0.000	0.007	0.001	0.001	0.001	0.001	0.001	0.001	0.002	0.001	0.001	0.023	0.014	0.033	0.001	0.017	0.014	0.025	0.022	0.001	0.002	0.001	0.003	0.002	0.001	0.001
F439_261.0282_17.5_N	0.006	0.004	0.006	0.005	0.007	0.012	0.002	0.012	0.007	0.004	0.007	0.008	0.010	0.008	0.008	0.011	0.012	0.007	0.007	0.006	0.006	0.007	0.013	0.008	0.005	0.006	0.010	0.006	0.013	0.009	0.011	0.010	0.006	0.008	0.011	0.011	0.013	0.011	0.011	0.010	0.015	0.008	0.020	0.011	0.025	0.017	0.006	0.007	0.010	0.022	0.015	0.014	0.005
F440_504.2443_4.4_P	0.002	0.003	0.027	0.022	0.040	0.037	0.006	0.041	0.021	0.012	0.029	0.012	0.025	0.013	0.024	0.024	0.019	0.002	0.002	0.011	0.031	0.029	0.020	0.024	0.024	0.029	0.020	0.027	0.029	0.018	0.019	0.018	0.031	0.016	0.021	0.031	0.018	0.023	0.009	0.009	0.016	0.002	0.016	0.010	0.020	0.012	0.025	0.035	0.015	0.020	0.014	0.028	0.003
F441_549.298_14.1_N	0.004	0.006	0.033	0.006	0.026	0.018	0.007	0.033	0.027	0.008	0.013	0.003	0.086	0.033	0.033	0.004	0.039	0.029	0.003	0.026	0.024	0.015	0.049	0.075	0.020	0.084	0.036	0.008	0.015	0.008	0.021	0.024	0.013	0.017	0.016	0.007	0.034	0.017	0.028	0.017	0.024	0.003	0.032	0.026	0.058	0.034	0.004	0.015	0.005	0.002	0.016	0.031	0.005
F442_331.191_4.3_N	0.012	0.005	0.001	0.002	0.010	0.001	0.009	0.029	0.002	0.004	0.015	0.009	0.015	0.018	0.001	0.009	0.001	0.024	0.002	0.031	0.001	0.021	0.042	0.001	0.006	0.001	0.002	0.011	0.001	0.003	0.018	0.022	0.020	0.032	0.036	0.001	0.008	0.001	0.001	0.002	0.001	0.002	0.001	0.003	0.001	0.001	0.001	0.016	0.001	0.019	0.028	0.003	0.003
F443_472.3459_3.3_N	0.004	0.007	0.006	0.006	0.035	0.007	0.006	0.010	0.006	0.008	0.006	0.006	0.007	0.018	0.010	0.007	0.010	0.004	0.004	0.005	0.016	0.005	0.005	0.009	0.008	0.012	0.011	0.009	0.006	0.005	0.006	0.009	0.020	0.004	0.004	0.005	0.013	0.006	0.010	0.007	0.016	0.003	0.004	0.006	0.005	0.006	0.007	0.007	0.006	0.006	0.008	0.016	0.004
F444_774.1184_14.3_N	0.004	0.002	0.006	0.001	0.013	0.013	0.003	0.013	0.006	0.007	0.014	0.012	0.013	0.013	0.010	0.015	0.011	0.002	0.002	0.008	0.009	0.010	0.009	0.018	0.011	0.015	0.019	0.020	0.021	0.022	0.011	0.014	0.013	0.007	0.007	0.020	0.011	0.012	0.016	0.022	0.021	0.015	0.020	0.020	0.022	0.021	0.035	0.039	0.040	0.036	0.032	0.044	0.007
F445_250.0795_10.3_P	0.014	0.007	0.009	0.017	0.020	0.016	0.005	0.017	0.003	0.008	0.006	0.006	0.009	0.026	0.011	0.007	0.008	0.009	0.003	0.002	0.002	0.006	0.007	0.018	0.005	0.019	0.005	0.013	0.008	0.012	0.006	0.010	0.002	0.007	0.007	0.021	0.009	0.008	0.024	0.024	0.029	0.034	0.027	0.025	0.031	0.035	0.055	0.052	0.049	0.066	0.037	0.043	0.038
F446_344.156_11_P	0.004	0.011	0.004	0.004	0.021	0.018	0.006	0.008	0.005	0.005	0.012	0.013	0.008	0.028	0.005	0.009	0.005	0.003	0.004	0.004	0.003	0.005	0.009	0.012	0.011	0.010	0.020	0.009	0.021	0.010	0.003	0.004	0.005	0.005	0.004	0.011	0.014	0.005	0.039	0.038	0.023	0.004	0.030	0.025	0.027	0.027	0.007	0.008	0.003	0.009	0.015	0.007	0.006
F448_214.0583_16.3_P	0.018	0.004	0.023	0.018	0.020	0.026	0.004	0.021	0.028	0.017	0.029	0.020	0.024	0.016	0.022	0.019	0.023	0.019	0.017	0.020	0.026	0.022	0.019	0.027	0.028	0.021	0.029	0.018	0.028	0.019	0.027	0.037	0.041	0.037	0.032	0.026	0.022	0.020	0.030	0.025	0.034	0.023	0.034	0.024	0.028	0.026	0.023	0.016	0.020	0.021	0.025	0.027	0.007
F450_423.0901_11.1_N	0.002	0.002	0.004	0.002	0.006	0.005	0.002	0.006	0.002	0.003	0.004	0.007	0.002	0.011	0.004	0.007	0.007	0.004	0.008	0.004	0.003	0.005	0.013	0.008	0.004	0.004	0.004	0.006	0.012	0.014	0.014	0.031	0.015	0.034	0.020	0.023	0.008	0.013	0.013	0.013	0.020	0.015	0.026	0.022	0.028	0.035	0.021	0.035	0.017	0.035	0.037	0.034	0.004

F451_350.2776_3.2_N	0.075	0.038	0.082	0.099	0.147	0.142	0.022	0.169	0.116	0.075	0.090	0.082	0.129	0.016	0.141	0.108	0.136	0.072	0.052	0.106	0.099	0.087	0.089	0.186	0.158	0.149	0.129	0.084	0.153	0.127	0.074	0.135	0.103	0.067	0.060	0.085	0.089	0.072	0.067	0.061	0.056	0.031	0.064	0.057	0.053	0.050	0.075	0.109	0.073	0.083	0.081	0.113	0.036
F453_145.097_7.4_P	0.016	0.049	0.032	0.030	0.040	0.049	0.054	0.043	0.033	0.020	0.029	0.035	0.044	0.232	0.037	0.044	0.029	0.030	0.017	0.027	0.023	0.031	0.027	0.041	0.028	0.029	0.035	0.025	0.037	0.032	0.031	0.045	0.020	0.031	0.023	0.043	0.062	0.036	0.049	0.035	0.028	0.012	0.036	0.049	0.026	0.029	0.028	0.028	0.024	0.037	0.034	0.024	0.037
F454_301.1392_11_P	0.022	0.013	0.028	0.024	0.018	0.020	0.009	0.026	0.027	0.020	0.032	0.040	0.029	0.031	0.043	0.035	0.035	0.032	0.024	0.032	0.042	0.037	0.030	0.034	0.034	0.042	0.035	0.040	0.021	0.042	0.059	0.038	0.039	0.035	0.055	0.044	0.051	0.053	0.027	0.023	0.035	0.040	0.043	0.027	0.034	0.036	0.023	0.038	0.049	0.037	0.044	0.038	0.022
F457_330.0688_11_N	0.063	0.064	0.041	0.056	0.050	0.036	0.006	0.035	0.041	0.040	0.044	0.030	0.039	0.021	0.033	0.026	0.049	0.052	0.062	0.052	0.034	0.030	0.032	0.023	0.040	0.055	0.030	0.040	0.046	0.056	0.062	0.048	0.067	0.054	0.051	0.041	0.053	0.068	0.034	0.023	0.017	0.098	0.043	0.026	0.075	0.019	0.050	0.041	0.058	0.059	0.003	0.006	0.097
F458_228.0799_9.5_P	0.004	0.006	0.008	0.002	0.007	0.013	0.007	0.011	0.003	0.002	0.002	0.009	0.005	0.030	0.005	0.008	0.010	0.005	0.003	0.007	0.003	0.006	0.006	0.009	0.002	0.009	0.013	0.003	0.013	0.010	0.002	0.006	0.001	0.004	0.003	0.012	0.009	0.005	0.036	0.034	0.038	0.025	0.033	0.038	0.032	0.037	0.003	0.004	0.002	0.007	0.010	0.001	0.005
F459_500.2956_12.6_P	0.073	0.032	0.043	0.063	0.020	0.032	0.018	0.027	0.054	0.060	0.039	0.044	0.046	0.049	0.042	0.038	0.050	0.082	0.064	0.059	0.042	0.037	0.036	0.034	0.043	0.041	0.026	0.050	0.029	0.043	0.056	0.060	0.039	0.055	0.068	0.054	0.050	0.071	0.036	0.025	0.035	0.071	0.028	0.036	0.042	0.026	0.062	0.039	0.046	0.048	0.028	0.045	0.056
F460_535.2822_3.1_N	0.021	0.011	0.036	0.035	0.029	0.043	0.013	0.043	0.042	0.040	0.028	0.026	0.047	0.011	0.046	0.034	0.038	0.027	0.026	0.030	0.035	0.054	0.035	0.034	0.028	0.029	0.029	0.021	0.023	0.075	0.054	0.051	0.048	0.059	0.038	0.033	0.063	0.091	0.022	0.022	0.035	0.022	0.037	0.022	0.036	0.043	0.033	0.036	0.031	0.028	0.029	0.037	0.018
F461_248.0639_9.9_P	0.002	0.006	0.001	0.002	0.001	0.001	0.004	0.001	0.002	0.002	0.002	0.002	0.001	0.021	0.001	0.001	0.001	0.002	0.003	0.002	0.001	0.001	0.001	0.001	0.001	0.001	0.001	0.001	0.001	0.001	0.002	0.004	0.001	0.003	0.004	0.008	0.006	0.007	0.003	0.002	0.005	0.004	0.005	0.001	0.003	0.002	0.024	0.016	0.049	0.043	0.028	0.023	0.041
F464_472.2815_4.3_P	0.085	0.024	0.063	0.043	0.035	0.035	0.013	0.071	0.005	0.023	0.016	0.015	0.018	0.020	0.015	0.015	0.023	0.030	0.012	0.017	0.023	0.012	0.005	0.045	0.020	0.026	0.020	0.018	0.029	0.030	0.005	0.016	0.016	0.025	0.013	0.020	0.011	0.015	0.025	0.020	0.023	0.034	0.041	0.029	0.022	0.012	0.018	0.071	0.031	0.038	0.012	0.042	0.012
F465_235.0392_10.5_N	0.013	0.008	0.018	0.011	0.018	0.018	0.003	0.021	0.020	0.010	0.018	0.023	0.026	0.012	0.026	0.022	0.015	0.013	0.009	0.012	0.018	0.019	0.020	0.028	0.023	0.015	0.028	0.020	0.023	0.016	0.029	0.026	0.026	0.017	0.017	0.024	0.025	0.026	0.030	0.027	0.022	0.011	0.021	0.021	0.021	0.021	0.026	0.021	0.027	0.031	0.039	0.034	0.009
F466_371.084_13.7_N	0.001	0.003	0.020	0.013	0.037	0.031	0.003	0.017	0.028	0.025	0.033	0.045	0.031	0.012	0.036	0.002	0.037	0.016	0.013	0.022	0.025	0.020	0.018	0.017	0.036	0.023	0.038	0.001	0.030	0.029	0.026	0.039	0.030	0.036	0.032	0.031	0.054	0.030	0.019	0.018	0.010	0.007	0.006	0.004	0.009	0.008	0.028	0.020	0.029	0.017	0.028	0.016	0.024
F467_351.1249_13.6_P	0.037	0.015	0.035	0.028	0.021	0.034	0.017	0.035	0.026	0.028	0.027	0.033	0.035	0.070	0.024	0.048	0.050	0.050	0.047	0.048	0.027	0.030	0.045	0.026	0.025	0.022	0.020	0.030	0.048	0.047	0.035	0.035	0.033	0.057	0.107	0.043	0.030	0.064	0.035	0.025	0.053	0.105	0.041	0.022	0.048	0.036	0.042	0.034	0.046	0.050	0.055	0.041	0.033
F468_332.0958_11.3_P	0.079	0.017	0.053	0.059	0.034	0.043	0.429	0.035	0.081	0.014	0.055	0.056	0.040	0.033	0.041	0.035	0.042	0.014	0.259	0.067	0.055	0.039	0.047	0.035	0.049	0.034	0.033	0.060	0.017	0.045	0.067	0.054	0.057	0.069	0.057	0.055	0.062	0.083	0.051	0.043	0.048	0.112	0.050	0.040	0.059	0.040	0.081	0.051	0.084	0.055	0.037	0.039	0.037
F469_307.0645_13.2_P	0.003	0.007	0.002	0.002	0.001	0.001	0.007	0.001	0.002	0.003	0.002	0.002	0.001	0.029	0.002	0.002	0.002	0.003	0.004	0.002	0.002	0.002	0.002	0.002	0.002	0.002	0.002	0.002	0.002	0.002	0.002	0.002	0.002	0.002	0.002	0.003	0.002	0.005	0.017	0.025	0.014	0.028	0.019	0.029	0.027	0.015	0.019	0.009	0.026	0.026	0.020	0.007	0.026
F471_293.0099_15.1_N	0.049	0.008	0.029	0.027	0.011	0.015	0.024	0.013	0.034	0.031	0.020	0.024	0.017	0.006	0.013	0.011	0.021	0.047	0.040	0.040	0.022	0.018	0.024	0.011	0.026	0.016	0.014	0.030	0.006	0.019	0.028	0.024	0.029	0.029	0.036	0.022	0.020	0.024	0.009	0.007	0.012	0.030	0.013	0.008	0.017	0.012	0.024	0.012	0.028	0.026	0.015	0.014	0.014
F472_247.1287_14_P	0.011	0.014	0.011	0.007	0.015	0.027	0.013	0.012	0.008	0.005	0.006	0.015	0.013	0.053	0.016	0.019	0.012	0.012	0.007	0.009	0.005	0.015	0.014	0.017	0.007	0.013	0.017	0.009	0.018	0.015	0.009	0.015	0.007	0.008	0.008	0.013	0.022	0.009	0.020	0.030	0.024	0.012	0.030	0.032	0.010	0.013	0.008	0.008	0.007	0.013	0.012	0.008	0.005
F473_178.0143_14.4_P	0.095	0.135	0.075	0.100	0.056	0.041	0.128	0.064	0.080	0.105	0.057	0.043	0.078	0.094	0.064	0.042	0.064	0.110	0.120	0.088	0.092	0.037	0.055	0.047	0.063	0.055	0.061	0.094	0.032	0.061	0.071	0.044	0.102	0.094	0.074	0.065	0.121	0.066	0.039	0.039	0.051	0.155	0.042	0.034	0.057	0.044	0.111	0.102	0.075	0.061	0.048	0.057	0.260
F474_221.1546_3.5_N	0.010	0.007	0.008	0.007	0.019	0.014	0.010	0.011	0.009	0.010	0.010	0.016	0.006	0.026	0.013	0.014	0.010	0.005	0.003	0.006	0.006	0.006	0.004	0.012	0.014	0.008	0.018	0.008	0.012	0.026	0.005	0.004	0.010	0.003	0.005	0.013	0.004	0.005	0.009	0.011	0.009	0.007	0.019	0.014	0.010	0.017	0.015	0.009	0.018	0.011	0.013	0.033	0.011
F475_373.1065_13.7_P	0.020	0.008	0.020	0.014	0.010	0.017	0.006	0.019	0.014	0.014	0.015	0.017	0.020	0.028	0.013	0.027	0.027	0.026	0.024	0.025	0.015	0.016	0.024	0.017	0.014	0.013	0.011	0.017	0.019	0.025	0.018	0.019	0.017	0.028	0.051	0.023	0.014	0.035	0.019	0.011	0.023	0.046	0.018	0.012	0.021	0.014	0.023	0.016	0.024	0.025	0.021	0.021	0.014
F476_327.2901_3.2_N	0.004	0.006	0.006	0.006	0.008	0.002	0.002	0.014	0.016	0.006	0.020	0.005	0.002	0.008	0.002	0.004	0.005	0.006	0.007	0.005	0.021	0.005	0.006	0.007	0.009	0.006	0.005	0.007	0.006	0.003	0.003	0.004	0.004	0.004	0.003	0.031	0.005	0.007	0.029	0.006	0.005	0.031	0.007	0.007	0.002	0.008	0.022	0.004	0.004	0.003	0.002	0.003	0.006
F477_326.1286_15.4_N	0.084	0.152	0.027	0.092	0.009	0.014	0.168	0.024	0.043	0.043	0.034	0.016	0.037	0.168	0.028	0.028	0.032	0.111	0.175	0.038	0.034	0.054	0.077	0.016	0.055	0.020	0.014	0.035	0.021	0.037	0.071	0.048	0.057	0.087	0.055	0.025	0.033	0.062	0.008	0.008	0.014	0.038	0.009	0.005	0.011	0.008	0.006	0.006	0.012	0.005	0.006	0.009	0.089
F478_581.3981_3.2_P	0.005	0.080	0.021	0.005	0.029	0.002	0.070	0.002	0.022	0.022	0.015	0.020	0.002	0.025	0.002	0.018	0.018	0.004	0.018	0.023	0.024	0.004	0.008	0.022	0.002	0.002	0.016	0.010	0.007	0.004	0.009	0.029	0.011	0.007	0.003	0.013	0.003	0.028	0.020	0.002	0.013	0.015	0.008	0.021	0.003	0.002	0.007	0.002	0.006	0.003	0.008	0.002	0.013
F479_173.0917_14.6_P	0.044	0.036	0.039	0.041	0.037	0.041	0.007	0.057	0.053	0.043	0.040	0.031	0.056	0.032	0.040	0.042	0.052	0.055	0.058	0.066	0.058	0.046	0.046	0.036	0.052	0.021	0.036	0.046	0.052	0.045	0.068	0.049	0.081	0.050	0.051	0.058	0.058	0.097	0.022	0.022	0.021	0.029	0.027	0.021	0.019	0.024	0.054	0.035	0.048	0.048	0.034	0.043	0.054
F480_680.48_3.2_P	0.029	0.038	0.014	0.028	0.008	0.010	0.037	0.013	0.021	0.018	0.017	0.027	0.013	0.093	0.013	0.011	0.024	0.036	0.046	0.026	0.031	0.019	0.023	0.009	0.019	0.011	0.012	0.015	0.012	0.028	0.028	0.030	0.024	0.039	0.045	0.021	0.023	0.062	0.015	0.010	0.015	0.020	0.012	0.009	0.026	0.011	0.023	0.019	0.030	0.021	0.008	0.016	0.057
F481_103.0542_10.1_P	0.018	0.007	0.027	0.015	0.034	0.031	0.007	0.032	0.028	0.016	0.029	0.027	0.034	0.030	0.035	0.033	0.031	0.023	0.015	0.027	0.034	0.033	0.025	0.026	0.026	0.028	0.031	0.024	0.033	0.033	0.032	0.031	0.035	0.030	0.032	0.028	0.038	0.023	0.038	0.048	0.048	0.027	0.044	0.040	0.045	0.047	0.027	0.025	0.029	0.031	0.044	0.031	0.009
F482_483.3277_4.1_P	0.084	0.069	0.056	0.098	0.044	0.034	0.056	0.033	0.069	0.048	0.069	0.033	0.040	0.021	0.046	0.031	0.048	0.066	0.055	0.048	0.053	0.059	0.050	0.057	0.046	0.044	0.031	0.038	0.049	0.040	0.057	0.033	0.049	0.030	0.034	0.045	0.019	0.038	0.064	0.049	0.049	0.081	0.057	0.035	0.052	0.038	0.063	0.052	0.057	0.043	0.032	0.043	0.033
F483_182.0128_13.1_N	0.011	0.009	0.006	0.007	0.006	0.006	0.007	0.005	0.006	0.010	0.007	0.007	0.006	0.013	0.008	0.006	0.008	0.008	0.006	0.006	0.006	0.009	0.007	0.008	0.006	0.006	0.007	0.011	0.008	0.011	0.009	0.008	0.007	0.006	0.008	0.009	0.008	0.008	0.007	0.011	0.011	0.011	0.011	0.008	0.011	0.011	0.017	0.031	0.019	0.011	0.013	0.020	0.022
F484_236.0927_4.5_N	0.000	0.001	0.012	0.008	0.021	0.017	0.001	0.012	0.013	0.007	0.015	0.017	0.014	0.003	0.011	0.014	0.013	0.000	0.000	0.015	0.011	0.012	0.011	0.014	0.021	0.018	0.024	0.021	0.016	0.019	0.017	0.023	0.013	0.028	0.025	0.016	0.030	0.012	0.021	0.022	0.019	0.000	0.013	0.017	0.018	0.020	0.019	0.016	0.023	0.021	0.024	0.017	0.000
F485_271.1646_9.9_P	0.007	0.009	0.009	0.004	0.007	0.020	0.010	0.014	0.004	0.004	0.003	0.014	0.005	0.043	0.005	0.011	0.024	0.012	0.005	0.008	0.003	0.005	0.015	0.007	0.004	0.003	0.008	0.003	0.017	0.008	0.010	0.009	0.003	0.008	0.006	0.012	0.011	0.013	0.012	0.004	0.009	0.007	0.009	0.004	0.007	0.003	0.010	0.008	0.009	0.029	0.025	0.010	0.007
F487_346.1451_3.4_N	0.004	0.006	0.006	0.006	0.006	0.006	0.006	0.005	0.005	0.005	0.005	0.005	0.007	0.018	0.007	0.005	0.005	0.004	0.004	0.005	0.005	0.006	0.005	0.006	0.005	0.005	0.005	0.004	0.005	0.004	0.005	0.004	0.004	0.004	0.003	0.005	0.003	0.005	0.003	0.003	0.002	0.003	0.004	0.003	0.003	0.003	0.003	0.003	0.004	0.004	0.004	0.004	0.004
F488_858.5635_3.2_P	0.061	0.145	0.038	0.075	0.051	0.055	0.025	0.043	0.030	0.049	0.005	0.048	0.041	0.078	0.054	0.020	0.028	0.038	0.058	0.041	0.042	0.032	0.049	0.033	0.056	0.042	0.028	0.032	0.036	0.026	0.030	0.045	0.028	0.031	0.035	0.030	0.051	0.049	0.028	0.029	0.018	0.053	0.023	0.023	0.023	0.013	0.016	0.020	0.007	0.005	0.011	0.016	0.035
F490_281.0988_8.8_N	0.013	0.003	0.016	0.008	0.019	0.015	0.003	0.011	0.011	0.016	0.015	0.019	0.012	0.013	0.015	0.016	0.015	0.012	0.008	0.015	0.008	0.010	0.009	0.018	0.016	0.017	0.018	0.021	0.024	0.016	0.014	0.014	0.010	0.014	0.021	0.018	0.017	0.009	0.021	0.021	0.017	0.016	0.015	0.016	0.016	0.018	0.021	0.020	0.024	0.022	0.023	0.017	0.016
F492_233.0776_12.6_N	0.038	0.023	0.029	0.028	0.014	0.017	0.034	0.013	0.027	0.028	0.021	0.023	0.027	0.021	0.017	0.018	0.023	0.032	0.047	0.030	0.014	0.025	0.046	0.015	0.032	0.016	0.013	0.033	0.038	0.022	0.022	0.020	0.037	0.038	0.032	0.027	0.024	0.047	0.017	0.012	0.015	0.033	0.018	0.010	0.018	0.029	0.018	0.015	0.018	0.022	0.015	0.016	0.028
F493_385.1813_10.8_P	0.006	0.008	0.015	0.012	0.008	0.018	0.011	0.025	0.009	0.006	0.008	0.005	0.008	0.038	0.005	0.016	0.032	0.019	0.008	0.021	0.017	0.013	0.013	0.004	0.008	0.014	0.013	0.015	0.018	0.016	0.016	0.005	0.007	0.014	0.005	0.020	0.005	0.046	0.014	0.008	0.016	0.011	0.014	0.005	0.024	0.013	0.040	0.027	0.004	0.021	0.014	0.004	0.012
F495_651.5338_3.1_P	0.035	0.028	0.023	0.038	0.015	0.022	0.033	0.012	0.024	0.049	0.021	0.030	0.015	0.042	0.018	0.014	0.036	0.037	0.035	0.023	0.014	0.017	0.019	0.024	0.025	0.019	0.019	0.023	0.016	0.024	0.023	0.027	0.026	0.033	0.033	0.022	0.022	0.044	0.014	0.012	0.021	0.023	0.012	0.008	0.010	0.011	0.022	0.022	0.020	0.022	0.015	0.015	0.035
F498_219.1085_16.7_P	0.012	0.005	0.011	0.007	0.019	0.026	0.004	0.014	0.009	0.005	0.014	0.019	0.015	0.019	0.017	0.024	0.015	0.011	0.006	0.006	0.009	0.018	0.016	0.017	0.014	0.008	0.018	0.015	0.028	0.016	0.014	0.022	0.011	0.015	0.012	0.021	0.018	0.013	0.016	0.014	0.013	0.005	0.011	0.013	0.008	0.007	0.010	0.010	0.009	0.019	0.019	0.012	0.004
F500_216.9091_19.5_N	0.006	0.012	0.019	0.016	0.018	0.026	0.005	0.020	0.019	0.008	0.012	0.023	0.017	0.010	0.021	0.013	0.017	0.011	0.006	0.009	0.019	0.011	0.007	0.019	0.010	0.014	0.015	0.007	0.020	0.008	0.011	0.020	0.014	0.015	0.006	0.012	0.017	0.014	0.029	0.019	0.025	0.012	0.016	0.017	0.019	0.012	0.023	0.021	0.022	0.025	0.021	0.020	0.009
F501_459.1512_9.9_P	0.002	0.006	0.001	0.002	0.001	0.001	0.004	0.001	0.002	0.002	0.002	0.002	0.001	0.020	0.001	0.001	0.002	0.002	0.003	0.002	0.001	0.001	0.001	0.001	0.002	0.002	0.001	0.001	0.001	0.001	0.002	0.004	0.001	0.003	0.005	0.007	0.004	0.008	0.004	0.002	0.006	0.007	0.005	0.001	0.003	0.002	0.025	0.011	0.046	0.034	0.020	0.017	0.052
F502_275.075_9.5_P	0.020	0.011	0.011	0.026	0.011	0.012	0.011	0.015	0.006	0.011	0.006	0.009	0.009	0.020	0.008	0.008	0.006	0.011	0.005	0.006	0.007	0.006	0.007	0.014	0.007	0.015	0.005	0.026	0.007	0.012	0.010	0.012	0.006	0.011	0.012	0.018	0.011	0.014	0.006	0.007	0.010	0.018	0.009	0.006	0.009	0.006	0.050	0.036	0.030	0.026	0.013	0.025	0.032
F503_226.9962_12.6_N	0.020	0.013	0.017	0.034	0.005	0.012	0.011	0.012	0.017	0.024	0.025	0.021	0.016	0.012	0.016	0.022	0.024	0.032	0.019	0.034	0.028	0.012	0.018	0.010	0.010	0.013	0.013	0.016	0.008	0.016	0.026	0.025	0.023	0.019	0.029	0.029	0.028	0.041	0.021	0.018	0.025	0.043	0.023	0.015	0.026	0.014	0.043	0.013	0.061	0.028	0.027	0.028	0.038
F504_439.1411_16.2_P	0.026	0.004	0.008	0.011	0.011	0.009	0.004	0.012	0.014	0.007	0.013	0.031	0.024	0.017	0.021	0.032	0.014	0.024	0.007	0.007	0.012	0.033	0.033	0.011	0.020	0.004	0.015	0.031	0.015	0.034	0.030	0.033	0.033	0.024	0.039	0.017	0.033	0.016	0.003	0.003	0.004	0.002	0.004	0.008	0.006	0.002	0.009	0.006	0.012	0.024	0.011	0.015	0.009
F505_346.0445_14.2_N	0.026	0.010	0.026	0.022	0.026	0.027	0.004	0.037	0.045	0.021	0.020	0.063	0.029	0.017	0.019	0.037	0.039	0.029	0.031	0.058	0.044	0.023	0.028	0.020	0.027	0.035	0.024	0.027	0.014	0.026	0.024	0.029	0.027	0.027	0.028	0.033	0.050	0.084	0.024	0.024	0.014	0.029	0.030	0.013	0.024	0.019	0.029	0.010	0.034	0.029	0.021	0.021	0.032
F506_190.0857_10_P	0.031	0.016	0.036	0.032	0.063	0.057	0.021	0.063	0.047	0.028	0.052	0.048	0.062	0.034	0.062	0.058	0.053	0.039	0.029	0.050	0.056	0.061	0.049	0.047	0.045	0.049	0.051	0.042	0.060	0.058	0.050	0.068	0.054	0.053	0.063	0.044	0.059	0.051	0.067	0.081	0.091	0.052	0.079	0.066	0.078	0.084	0.043	0.041	0.052	0.052	0.077	0.048	0.024
F507_222.098_13.2_N	0.003	0.002	0.005	0.002	0.009	0.007	0.004	0.007	0.004	0.007	0.009	0.010	0.007	0.011	0.008	0.011	0.007	0.004	0.003	0.004	0.007	0.009	0.008	0.006	0.007	0.007	0.013	0.007	0.012	0.010	0.008	0.007	0.009	0.009	0.006	0.012	0.007	0.004	0.005	0.006	0.012	0.007	0.009	0.007	0.005	0.009	0.014	0.019	0.015	0.012	0.016	0.036	0.005
F508_219.0792_7.9_P	0.011	0.009	0.016	0.015	0.020	0.027	0.010	0.015	0.013	0.012	0.014	0.023	0.015	0.022	0.015	0.016	0.010	0.014	0.007	0.012	0.008	0.011	0.010	0.024	0.011	0.012	0.026	0.012	0.025	0.011	0.011	0.016	0.010	0.010	0.009	0.017	0.018	0.007	0.015	0.016	0.017	0.012	0.017	0.019	0.027	0.017	0.017	0.017	0.023	0.028	0.030	0.024	0.011
F509_529.2241_11.1_P	0.009	0.017	0.008	0.012	0.009	0.008	0.009	0.010	0.009	0.011	0.012	0.010	0.015	0.035	0.010	0.019	0.014	0.012	0.010	0.015	0.008	0.009	0.009	0.007	0.009	0.008	0.009	0.010	0.018	0.011	0.022	0.029	0.024	0.008	0.016	0.027	0.020	0.015	0.025	0.025	0.033	0.010	0.017	0.045	0.050	0.039	0.012	0.011	0.025	0.040	0.041	0.017	0.016
F512_623.5028_3.1_P	0.041	0.055	0.036	0.053	0.016	0.034	0.053	0.029	0.052	0.060	0.030	0.045	0.030	0.072	0.036	0.032	0.058	0.055	0.050	0.038	0.032	0.027	0.034	0.027	0.050	0.043	0.035	0.040	0.016	0.040	0.039	0.048	0.028	0.036	0.036	0.043	0.041	0.055	0.034	0.020	0.035	0.036	0.024	0.015	0.028	0.013	0.032	0.023	0.029	0.030	0.019	0.020	0.046
F513_270.1332_7.9_P	0.032	0.008	0.017	0.008	0.013	0.036	0.007	0.010	0.004	0.003	0.008	0.051	0.018	0.032	0.005	0.012	0.009	0.013	0.015	0.006	0.004	0.023	0.014	0.009	0.008	0.009	0.011	0.007	0.038	0.015	0.008	0.009	0.005	0.024	0.022	0.016	0.017	0.002	0.011	0.012	0.013	0.011	0.012	0.021	0.008	0.007	0.003	0.003	0.001	0.006	0.015	0.002	0.005
F514_242.0824_3.8_N	0.002	0.004	0.002	0.003	0.004	0.003	0.005	0.002	0.003	0.002	0.002	0.002	0.003	0.018	0.003	0.003	0.002	0.002	0.002	0.002	0.002	0.002	0.002	0.002	0.002	0.002	0.002	0.002	0.002	0.002	0.002	0.002	0.002	0.002	0.002	0.002	0.002	0.002	0.002	0.002	0.001	0.002	0.002	0.002	0.002	0.002	0.001	0.001	0.001	0.001	0.002	0.002	0.004
F515_156.0218_14.3_N	0.034	0.039	0.023	0.032	0.023	0.024	0.044	0.028	0.024	0.035	0.029	0.027	0.026	0.038	0.024	0.026	0.025	0.029	0.033	0.029	0.024	0.026	0.025	0.029	0.026	0.029	0.034	0.040	0.036	0.039	0.028	0.028	0.028	0.022	0.029	0.035	0.029	0.034	0.029	0.040	0.036	0.045	0.037	0.036	0.037	0.037	0.070	0.089	0.064	0.059	0.057	0.071	0.100

F517_628.2209_11.5_P	0.034	0.077	0.043	0.036	0.006	0.010	0.019	0.007	0.024	0.058	0.035	0.041	0.016	0.029	0.030	0.018	0.033	0.035	0.022	0.023	0.019	0.010	0.027	0.009	0.022	0.023	0.005	0.040	0.005	0.015	0.017	0.007	0.008	0.018	0.015	0.015	0.007	0.033	0.003	0.002	0.003	0.016	0.003	0.005	0.005	0.003	0.019	0.006	0.010	0.007	0.003	0.004	0.006
F518_315.1942_11.4_P	0.023	0.041	0.039	0.026	0.012	0.019	0.011	0.014	0.016	0.041	0.034	0.032	0.019	0.033	0.038	0.023	0.043	0.021	0.017	0.017	0.021	0.013	0.033	0.023	0.019	0.037	0.013	0.045	0.019	0.018	0.014	0.009	0.008	0.022	0.013	0.017	0.014	0.036	0.004	0.004	0.006	0.011	0.010	0.006	0.008	0.006	0.016	0.012	0.008	0.010	0.006	0.009	0.007
F519_191.0558_13.5_N	0.033	0.025	0.015	0.018	0.020	0.023	0.018	0.019	0.017	0.014	0.012	0.022	0.029	0.134	0.027	0.033	0.031	0.024	0.013	0.010	0.012	0.030	0.021	0.025	0.015	0.009	0.023	0.021	0.030	0.031	0.026	0.034	0.021	0.023	0.028	0.034	0.038	0.022	0.023	0.016	0.018	0.012	0.021	0.022	0.021	0.012	0.014	0.015	0.017	0.030	0.032	0.026	0.030
F521_785.6521_3.6_P	0.099	0.067	0.079	0.086	0.044	0.040	0.040	0.033	0.053	0.049	0.010	0.047	0.057	0.158	0.049	0.025	0.051	0.085	0.052	0.046	0.040	0.025	0.048	0.034	0.040	0.072	0.037	0.056	0.028	0.032	0.087	0.040	0.068	0.048	0.061	0.039	0.053	0.046	0.031	0.039	0.031	0.043	0.037	0.026	0.032	0.030	0.035	0.029	0.049	0.042	0.055	0.031	0.114
F522_128.0703_7.3_P	0.011	0.032	0.019	0.016	0.020	0.023	0.008	0.019	0.018	0.012	0.016	0.023	0.022	0.138	0.020	0.023	0.015	0.017	0.016	0.015	0.011	0.015	0.014	0.023	0.014	0.016	0.016	0.015	0.018	0.013	0.018	0.026	0.013	0.017	0.016	0.023	0.034	0.022	0.030	0.021	0.014	0.001	0.020	0.028	0.014	0.013	0.017	0.012	0.014	0.020	0.018	0.012	0.023
F523_242.0516_17.8_N	0.020	0.033	0.023	0.037	0.013	0.018	0.021	0.023	0.024	0.028	0.020	0.024	0.030	0.009	0.026	0.008	0.026	0.031	0.017	0.019	0.024	0.016	0.013	0.024	0.028	0.021	0.007	0.026	0.004	0.013	0.021	0.026	0.014	0.021	0.022	0.024	0.024	0.020	0.002	0.001	0.001	0.015	0.000	0.003	0.000	0.000	0.020	0.002	0.024	0.002	0.001	0.001	0.026
F524_353.3059_3.2_N	0.018	0.031	0.021	0.024	0.029	0.017	0.032	0.026	0.024	0.020	0.019	0.026	0.035	0.012	0.030	0.030	0.019	0.018	0.019	0.026	0.022	0.015	0.018	0.032	0.019	0.020	0.027	0.026	0.023	0.017	0.017	0.018	0.027	0.015	0.011	0.015	0.020	0.018	0.006	0.006	0.004	0.006	0.011	0.011	0.005	0.008	0.026	0.011	0.020	0.022	0.014	0.018	0.021
F525_283.1397_8.5_P	0.015	0.005	0.013	0.006	0.008	0.014	0.004	0.007	0.004	0.007	0.010	0.045	0.007	0.021	0.011	0.019	0.011	0.011	0.011	0.005	0.003	0.010	0.015	0.018	0.009	0.012	0.009	0.011	0.023	0.011	0.014	0.007	0.005	0.012	0.020	0.030	0.009	0.005	0.005	0.008	0.010	0.011	0.008	0.013	0.008	0.009	0.010	0.012	0.010	0.022	0.023	0.008	0.007
F526_131.0812_7.8_P	0.031	0.032	0.049	0.035	0.068	0.075	0.034	0.049	0.063	0.029	0.078	0.056	0.096	0.033	0.059	0.067	0.051	0.044	0.049	0.039	0.043	0.037	0.042	0.040	0.044	0.028	0.069	0.030	0.069	0.044	0.057	0.057	0.040	0.057	0.062	0.059	0.078	0.045	0.068	0.039	0.057	0.030	0.062	0.040	0.048	0.048	0.065	0.050	0.070	0.070	0.088	0.064	0.047
F527_247.1703_3.5_N	0.040	0.048	0.067	0.055	0.107	0.095	0.030	0.073	0.065	0.029	0.080	0.066	0.071	0.018	0.068	0.056	0.033	0.029	0.014	0.040	0.039	0.065	0.056	0.066	0.051	0.049	0.047	0.039	0.048	0.060	0.031	0.030	0.027	0.029	0.018	0.039	0.020	0.029	0.060	0.066	0.049	0.033	0.084	0.072	0.073	0.089	0.020	0.027	0.019	0.028	0.027	0.038	0.015
F528_328.1614_12.9_P	0.258	0.249	0.094	0.172	0.028	0.045	0.416	0.058	0.169	0.176	0.122	0.066	0.056	0.399	0.048	0.066	0.154	0.255	0.478	0.146	0.127	0.074	0.121	0.073	0.105	0.066	0.050	0.102	0.063	0.093	0.128	0.072	0.065	0.113	0.119	0.103	0.060	0.144	0.065	0.070	0.076	0.204	0.075	0.068	0.101	0.089	0.081	0.064	0.103	0.079	0.064	0.075	0.205
F529_557.3281_4_N	0.397	0.392	0.462	0.514	0.450	0.314	0.430	0.334	0.491	0.293	0.287	0.394	0.340	0.227	0.198	0.197	0.309	0.369	0.326	0.455	0.331	0.322	0.335	0.404	0.331	0.439	0.351	0.423	0.333	0.217	0.280	0.258	0.285	0.203	0.251	0.268	0.221	0.193	0.341	0.328	0.254	0.355	0.314	0.282	0.275	0.183	0.290	0.285	0.284	0.246	0.211	0.239	0.226
F531_101.0708_15.4_P	0.438	0.252	0.427	0.415	0.391	0.384	0.243	0.475	0.558	0.450	0.513	0.403	0.568	0.662	0.467	0.440	0.489	0.490	0.433	0.535	0.557	0.520	0.517	0.386	0.571	0.396	0.477	0.505	0.490	0.439	0.568	0.575	0.641	0.598	0.536	0.497	0.529	0.519	0.333	0.351	0.375	0.319	0.362	0.250	0.328	0.343	0.344	0.324	0.414	0.338	0.457	0.375	0.259
F532_241.2174_3.4_N	0.234	0.345	0.448	0.302	0.507	0.479	0.189	0.411	0.490	0.255	0.454	0.388	0.537	0.318	0.456	0.364	0.349	0.244	0.179	0.372	0.400	0.437	0.419	0.542	0.444	0.379	0.386	0.305	0.430	0.333	0.344	0.286	0.330	0.301	0.193	0.349	0.221	0.321	0.254	0.203	0.154	0.106	0.245	0.232	0.188	0.231	0.221	0.208	0.229	0.294	0.241	0.286	0.128
F533_325.1532_9.6_P	0.322	0.172	0.198	0.175	0.102	0.414	0.229	0.113	0.056	0.054	0.026	0.367	0.029	0.060	0.062	0.266	0.495	0.198	0.261	0.117	0.048	0.120	0.260	0.094	0.035	0.127	0.090	0.051	0.281	0.194	0.048	0.043	0.024	0.065	0.057	0.230	0.091	0.050	0.495	0.161	0.529	1.074	0.317	0.429	0.700	0.221	0.025	0.047	0.025	0.126	0.248	0.020	0.025
F534_733.5565_3.5_P	1.365	2.185	0.717	1.137	0.464	0.549	3.641	0.496	0.981	1.468	0.553	1.049	0.693	2.334	0.763	0.538	0.583	1.706	1.285	0.715	0.759	0.338	0.564	0.681	0.535	0.880	0.689	0.695	0.274	1.008	0.903	0.773	0.359	0.397	0.192	0.931	0.418	1.479	0.518	0.505	0.305	1.427	0.519	0.589	0.826	0.409	1.193	0.635	1.489	1.009	0.575	0.995	2.164
F535_516.3079_4_P	0.275	0.368	0.402	0.358	0.362	0.262	0.184	0.272	0.103	0.249	0.132	0.279	0.245	0.101	0.262	0.060	0.238	0.265	0.202	0.298	0.202	0.228	0.232	0.239	0.248	0.302	0.156	0.204	0.227	0.168	0.098	0.135	0.094	0.163	0.143	0.130	0.130	0.117	0.332	0.215	0.263	0.388	0.341	0.206	0.315	0.262	0.148	0.212	0.197	0.123	0.050	0.143	0.124
F536_466.2925_4_P	0.568	1.064	0.693	0.863	0.575	0.540	0.688	0.714	0.573	0.470	0.248	0.733	0.511	0.361	0.465	0.232	0.338	0.496	0.439	0.382	0.392	0.410	0.372	0.526	0.452	0.756	0.402	0.619	0.280	0.240	0.310	0.335	0.271	0.235	0.273	0.445	0.346	0.290	0.456	0.414	0.223	0.478	0.402	0.523	0.238	0.271	0.252	0.281	0.273	0.295	0.110	0.239	0.307
F537_203.1386_14.8_P	0.087	0.014	0.084	0.043	0.089	0.185	0.010	0.140	0.079	0.030	0.061	0.073	0.087	0.028	0.088	0.092	0.119	0.080	0.070	0.100	0.058	0.093	0.117	0.087	0.116	0.062	0.157	0.097	0.209	0.157	0.136	0.149	0.102	0.086	0.118	0.239	0.119	0.128	0.342	0.272	0.358	0.248	0.438	0.436	0.268	0.359	0.071	0.049	0.069	0.181	0.194	0.057	0.009
F538_112.0503_12.1_P	0.182	0.138	0.152	0.157	0.272	0.274	0.061	0.200	0.202	0.266	0.261	0.291	0.216	0.174	0.302	0.244	0.262	0.182	0.151	0.196	0.129	0.164	0.162	0.192	0.251	0.191	0.299	0.127	0.322	0.371	0.224	0.247	0.219	0.258	0.275	0.378	0.317	0.267	0.326	0.312	0.272	0.227	0.303	0.262	0.436	0.353	0.520	0.364	0.579	0.493	0.544	0.473	0.473
F539_775.6033_3.5_P	1.590	0.673	2.126	2.401	1.377	1.277	6.749	1.593	2.115	2.402	0.670	2.090	2.401	0.211	1.526	1.276	1.217	2.362	2.603	1.330	1.033	1.206	1.474	1.227	1.647	1.238	1.243	2.193	0.641	1.529	1.402	1.760	1.287	1.154	1.956	1.244	1.592	1.778	1.223	1.228	1.001	1.521	0.828	0.804	1.505	0.998	1.331	1.144	1.875	1.046	0.499	1.580	4.698
F540_454.2929_4_P	0.929	1.370	1.069	1.241	0.793	0.889	0.733	0.873	0.945	0.777	0.516	0.987	0.768	0.584	0.593	0.363	0.499	0.816	0.877	0.805	0.624	0.839	0.773	0.807	0.726	0.848	0.500	0.656	0.474	0.534	0.498	0.466	0.533	0.504	0.558	0.555	0.451	0.447	0.854	0.578	0.456	0.843	0.568	0.587	0.688	0.503	0.514	0.558	0.541	0.426	0.330	0.413	0.642
F541_166.0178_9.5_N	0.159	0.080	0.128	0.102	0.147	0.212	0.078	0.164	0.127	0.091	0.144	0.184	0.151	0.061	0.179	0.171	0.174	0.133	0.131	0.134	0.117	0.181	0.154	0.133	0.114	0.124	0.162	0.121	0.250	0.318	0.162	0.148	0.150	0.153	0.172	0.186	0.172	0.142	0.204	0.215	0.354	0.211	0.230	0.206	0.390	0.350	0.153	0.239	0.189	0.251	0.297	0.377	0.097
F542_104.0527_11.6_P	0.090	0.073	0.131	0.104	0.147	0.165	0.054	0.177	0.130	0.078	0.132	0.133	0.166	0.064	0.160	0.174	0.158	0.124	0.071	0.126	0.199	0.162	0.121	0.150	0.128	0.169	0.165	0.123	0.145	0.132	0.151	0.178	0.148	0.111	0.122	0.143	0.188	0.127	0.284	0.357	0.361	0.204	0.357	0.427	0.314	0.401	0.109	0.104	0.125	0.140	0.173	0.142	0.066
F543_343.164_14.9_P	0.412	0.286	0.599	0.501	0.544	0.431	0.160	0.300	0.339	0.723	0.424	0.515	0.357	0.102	0.302	0.378	0.397	0.647	0.429	0.441	0.324	0.258	0.295	0.486	0.309	0.644	0.314	0.352	0.286	0.327	0.332	0.491	0.339	0.694	0.526	0.428	0.479	0.358	0.344	0.343	0.491	0.364	0.250	0.198	0.351	0.239	0.577	0.636	0.415	0.470	0.433	0.362	0.318
F544_381.3371_3.1_N	0.231	0.345	0.276	0.402	0.355	0.280	0.333	0.421	0.339	0.317	0.311	0.334	0.534	0.179	0.469	0.397	0.302	0.254	0.237	0.294	0.371	0.211	0.230	0.491	0.334	0.340	0.348	0.428	0.280	0.260	0.299	0.338	0.350	0.189	0.172	0.308	0.352	0.280	0.051	0.048	0.046	0.045	0.068	0.053	0.045	0.047	0.322	0.281	0.304	0.300	0.254	0.277	0.258
F545_286.1392_13.9_P	0.215	0.121	0.235	0.195	0.368	0.484	0.099	0.338	0.205	0.138	0.323	0.310	0.361	0.305	0.481	0.423	0.239	0.205	0.127	0.164	0.351	0.281	0.265	0.379	0.334	0.326	0.387	0.303	0.563	0.544	0.305	0.467	0.453	0.261	0.284	0.479	0.425	0.375	0.358	0.505	0.401	0.163	0.472	0.425	0.229	0.359	0.316	0.285	0.306	0.318	0.243	0.361	0.133
F546_175.1438_21.9_P	0.106	0.058	0.098	0.082	0.097	0.120	0.050	0.039	0.106	0.113	0.105	0.083	0.137	0.039	0.131	0.101	0.155	0.158	0.091	0.102	0.133	0.124	0.127	0.143	0.084	0.096	0.112	0.109	0.110	0.081	0.199	0.213	0.094	0.199	0.218	0.190	0.132	0.055	0.071	0.075	0.157	0.119	0.092	0.064	0.151	0.078	0.308	0.453	0.216	0.165	0.287	0.238	0.181
F547_186.1123_10.2_P	0.117	0.036	0.071	0.071	0.088	0.079	0.057	0.091	0.116	0.092	0.572	0.131	0.204	0.178	0.208	0.301	0.129	0.129	0.107	0.123	0.184	0.214	0.222	0.084	0.118	0.064	0.103	0.083	0.208	0.120	0.184	0.130	0.194	0.115	0.144	0.118	0.094	0.087	0.064	0.041	0.127	0.075	0.103	0.075	0.135	0.135	0.086	0.053	0.137	0.161	0.105	0.137	0.044
F548_133.1049_11.3_P	0.594	0.515	0.438	0.561	0.343	0.365	0.480	0.394	0.531	0.437	0.510	0.362	0.517	0.521	0.567	0.573	0.746	0.682	0.693	0.620	0.774	0.653	0.558	0.287	0.389	0.437	0.368	0.331	0.446	0.423	0.945	0.643	0.951	0.681	0.971	0.660	0.767	0.804	0.813	0.802	0.925	0.733	0.725	1.072	1.025	0.963	0.570	0.419	0.711	0.754	1.094	0.543	0.646
F550_786.9405_12.5_N	0.014	0.014	0.021	0.006	0.016	0.019	0.015	0.054	0.018	0.051	0.014	0.004	0.027	0.059	0.040	0.006	0.044	0.091	0.008	0.004	0.004	0.028	0.011	0.083	0.003	0.057	0.017	0.043	0.028	0.051	0.017	0.038	0.006	0.019	0.021	0.072	0.028	0.007	0.051	0.085	0.079	0.110	0.118	0.052	0.130	0.057	0.222	0.485	0.198	0.133	0.078	0.165	0.072
F551_818.5548_3.4_N	0.117	0.878	0.276	0.912	0.682	0.339	1.889	0.150	0.291	0.543	0.228	0.695	0.522	2.349	0.522	0.269	0.408	0.517	0.302	0.248	0.278	0.257	0.332	0.577	0.293	0.714	0.357	0.755	0.130	0.304	0.361	0.390	0.348	0.191	0.266	0.320	0.700	0.577	0.384	0.261	0.205	0.487	0.173	0.321	0.261	0.169	0.234	0.490	0.366	0.425	0.186	0.275	0.459
F552_733.556_3.5_P	1.047	1.775	0.567	1.205	0.322	0.409	1.642	0.403	0.923	0.957	0.669	0.815	0.503	2.200	0.604	0.407	0.391	0.883	0.974	0.556	0.634	0.231	0.683	0.440	0.387	0.405	0.519	0.421	0.194	0.821	0.668	0.702	0.237	0.290	0.678	0.760	0.389	1.169	0.364	0.376	0.223	0.947	0.609	0.385	0.521	0.325	1.122	0.444	1.508	0.838	0.505	0.594	1.585
F553_619.2881_3.6_N	0.161	0.177	0.133	0.139	0.158	0.163	0.361	0.161	0.143	0.123	0.093	0.124	0.127	0.189	0.129	0.157	0.128	0.148	0.133	0.176	0.137	0.130	0.159	0.143	0.061	0.216	0.182	0.106	0.137	0.145	0.099	0.069	0.144	0.086	0.077	0.154	0.123	0.114	0.231	0.345	0.254	0.278	0.221	0.628	0.239	0.290	0.064	0.069	0.069	0.084	0.090	0.071	0.075
F554_310.283_3.2_N	0.410	0.638	0.845	0.564	0.904	1.051	0.371	0.707	0.750	0.528	0.863	0.712	1.225	0.298	0.976	0.888	0.587	0.353	0.453	0.739	0.651	0.985	0.829	0.966	0.677	0.775	0.648	0.501	0.839	0.562	0.586	0.460	0.679	0.432	0.282	0.611	0.292	0.459	0.264	0.244	0.124	0.094	0.263	0.310	0.189	0.283	0.307	0.186	0.249	0.548	0.460	0.229	0.208
F555_124.9913_11.9_N	0.310	0.248	0.293	0.261	0.250	0.455	0.185	0.463	0.281	0.282	0.316	0.338	0.390	0.201	0.418	0.512	0.566	0.404	0.366	0.352	0.359	0.319	0.435	0.280	0.290	0.213	0.284	0.235	0.533	0.541	0.396	0.433	0.354	0.369	0.502	0.440	0.500	0.764	0.403	0.302	0.434	0.359	0.361	0.321	0.558	0.482	0.451	0.340	0.477	0.493	0.625	0.485	0.399
F556_157.0969_12.9_P	0.902	0.283	0.648	0.351	0.203	0.228	0.664	0.459	0.540	0.386	0.722	0.226	0.226	0.410	0.215	0.275	0.800	0.773	1.150	0.517	0.793	0.802	0.766	0.296	0.419	0.410	0.361	0.388	0.453	0.415	0.695	0.263	0.552	0.426	0.458	0.609	0.242	0.462	0.413	0.544	0.475	0.559	0.594	0.574	0.538	0.732	0.362	0.301	0.357	0.326	0.408	0.219	0.295
F557_426.3569_3.9_P	0.610	0.480	0.571	0.621	0.376	0.327	0.587	0.277	0.757	0.312	0.400	0.579	0.387	0.427	0.495	0.400	0.304	0.628	0.395	0.862	0.310	0.223	0.495	0.348	0.434	0.542	0.382	0.487	0.328	0.756	0.582	0.297	0.491	0.396	0.523	0.505	0.301	0.825	0.268	0.126	0.193	0.563	0.418	0.370	0.304	0.399	0.594	0.244	1.019	0.530	0.478	0.905	0.528
F558_173.0589_14.3_P	2.268	1.468	2.196	1.751	3.618	3.633	1.394	4.273	2.518	2.826	3.570	2.950	3.699	1.085	3.299	3.783	3.158	1.939	1.767	2.711	3.206	3.095	2.937	4.373	3.020	4.109	5.448	5.056	5.518	5.292	3.170	3.419	3.740	2.545	2.626	5.041	3.917	3.990	4.695	6.428	5.985	3.950	5.995	5.931	5.527	6.697	9.797	13.54	8.794	9.063	9.215	11.37	5.700
F559_478.2935_3.8_N	1.287	1.980	1.556	1.650	0.164	1.198	0.059	1.205	1.087	0.982	0.720	1.712	1.273	1.035	1.234	0.558	0.856	1.120	1.175	1.190	0.914	1.221	1.090	1.141	1.154	1.480	0.940	0.985	0.723	0.635	0.863	0.722	0.767	0.666	0.845	0.884	0.750	0.695	1.096	0.851	0.642	1.166	0.864	1.158	0.756	0.872	0.837	0.804	0.928	0.820	0.508	0.681	0.870
F560_133.0685_14.8_P	1.340	1.041	1.117	1.202	1.046	1.424	0.845	1.685	1.643	1.217	1.367	0.902	1.637	1.017	1.314	1.217	1.739	1.643	1.476	1.923	1.818	1.281	1.332	1.030	1.724	0.765	1.299	1.334	1.521	1.682	1.864	1.768	2.365	1.511	1.611	1.710	1.672	2.595	0.864	0.708	0.845	0.939	1.108	0.729	0.751	0.867	1.502	0.983	1.578	1.413	1.219	1.357	1.339
F561_260.1714_24.3_P	0.216	0.079	0.186	0.187	0.245	0.446	0.083	0.256	0.099	0.053	0.079	0.264	0.226	0.029	0.189	0.270	0.308	0.186	0.096	0.112	0.146	0.238	0.264	0.228	0.128	0.204	0.279	0.103	0.365	0.261	0.147	0.247	0.102	0.201	0.207	0.314	0.388	0.133	0.464	0.615	0.605	0.347	0.545	0.511	0.249	0.462	0.102	0.162	0.076	0.305	0.472	0.101	0.048
F562_160.0965_11_P	0.396	0.383	0.534	0.452	0.460	0.593	0.358	0.610	0.514	0.496	0.472	0.524	0.543	0.112	0.512	0.533	0.681	0.639	0.397	0.539	0.523	0.488	0.629	0.480	0.489	0.442	0.535	0.496	0.587	0.501	0.554	0.554	0.381	0.610	0.452	0.694	0.677	0.755	0.657	0.543	0.764	0.653	0.655	0.580	0.702	0.550	0.599	0.553	0.528	0.679	0.529	0.395	0.437
F563_159.1125_12.6_P	0.499	0.377	0.447	0.503	0.381	0.473	0.357	0.497	0.511	0.422	0.513	0.494	0.562	0.304	0.634	0.623	0.614	0.565	0.473	0.562	0.640	0.576	0.533	0.411	0.480	0.464	0.458	0.485	0.540	0.588	0.777	0.727	0.753	0.613	0.821	0.652	0.729	0.792	0.611	0.580	0.665	0.565	0.581	0.712	0.716	0.691	0.653	0.504	0.733	0.726	0.764	0.660	0.559
F564_246.1695_7.5_P	0.094	0.023	0.098	0.074	0.091	0.126	0.081	0.070	0.052	0.043	0.063	0.100	0.080	0.021	0.122	0.109	0.064	0.068	0.065	0.074	0.052	0.072	0.051	0.098	0.043	0.096	0.094	0.070	0.143	0.079	0.093	0.098	0.085	0.077	0.071	0.103	0.125	0.054	0.353	0.323	0.362	0.281	0.340	0.607	0.318	0.359	0.107	0.085	0.104	0.150	0.174	0.102	0.085
F565_580.325_4_N	0.481	0.546	0.566	0.509	0.573	0.300	0.539	0.422	0.600	0.344	0.304	0.572	0.430	0.303	0.459	0.287	0.357	0.392	0.315	0.274	0.365	0.400	0.352	0.445	0.380	0.539	0.477	0.547	0.411	0.308	0.341	0.279	0.307	0.221	0.280	0.347	0.250	0.209	0.544	0.367	0.393	0.491	0.405	0.378	0.370	0.359	0.301	0.347	0.370	0.307	0.252	0.251	0.270
F566_161.0453_15.5_N	0.442	0.228	0.367	0.286	0.407	0.333	0.379	0.533	0.505	0.371	0.437	0.290	0.537	1.161	0.428	0.654	0.515	0.482	0.477	0.450	0.597	0.919	0.630	0.412	0.805	0.370	0.374	0.424	0.569	0.517	0.666	0.590	0.803	1.153	0.654	0.375	0.546	0.421	0.220	0.186	0.220	0.278	0.334	0.222	0.251	0.284	0.349	0.234	0.379	0.257	0.251	0.391	0.229
F567_793.592_3.4_P	1.607	2.236	1.351	1.813	0.061	0.599	0.951	0.586	1.462	1.097	0.954	0.451	0.630	2.808	0.919	0.581	1.237	1.004	1.474	0.827	1.004	0.714	0.719	0.394	1.128	1.061	0.983	0.805	0.305	1.097	0.977	1.049	0.902	0.774	0.912	0.989	1.233	1.463	0.320	0.887	0.824	1.167	0.409	0.185	0.636	0.341	0.777	1.121	1.571	1.191	0.688	0.655	2.730
F568_788.5233_3.3_N	0.455	0.867	0.382	0.575	0.351	0.417	0.667	0.272	0.579	0.685	0.473	0.284	0.392	1.478	0.443	0.337	0.528	0.443	0.735	0.438	0.424	0.451	0.365	0.511	0.613	0.512	0.762	0.373	0.457	0.405	0.774	0.500	0.420	0.435	0.509	0.428	0.560	0.423	0.508	0.359	0.381	0.392	0.227	0.207	0.267	0.345	0.416	0.486	0.461	0.366	0.221	0.278	0.741
F569_149.0453_12.6_N	0.156	0.105	0.096	0.106	0.061	0.129	0.095	0.103	0.105	0.110	0.119	0.138	0.093	0.161	0.108	0.179	0.209	0.180	0.302	0.117	0.123	0.147	0.175	0.095	0.117	0.072	0.118	0.103	0.188	0.139	0.186	0.204	0.148	0.212	0.314	0.287	0.204	0.272	0.111	0.098	0.140	0.128	0.128	0.116	0.186	0.159	0.364	0.268	0.336	0.558	0.769	0.362	0.429

F570_215.1386_9.5_P	0.167	0.092	0.209	0.107	0.150	0.289	0.090	0.192	0.172	0.126	0.230	0.208	0.165	0.053	0.185	0.178	0.238	0.209	0.169	0.210	0.169	0.233	0.347	0.171	0.130	0.144	0.192	0.159	0.369	0.186	0.307	0.190	0.149	0.131	0.282	0.332	0.168	0.203	0.242	0.188	0.341	0.277	0.305	0.241	0.416	0.358	0.255	0.216	0.362	0.419	0.587	0.318	0.121
F571_643.2884_3.6_N	0.164	0.140	0.150	0.098	0.163	0.109	0.323	0.104	0.113	0.106	0.079	0.127	0.112	0.212	0.091	0.147	0.108	0.138	0.115	0.127	0.117	0.095	0.133	0.119	0.066	0.144	0.129	0.097	0.090	0.089	0.077	0.063	0.104	0.064	0.082	0.099	0.083	0.075	0.211	0.342	0.195	0.237	0.185	0.432	0.132	0.178	0.034	0.042	0.043	0.076	0.062	0.042	0.031
F572_204.134_11.7_P	0.323	0.050	0.191	0.081	0.227	0.410	0.129	0.175	0.120	0.139	0.353	0.779	0.232	0.052	0.235	0.420	0.205	0.151	0.153	0.081	0.087	0.180	0.378	0.112	0.234	0.118	0.202	0.136	0.438	0.213	0.253	0.174	0.262	0.482	0.364	0.402	0.316	0.184	0.113	0.127	0.080	0.035	0.054	0.072	0.063	0.070	0.318	0.176	0.316	0.804	0.656	0.358	0.180
F573_811.6032_3.5_P	2.620	5.259	1.381	3.121	1.222	1.348	3.912	1.513	3.380	2.490	1.378	1.078	1.368	0.572	1.501	1.305	2.335	2.127	3.474	2.094	1.730	1.633	1.478	1.272	1.657	2.151	2.135	1.904	1.073	2.607	2.323	2.195	1.930	1.966	2.069	1.734	1.740	3.103	0.570	1.100	1.267	2.275	1.001	0.688	1.485	0.878	2.235	2.364	2.044	1.577	1.707	1.476	4.562
F574_258.9819_15.5_N	6.260	6.497	5.861	5.891	6.037	2.362	5.179	2.470	7.561	6.431	5.237	6.760	2.261	3.973	5.086	10.01	9.302	7.825	7.337	7.489	4.145	5.188	7.410	5.172	8.166	6.198	2.096	6.358	1.997	8.091	4.874	9.722	6.957	9.686	8.090	2.854	9.279	9.151	2.010	0.117	0.151	6.359	1.917	3.278	3.493	0.145	6.224	3.112	7.312	1.751	0.182	1.569	7.983
F575_207.1123_10_P	0.230	0.148	0.314	0.235	0.418	0.397	0.123	0.423	0.349	0.220	0.383	0.345	0.424	0.146	0.448	0.405	0.384	0.272	0.204	0.348	0.372	0.427	0.344	0.312	0.328	0.350	0.367	0.305	0.397	0.422	0.381	0.444	0.442	0.347	0.426	0.350	0.433	0.362	0.451	0.502	0.596	0.362	0.502	0.438	0.571	0.559	0.327	0.289	0.376	0.349	0.517	0.352	0.188
F576_339.2524_3.6_P	0.243	0.654	0.168	0.331	0.156	0.205	0.229	0.342	0.248	0.191	0.058	0.088	0.130	0.208	0.219	0.086	0.112	0.209	0.064	0.132	0.111	0.181	0.070	0.241	0.075	0.180	0.098	0.193	0.080	0.162	0.089	0.061	0.089	0.047	0.078	0.108	0.119	0.105	0.551	0.669	0.387	0.659	0.602	0.854	0.439	0.604	0.271	0.486	0.266	0.222	0.098	0.250	0.430
F577_174.1484_7.6_P	0.160	0.012	0.078	0.085	0.186	0.186	0.071	0.144	0.113	0.187	0.172	0.154	0.142	0.069	0.106	0.143	0.099	0.126	0.082	0.139	0.108	0.203	0.159	0.183	0.152	0.178	0.351	0.286	0.420	0.201	0.188	0.126	0.104	0.153	0.135	0.336	0.138	0.164	0.235	0.373	0.351	0.266	0.338	0.395	0.353	0.371	0.546	0.577	0.327	0.523	0.535	0.419	0.245
F578_261.144_11.1_P	0.131	0.069	0.184	0.086	0.233	0.189	0.044	0.173	0.182	0.131	0.222	0.250	0.207	0.065	0.259	0.305	0.279	0.174	0.135	0.208	0.197	0.227	0.185	0.191	0.180	0.181	0.266	0.159	0.303	0.225	0.406	0.272	0.322	0.183	0.284	0.393	0.363	0.306	0.389	0.347	0.426	0.185	0.319	0.508	0.565	0.372	0.290	0.247	0.441	0.541	0.493	0.274	0.139
F579_189.123_11.8_P	0.222	0.098	0.172	0.165	0.175	0.271	0.149	0.191	0.157	0.237	0.258	0.198	0.210	0.044	0.216	0.306	0.232	0.212	0.155	0.226	0.198	0.295	0.285	0.183	0.187	0.181	0.291	0.275	0.426	0.342	0.255	0.218	0.189	0.296	0.282	0.401	0.253	0.317	0.281	0.339	0.529	0.466	0.456	0.369	0.571	0.430	0.624	0.637	0.437	0.562	0.644	0.474	0.339
F580_166.0859_4.3_P	0.713	0.286	0.975	0.802	0.624	0.642	0.620	0.946	2.583	0.661	0.948	1.496	1.190	0.234	1.185	1.024	0.901	1.046	0.813	1.354	0.916	0.637	0.829	0.641	0.965	0.881	0.685	0.690	0.429	0.608	1.114	0.820	1.140	0.756	1.338	0.981	1.164	1.046	0.565	0.398	0.459	0.754	0.487	0.559	0.658	0.405	0.995	0.712	0.962	0.692	0.461	0.223	0.636
F581_868.5642_3.3_N	0.443	1.577	0.605	0.800	0.238	0.638	1.305	0.267	0.540	0.507	0.463	0.522	0.291	3.239	0.589	0.438	0.635	0.758	0.822	0.628	0.450	0.617	0.454	0.679	0.361	0.647	0.586	0.584	0.402	0.525	0.763	0.655	0.527	0.498	0.531	0.467	0.767	0.620	0.447	0.194	0.469	0.627	0.375	0.317	0.435	0.331	0.567	0.539	0.557	0.481	0.350	0.380	0.645
F582_482.3235_4.1_P	0.564	0.503	0.436	0.497	0.266	0.280	0.479	0.253	0.352	0.290	0.292	0.392	0.244	0.172	0.270	0.168	0.227	0.433	0.434	0.514	0.250	0.235	0.240	0.272	0.331	0.340	0.224	0.392	0.241	0.248	0.347	0.238	0.312	0.217	0.299	0.283	0.219	0.199	0.386	0.298	0.306	0.414	0.275	0.199	0.240	0.218	0.290	0.314	0.368	0.312	0.284	0.301	0.264
F583_140.0374_13.7_P	0.098	0.060	0.130	0.068	0.167	0.202	0.053	0.236	0.092	0.082	0.124	0.150	0.197	0.103	0.140	0.244	0.235	0.131	0.123	0.160	0.151	0.177	0.213	0.164	0.111	0.137	0.164	0.131	0.337	0.225	0.161	0.183	0.158	0.225	0.359	0.214	0.173	0.252	0.213	0.180	0.322	0.285	0.293	0.151	0.222	0.274	0.180	0.160	0.166	0.191	0.324	0.192	0.086
F585_195.1224_4.5_P	0.016	0.026	0.148	0.019	0.032	0.128	0.033	0.241	0.406	0.121	0.089	0.232	0.074	0.169	0.099	0.040	0.152	0.008	0.016	0.216	0.238	0.199	0.429	0.158	0.381	0.194	0.119	0.209	0.301	0.185	0.023	0.947	0.321	0.349	0.414	0.175	1.794	0.321	0.150	0.276	0.046	0.018	0.045	0.300	0.080	0.109	0.395	1.151	0.552	0.255	0.342	0.053	0.023
F586_292.0556_10.2_P	0.019	0.007	0.029	0.015	0.031	0.045	0.007	0.048	0.030	0.044	0.044	0.038	0.040	0.032	0.024	0.052	0.036	0.024	0.017	0.050	0.044	0.062	0.049	0.065	0.048	0.041	0.084	0.109	0.102	0.096	0.068	0.049	0.049	0.045	0.044	0.074	0.048	0.058	0.081	0.078	0.144	0.098	0.124	0.123	0.155	0.130	0.324	0.359	0.224	0.263	0.231	0.243	0.119
F588_219.1337_12.8_P	0.083	0.098	0.146	0.119	0.203	0.305	0.072	0.228	0.098	0.060	0.073	0.163	0.188	0.158	0.175	0.169	0.130	0.099	0.082	0.119	0.077	0.106	0.105	0.212	0.097	0.155	0.233	0.094	0.191	0.168	0.102	0.185	0.074	0.106	0.093	0.186	0.302	0.105	0.311	0.271	0.289	0.203	0.328	0.304	0.201	0.208	0.100	0.114	0.094	0.135	0.139	0.090	0.065
F589_199.1703_3.5_N	0.068	0.080	0.095	0.081	0.103	0.119	0.111	0.106	0.546	0.060	0.091	0.088	0.113	1.159	0.118	0.091	0.121	0.051	0.055	0.089	0.114	0.107	0.087	0.112	0.096	0.090	0.103	0.076	0.109	0.097	0.106	0.086	0.121	0.102	0.057	0.096	0.125	0.151	0.075	0.061	0.069	0.029	0.088	0.072	0.058	0.090	0.074	0.067	0.088	0.069	0.076	0.087	0.056
F590_169.0581_15.3_P	0.674	0.537	0.268	0.744	0.108	0.129	0.456	0.149	0.392	0.540	0.276	0.303	0.236	0.310	0.159	0.134	0.229	0.846	1.305	0.352	0.230	0.174	0.220	0.126	0.286	0.155	0.107	0.267	0.086	0.239	0.367	0.181	0.257	0.525	0.365	0.131	0.194	0.278	0.106	0.081	0.114	0.384	0.082	0.057	0.130	0.077	0.144	0.072	0.232	0.100	0.108	0.094	0.578
F591_361.1453_11.6_P	0.285	0.242	0.260	0.317	0.134	0.192	0.194	0.193	0.258	0.238	0.230	0.248	0.232	0.115	0.246	0.228	0.286	0.331	0.233	0.303	0.258	0.227	0.219	0.187	0.231	0.211	0.130	0.222	0.129	0.235	0.290	0.265	0.237	0.274	0.303	0.220	0.307	0.340	0.281	0.240	0.291	0.477	0.300	0.304	0.367	0.244	0.229	0.187	0.281	0.249	0.182	0.210	0.242
F592_189.0403_11.6_N	0.101	0.129	0.175	0.189	0.211	0.284	0.145	0.257	0.174	0.127	0.158	0.250	0.258	0.081	0.296	0.287	0.288	0.203	0.155	0.219	0.205	0.180	0.212	0.241	0.145	0.172	0.215	0.164	0.189	0.219	0.240	0.260	0.144	0.171	0.183	0.261	0.379	0.322	0.306	0.234	0.310	0.174	0.283	0.317	0.289	0.304	0.245	0.214	0.309	0.351	0.300	0.254	0.213
F593_364.1354_10.4_P	0.058	0.012	0.066	0.062	0.140	0.142	0.011	0.119	0.042	0.045	0.042	0.061	0.056	0.050	0.061	0.056	0.063	0.046	0.015	0.033	0.024	0.039	0.045	0.123	0.048	0.113	0.062	0.068	0.075	0.082	0.046	0.062	0.022	0.046	0.044	0.121	0.078	0.056	0.166	0.172	0.197	0.146	0.214	0.142	0.179	0.204	0.330	0.371	0.264	0.387	0.329	0.230	0.125
F594_71.0138_15.4_N	0.145	0.050	0.137	0.136	0.147	0.145	0.241	0.274	0.213	0.162	0.178	0.163	0.242	0.239	0.206	0.271	0.224	0.185	0.220	0.189	0.280	0.402	0.392	0.183	0.343	0.156	0.170	0.182	0.285	0.299	0.299	0.307	0.321	0.287	0.241	0.221	0.327	0.295	0.109	0.091	0.111	0.149	0.162	0.096	0.145	0.143	0.143	0.164	0.176	0.147	0.122	0.204	0.187
F595_512.2683_3.9_N	0.083	0.008	0.043	0.025	0.015	0.005	0.023	0.002	0.002	0.003	0.067	0.001	0.002	0.057	0.002	0.118	0.001	0.010	0.036	0.100	0.001	0.007	0.042	0.069	0.001	0.021	0.171	0.132	0.022	0.077	0.101	0.001	0.089	0.001	0.001	0.011	0.019	0.002	0.034	0.084	0.082	0.027	0.030	0.154	0.071	0.062	0.001	0.001	0.001	0.001	0.258	0.001	0.003
F596_84.0443_15.4_P	0.304	0.173	0.288	0.288	0.280	0.272	0.176	0.332	0.404	0.327	0.353	0.282	0.399	0.217	0.333	0.313	0.348	0.347	0.306	0.386	0.394	0.362	0.358	0.278	0.409	0.278	0.335	0.348	0.350	0.304	0.403	0.402	0.447	0.412	0.370	0.343	0.386	0.371	0.228	0.248	0.267	0.230	0.265	0.175	0.229	0.237	0.244	0.240	0.297	0.239	0.321	0.264	0.199
F597_400.182_11.2_P	0.075	0.032	0.078	0.069	0.067	0.108	0.025	0.081	0.083	0.053	0.084	0.078	0.075	0.046	0.067	0.088	0.107	0.074	0.065	0.105	0.126	0.083	0.056	0.061	0.067	0.096	0.064	0.069	0.091	0.106	0.145	0.096	0.161	0.106	0.111	0.113	0.128	0.132	0.172	0.181	0.129	0.118	0.193	0.105	0.201	0.233	0.114	0.093	0.142	0.179	0.198	0.121	0.075
F598_344.2274_4.4_P	0.015	0.008	0.013	0.007	0.011	0.005	0.017	0.014	0.531	0.007	0.007	0.012	0.014	0.040	0.011	0.008	0.011	0.007	0.007	0.013	0.013	0.016	0.009	0.010	0.007	0.010	0.015	0.007	0.005	0.012	0.005	0.010	0.011	0.013	0.011	0.008	0.013	0.020	0.007	0.007	0.008	0.008	0.007	0.006	0.008	0.007	0.004	0.010	0.019	0.006	0.011	0.005	0.010
F599_178.0718_10.4_P	0.100	0.067	0.126	0.103	0.125	0.121	0.094	0.110	0.109	0.113	0.078	0.093	0.053	0.127	0.067	0.096	0.088	0.074	0.101	0.089	0.072	0.052	0.066	0.096	0.101	0.095	0.090	0.136	0.081	0.105	0.088	0.081	0.076	0.093	0.140	0.131	0.124	0.132	0.191	0.159	0.160	0.292	0.234	0.165	0.269	0.212	0.226	0.196	0.349	0.324	0.235	0.165	0.333
F600_198.0846_16.3_P	0.114	0.040	0.182	0.140	0.159	0.201	0.060	0.181	0.213	0.124	0.221	0.147	0.184	0.030	0.178	0.159	0.171	0.135	0.118	0.163	0.218	0.174	0.157	0.223	0.211	0.179	0.204	0.138	0.200	0.156	0.200	0.298	0.300	0.281	0.269	0.202	0.179	0.163	0.226	0.178	0.251	0.169	0.252	0.178	0.210	0.202	0.173	0.126	0.135	0.168	0.193	0.204	0.073
F601_826.5361_3.2_P	0.904	1.360	0.465	0.876	0.155	0.357	1.095	0.300	0.524	0.482	0.251	0.453	0.287	1.672	0.194	0.240	0.278	0.840	1.011	0.559	0.372	0.252	0.376	0.316	0.380	0.288	0.321	0.422	0.201	0.429	0.333	0.343	0.363	0.407	0.467	0.317	0.329	0.425	0.395	0.156	0.482	1.040	0.550	0.341	0.442	0.363	0.179	0.259	0.206	0.211	0.166	0.222	0.470
F602_113.0244_15.4_N	0.199	0.009	0.133	0.153	0.160	0.158	0.252	0.241	0.206	0.166	0.249	0.122	0.228	0.234	0.205	0.214	0.228	0.223	0.220	0.218	0.280	0.325	0.395	0.150	0.345	0.142	0.188	0.190	0.274	0.252	0.301	0.309	0.308	0.354	0.295	0.191	0.263	0.275	0.118	0.090	0.123	0.173	0.185	0.122	0.135	0.145	0.182	0.175	0.176	0.112	0.152	0.168	0.191
F603_842.5521_3.4_N	0.356	0.789	0.181	0.353	0.217	0.285	0.688	0.244	0.233	0.125	0.260	0.242	0.198	1.386	0.311	0.215	0.091	0.459	0.490	0.358	0.262	0.241	0.131	0.314	0.299	0.372	0.253	0.316	0.260	0.281	0.392	0.343	0.200	0.145	0.268	0.232	0.276	0.281	0.116	0.246	0.183	0.279	0.201	0.224	0.193	0.176	0.251	0.254	0.340	0.164	0.211	0.224	0.433
F604_349.2731_3.2_P	0.049	0.102	0.024	0.050	0.029	0.037	0.005	0.051	0.046	0.062	0.020	0.027	0.036	0.196	0.045	0.026	0.025	0.063	0.023	0.029	0.030	0.042	0.020	0.042	0.027	0.045	0.037	0.046	0.029	0.043	0.032	0.022	0.033	0.022	0.029	0.027	0.037	0.028	0.065	0.133	0.073	0.089	0.120	0.168	0.075	0.088	0.056	0.078	0.074	0.048	0.030	0.058	0.046
F605_145.097_15.1_P	0.098	0.035	0.094	0.087	0.074	0.204	0.047	0.112	0.051	0.025	0.031	0.116	0.080	0.286	0.078	0.092	0.144	0.093	0.057	0.079	0.053	0.065	0.114	0.089	0.067	0.071	0.104	0.056	0.151	0.129	0.058	0.089	0.034	0.055	0.109	0.143	0.120	0.058	0.267	0.224	0.263	0.254	0.283	0.271	0.267	0.246	0.048	0.051	0.039	0.137	0.136	0.038	0.055
F606_408.1407_10_N	0.142	0.037	0.138	0.080	0.228	0.255	0.029	0.153	0.234	0.291	0.284	0.382	0.291	0.011	0.376	0.326	0.301	0.200	0.156	0.219	0.184	0.176	0.142	0.106	0.259	0.148	0.230	0.200	0.175	0.266	0.179	0.235	0.190	0.216	0.288	0.260	0.327	0.214	0.245	0.279	0.250	0.171	0.201	0.231	0.268	0.264	0.205	0.164	0.247	0.166	0.175	0.170	0.105
F607_90.0548_14.3_P	0.223	0.166	0.169	0.176	0.206	0.271	0.144	0.248	0.190	0.187	0.322	0.250	0.269	0.104	0.281	0.321	0.252	0.266	0.195	0.243	0.265	0.357	0.277	0.268	0.256	0.257	0.306	0.291	0.262	0.215	0.330	0.356	0.236	0.289	0.276	0.279	0.350	0.185	0.209	0.319	0.212	0.188	0.238	0.375	0.228	0.292	0.336	0.322	0.376	0.366	0.316	0.308	0.248
F608_159.0275_10.3_P	0.243	0.192	0.296	0.266	0.301	0.302	0.010	0.266	0.276	0.285	0.190	0.230	0.146	0.129	0.176	0.251	0.213	0.179	0.254	0.205	0.176	0.129	0.167	0.204	0.230	0.211	0.226	0.300	0.187	0.246	0.215	0.215	0.189	0.226	0.318	0.307	0.289	0.328	0.509	0.470	0.434	0.628	0.601	0.502	0.573	0.493	0.495	0.401	0.745	0.712	0.478	0.385	0.845
F609_499.348_4.1_P	0.458	0.420	0.427	0.512	0.281	0.241	0.394	0.270	0.350	0.250	0.276	0.355	0.232	0.168	0.239	0.153	0.240	0.290	0.390	0.449	0.272	0.239	0.260	0.296	0.292	0.299	0.192	0.348	0.262	0.238	0.265	0.201	0.263	0.188	0.255	0.237	0.182	0.201	0.273	0.211	0.271	0.419	0.294	0.141	0.244	0.227	0.303	0.286	0.346	0.291	0.240	0.241	0.240
F610_168.0765_14.2_P	0.213	0.079	0.162	0.112	0.149	0.361	0.086	0.215	0.112	0.077	0.111	0.287	0.160	0.067	0.160	0.276	0.198	0.239	0.108	0.137	0.178	0.254	0.281	0.199	0.128	0.140	0.226	0.205	0.319	0.283	0.152	0.247	0.221	0.269	0.215	0.270	0.191	0.172	0.185	0.211	0.199	0.135	0.193	0.207	0.182	0.149	0.075	0.172	0.096	0.242	0.383	0.124	0.070
F611_325.1247_15.4_N	0.857	1.217	0.217	0.597	0.102	0.119	1.252	0.199	0.357	0.275	0.288	0.176	0.304	1.522	0.220	0.240	0.259	0.806	1.274	0.304	0.303	0.412	0.596	0.136	0.464	0.185	0.136	0.288	0.187	0.305	0.551	0.398	0.472	0.668	0.438	0.214	0.266	0.477	0.088	0.075	0.125	0.333	0.096	0.056	0.108	0.093	0.084	0.073	0.131	0.073	0.083	0.086	0.671
F612_232.1538_8.2_P	0.088	0.051	0.087	0.043	0.093	0.112	0.067	0.075	0.065	0.088	0.109	0.167	0.063	0.046	0.100	0.117	0.079	0.072	0.063	0.076	0.055	0.096	0.080	0.098	0.073	0.073	0.142	0.096	0.194	0.152	0.088	0.079	0.108	0.092	0.077	0.138	0.093	0.058	0.155	0.190	0.193	0.141	0.167	0.313	0.163	0.167	0.185	0.136	0.196	0.176	0.280	0.293	0.128
F613_498.3452_4.1_P	0.337	0.530	0.465	0.523	0.372	0.284	0.340	0.310	0.474	0.357	0.268	0.423	0.307	0.167	0.349	0.233	0.273	0.278	0.385	0.431	0.289	0.256	0.343	0.341	0.351	0.395	0.275	0.419	0.315	0.221	0.263	0.214	0.263	0.213	0.268	0.247	0.215	0.215	0.332	0.268	0.216	0.401	0.247	0.267	0.276	0.198	0.308	0.300	0.291	0.185	0.200	0.214	0.246
F614_478.2923_4_P	1.071	1.067	1.018	0.967	0.797	0.628	0.724	0.857	0.897	0.746	0.512	0.882	0.667	0.552	0.612	0.342	0.710	0.714	0.938	0.955	0.616	0.800	0.801	0.673	0.695	0.690	0.454	0.733	0.456	0.470	0.496	0.414	0.531	0.511	0.540	0.515	0.393	0.531	0.682	0.507	0.522	1.017	0.657	0.524	0.663	0.521	0.527	0.522	0.491	0.469	0.284	0.511	0.565
F615_121.0838_10.1_P	0.124	0.076	0.186	0.131	0.250	0.242	0.063	0.238	0.200	0.125	0.227	0.203	0.253	0.031	0.254	0.234	0.217	0.142	0.115	0.199	0.243	0.232	0.191	0.178	0.186	0.195	0.221	0.174	0.243	0.235	0.208	0.257	0.263	0.218	0.247	0.201	0.282	0.188	0.275	0.318	0.385	0.205	0.307	0.287	0.333	0.341	0.192	0.169	0.210	0.212	0.323	0.210	0.109
F616_311.2954_3.3_N	0.129	0.200	0.210	0.204	0.376	0.346	0.125	0.350	0.299	0.227	0.285	0.254	0.387	0.153	0.335	0.260	0.260	0.143	0.133	0.239	0.260	0.354	0.244	0.409	0.270	0.269	0.279	0.190	0.337	0.215	0.212	0.205	0.232	0.138	0.108	0.235	0.159	0.151	0.175	0.168	0.126	0.090	0.176	0.197	0.091	0.176	0.165	0.137	0.168	0.185	0.209	0.198	0.102
F617_86.0599_14.8_P	0.248	0.192	0.216	0.247	0.220	0.316	0.136	0.397	0.324	0.217	0.305	0.175	0.375	0.600	0.272	0.261	0.354	0.338	0.285	0.411	0.392	0.271	0.275	0.207	0.356	0.151	0.285	0.289	0.343	0.383	0.383	0.380	0.520	0.292	0.323	0.374	0.352	0.549	0.174	0.137	0.159	0.194	0.236	0.155	0.146	0.187	0.305	0.219	0.348	0.326	0.305	0.320	0.242
F618_864.5388_3.3_N	0.515	1.036	0.339	0.478	0.232	0.226	0.855	0.350	0.549	0.461	0.365	0.295	0.314	1.999	0.196	0.213	0.689	0.411	0.602	0.336	0.355	0.254	0.284	0.298	0.298	0.364	0.130	0.404	0.187	0.369	0.504	0.377	0.371	0.419	0.390	0.262	0.389	0.423	0.438	0.271	0.320	0.552	0.191	0.181	0.284	0.196	0.369	0.399	0.380	0.362	0.234	0.355	0.677
F619_862.6282_3.4_P	1.088	0.785	0.608	1.041	0.230	0.631	0.202	0.341	1.022	1.285	1.064	0.577	0.448	1.795	0.270	0.490	0.677	1.116	0.934	0.629	0.237	0.411	0.654	0.511	0.694	0.581	0.522	0.663	0.389	0.865	0.851	0.841	0.715	1.188	1.151	0.807	0.833	0.788	0.618	0.224	0.370	0.857	0.328	0.206	0.577	0.364	0.614	0.451	0.851	0.559	0.551	0.450	1.883
F620_838.5234_3.3_N	0.405	0.882	0.304	0.315	0.228	0.314	0.677	0.475	0.414	0.350	0.361	0.362	0.408	1.866	0.415	0.364	0.517	0.396	0.599	0.405	0.496	0.282	0.433	0.266	0.260	0.367	0.353	0.455	0.386	0.509	0.513	0.348	0.278	0.508	0.439	0.397	0.396	0.513	0.325	0.167	0.310	0.368	0.384	0.206	0.378	0.213	0.411	0.293	0.330	0.429	0.324	0.461	0.569
F621_174.1235_7.4_P	0.012	0.033	0.272	0.314	0.357	0.447	0.142	0.363	0.256	0.129	0.183	0.266	0.302	0.153	0.265	0.272	0.271	0.283	0.176	0.218	0.185	0.222	0.228	0.355	0.214	0.185	0.260	0.160	0.376	0.240	0.152	0.343	0.159	0.322	0.191	0.339	0.342	0.202	0.292	0.215	0.255	0.010	0.222	0.256	0.183	0.185	0.200	0.195	0.171	0.253	0.253	0.168	0.137

F622_744.5887_3.4_P	0.701	1.505	0.617	1.265	0.568	0.525	1.277	0.293	0.593	0.892	0.604	0.277	0.342	3.287	0.625	0.719	0.763	1.384	1.564	1.006	0.712	1.085	0.497	0.402	0.368	0.295	0.721	0.884	0.872	0.881	0.492	0.887	0.943	0.793	0.456	0.629	0.862	0.714	0.259	0.473	0.197	0.835	0.397	0.293	0.539	0.390	0.550	0.531	0.634	0.998	0.578	0.595	1.835
F623_273.1471_25.5_P	0.293	0.223	0.241	0.257	0.280	0.281	0.213	0.356	0.261	0.268	0.265	0.214	0.207	0.083	0.197	0.308	0.311	0.260	0.198	0.265	0.234	0.278	0.260	0.336	0.302	0.336	0.264	0.291	0.301	0.423	0.225	0.230	0.262	0.270	0.291	0.291	0.238	0.356	0.320	0.311	0.338	0.263	0.340	0.299	0.382	0.261	0.423	0.400	0.466	0.439	0.400	0.400	0.362
F624_178.072_11.2_N	0.144	0.176	0.152	0.151	0.270	0.274	0.106	0.314	0.195	0.181	0.181	0.232	0.269	0.074	0.233	0.272	0.222	0.163	0.105	0.197	0.190	0.192	0.172	0.294	0.280	0.210	0.376	0.267	0.311	0.230	0.166	0.242	0.165	0.160	0.157	0.226	0.218	0.180	0.201	0.188	0.170	0.136	0.174	0.223	0.162	0.225	0.243	0.252	0.171	0.187	0.135	0.282	0.158
F625_269.2123_3.4_N	0.162	0.170	0.245	0.200	0.360	0.310	0.147	0.271	0.262	0.185	0.280	0.254	0.293	0.242	0.299	0.257	0.198	0.158	0.109	0.201	0.209	0.267	0.265	0.317	0.272	0.223	0.225	0.188	0.244	0.214	0.180	0.161	0.177	0.182	0.104	0.194	0.146	0.192	0.161	0.147	0.113	0.092	0.192	0.169	0.146	0.181	0.150	0.163	0.144	0.190	0.145	0.194	0.111
F626_517.3299_3.8_P	0.353	0.033	0.033	0.022	0.011	0.011	0.854	0.012	0.016	0.019	0.054	0.014	0.013	0.119	0.012	0.379	0.014	0.019	0.028	0.020	0.013	0.012	0.020	0.041	0.013	0.013	0.123	0.309	0.011	0.022	0.064	0.012	0.051	0.014	0.013	0.011	0.013	0.016	0.013	0.120	0.045	0.018	0.012	0.233	0.017	0.016	0.013	0.011	0.014	0.012	0.284	0.010	0.029
F627_473.2817_3.1_N	0.177	0.128	0.301	0.317	0.263	0.410	0.148	0.386	0.388	0.287	0.267	0.234	0.446	0.093	0.416	0.315	0.344	0.208	0.218	0.282	0.310	0.439	0.290	0.296	0.257	0.262	0.273	0.184	0.227	0.603	0.475	0.421	0.443	0.461	0.388	0.274	0.488	0.736	0.210	0.173	0.288	0.182	0.312	0.189	0.278	0.370	0.260	0.240	0.244	0.255	0.254	0.325	0.169
F628_300.2014_4.5_P	0.025	0.015	0.024	0.010	0.018	0.018	0.019	0.035	0.648	0.011	0.017	0.019	0.024	0.059	0.022	0.016	0.028	0.046	0.013	0.063	0.073	0.052	0.045	0.033	0.048	0.035	0.040	0.032	0.018	0.040	0.010	0.047	0.076	0.078	0.076	0.030	0.068	0.082	0.034	0.024	0.020	0.024	0.023	0.033	0.017	0.037	0.027	0.048	0.073	0.048	0.094	0.018	0.033
F629_291.0692_10.9_P	0.756	2.266	0.287	0.053	0.132	0.150	0.017	0.154	0.333	1.098	0.238	0.290	0.244	1.927	0.223	0.153	0.268	0.672	0.045	0.496	0.204	0.150	0.172	0.146	0.254	0.197	0.123	0.328	0.131	0.228	0.296	0.210	0.228	0.341	0.400	0.216	0.223	0.493	0.157	0.128	0.163	0.454	0.180	0.175	0.212	0.150	0.299	0.182	0.337	0.179	0.204	0.166	0.527
F630_247.1396_14.2_P	0.112	0.050	0.080	0.088	0.148	0.149	0.040	0.128	0.112	0.103	0.145	0.140	0.140	0.029	0.106	0.183	0.112	0.139	0.098	0.142	0.157	0.190	0.142	0.149	0.254	0.176	0.345	0.189	0.335	0.285	0.446	0.404	0.357	0.548	0.514	0.350	0.456	0.155	0.088	0.100	0.091	0.060	0.096	0.052	0.095	0.110	0.229	0.228	0.307	0.321	0.339	0.257	0.149
F631_495.3265_4_P	0.428	0.392	0.398	0.415	0.351	0.254	0.384	0.288	0.418	0.299	0.225	0.350	0.265	0.185	0.287	0.205	0.275	0.283	0.315	0.382	0.235	0.239	0.311	0.296	0.307	0.335	0.230	0.320	0.248	0.213	0.204	0.192	0.220	0.181	0.210	0.240	0.177	0.195	0.358	0.178	0.239	0.367	0.300	0.247	0.311	0.233	0.202	0.263	0.237	0.282	0.150	0.209	0.196
F632_225.1229_14.9_P	0.109	0.074	0.089	0.115	0.098	0.213	0.080	0.156	0.106	0.045	0.045	0.130	0.157	0.080	0.125	0.108	0.132	0.120	0.094	0.098	0.095	0.096	0.106	0.108	0.097	0.079	0.163	0.091	0.168	0.154	0.096	0.153	0.065	0.074	0.086	0.202	0.198	0.131	0.253	0.183	0.180	0.185	0.290	0.296	0.112	0.178	0.081	0.055	0.090	0.172	0.148	0.057	0.071
F633_126.0771_8.4_P	0.019	0.004	0.005	0.002	0.001	0.002	0.005	0.454	0.018	0.003	0.001	0.001	0.001	0.024	0.001	0.001	0.001	0.001	0.003	0.001	0.031	0.001	0.001	0.001	0.001	0.003	0.005	0.160	0.001	0.019	0.001	0.001	0.001	0.001	0.004	0.002	0.002	0.004	0.001	0.002	0.001	0.001	0.002	0.001	0.001	0.001	0.001	0.001	0.014	0.001	0.001	0.001	0.005
F634_528.2932_4.2_N	0.187	0.158	0.177	0.170	0.177	0.138	0.130	0.181	0.211	0.113	0.133	0.138	0.127	0.053	0.140	0.109	0.158	0.142	0.145	0.174	0.174	0.159	0.161	0.150	0.153	0.165	0.129	0.143	0.165	0.115	0.132	0.099	0.123	0.098	0.110	0.117	0.087	0.094	0.164	0.104	0.133	0.148	0.156	0.099	0.133	0.109	0.136	0.149	0.139	0.132	0.114	0.128	0.093
F635_285.0824_13.1_P	0.010	0.013	0.012	0.006	0.010	0.017	0.013	0.008	0.005	0.005	0.003	0.005	0.003	0.052	0.007	0.005	0.006	0.005	0.008	0.006	0.005	0.006	0.004	0.030	0.004	0.024	0.019	0.017	0.015	0.036	0.024	0.019	0.031	0.015	0.020	0.029	0.016	0.035	0.399	0.660	0.360	0.430	0.495	0.739	0.576	0.428	0.323	0.310	0.391	0.540	0.577	0.199	0.333
F636_143.0482_15.9_P	0.271	0.140	0.280	0.178	0.476	0.468	0.149	0.442	0.328	0.346	0.452	0.347	0.404	0.175	0.390	0.598	0.434	0.210	0.235	0.365	0.412	0.377	0.361	0.480	0.379	0.416	0.521	0.526	0.622	0.511	0.307	0.361	0.431	0.369	0.330	0.515	0.393	0.501	0.473	0.535	0.512	0.330	0.568	0.464	0.523	0.544	0.770	0.911	0.665	0.781	0.701	0.971	0.407
F637_120.0806_10_P	1.599	1.166	2.394	1.664	3.225	3.019	1.007	3.002	2.542	1.650	2.895	2.545	3.215	0.899	3.254	2.999	2.774	1.954	1.554	2.548	3.094	2.981	2.430	2.335	2.442	2.527	2.758	2.265	2.958	3.006	2.714	3.294	3.291	2.816	3.155	2.596	3.528	2.433	3.557	4.129	4.658	2.506	3.927	3.520	4.201	4.026	2.467	2.155	2.719	2.737	4.040	2.698	1.470
F638_830.5674_3.2_P	9.910	17.37	5.833	10.74	4.120	3.720	14.26	5.431	6.627	8.559	5.953	5.400	5.045	24.42	5.117	4.437	6.629	10.01	13.08	7.729	5.638	4.365	4.524	5.820	5.251	4.271	4.103	4.936	4.539	6.689	5.139	5.021	5.143	7.178	6.706	4.503	6.434	8.215	4.433	3.536	4.374	10.42	5.331	3.240	3.898	3.344	4.869	4.218	5.522	4.435	2.840	4.306	12.77
F639_148.0965_12_P	3.219	2.551	3.669	4.597	2.156	3.141	2.968	3.414	4.363	3.699	6.503	3.065	4.223	2.050	3.398	4.747	5.266	5.773	4.345	4.314	6.114	5.766	9.060	2.963	5.297	2.733	2.539	3.660	3.878	5.904	5.351	3.965	5.910	6.782	4.356	5.715	4.922	7.891	1.458	2.033	4.247	2.642	4.215	3.477	2.868	2.307	3.274	2.844	3.765	5.399	4.626	4.089	3.175
F640_229.1543_12.8_P	1.585	0.868	1.828	1.423	1.868	2.392	1.112	1.539	1.282	1.268	1.401	1.784	1.702	0.906	1.751	2.676	1.794	1.934	1.416	1.331	1.259	1.213	1.873	1.883	1.058	1.566	2.007	1.539	3.519	2.781	2.073	1.809	1.231	3.000	2.455	2.183	3.083	1.895	2.860	1.839	2.761	2.647	2.015	1.654	1.540	1.542	2.258	1.752	2.059	3.062	3.949	2.360	2.207
F641_253.2174_3.4_N	1.878	2.691	3.910	2.812	4.639	4.171	1.671	3.788	3.530	2.139	3.743	3.273	4.666	1.098	4.065	3.437	2.660	1.818	1.280	2.890	3.288	3.510	3.426	4.156	3.564	3.473	3.083	2.859	2.805	2.804	2.785	2.294	2.730	2.344	1.674	2.788	2.033	2.715	2.149	2.027	1.523	0.987	2.229	2.481	1.959	2.298	2.041	2.130	1.941	2.605	2.320	2.596	1.301
F642_227.2017_3.5_N	1.444	2.160	2.949	1.978	3.618	3.143	1.194	2.755	3.088	1.587	2.959	2.603	3.341	1.249	3.018	2.330	2.099	1.520	1.134	2.366	2.349	2.934	2.659	3.435	2.582	2.531	2.249	1.853	2.449	1.950	2.203	1.695	1.779	1.721	1.096	1.925	1.226	1.746	1.588	1.442	1.036	0.737	1.623	1.534	1.404	1.759	1.369	1.298	1.185	1.783	1.403	1.740	0.855
F643_171.0761_9.3_P	1.227	0.315	0.590	0.329	0.470	1.434	0.261	0.429	0.394	1.088	0.724	5.973	0.502	0.367	0.716	2.431	2.117	0.830	1.634	0.843	0.253	0.783	1.534	0.257	0.505	0.301	0.856	0.576	1.743	0.703	0.548	0.424	0.364	0.693	1.127	0.701	1.014	0.897	3.027	7.443	2.421	1.248	1.268	1.120	4.899	2.750	1.055	0.749	1.037	1.142	1.583	0.916	1.030
F644_220.1175_8.8_P	1.892	1.038	1.737	1.253	1.918	1.786	0.902	1.599	1.428	2.272	1.749	2.595	1.474	1.006	1.864	1.960	1.919	1.690	1.224	1.843	0.939	1.158	1.286	2.064	1.982	1.963	2.625	2.674	3.083	1.824	1.758	1.301	1.209	1.403	2.204	2.280	2.041	1.108	2.551	2.795	2.098	2.413	2.102	2.457	2.864	2.172	2.359	2.437	2.972	2.933	3.159	2.159	2.003
F645_866.5543_3.3_N	3.158	6.992	3.254	6.091	0.862	2.895	4.973	4.121	4.380	4.461	2.722	3.698	4.064	23.18	4.555	2.980	4.279	4.108	6.130	4.503	5.816	3.252	3.733	3.690	3.662	3.196	2.745	4.202	2.824	4.228	4.287	4.453	4.329	4.271	4.063	3.861	5.031	3.938	3.331	2.103	3.244	4.393	2.716	2.053	3.001	2.562	3.858	3.814	4.200	2.844	2.362	3.613	6.733
F646_72.0807_12.6_P	1.743	1.382	1.700	1.818	1.353	1.656	1.207	1.767	1.905	1.464	1.868	1.771	2.092	1.156	2.248	2.271	2.291	2.229	1.650	1.998	2.548	2.090	1.913	1.481	1.824	1.713	1.638	1.731	1.985	1.933	2.787	2.675	2.809	2.231	3.070	2.315	2.496	2.213	2.442	2.301	2.584	2.037	2.245	2.761	2.606	2.511	2.382	1.816	2.548	2.774	2.999	2.454	1.934
F647_98.9668_15.2_N	1.999	0.014	2.150	2.155	3.022	2.849	2.057	3.638	2.613	2.004	2.013	1.983	3.066	0.922	3.599	3.419	2.413	2.545	2.197	2.739	3.323	3.249	2.595	2.818	2.493	2.512	3.007	2.189	2.709	3.041	3.937	2.219	2.861	2.860	3.520	3.108	4.218	4.138	2.394	2.344	2.322	2.106	2.635	1.974	2.866	3.043	2.991	2.916	2.921	2.897	3.342	3.211	2.997
F648_284.0983_13_P	0.427	0.382	0.482	0.406	1.234	1.043	0.228	0.801	0.430	0.480	0.598	0.885	0.623	0.221	0.865	0.724	1.242	0.450	0.452	0.566	0.669	0.601	0.494	0.764	0.987	0.616	1.737	1.161	1.386	1.524	0.793	1.070	0.808	0.894	0.675	1.248	1.077	1.311	2.259	1.869	1.800	1.655	2.659	1.492	2.714	2.419	2.040	1.819	1.965	3.498	2.140	3.164	1.534
F649_521.3423_3.9_P	2.682	2.696	2.136	2.219	1.746	1.292	2.541	1.595	2.435	1.935	1.330	2.207	1.703	1.530	1.707	1.172	1.528	2.125	2.141	2.077	1.554	1.737	1.780	1.695	1.844	2.179	1.677	2.138	1.568	1.374	1.602	1.219	1.397	1.141	1.440	1.391	1.003	1.067	1.740	1.756	1.699	2.655	1.778	1.675	2.100	1.456	1.359	1.136	1.493	1.282	1.100	1.174	1.259
F650_70.0651_12.9_P	2.809	0.815	2.002	0.991	0.578	0.666	1.895	1.376	1.642	1.145	2.279	0.590	0.688	1.073	0.618	0.746	2.613	2.126	3.495	1.515	3.203	2.495	2.664	0.877	1.355	1.244	1.124	1.243	1.367	1.134	2.158	0.770	1.686	1.198	1.264	1.817	0.686	1.251	1.281	1.789	1.437	1.714	1.828	1.890	1.630	2.214	1.070	0.899	0.982	0.927	1.252	0.649	0.862
F651_498.2887_3.8_N	1.194	0.061	0.162	0.075	0.046	0.029	2.355	0.039	0.033	0.022	0.311	0.045	0.025	0.408	0.034	2.762	0.016	0.035	0.122	0.081	0.017	0.038	0.149	0.334	0.020	0.071	1.370	1.639	0.111	0.159	0.391	0.016	0.263	0.013	0.023	0.019	0.028	0.013	0.114	1.202	0.262	0.102	0.101	1.734	0.139	0.161	0.017	0.022	0.016	0.014	1.987	0.012	0.014
F652_497.3421_4_P	2.974	4.672	3.394	3.907	2.633	2.048	3.488	2.138	3.154	2.238	1.857	2.781	2.193	1.747	2.425	1.346	1.869	2.613	2.608	3.354	2.081	2.065	2.219	2.429	2.434	2.822	1.944	2.775	2.088	1.625	1.743	1.595	1.886	1.437	1.648	1.788	0.773	1.478	2.318	1.824	1.608	3.010	1.885	2.086	2.070	1.568	1.871	1.856	1.749	1.700	1.429	1.556	1.828
F653_782.567_3.4_P	6.372	15.03	3.242	4.849	4.189	4.244	15.51	5.445	5.110	8.420	4.319	5.508	3.501	22.35	7.059	5.110	3.949	6.560	10.53	4.137	5.266	3.461	5.148	4.287	3.138	3.572	4.608	5.555	2.961	4.299	6.120	5.068	6.018	3.646	5.962	4.903	6.309	8.471	4.116	3.602	4.087	6.718	3.501	4.047	5.439	4.076	4.050	3.359	5.639	2.865	3.135	3.208	10.45
F654_86.0962_10.7_P	0.986	0.680	0.974	0.919	0.716	0.694	0.604	0.923	1.167	0.704	0.946	0.823	0.953	0.536	1.104	1.024	1.316	1.141	0.979	1.349	1.647	1.203	0.971	0.635	0.805	0.964	0.813	0.812	0.913	0.920	1.573	1.464	1.887	1.134	1.644	1.158	1.250	1.326	1.322	1.166	1.670	1.477	1.563	2.023	1.725	1.816	1.323	1.099	1.498	1.526	1.963	1.346	0.927
F655_367.2836_3.5_P	2.524	5.256	1.233	3.435	0.882	0.978	4.379	1.573	1.794	2.000	0.451	0.736	0.943	3.033	1.305	0.747	0.898	1.999	1.919	1.500	0.944	1.181	0.930	1.194	0.715	1.465	0.780	1.324	0.664	1.310	1.156	1.011	0.917	1.095	1.386	1.137	1.317	1.730	1.942	2.526	1.952	3.975	2.849	3.011	2.243	2.402	1.870	2.035	2.401	1.488	0.844	1.826	3.729
F656_810.5974_3.5_P	6.348	7.257	2.839	4.074	1.505	3.118	8.243	2.415	5.324	7.233	3.457	4.562	3.784	4.200	3.366	1.848	5.090	4.765	6.777	3.176	4.316	3.945	3.391	4.567	4.090	3.070	3.627	5.975	2.905	5.641	4.535	5.507	4.083	3.129	3.524	4.816	2.751	4.897	1.664	1.930	1.226	4.405	2.250	2.211	3.003	2.352	5.903	4.851	6.449	6.613	2.908	3.273	9.444
F657_556.3247_4_N	1.530	1.631	1.707	1.542	1.943	1.239	1.598	1.275	1.944	0.959	1.070	1.549	1.411	1.108	1.429	1.059	1.233	1.477	1.411	1.356	1.314	1.276	1.199	1.637	1.460	1.741	1.377	1.546	1.118	0.880	1.182	0.921	1.283	0.786	1.008	1.046	0.995	0.756	1.343	0.671	0.977	1.611	1.162	1.187	0.995	0.916	1.088	1.184	0.954	0.908	1.021	0.730	0.941
F658_297.2435_3.4_N	2.002	3.011	4.578	3.157	4.987	5.594	1.960	3.933	4.077	2.660	4.569	3.855	5.532	1.571	4.787	3.717	2.824	2.013	1.703	3.828	3.416	4.501	4.081	4.915	4.091	3.786	3.245	2.795	3.813	3.053	3.011	2.419	3.009	2.509	1.814	2.907	1.776	2.619	2.343	2.363	1.573	1.153	2.556	2.797	2.261	3.003	1.867	1.873	2.031	2.692	2.581	2.528	1.456
F659_184.0636_11.5_P	0.757	1.154	1.925	0.970	0.941	1.168	0.609	1.139	0.787	1.384	1.960	1.564	1.260	0.428	2.618	1.506	2.558	0.846	0.544	0.731	1.340	0.907	1.862	1.398	1.032	2.471	1.068	2.589	1.628	0.997	0.920	0.617	0.614	1.028	0.567	1.220	0.915	1.702	0.393	0.426	0.530	0.519	0.782	0.536	0.628	0.635	1.014	0.724	0.558	0.705	0.615	0.670	0.414
F660_170.0477_9.7_P	1.184	0.490	1.011	0.648	1.602	1.903	0.670	1.603	1.099	1.201	1.702	0.976	1.657	0.557	1.014	1.546	1.274	0.757	0.794	1.504	1.073	1.580	1.145	1.692	1.297	1.401	2.231	1.805	2.734	2.291	1.108	1.253	1.839	1.162	1.326	2.096	1.341	1.433	2.105	2.265	2.471	1.955	2.360	2.356	2.633	2.825	3.305	5.192	2.980	3.618	5.234	4.448	2.205
F661_629.3282_3.8_N	1.500	1.693	1.274	1.646	0.734	1.198	1.168	1.272	1.556	0.997	1.088	1.474	1.256	0.927	1.206	0.947	1.289	0.945	1.189	1.386	1.504	1.139	1.263	1.304	1.140	1.420	1.179	1.539	1.052	0.961	0.832	0.820	1.184	0.893	0.863	0.988	0.832	0.886	1.946	1.328	1.560	1.930	1.788	1.312	1.597	0.870	1.023	1.053	1.123	1.096	0.802	0.820	0.963
F662_547.3578_3.9_P	9.632	10.14	8.334	8.520	7.331	5.130	7.834	6.394	7.624	5.606	4.202	7.334	5.387	4.973	5.616	4.550	4.983	6.906	6.267	7.300	5.835	5.709	5.864	5.910	5.677	6.523	4.651	7.294	4.528	5.226	4.555	4.477	4.774	4.455	5.216	5.483	3.933	5.028	9.645	7.778	7.140	10.20	8.861	9.209	7.175	5.931	5.348	5.061	5.822	4.926	4.289	4.165	5.728
F663_804.5526_3.3_P	13.32	21.33	7.520	14.70	4.941	5.960	22.86	11.07	10.81	10.68	5.820	7.092	7.310	40.86	9.481	6.039	8.333	11.27	19.21	12.96	10.84	8.567	14.24	5.962	5.958	5.531	4.120	5.751	5.457	13.04	7.714	8.214	5.192	9.064	12.63	11.13	12.02	15.01	4.566	3.734	5.499	16.27	8.337	4.710	8.301	7.121	7.039	6.230	5.975	4.965	3.563	7.858	22.83
F664_546.3548_3.9_P	35.67	37.08	31.11	32.63	29.98	19.54	32.21	24.93	26.71	22.24	18.32	19.90	20.77	19.43	24.80	15.55	18.64	24.92	24.47	27.40	20.59	18.88	20.92	25.05	23.72	23.89	17.02	30.10	16.25	18.00	17.50	12.89	17.79	15.69	20.22	13.86	15.68	18.58	33.24	31.08	27.51	38.66	34.87	32.48	25.67	23.32	21.60	19.10	23.56	17.97	14.80	17.83	16.62
F665_151.1438_10.7_P	4.641	1.299	8.474	3.952	21.72	24.78	1.156	12.60	9.928	10.75	17.09	20.20	15.10	0.148	13.93	13.43	9.744	5.446	4.047	4.474	5.074	7.437	8.836	17.41	18.46	13.73	26.00	12.68	22.30	8.104	6.989	5.510	5.207	9.777	3.114	10.18	12.87	2.676	34.69	34.26	15.94	3.553	15.42	16.59	11.26	13.01	3.107	5.490	2.582	4.201	5.684	4.105	1.137
F666_568.3391_3.9_P	35.28	36.04	29.40	30.92	28.69	18.20	29.48	23.29	25.62	21.37	17.63	18.60	19.19	17.95	22.84	14.32	18.14	24.17	23.36	26.03	19.37	17.61	19.38	23.62	23.09	22.85	16.29	28.48	15.37	16.74	17.01	12.15	16.63	14.86	18.90	12.90	14.45	17.44	32.21	29.79	26.57	36.82	32.86	30.37	23.79	23.91	20.99	18.43	22.57	17.01	14.03	16.78	15.43
F667_281.2484_3.4_N	10.81	18.03	29.09	17.73	27.82	30.15	11.81	21.28	21.02	15.29	25.30	21.13	30.44	9.300	29.44	21.28	16.94	10.72	9.216	20.49	17.95	24.53	21.95	27.72	22.48	22.31	19.64	16.40	23.22	16.02	20.63	16.26	19.22	14.11	10.75	16.81	12.68	16.21	9.766	10.61	7.005	4.161	10.59	12.48	8.222	12.41	11.15	10.2	12.04	16.51	15.23	14.49	7.244
F668_514.2834_4.2_N	10.28	0.371	1.000	0.322	0.114	0.059	44.74	0.077	0.038	0.024	1.788	0.036	0.052	7.595	0.063	28.46	0.025	0.088	1.394	0.869	0.017	0.074	1.135	1.915	0.020	0.308	9.511	13.52	0.332	1.861	6.194	0.027	8.930	0.017	0.052	0.059	0.130	0.020	0.479	9.059	3.220	0.533	0.446	18.36	1.441	2.926	0.017	0.038	0.023	0.021	23.02	0.015	0.033
F669_156.0322_14.3_P	7.447	7.751	5.573	5.975	7.144	7.398	8.291	8.269	6.517	8.337	7.832	6.906	7.675	11.98	6.979	7.728	7.113	6.372	6.990	7.550	6.802	6.923	6.587	8.746	6.989	8.353	10.59	10.94	10.97	10.77	7.491	7.666	8.261	6.145	6.894	10.36	8.284	9.508	9.140	12.67	11.77	10.66	11.56	11.22	11.00	12.42	20.61	28.00	17.78	18.63	18.77	22.41	18.91
F670_832.5834_3.3_P	40.99	45.85	22.21	41.22	8.990	26.95	53.22	13.62	29.64	33.02	17.54	18.30	9.267	86.90	20.17	24.16	26.62	38.49	53.06	39.55	28.38	22.92	9.039	11.89	21.79	10.20	9.126	22.61	11.45	37.48	23.87	31.43	15.97	13.15	32.45	24.56	21.25	33.48	14.69	8.947	10.84	40.83	12.27	14.14	23.54	15.96	29.67	21.85	28.86	21.24	17.58	16.25	62.95
F671_299.2591_3.4_N	10.85	18.12	29.13	17.79	27.86	30.17	11.88	21.31	21.06	15.35	25.34	21.18	30.50	9.358	29.50	21.30	16.99	10.77	9.256	20.54	18.00	24.56	21.97	27.74	22.52	22.35	19.67	16.44	23.26	16.05	20.67	16.33	19.28	14.16	10.78	16.85	12.74	16.26	9.772	10.61	7.007	4.162	10.59	12.50	8.224	12.41	11.19	10.24	12.07	16.53	15.25	14.51	7.272

F672_327.2326_3.3_N	7.292	10.58	11.41	11.60	16.94	13.95	7.27	13.86	12.92	8.88	15.39	10.87	17.09	4.495	16.19	13.35	9.627	5.518	5.047	10.70	11.29	9.59	9.523	16.54	13.13	12.53	12.56	10.36	12.25	10.54	10.54	8.09	9.841	8.311	5.912	9.234	6.927	7.717	7.580	6.817	5.019	3.873	8.821	6.812	6.489	7.736	6.600	7.707	6.903	9.509	7.710	6.192	5.435
F673_255.2329_3.4_N	5.719	10.51	13.80	10.25	14.05	14.40	6.253	10.05	12.08	8.084	13.70	12.13	16.83	5.38	14.79	10.98	9.684	5.721	5.054	9.081	10.35	11.64	10.30	15.52	13.81	11.86	11.29	9.062	11.37	8.581	11.15	7.256	9.102	7.466	4.941	9.870	5.374	7.836	6.073	7.291	3.870	2.569	6.014	7.186	4.868	5.991	6.284	4.820	6.704	10.576	8.132	6.792	4.152
F674_229.1543_9.6_P	4.759	2.587	6.896	4.120	7.126	8.517	2.971	5.865	5.033	4.235	4.489	6.435	5.774	2.329	6.775	9.884	6.308	5.743	4.151	4.624	4.098	4.555	8.024	5.729	4.212	5.217	11.344	5.629	9.722	6.149	5.826	5.647	3.613	6.068	7.420	9.413	10.551	5.418	12.681	12.088	7.908	7.337	7.236	9.126	5.681	7.222	6.660	5.096	6.788	10.20	10.04	5.436	4.837
F675_496.3389_4_P	12.34	13.45	15.92	15.66	8.758	8.334	15.02	8.458	13.14	9.149	7.403	11.41	10.77	7.822	9.822	5.310	7.835	10.83	10.96	13.67	8.235	8.377	8.993	10.30	10.13	11.17	8.10	11.69	8.653	6.727	7.226	6.415	7.260	5.770	8.118	8.464	5.047	5.802	9.276	7.189	6.854	13.25	7.574	7.833	7.727	6.228	7.514	7.374	6.516	6.473	5.742	6.420	7.688
F676_569.342_3.9_P	10.20	10.11	9.711	11.65	8.136	7.002	9.059	10.32	9.348	6.453	4.572	7.840	6.907	6.774	6.723	6.437	3.594	7.129	7.605	9.281	7.326	6.964	7.123	7.021	6.317	6.685	5.100	8.796	5.099	7.131	4.494	7.801	6.466	5.689	6.674	6.341	5.528	6.288	11.457	7.314	8.055	10.748	12.083	10.791	10.936	7.334	6.342	6.103	7.256	6.948	5.606	6.001	8.630
F677_520.3392_4_P	11.52	9.522	10.90	11.27	8.350	6.199	12.53	7.822	10.92	8.109	5.933	10.45	7.950	5.323	8.278	5.350	7.500	9.646	9.110	9.731	6.513	8.258	8.235	7.260	7.074	9.612	7.310	10.01	7.047	6.767	6.422	5.159	6.322	5.074	6.503	6.506	5.196	5.139	6.889	7.662	7.876	11.793	6.708	7.899	9.283	7.472	5.418	5.061	6.400	6.095	4.688	5.250	5.340
F678_205.0704_13.7_P	7.336	16.06	8.484	11.16	9.226	7.542	14.85	10.32	8.136	7.843	8.838	8.201	10.08	18.87	10.16	7.522	9.191	8.945	9.789	7.184	10.605	7.130	7.165	9.211	8.901	9.495	9.970	9.059	6.028	7.650	7.598	9.623	9.007	7.334	7.046	7.627	10.02	11.30	7.759	5.442	4.544	6.536	7.001	6.595	5.179	4.628	7.764	5.935	8.160	6.145	5.470	6.974	14.25
F679_542.3231_4_P	8.881	5.576	5.950	7.322	4.867	3.799	7.859	5.303	7.251	5.630	4.266	6.705	5.455	4.117	5.385	3.948	5.500	7.620	7.060	7.274	4.950	6.090	6.513	4.438	5.251	6.185	4.967	6.394	5.455	4.871	4.908	3.799	4.376	3.958	5.115	4.321	3.490	3.590	4.706	4.914	5.159	9.193	4.693	4.864	6.769	5.070	3.817	2.953	4.526	4.011	3.517	3.567	3.731
F680_533.3245_4.2_P	6.608	0.252	0.506	0.211	0.043	0.023	27.46	0.032	0.016	0.015	0.862	0.027	0.025	4.879	0.026	9.953	0.014	0.052	0.933	0.480	0.013	0.031	0.506	0.820	0.012	0.125	3.451	6.379	0.127	0.934	2.900	0.014	4.045	0.015	0.027	0.028	0.066	0.016	0.191	3.223	1.361	0.337	0.178	5.875	0.645	1.103	0.010	0.016	0.013	0.010	8.241	0.011	0.023
F681_522.3545_4_P	7.871	6.571	7.216	7.359	4.712	3.350	8.349	4.248	6.572	4.369	3.552	5.474	3.375	3.249	4.250	2.885	4.448	5.322	5.440	5.698	3.902	4.377	4.934	4.297	3.836	4.229	3.597	5.184	3.902	3.402	3.704	3.356	3.968	3.250	3.842	3.413	2.551	3.145	5.927	3.912	4.458	7.161	4.573	4.332	4.647	3.747	5.012	4.063	5.137	4.062	3.477	3.587	3.783
F682_132.0669_14.7_P	0.855	0.944	9.344	9.850	10.89	12.90	0.970	15.20	0.840	11.10	11.24	7.73	15.76	1.178	12.39	10.81	0.793	10.89	10.35	1.144	17.79	16.79	15.92	12.82	13.43	6.390	10.46	9.788	12.73	16.18	0.964	16.04	25.33	28.27	18.93	9.805	18.17	16.56	8.682	7.535	7.932	0.642	9.337	6.053	5.768	8.384	11.84	13.48	14.31	12.51	13.95	12.15	1.468
F683_258.9819_15.4_N	6.297	6.566	6.134	6.336	6.537	6.722	5.812	7.719	7.749	6.667	5.531	7.172	8.445	4.450	5.759	11.77	9.423	8.038	7.731	8.036	4.581	5.944	8.545	5.252	8.311	6.441	7.060	6.760	2.629	9.392	4.914	10.504	7.551	10.58	9.101	3.218	10.58	10.60	2.978	4.711	4.278	6.809	4.780	3.647	4.011	3.976	7.325	4.177	7.740	4.983	7.096	5.985	8.921
F684_808.5776_3.4_P	14.75	31.25	10.42	19.54	7.564	9.362	32.26	6.089	12.92	17.59	8.473	13.23	20.98	46.85	14.08	11.31	4.900	9.110	22.68	17.92	9.486	10.13	13.95	9.786	11.89	12.71	9.270	14.08	8.514	12.22	19.65	13.92	11.13	13.51	15.35	14.75	16.35	22.46	7.226	3.814	6.464	8.155	6.582	7.639	11.18	5.795	9.013	10.28	12.91	11.05	7.933	10.31	30.42
F686_179.0043_10.9_P	4.578	2.937	4.980	4.125	4.004	3.695	2.230	5.587	5.066	4.516	5.810	3.930	4.899	1.968	4.227	5.476	5.953	5.463	4.670	4.804	7.205	7.372	9.533	4.633	5.153	5.758	4.440	6.280	4.751	5.952	8.368	5.937	8.248	7.649	6.360	6.211	5.876	8.113	1.182	2.371	3.372	3.139	3.196	2.004	2.877	3.991	5.125	3.831	5.623	6.763	4.355	5.758	3.367
F687_628.3248_3.8_N	3.557	3.773	5.942	5.277	2.257	3.573	4.928	4.022	3.557	3.254	3.378	3.260	3.671	2.870	3.419	2.765	2.656	3.589	3.413	4.298	4.852	3.569	3.985	3.220	3.795	4.166	3.623	3.415	3.089	3.079	4.045	2.021	2.442	2.963	2.631	3.044	2.365	1.919	5.624	3.886	5.234	5.995	5.975	5.594	2.971	3.430	3.054	3.324	3.705	2.686	3.216	1.954	2.128
F688_173.1281_10.7_P	0.415	0.320	0.356	0.374	0.188	0.225	0.268	0.351	0.393	0.275	0.324	0.292	0.343	0.258	0.414	0.430	0.498	0.456	0.385	0.517	0.636	0.515	0.400	0.238	0.289	0.311	0.256	0.292	0.321	0.407	0.634	0.585	0.693	0.434	0.634	0.477	0.472	0.597	0.441	0.325	0.630	0.568	0.540	0.640	0.674	0.688	0.556	0.417	0.604	0.606	0.739	0.490	0.397
F689_852.5524_3.2_P	3.291	6.609	1.664	3.506	1.469	1.044	5.834	1.301	2.484	2.671	1.876	1.885	1.574	8.834	1.908	1.434	1.589	3.553	4.637	2.485	1.613	1.652	1.440	1.546	1.643	1.389	1.574	1.764	1.800	2.195	1.748	1.888	1.742	2.368	2.105	1.207	2.024	1.902	1.158	1.239	1.617	3.404	1.539	1.243	1.026	1.171	1.257	1.093	1.147	1.097	0.768	1.146	3.803
F690_478.2934_3.9_N	2.924	4.479	3.527	3.515	2.753	2.020	4.031	2.182	2.415	2.143	1.386	3.397	2.248	2.265	2.175	1.191	1.629	2.377	2.534	2.493	1.524	2.024	1.955	2.050	2.037	2.503	1.634	2.191	1.234	1.258	1.577	1.386	1.484	1.402	1.542	1.681	1.394	1.369	1.999	1.735	1.315	2.451	1.446	2.476	1.396	1.450	1.549	1.422	1.658	1.312	1.045	1.197	1.823
F691_828.552_3.2_P	2.707	4.098	1.391	2.880	0.736	0.849	2.810	1.301	1.435	1.843	1.255	1.074	1.113	6.669	1.023	1.011	1.006	2.456	3.378	1.983	0.879	1.068	1.189	0.720	0.877	0.881	0.650	1.195	0.967	1.638	1.433	1.234	1.263	1.824	1.533	0.992	0.917	1.586	0.971	0.757	1.381	2.888	1.300	0.740	1.313	0.780	1.251	0.898	1.187	1.145	0.689	1.087	3.207
F692_62.0602_25.5_P	0.608	0.426	0.785	0.641	0.979	0.909	0.464	1.028	0.555	0.643	0.788	0.631	0.671	0.260	0.763	0.835	0.767	0.554	0.396	0.629	0.676	0.748	0.735	1.055	0.784	0.904	0.912	0.938	0.929	1.007	0.580	0.516	0.741	0.622	0.672	0.768	0.602	0.819	0.963	0.990	1.043	0.761	1.145	0.895	1.122	1.053	1.182	1.138	1.184	1.269	1.338	1.304	0.795
F693_167.0481_15.9_P	0.376	0.201	0.411	0.270	0.692	0.657	0.223	0.759	0.452	0.490	0.651	0.503	0.586	0.195	0.571	0.839	0.603	0.301	0.348	0.538	0.608	0.535	0.522	0.642	0.540	0.613	0.772	0.785	0.926	0.750	0.421	0.510	0.617	0.540	0.473	0.723	0.555	0.730	0.688	0.710	0.743	0.503	0.819	0.638	0.783	0.785	1.181	1.293	0.993	1.128	0.989	1.378	0.564
F694_386.2062_27.1_P	1.323	0.992	1.121	1.619	1.252	1.339	0.543	1.369	1.714	1.497	1.135	1.265	1.669	0.863	1.192	1.280	1.216	1.794	1.481	1.551	1.680	1.528	1.111	1.077	1.514	1.327	1.326	0.939	1.611	1.573	2.174	2.126	2.187	2.325	2.460	1.731	1.347	1.549	1.071	0.865	0.779	1.012	1.147	0.707	1.133	0.899	1.600	1.282	1.526	1.621	1.689	1.235	1.404
F695_148.0791_15.4_P	1.927	1.115	1.677	1.670	1.393	1.475	1.088	1.815	2.138	1.728	1.819	1.785	2.158	1.235	1.794	1.641	1.818	2.087	1.869	1.910	2.147	1.950	2.093	1.477	2.114	1.497	1.670	1.938	1.809	1.690	2.271	2.218	2.424	2.265	2.143	1.901	1.908	2.079	1.266	1.279	1.375	1.260	1.295	0.886	1.278	1.272	1.281	1.095	1.603	1.246	1.618	1.261	1.062
F696_783.5709_3.5_P	2.654	4.854	2.683	3.194	1.362	1.681	3.108	1.870	2.584	2.087	1.635	1.121	1.528	7.939	1.452	0.299	2.524	2.739	4.545	2.555	1.538	1.653	2.204	1.525	1.940	1.721	1.434	2.489	1.113	1.598	2.372	1.569	2.180	1.601	2.313	0.997	2.638	2.594	0.996	1.110	1.412	2.786	1.057	1.377	1.534	0.964	2.343	1.513	2.128	1.529	1.103	1.660	4.785
F697_112.0504_10.4_P	0.300	0.223	0.183	0.325	0.382	0.392	0.090	0.380	0.109	0.187	0.119	0.152	0.204	0.124	0.218	0.145	0.201	0.195	0.051	0.075	0.069	0.167	0.133	0.447	0.129	0.437	0.178	0.252	0.236	0.292	0.172	0.220	0.080	0.167	0.155	0.412	0.213	0.200	0.378	0.395	0.566	0.520	0.495	0.328	0.478	0.441	1.132	1.252	0.822	1.063	0.836	0.858	0.656
F698_518.3237_4_P	0.744	0.884	1.054	0.858	0.771	0.561	0.752	0.657	0.898	0.461	0.483	0.792	0.619	0.549	0.676	0.400	0.596	0.648	0.675	0.859	0.423	0.635	0.714	0.534	0.485	0.585	0.510	0.770	0.599	0.505	0.446	0.315	0.345	0.397	0.489	0.488	0.387	0.244	0.764	0.660	0.725	1.018	0.568	0.657	0.723	0.503	0.460	0.578	0.606	0.479	0.360	0.392	0.397
F699_343.153_14.7_P	1.226	1.157	0.870	1.134	0.595	0.916	0.652	0.984	1.290	0.996	0.866	0.668	1.129	0.736	0.805	0.690	1.141	1.586	1.312	1.549	1.122	0.719	0.675	0.658	1.101	0.532	0.797	0.968	0.745	1.046	1.351	1.125	1.425	0.997	1.083	1.150	1.126	2.159	0.654	0.450	0.577	0.882	0.639	0.513	0.577	0.512	1.332	0.667	1.354	1.075	0.761	0.881	1.127
F700_524.3721_3.9_P	1.002	1.434	1.693	1.176	1.130	0.818	2.020	0.720	1.048	0.818	0.487	1.548	0.991	0.926	1.200	0.475	0.482	0.664	0.953	0.959	0.698	0.787	0.843	0.846	0.682	1.086	0.679	1.340	0.535	0.551	0.549	0.712	0.969	0.351	0.496	0.896	0.919	0.289	1.086	0.762	0.411	1.026	0.777	1.561	0.899	0.783	0.891	0.585	0.631	0.552	0.575	0.496	0.927
F701_296.1487_9.4_P	0.172	0.031	0.086	0.037	0.029	0.253	0.031	0.037	0.020	0.027	0.052	0.270	0.032	0.035	0.046	0.130	0.473	0.066	0.190	0.092	0.017	0.097	0.128	0.039	0.014	0.048	0.039	0.047	0.319	0.110	0.096	0.028	0.029	0.053	0.040	0.133	0.040	0.044	0.612	0.904	1.018	0.517	0.969	0.869	1.192	0.912	0.047	0.090	0.027	0.156	0.470	0.045	0.009
F702_787.6028_3.6_P	3.292	0.154	1.974	3.312	0.499	1.247	3.612	1.956	0.972	1.317	1.419	2.085	1.294	0.253	1.371	1.289	3.166	3.422	2.247	1.904	1.632	1.173	1.245	2.766	1.694	1.323	1.604	1.842	1.093	1.771	2.069	1.676	0.589	1.868	2.136	1.750	1.915	0.390	1.460	0.936	1.121	1.676	0.929	0.885	0.500	0.822	2.636	1.572	3.027	2.199	0.766	1.928	1.641
F704_854.5649_3.2_P	1.615	1.226	0.868	1.506	0.669	0.730	2.201	0.748	1.076	1.324	0.583	0.919	0.666	3.872	0.758	0.556	0.649	1.634	2.004	1.146	0.908	0.662	0.661	0.661	0.573	0.557	0.693	0.844	0.690	0.702	0.832	1.043	0.737	0.890	1.201	0.760	0.742	1.137	0.559	0.597	0.792	1.563	0.860	0.452	0.500	0.515	0.669	0.603	0.707	0.638	0.413	0.788	1.813
F705_518.3237_4_P	0.744	0.884	1.057	0.859	0.772	0.562	0.754	0.658	0.898	0.461	0.484	0.793	0.622	0.551	0.684	0.401	0.596	0.649	0.676	0.861	0.424	0.638	0.715	0.534	0.485	0.585	0.510	0.773	0.603	0.506	0.447	0.316	0.345	0.398	0.490	0.490	0.388	0.245	0.767	0.660	0.726	1.019	0.569	0.658	0.725	0.505	0.460	0.578	0.603	0.481	0.361	0.393	0.398
F706_196.0283_11.6_N	0.372	0.296	0.311	0.318	0.348	0.430	0.277	0.415	0.343	0.389	0.428	0.411	0.415	0.230	0.479	0.514	0.461	0.393	0.350	0.403	0.355	0.495	0.416	0.427	0.364	0.421	0.477	0.546	0.526	0.596	0.544	0.398	0.387	0.358	0.392	0.592	0.446	0.485	0.595	0.781	0.826	0.634	0.816	0.794	0.760	0.826	0.881	0.950	0.917	1.063	0.937	0.856	0.832
F707_226.0817_9.8_P	0.004	0.010	0.005	0.007	0.022	0.016	0.010	0.028	0.006	0.013	0.007	0.010	0.011	0.047	0.014	0.012	0.011	0.011	0.031	0.014	0.010	0.015	0.006	0.025	0.012	0.010	0.029	0.013	0.024	0.032	0.045	0.088	0.052	0.058	0.075	0.131	0.083	0.088	0.092	0.063	0.115	0.083	0.123	0.035	0.058	0.071	0.488	0.398	0.783	1.054	0.890	0.619	0.413
F708_301.238_3.4_N	0.443	0.628	0.989	0.820	1.094	1.007	0.419	0.989	0.841	0.573	0.951	0.815	1.044	0.231	1.030	0.823	0.655	0.375	0.309	0.700	0.844	0.881	0.686	0.981	0.850	0.990	0.781	0.617	0.688	0.699	0.738	0.521	0.576	0.479	0.370	0.649	0.437	0.595	0.732	0.696	0.447	0.354	0.915	0.802	0.688	0.808	0.492	0.568	0.443	0.678	0.570	0.537	0.386
F709_591.3897_3.1_N	0.341	0.648	0.378	0.732	0.798	0.792	0.816	0.786	0.490	0.428	0.387	0.541	0.738	0.733	0.672	0.673	0.412	0.470	0.231	0.565	0.544	0.374	0.455	0.840	0.446	0.596	0.455	0.496	0.476	0.638	0.730	0.600	0.540	0.323	0.320	0.415	0.797	0.425	0.453	0.767	0.407	0.231	0.435	0.871	0.195	0.355	0.331	0.400	0.333	0.368	0.314	0.299	0.428
F710_162.112_11.8_P	0.665	0.262	0.438	0.467	0.682	0.813	0.403	0.524	0.439	0.820	0.827	0.481	0.589	0.367	0.559	0.931	0.540	0.574	0.332	0.753	0.673	0.756	0.842	0.461	0.594	0.478	0.724	0.817	1.032	0.886	0.557	0.378	0.415	0.622	0.584	1.037	0.463	0.836	0.704	0.891	1.115	0.971	1.027	0.911	1.191	0.995	1.583	1.751	0.977	1.465	1.548	1.272	0.795
F711_349.2745_3.2_N	0.468	0.311	0.495	0.737	0.837	0.804	0.303	1.073	0.600	0.478	0.528	0.460	0.717	0.170	0.819	0.690	0.701	0.442	0.307	0.586	0.564	0.572	0.518	0.993	0.869	0.757	0.667	0.484	0.927	0.720	0.505	0.714	0.550	0.419	0.381	0.516	0.494	0.486	0.349	0.351	0.305	0.202	0.451	0.271	0.340	0.328	0.398	0.547	0.450	0.532	0.482	0.633	0.292
F712_176.1217_27.1_P	0.565	0.394	0.463	0.642	0.568	0.604	0.214	0.739	0.661	0.602	0.868	0.534	0.723	0.385	0.747	0.626	0.703	0.760	0.564	0.812	0.865	0.858	0.678	0.587	0.725	0.618	0.607	0.403	0.846	0.840	1.080	1.045	1.203	1.145	0.951	0.888	0.833	0.738	0.439	0.399	0.566	0.417	0.542	0.318	0.537	0.437	0.684	0.731	0.842	0.752	0.950	0.779	0.581
F713_275.2018_3.4_N	0.404	0.556	0.785	0.557	1.024	0.944	0.310	0.704	0.761	0.424	0.871	0.607	0.984	0.197	0.815	0.616	0.488	0.333	0.237	0.578	0.579	0.542	0.717	0.885	0.777	0.498	0.510	0.456	0.631	0.425	0.517	0.359	0.472	0.402	0.221	0.434	0.187	0.418	0.393	0.418	0.286	0.187	0.425	0.434	0.396	0.444	0.283	0.302	0.201	0.358	0.288	0.395	0.180
F714_293.2121_3.4_N	0.477	0.627	0.893	0.696	1.206	1.068	0.372	0.878	0.909	0.523	0.993	0.728	1.160	0.224	0.973	0.750	0.598	0.435	0.285	0.662	0.716	0.654	0.817	1.108	0.966	0.646	0.671	0.565	0.761	0.545	0.579	0.473	0.549	0.490	0.277	0.528	0.298	0.498	0.520	0.529	0.361	0.237	0.546	0.527	0.486	0.533	0.342	0.387	0.252	0.427	0.342	0.467	0.233
F715_277.2172_3.4_N	0.745	1.058	1.604	1.162	1.914	1.817	0.657	1.575	1.612	0.961	1.934	1.360	2.034	0.417	1.697	1.324	1.019	0.708	0.571	1.436	1.332	1.621	1.487	1.914	1.647	1.301	1.175	0.968	1.389	1.149	1.147	0.810	1.044	0.933	0.580	1.062	0.584	0.968	1.035	1.020	0.692	0.474	1.116	1.087	1.020	1.315	0.678	0.697	0.628	0.975	0.803	0.971	0.474
F716_141.0655_10.9_P	0.259	0.130	0.142	0.103	0.205	0.322	0.445	0.125	0.294	0.382	0.261	0.647	0.559	0.229	0.225	0.219	0.241	0.265	0.448	0.456	0.065	0.216	0.286	0.389	0.250	0.198	1.401	0.313	0.452	0.229	0.165	0.239	0.248	0.343	0.369	1.010	0.656	0.354	0.390	0.370	0.420	0.327	0.207	0.328	0.418	0.260	0.217	0.442	0.579	0.399	0.209	0.090	0.484
F717_289.1387_13.7_P	0.572	0.531	0.655	0.547	0.660	0.615	0.486	0.705	0.614	0.556	0.742	0.784	0.730	0.352	0.913	0.926	0.981	0.538	0.593	0.763	0.882	0.947	0.842	0.793	0.845	0.759	0.768	0.841	0.822	0.726	1.098	0.736	0.958	0.675	0.892	0.915	0.879	0.785	0.588	0.715	0.838	0.715	1.029	0.800	0.879	1.070	0.776	0.839	0.891	0.895	0.933	1.032	0.488
F718_776.6138_3.6_P	4.227	0.041	0.126	0.184	1.839	1.953	7.638	1.919	2.548	4.104	1.922	1.633	1.434	0.101	2.140	1.647	3.078	0.212	4.449	1.786	2.083	1.736	1.955	1.326	2.048	1.612	2.038	2.786	1.498	2.047	2.945	0.131	2.663	2.293	3.713	1.718	4.154	4.457	2.129	1.369	1.724	3.344	1.214	1.672	1.988	1.814	2.312	3.198	2.308	3.197	1.654	1.725	5.485
F719_212.1387_9.8_P	0.396	0.122	0.215	0.104	0.282	0.408	0.122	0.425	0.131	0.272	0.379	0.152	0.465	0.223	0.348	0.499	0.219	0.179	0.177	0.342	0.295	0.275	0.404	0.349	0.337	0.419	0.749	0.749	0.634	0.701	0.319	0.352	0.163	0.308	0.359	0.438	0.246	0.351	0.339	0.298	0.368	0.441	0.421	0.295	0.683	0.689	0.927	1.114	0.865	0.843	1.683	0.555	0.615
F720_537.1676_11.1_P	0.980	0.640	1.019	1.044	1.630	1.239	0.478	1.310	1.020	0.790	1.029	1.145	1.208	0.106	1.045	1.088	1.359	0.895	0.808	1.354	1.189	0.936	0.978	1.315	1.146	1.202	1.759	1.302	1.687	1.613	1.264	1.092	1.436	1.197	1.114	1.328	1.468	1.566	2.220	2.071	1.906	1.944	2.824	1.538	2.521	2.508	1.727	1.768	1.873	2.124	1.940	2.045	1.362
F721_147.0761_7.5_P	0.465	0.460	0.727	0.565	0.639	0.726	0.402	0.567	0.582	0.367	0.538	0.700	0.611	0.246	0.563	0.514	0.512	0.562	0.523	0.561	0.326	0.404	0.411	0.623	0.415	0.555	0.710	0.428	0.733	0.441	0.429	0.639	0.388	0.464	0.407	0.628	0.674	0.350	0.900	0.914	1.031	0.899	0.964	0.984	0.724	0.706	0.398	0.338	0.375	0.503	0.496	0.311	0.287
F722_468.308_4.2_P	1.397	1.224	1.239	1.295	1.105	0.787	1.123	1.080	1.310	0.882	0.883	1.010	0.878	0.637	0.946	0.738	1.067	1.080	1.134	1.350	1.021	0.980	1.155	1.015	1.011	1.061	0.745	1.037	0.935	0.835	0.958	0.710	0.817	0.784	0.899	0.781	0.631	0.766	0.967	0.719	0.855	1.307	1.006	0.627	0.859	0.711	1.039	0.974	0.989	0.890	0.622	0.831	0.690
F723_544.34_3.9_P	1.301	1.675	1.646	1.467	1.044	0.750	1.716	0.780	1.394	0.986	0.612	1.255	0.830	0.799	0.897	0.649	0.873	1.231	1.001	1.060	0.756	0.843	0.901	0.825	0.946	1.081	0.698	1.274	0.522	0.640	0.863	0.717	0.849	0.592	0.496	0.667	0.557	0.609	1.096	0.956	0.783	1.237	0.901	0.983	0.887	0.647	0.798	0.742	0.902	0.895	0.550	0.594	0.761

F724_494.3238_4.1_P	2.097	2.085	2.123	1.741	1.775	1.248	1.959	1.490	1.442	1.414	1.215	1.608	1.400	0.957	1.164	0.976	1.358	1.694	1.785	1.903	1.178	1.087	1.538	1.409	1.453	1.406	1.120	1.545	1.344	1.091	1.061	0.928	1.073	0.966	0.888	0.902	0.914	0.973	1.308	1.182	1.220	1.877	1.495	1.240	1.382	1.132	1.286	1.331	1.430	0.881	0.831	1.087	1.109
F725_809.5831_3.4_P	4.183	8.430	3.366	5.002	2.466	1.840	8.026	1.636	2.885	4.681	2.390	2.270	2.316	11.75	3.343	2.268	2.541	4.709	4.391	4.038	2.173	3.034	2.816	2.926	2.463	2.763	2.271	3.281	1.385	3.587	4.265	3.783	5.199	4.365	3.732	2.600	4.113	5.197	2.134	1.877	1.757	4.001	2.283	1.765	1.855	2.017	2.165	2.733	3.960	2.415	1.963	1.674	4.754
F726_295.2271_3.4_N	0.770	1.087	1.635	1.197	1.970	1.858	0.684	1.618	1.660	0.983	1.983	1.399	2.077	0.516	1.744	1.362	1.053	0.734	0.587	1.468	1.367	1.655	1.516	1.960	1.697	1.343	1.216	1.002	1.430	1.178	1.183	0.838	1.068	0.955	0.598	1.095	0.608	0.997	1.091	1.063	0.727	0.505	1.178	1.140	1.066	1.385	0.698	0.724	0.649	1.007	0.829	1.004	0.497
F727_548.4343_3.2_P	1.969	3.714	1.574	3.388	0.528	0.770	3.235	0.896	1.700	2.482	1.063	1.055	1.130	8.393	1.138	0.711	1.628	2.775	3.399	1.627	1.195	0.809	1.398	1.210	1.152	0.957	1.002	1.247	0.959	1.453	2.120	1.612	1.694	2.257	1.876	1.478	1.467	2.421	0.928	0.885	1.037	1.719	0.898	0.773	1.257	0.908	1.742	1.378	1.481	1.571	1.033	0.964	3.480
F728_602.3094_3.9_N	1.067	0.856	0.863	0.880	0.794	0.646	0.890	0.821	1.089	0.663	0.647	0.960	0.824	0.686	0.771	0.632	0.733	0.859	0.894	0.875	0.804	0.971	0.934	0.728	0.824	0.992	0.949	0.982	0.877	0.724	0.796	0.533	0.688	0.563	0.683	0.631	0.533	0.465	0.838	0.910	0.843	1.176	0.906	0.887	0.756	0.762	0.527	0.500	0.647	0.594	0.572	0.500	0.428
F729_86.0962_11.2_P	0.615	0.478	0.559	0.715	0.418	0.503	0.540	0.530	0.579	0.482	0.669	0.439	0.633	0.458	0.639	0.628	0.763	0.758	0.824	0.751	0.964	0.800	0.683	0.329	0.518	0.524	0.449	0.417	0.568	0.593	1.010	0.784	1.099	0.856	1.059	0.671	0.802	0.905	1.220	1.089	1.201	0.880	0.905	1.348	1.055	1.170	0.656	0.513	0.828	0.921	1.229	0.696	0.794
F730_285.1439_9.8_P	0.457	0.267	0.653	0.484	0.683	0.575	0.114	0.906	0.642	0.492	0.730	0.826	0.847	0.229	0.927	0.921	0.956	0.532	0.563	0.809	1.210	1.119	0.845	1.086	0.949	0.986	1.076	0.925	0.913	0.963	1.217	0.967	1.186	0.780	1.097	1.107	1.127	1.086	0.709	0.632	0.866	0.719	0.955	0.766	0.954	1.198	0.932	1.100	1.004	1.017	1.407	1.400	0.553
F731_867.5585_3.4_N	1.880	3.579	1.429	2.488	1.213	1.587	1.587	1.829	1.793	2.061	1.436	1.228	1.768	9.446	1.869	1.314	1.992	2.337	2.436	2.061	1.219	1.438	1.503	1.747	0.312	1.325	1.760	1.776	1.199	1.842	2.241	1.873	1.797	1.701	1.755	1.534	2.028	1.085	1.366	1.532	1.057	1.668	1.073	0.988	1.271	0.788	1.604	1.599	1.775	1.506	1.211	1.101	3.044
F732_228.0975_10.4_P	0.336	0.232	0.202	0.325	0.456	0.462	0.124	0.424	0.122	0.216	0.119	0.143	0.226	0.275	0.248	0.146	0.220	0.207	0.049	0.071	0.068	0.167	0.155	0.504	0.138	0.492	0.173	0.275	0.267	0.316	0.174	0.236	0.074	0.157	0.136	0.514	0.270	0.206	0.421	0.479	0.647	0.548	0.547	0.395	0.552	0.532	1.327	1.572	0.887	1.251	1.026	0.989	0.697
F733_146.0813_13.3_P	0.573	0.609	0.732	0.655	0.851	1.065	0.448	1.103	0.822	0.598	0.786	0.894	1.116	0.536	0.927	1.151	1.314	0.923	0.705	0.787	0.783	0.799	1.070	0.923	0.784	0.685	0.948	0.857	1.232	0.988	0.957	1.113	0.668	0.941	0.842	1.023	1.309	1.159	1.029	0.833	1.110	0.758	1.103	0.829	1.423	1.187	1.136	0.945	1.259	1.409	1.153	1.010	0.710
F734_76.0392_16.2_P	0.690	0.371	0.619	0.569	1.000	1.024	0.351	1.111	0.806	0.703	1.176	0.712	0.891	0.318	0.681	0.894	0.833	0.619	0.690	0.808	0.990	1.107	1.004	1.109	0.965	0.741	1.162	0.887	1.204	0.881	0.926	0.973	1.200	1.131	1.029	0.973	0.771	1.063	0.778	0.664	0.784	0.612	0.940	0.586	0.829	1.009	1.157	1.171	1.146	1.288	1.326	1.556	0.630
F735_526.2918_3.8_P	1.027	1.239	1.198	1.061	0.945	0.653	0.850	0.749	0.942	0.714	0.496	0.824	0.736	0.704	0.721	0.467	0.644	0.899	0.984	1.154	0.634	0.760	0.834	0.657	0.808	0.672	0.533	0.772	0.364	0.592	0.519	0.485	0.597	0.604	0.528	0.579	0.515	0.506	1.378	1.051	1.084	1.997	1.673	1.297	1.400	1.145	0.373	0.498	0.549	0.446	0.284	0.433	0.489
F736_179.056_13.1_N	0.664	0.403	0.762	1.191	0.584	1.033	0.450	1.503	0.803	0.482	1.058	0.647	0.966	0.218	0.446	1.263	1.012	1.519	1.194	0.660	0.954	1.090	1.112	0.655	0.440	1.027	1.069	1.064	1.179	0.885	0.995	0.770	0.787	0.973	0.721	1.219	0.976	1.327	0.766	0.472	1.218	1.108	0.976	0.880	1.204	0.995	1.208	1.045	0.256	1.637	0.823	0.610	1.147
F737_325.2751_3.3_N	0.470	0.679	1.031	0.703	1.056	0.957	0.457	0.854	0.969	0.529	1.063	0.758	1.063	0.310	0.990	0.886	0.714	0.425	0.413	0.763	0.888	1.033	0.752	1.401	0.862	0.943	0.767	0.642	0.943	0.698	0.754	0.508	0.708	0.522	0.325	0.642	0.383	0.533	0.349	0.314	0.188	0.165	0.376	0.345	0.207	0.405	0.400	0.290	0.379	0.546	0.492	0.460	0.309
F738_171.0761_15.4_P	1.260	0.774	1.126	1.148	0.969	0.979	0.749	1.121	1.484	1.184	1.206	1.174	1.397	0.924	1.173	1.127	1.216	1.367	1.234	1.308	1.411	1.313	1.376	0.995	1.362	0.999	0.866	1.279	1.198	1.120	1.518	1.524	1.620	1.533	1.423	1.234	1.305	1.427	0.858	0.862	0.894	0.868	0.871	0.599	0.860	0.838	0.882	0.774	1.077	0.998	1.083	0.917	0.740
F739_805.5902_3.4_P	3.133	6.829	1.510	3.628	1.396	1.354	5.556	1.005	2.639	3.875	1.367	2.058	1.775	8.209	2.546	1.286	2.222	3.520	4.352	3.597	1.660	1.972	2.575	2.766	2.263	2.069	1.812	3.147	1.053	3.266	2.739	1.776	2.571	2.681	2.045	2.475	3.057	3.825	2.199	1.757	1.393	1.537	1.700	1.416	1.286	1.423	1.630	0.900	3.077	1.633	1.418	1.154	3.302
F740_531.4069_3.2_P	1.959	3.708	1.566	3.369	0.527	0.767	3.224	0.891	1.691	2.466	1.058	1.049	1.124	8.365	1.131	0.703	1.620	2.761	3.386	1.619	1.188	0.803	1.388	1.204	1.145	0.951	0.995	1.242	0.953	1.443	2.106	1.603	1.684	2.242	1.862	1.470	1.453	2.401	0.924	0.881	1.030	1.709	0.893	0.769	1.248	0.902	1.733	1.370	1.475	1.562	1.027	0.960	3.467
F741_122.081_15.8_P	0.003	0.010	0.006	0.013	0.002	0.002	0.030	0.002	2.231	0.003	0.002	0.002	0.002	0.158	0.003	0.002	0.010	0.004	0.004	0.004	0.002	0.002	0.002	0.002	0.006	0.010	0.002	0.002	0.002	0.002	0.003	0.002	0.008	0.002	0.002	0.003	0.002	0.005	0.002	0.002	0.002	0.003	0.002	0.002	0.002	0.002	0.003	0.003	0.006	0.002	0.003	0.003	0.021
F742_176.1158_27.1_P	0.127	0.079	0.129	0.143	0.147	0.136	0.041	0.159	0.158	0.130	0.172	0.118	0.192	0.072	0.140	0.147	0.133	0.164	0.128	0.152	0.192	0.201	0.160	0.139	0.167	0.123	0.134	0.091	0.175	0.186	0.241	0.228	0.261	0.254	0.262	0.199	0.190	0.168	0.099	0.090	0.124	0.086	0.118	0.071	0.123	0.103	0.135	0.138	0.176	0.172	0.224	0.174	0.124
F743_225.186_3.5_N	0.115	0.108	0.187	0.132	0.258	0.196	0.073	0.235	0.223	0.134	0.180	0.200	0.231	0.082	0.224	0.199	0.182	0.112	0.078	0.167	0.195	0.183	0.163	0.247	0.219	0.201	0.222	0.168	0.199	0.220	0.178	0.163	0.168	0.129	0.114	0.180	0.153	0.165	0.185	0.162	0.147	0.110	0.245	0.217	0.154	0.217	0.203	0.204	0.226	0.188	0.215	0.299	0.100
F744_131.053_15.4_P	0.459	0.248	0.451	0.435	0.436	0.382	0.248	0.472	0.574	0.452	0.482	0.465	0.562	0.282	0.460	0.437	0.484	0.528	0.454	0.496	0.578	0.556	0.537	0.389	0.568	0.408	0.447	0.507	0.491	0.454	0.595	0.597	0.661	0.584	0.525	0.478	0.490	0.516	0.331	0.334	0.369	0.319	0.354	0.219	0.328	0.338	0.342	0.302	0.413	0.338	0.428	0.368	0.245
F745_117.0674_12.9_P	0.242	0.057	0.175	0.085	0.049	0.058	0.166	0.115	0.141	0.098	0.211	0.049	0.062	0.046	0.052	0.063	0.214	0.187	0.292	0.139	0.277	0.229	0.244	0.074	0.114	0.104	0.096	0.101	0.112	0.105	0.182	0.063	0.143	0.099	0.112	0.162	0.059	0.107	0.108	0.161	0.125	0.147	0.160	0.167	0.143	0.207	0.092	0.080	0.081	0.083	0.105	0.061	0.068
F746_266.1382_7.8_P	0.070	0.067	0.086	0.055	0.116	0.114	0.079	0.089	0.104	0.066	0.047	0.109	0.115	0.023	0.114	0.066	0.093	0.065	0.086	0.109	0.074	0.134	0.099	0.096	0.106	0.075	0.111	0.084	0.070	0.071	0.077	0.069	0.075	0.086	0.093	0.066	0.090	0.052	0.083	0.085	0.053	0.049	0.066	0.083	0.116	0.056	0.054	0.092	0.111	0.063	0.108	0.075	0.047
F747_240.1088_10.4_P	0.133	0.102	0.121	0.125	0.070	0.077	0.117	0.073	0.126	0.141	0.065	0.089	0.049	0.036	0.063	0.071	0.085	0.098	0.123	0.094	0.055	0.046	0.061	0.067	0.105	0.071	0.058	0.113	0.045	0.092	0.085	0.079	0.065	0.091	0.124	0.101	0.100	0.153	0.089	0.061	0.076	0.262	0.089	0.078	0.112	0.060	0.155	0.100	0.202	0.150	0.081	0.092	0.436
F748_317.2121_3.4_N	0.063	0.071	0.079	0.080	0.122	0.093	0.092	0.101	0.084	0.079	0.097	0.088	0.068	0.067	0.103	0.087	0.065	0.067	0.052	0.062	0.087	0.084	0.039	0.106	0.092	0.089	0.086	0.073	0.094	0.091	0.062	0.060	0.075	0.067	0.047	0.073	0.057	0.083	0.070	0.066	0.062	0.062	0.105	0.091	0.066	0.105	0.048	0.066	0.048	0.062	0.051	0.071	0.059
F749_744.5529_3.5_P	0.309	0.188	0.148	0.213	0.101	0.086	0.499	0.120	0.269	0.300	0.113	0.148	0.094	0.566	0.072	0.087	0.062	0.442	0.319	0.115	0.132	0.105	0.126	0.128	0.131	0.104	0.071	0.162	0.053	0.134	0.119	0.077	0.118	0.108	0.130	0.107	0.101	0.230	0.008	0.048	0.088	0.176	0.074	0.042	0.043	0.069	0.137	0.141	0.152	0.085	0.052	0.164	0.405
F750_229.118_15.1_P	0.208	0.023	0.174	0.099	0.104	0.174	0.030	0.209	0.184	0.070	0.181	0.132	0.136	0.099	0.205	0.164	0.216	0.120	0.139	0.193	0.229	0.151	0.163	0.183	0.190	0.095	0.193	0.158	0.295	0.245	0.306	0.258	0.240	0.200	0.172	0.337	0.199	0.336	0.188	0.163	0.250	0.167	0.271	0.209	0.222	0.215	0.232	0.088	0.230	0.259	0.221	0.192	0.052
F751_118.061_16.3_P	0.018	0.007	0.009	0.014	0.030	0.027	0.004	0.020	0.023	0.020	0.031	0.018	0.026	0.020	0.030	0.030	0.024	0.011	0.017	0.025	0.008	0.030	0.019	0.019	0.015	0.020	0.014	0.011	0.030	0.031	0.014	0.017	0.013	0.019	0.028	0.018	0.022	0.011	0.075	0.053	0.120	0.039	0.108	0.014	0.113	0.056	0.027	0.037	0.026	0.032	0.043	0.036	0.008
F752_343.2948_4.3_P	0.040	0.081	0.025	0.087	0.026	0.006	0.064	0.005	0.026	0.032	0.025	0.016	0.048	0.033	0.005	0.020	0.100	0.017	0.006	0.029	0.037	0.028	0.014	0.083	0.012	0.005	0.013	0.025	0.004	0.029	0.028	0.032	0.073	0.031	0.020	0.148	0.009	0.111	0.040	0.004	0.027	0.042	0.006	0.012	0.047	0.013	0.147	0.010	0.049	0.027	0.024	0.017	0.073
F753_117.1018_10.7_P	0.129	0.137	0.116	0.129	0.147	0.200	0.150	0.154	0.142	0.170	0.108	0.181	0.184	0.135	0.156	0.146	0.137	0.132	0.157	0.108	0.104	0.137	0.134	0.154	0.158	0.136	0.117	0.142	0.181	0.144	0.132	0.114	0.121	0.175	0.090	0.121	0.186	0.121	0.213	0.194	0.155	0.111	0.143	0.112	0.147	0.157	0.074	0.103	0.077	0.087	0.096	0.092	0.113
F754_498.262_3.9_N	0.053	0.140	0.197	0.089	0.108	0.070	0.073	0.107	0.084	0.090	0.054	0.131	0.104	0.068	0.105	0.030	0.083	0.129	0.120	0.141	0.067	0.130	0.109	0.065	0.086	0.089	0.063	0.072	0.057	0.062	0.060	0.061	0.062	0.073	0.074	0.099	0.051	0.076	0.154	0.140	0.128	0.205	0.151	0.138	0.213	0.144	0.037	0.063	0.069	0.038	0.013	0.062	0.034
F755_177.0615_16.2_P	0.030	0.010	0.004	0.004	0.002	0.003	0.071	0.003	0.003	0.003	0.002	0.003	0.003	0.047	0.002	0.050	0.002	0.004	0.005	0.004	0.003	0.003	0.003	0.006	0.002	0.003	0.003	0.191	0.002	0.003	0.002	0.003	0.003	0.003	0.003	0.003	0.003	0.004	0.003	0.007	0.006	0.004	0.002	0.004	0.003	0.012	0.002	0.002	0.003	0.003	0.005	0.003	0.008
F757_144.0765_11.8_P	0.055	0.036	0.086	0.077	0.073	0.145	0.039	0.100	0.054	0.030	0.054	0.100	0.090	0.043	0.082	0.107	0.112	0.075	0.040	0.055	0.064	0.073	0.088	0.079	0.060	0.047	0.071	0.042	0.091	0.082	0.057	0.095	0.049	0.069	0.078	0.109	0.115	0.061	0.077	0.070	0.084	0.058	0.075	0.082	0.060	0.045	0.044	0.052	0.034	0.110	0.123	0.038	0.028
F758_118.0648_10_P	0.029	0.019	0.057	0.030	0.068	0.065	0.035	0.062	0.052	0.033	0.065	0.049	0.065	0.138	0.068	0.069	0.061	0.038	0.029	0.050	0.067	0.061	0.048	0.046	0.052	0.052	0.059	0.048	0.061	0.056	0.060	0.067	0.071	0.057	0.065	0.050	0.069	0.048	0.080	0.095	0.112	0.053	0.077	0.076	0.092	0.099	0.047	0.047	0.054	0.059	0.087	0.058	0.026
F760_137.0455_8_P	0.013	0.016	0.009	0.010	0.019	0.017	0.008	0.016	0.012	0.009	0.011	0.010	0.013	0.045	0.016	0.012	0.009	0.013	0.005	0.013	0.009	0.013	0.012	0.013	0.008	0.014	0.013	0.012	0.015	0.018	0.015	0.017	0.012	0.017	0.014	0.016	0.019	0.014	0.010	0.013	0.009	0.010	0.016	0.009	0.016	0.010	0.052	0.092	0.026	0.030	0.118	0.033	0.022
F761_87.0439_15.7_P	0.078	0.061	0.054	0.039	0.068	0.084	0.054	0.062	0.063	0.066	0.086	0.089	0.075	0.031	0.069	0.080	0.053	0.038	0.062	0.056	0.048	0.112	0.054	0.068	0.088	0.082	0.149	0.173	0.138	0.100	0.066	0.081	0.095	0.057	0.083	0.147	0.087	0.075	0.082	0.168	0.144	0.135	0.121	0.142	0.133	0.145	0.217	0.209	0.225	0.210	0.190	0.153	0.155
F762_178.0719_11.1_P	0.088	0.070	0.065	0.083	0.083	0.066	0.075	0.070	0.078	0.053	0.069	0.071	0.073	0.050	0.068	0.061	0.080	0.057	0.081	0.099	0.065	0.074	0.073	0.074	0.068	0.076	0.082	0.069	0.082	0.123	0.079	0.060	0.098	0.067	0.071	0.087	0.098	0.144	0.110	0.106	0.109	0.151	0.136	0.086	0.158	0.118	0.113	0.104	0.131	0.128	0.114	0.107	0.137
F763_100.0755_15.6_P	0.042	0.027	0.035	0.024	0.053	0.067	0.039	0.041	0.037	0.040	0.061	0.044	0.038	0.020	0.038	0.043	0.034	0.028	0.032	0.033	0.027	0.052	0.033	0.044	0.053	0.052	0.066	0.072	0.065	0.069	0.031	0.037	0.044	0.042	0.036	0.063	0.040	0.033	0.067	0.089	0.084	0.087	0.078	0.086	0.073	0.081	0.092	0.086	0.085	0.098	0.084	0.073	0.069
F764_188.1024_12.8_P	0.045	0.027	0.033	0.033	0.039	0.076	0.039	0.036	0.034	0.044	0.035	0.092	0.055	0.042	0.048	0.056	0.040	0.043	0.040	0.037	0.010	0.030	0.042	0.046	0.045	0.030	0.053	0.039	0.090	0.061	0.045	0.062	0.044	0.090	0.089	0.090	0.090	0.053	0.090	0.069	0.111	0.068	0.058	0.056	0.104	0.061	0.064	0.060	0.066	0.115	0.120	0.064	0.061
F765_119.0349_11.4_N	0.053	0.053	0.071	0.057	0.075	0.109	0.033	0.084	0.075	0.044	0.078	0.083	0.082	0.011	0.084	0.076	0.081	0.070	0.047	0.049	0.048	0.068	0.059	0.084	0.054	0.064	0.106	0.057	0.108	0.070	0.063	0.080	0.052	0.061	0.059	0.087	0.090	0.052	0.078	0.063	0.065	0.055	0.073	0.082	0.051	0.064	0.049	0.036	0.048	0.072	0.065	0.043	0.027
F766_175.1074_11.6_P	0.049	0.035	0.053	0.043	0.056	0.075	0.030	0.060	0.037	0.048	0.047	0.056	0.052	0.043	0.057	0.060	0.050	0.044	0.042	0.048	0.036	0.056	0.055	0.049	0.045	0.038	0.067	0.060	0.095	0.068	0.055	0.054	0.044	0.053	0.058	0.095	0.087	0.062	0.071	0.088	0.084	0.071	0.097	0.077	0.069	0.087	0.076	0.090	0.061	0.092	0.115	0.067	0.044
F767_343.2272_3.4_N	0.061	0.062	0.067	0.072	0.089	0.068	0.066	0.084	0.064	0.057	0.058	0.038	0.069	0.062	0.070	0.057	0.043	0.043	0.036	0.049	0.051	0.057	0.046	0.081	0.068	0.074	0.068	0.056	0.067	0.054	0.049	0.044	0.050	0.050	0.034	0.049	0.048	0.045	0.066	0.052	0.079	0.062	0.086	0.071	0.061	0.089	0.033	0.057	0.037	0.055	0.041	0.051	0.040
F768_124.0391_7.9_P	0.008	0.015	0.010	0.008	0.008	0.008	0.009	0.004	0.003	0.003	0.004	0.047	0.004	0.050	0.006	0.011	0.010	0.011	0.004	0.006	0.005	0.004	0.004	0.006	0.005	0.006	0.009	0.009	0.010	0.006	0.006	0.005	0.005	0.005	0.005	0.005	0.008	0.007	0.047	0.088	0.116	0.030	0.015	0.060	0.068	0.036	0.009	0.006	0.011	0.016	0.016	0.007	0.015
F769_792.5394_3.4_N	0.113	0.331	0.070	0.205	0.086	0.075	0.294	0.089	0.127	0.168	0.103	0.117	0.130	0.465	0.048	0.089	0.058	0.181	0.118	0.126	0.109	0.037	0.079	0.119	0.117	0.150	0.079	0.094	0.075	0.079	0.131	0.048	0.082	0.116	0.090	0.051	0.076	0.107	0.102	0.069	0.099	0.125	0.070	0.108	0.053	0.084	0.127	0.107	0.141	0.122	0.081	0.105	0.330
F770_258.108_9_P	0.017	0.007	0.018	0.017	0.031	0.036	0.006	0.026	0.018	0.017	0.021	0.022	0.020	0.061	0.027	0.023	0.019	0.019	0.009	0.013	0.013	0.025	0.021	0.030	0.019	0.020	0.031	0.024	0.034	0.030	0.023	0.027	0.020	0.027	0.027	0.031	0.030	0.022	0.030	0.034	0.023	0.017	0.035	0.031	0.029	0.032	0.071	0.068	0.070	0.074	0.117	0.082	0.034
F771_290.1422_13.7_P	0.062	0.052	0.075	0.064	0.079	0.075	0.053	0.086	0.073	0.067	0.085	0.093	0.089	0.041	0.109	0.116	0.116	0.064	0.066	0.088	0.113	0.117	0.100	0.092	0.096	0.089	0.095	0.100	0.103	0.100	0.133	0.087	0.115	0.079	0.111	0.114	0.110	0.104	0.069	0.086	0.101	0.080	0.129	0.096	0.105	0.127	0.091	0.098	0.104	0.110	0.118	0.125	0.048
F772_215.1388_12.4_P	0.112	0.035	0.107	0.076	0.082	0.154	0.071	0.087	0.067	0.095	0.082	0.142	0.089	0.045	0.104	0.167	0.163	0.086	0.096	0.058	0.072	0.109	0.141	0.072	0.095	0.064	0.107	0.087	0.128	0.145	0.091	0.122	0.093	0.165	0.180	0.168	0.156	0.116	0.145	0.110	0.126	0.118	0.094	0.068	0.082	0.066	0.172	0.144	0.143	0.166	0.150	0.144	0.163
F773_242.1132_9.2_P	0.015	0.011	0.019	0.033	0.044	0.051	0.006	0.048	0.014	0.016	0.016	0.016	0.027	0.028	0.025	0.018	0.024	0.018	0.005	0.014	0.018	0.022	0.023	0.042	0.018	0.049	0.023	0.023	0.040	0.032	0.025	0.026	0.019	0.020	0.021	0.051	0.030	0.024	0.037	0.040	0.052	0.035	0.062	0.037	0.042	0.047	0.114	0.161	0.065	0.091	0.075	0.097	0.045
F774_182.092_11.7_P	0.067	0.027	0.058	0.046	0.042	0.111	0.047	0.056	0.029	0.021	0.027	0.080	0.033	0.050	0.040	0.050	0.041	0.069	0.031	0.045	0.036	0.064	0.062	0.059	0.033	0.045	0.044	0.034	0.083	0.064	0.030	0.056	0.054	0.052	0.054	0.074	0.051	0.037	0.067	0.103	0.084	0.065	0.109	0.114	0.098	0.086	0.028	0.061	0.032	0.065	0.153	0.044	0.022
F776_144.0301_11.3_N	0.065	0.049	0.065	0.058	0.058	0.090	0.064	0.064	0.065	0.047	0.064	0.085	0.067	0.075	0.075	0.092	0.079	0.072	0.070	0.055	0.041	0.079	0.094	0.064	0.053	0.058	0.095	0.060	0.129	0.056	0.072	0.078	0.051	0.071	0.068	0.081	0.080	0.050	0.072	0.073	0.070	0.060	0.062	0.105	0.051	0.080	0.060	0.042	0.059	0.078	0.110	0.051	0.059

F777_190.1435_8_P	0.051	0.047	0.070	0.061	0.098	0.120	0.016	0.101	0.083	0.044	0.077	0.067	0.113	0.033	0.107	0.102	0.089	0.066	0.044	0.060	0.062	0.066	0.044	0.126	0.076	0.085	0.137	0.058	0.085	0.093	0.077	0.146	0.074	0.077	0.059	0.081	0.173	0.047	0.089	0.091	0.085	0.048	0.098	0.085	0.048	0.066	0.062	0.058	0.066	0.115	0.108	0.054	0.037
F778_168.1491_9.8_P	0.031	0.006	0.017	0.006	0.022	0.028	0.005	0.026	0.009	0.019	0.025	0.010	0.031	0.025	0.027	0.039	0.019	0.012	0.012	0.025	0.021	0.020	0.027	0.025	0.026	0.031	0.059	0.059	0.049	0.055	0.030	0.021	0.014	0.020	0.028	0.032	0.017	0.023	0.028	0.024	0.029	0.032	0.032	0.020	0.049	0.061	0.090	0.093	0.076	0.059	0.123	0.049	0.045
F779_466.3285_4.2_P	0.242	0.129	0.157	0.153	0.164	0.116	0.036	0.130	0.114	0.077	0.073	0.067	0.083	0.074	0.084	0.050	0.089	0.100	0.065	0.106	0.098	0.067	0.051	0.117	0.099	0.061	0.085	0.121	0.100	0.096	0.061	0.076	0.077	0.079	0.057	0.088	0.088	0.078	0.078	0.066	0.071	0.070	0.095	0.078	0.082	0.059	0.090	0.173	0.127	0.102	0.049	0.106	0.085
F780_284.0984_16.3_N	0.072	0.029	0.068	0.053	0.082	0.080	0.023	0.106	0.092	0.071	0.143	0.044	0.092	0.023	0.074	0.088	0.093	0.101	0.063	0.095	0.133	0.150	0.151	0.093	0.170	0.067	0.081	0.074	0.151	0.090	0.150	0.139	0.184	0.199	0.114	0.080	0.075	0.064	0.048	0.042	0.066	0.054	0.091	0.029	0.066	0.069	0.040	0.046	0.054	0.054	0.063	0.076	0.016
F781_204.0862_8.1_P	0.044	0.042	0.076	0.062	0.090	0.118	0.042	0.103	0.075	0.041	0.057	0.085	0.107	0.047	0.091	0.089	0.078	0.070	0.048	0.067	0.049	0.060	0.070	0.090	0.058	0.084	0.103	0.058	0.117	0.080	0.069	0.100	0.045	0.072	0.060	0.103	0.129	0.059	0.120	0.110	0.109	0.080	0.120	0.122	0.101	0.120	0.057	0.049	0.049	0.068	0.079	0.048	0.029
F782_766.5353_3.3_P	0.395	0.520	0.108	0.273	0.060	0.052	0.463	0.110	0.173	0.281	0.092	0.163	0.193	1.035	0.135	0.096	0.166	0.380	0.492	0.320	0.162	0.178	0.163	0.126	0.134	0.121	0.058	0.110	0.134	0.181	0.198	0.193	0.101	0.280	0.192	0.057	0.112	0.175	0.096	0.072	0.174	0.380	0.097	0.093	0.106	0.056	0.159	0.095	0.166	0.117	0.184	0.132	0.330
F783_131.0813_15.6_P	0.082	0.038	0.060	0.053	0.062	0.055	0.046	0.045	0.051	0.081	0.092	0.040	0.055	0.119	0.054	0.088	0.075	0.046	0.111	0.062	0.057	0.065	0.067	0.060	0.044	0.086	0.049	0.043	0.082	0.073	0.060	0.053	0.081	0.081	0.100	0.075	0.050	0.093	0.030	0.024	0.080	0.080	0.074	0.021	0.059	0.048	0.120	0.106	0.120	0.108	0.130	0.154	0.087
F784_297.152_9.4_P	0.018	0.006	0.009	0.003	0.002	0.032	0.007	0.002	0.002	0.003	0.004	0.034	0.001	0.033	0.004	0.017	0.066	0.005	0.016	0.012	0.001	0.010	0.015	0.003	0.001	0.004	0.003	0.004	0.041	0.012	0.011	0.001	0.001	0.003	0.003	0.016	0.002	0.006	0.086	0.131	0.141	0.069	0.139	0.126	0.172	0.147	0.003	0.009	0.002	0.019	0.068	0.003	0.005
F785_201.123_13.3_P	0.013	0.008	0.009	0.003	0.009	0.021	0.008	0.014	0.008	0.003	0.005	0.008	0.007	0.034	0.006	0.008	0.012	0.015	0.004	0.013	0.009	0.014	0.011	0.009	0.009	0.008	0.009	0.008	0.012	0.015	0.008	0.009	0.011	0.012	0.008	0.012	0.010	0.006	0.092	0.107	0.103	0.069	0.084	0.094	0.116	0.075	0.008	0.010	0.006	0.014	0.020	0.013	0.018
F786_86.0711_9.7_P	0.013	0.007	0.008	0.007	0.007	0.044	0.007	0.007	0.002	0.002	0.001	0.020	0.002	0.031	0.003	0.019	0.039	0.010	0.012	0.004	0.003	0.008	0.017	0.006	0.001	0.009	0.009	0.002	0.028	0.012	0.002	0.003	0.001	0.002	0.002	0.015	0.005	0.002	0.110	0.101	0.142	0.093	0.107	0.102	0.140	0.113	0.002	0.002	0.002	0.010	0.025	0.001	0.006
F787_859.5328_3.1_N	0.072	0.092	0.077	0.104	0.047	0.114	0.090	0.053	0.101	0.106	0.093	0.086	0.072	0.150	0.061	0.073	0.116	0.101	0.082	0.079	0.062	0.046	0.057	0.130	0.083	0.073	0.152	0.075	0.057	0.104	0.130	0.107	0.089	0.086	0.087	0.074	0.078	0.107	0.076	0.089	0.101	0.068	0.045	0.049	0.050	0.031	0.079	0.061	0.075	0.091	0.063	0.037	0.093
F788_154.0719_10.4_P	0.039	0.027	0.054	0.042	0.057	0.059	0.027	0.058	0.053	0.044	0.030	0.038	0.031	0.030	0.032	0.042	0.042	0.027	0.034	0.036	0.032	0.024	0.033	0.053	0.045	0.048	0.046	0.058	0.038	0.050	0.041	0.038	0.033	0.040	0.056	0.063	0.057	0.057	0.094	0.083	0.080	0.109	0.112	0.077	0.119	0.105	0.105	0.098	0.156	0.146	0.120	0.080	0.124
F789_547.2935_4.2_N	0.047	0.003	0.002	0.001	0.001	0.001	0.099	0.001	0.001	0.001	0.001	0.001	0.001	0.015	0.001	0.136	0.001	0.001	0.001	0.007	0.001	0.001	0.006	0.001	0.001	0.001	0.037	0.067	0.001	0.018	0.043	0.001	0.146	0.001	0.001	0.001	0.002	0.001	0.003	0.050	0.012	0.003	0.005	0.072	0.006	0.015	0.001	0.001	0.001	0.001	0.066	0.001	0.002
F790_307.0431_11.3_P	0.095	0.017	0.107	0.414	0.050	0.052	0.624	0.038	0.136	0.101	0.081	0.089	0.074	0.030	0.062	0.052	0.056	0.119	0.439	0.117	0.094	0.065	0.077	0.059	0.075	0.050	0.047	0.077	0.038	0.084	0.103	0.090	0.093	0.081	0.063	0.072	0.103	0.119	0.075	0.061	0.069	0.172	0.062	0.077	0.102	0.036	0.104	0.072	0.123	0.083	0.055	0.064	0.733
F791_336.0969_14.4_N	0.156	0.157	0.140	0.184	0.076	0.133	0.115	0.106	0.137	0.114	0.072	0.154	0.106	0.028	0.112	0.088	0.142	0.190	0.144	0.137	0.067	0.082	0.096	0.117	0.093	0.104	0.099	0.100	0.076	0.119	0.097	0.123	0.054	0.104	0.104	0.127	0.154	0.112	0.122	0.086	0.116	0.228	0.098	0.099	0.098	0.065	0.067	0.049	0.068	0.075	0.032	0.024	0.139
F793_817.5552_3.3_P	0.249	0.480	0.181	0.321	0.103	0.145	0.484	0.144	0.147	0.232	0.102	0.162	0.129	0.831	0.153	0.113	0.138	0.319	0.428	0.179	0.107	0.140	0.168	0.079	0.160	0.111	0.121	0.132	0.137	0.194	0.139	0.100	0.113	0.188	0.136	0.101	0.156	0.190	0.120	0.143	0.159	0.401	0.212	0.150	0.100	0.141	0.099	0.080	0.081	0.104	0.055	0.083	0.285
F794_448.2509_14.2_P	0.020	0.007	0.047	0.032	0.048	0.041	0.010	0.056	0.023	0.018	0.034	0.006	0.091	0.034	0.069	0.017	0.042	0.037	0.004	0.021	0.101	0.027	0.068	0.129	0.047	0.098	0.030	0.039	0.062	0.047	0.049	0.060	0.063	0.044	0.022	0.037	0.035	0.081	0.017	0.011	0.033	0.025	0.022	0.007	0.035	0.018	0.029	0.029	0.025	0.013	0.029	0.034	0.006
F795_424.3412_4_P	0.146	0.058	0.132	0.092	0.079	0.070	0.133	0.078	0.175	0.086	0.103	0.137	0.065	0.072	0.131	0.103	0.083	0.176	0.082	0.160	0.146	0.050	0.095	0.093	0.113	0.083	0.117	0.083	0.094	0.198	0.115	0.116	0.131	0.056	0.153	0.108	0.038	0.166	0.069	0.035	0.051	0.157	0.159	0.115	0.049	0.117	0.131	0.037	0.261	0.157	0.157	0.477	0.086
F796_497.2266_14_P	0.082	0.063	0.068	0.088	0.080	0.079	0.042	0.075	0.063	0.045	0.091	0.083	0.092	0.059	0.117	0.102	0.059	0.086	0.047	0.059	0.067	0.064	0.041	0.079	0.088	0.067	0.076	0.066	0.147	0.112	0.085	0.145	0.130	0.098	0.100	0.170	0.079	0.135	0.091	0.054	0.097	0.120	0.118	0.078	0.078	0.074	0.092	0.064	0.105	0.126	0.094	0.100	0.071
F797_353.2681_3.5_P	0.150	0.210	0.080	0.142	0.062	0.100	0.174	0.178	0.130	0.121	0.059	0.079	0.072	0.351	0.114	0.066	0.077	0.111	0.147	0.079	0.062	0.097	0.081	0.098	0.065	0.087	0.047	0.105	0.037	0.118	0.072	0.105	0.062	0.078	0.115	0.085	0.094	0.232	0.192	0.216	0.188	0.250	0.229	0.220	0.166	0.160	0.144	0.202	0.177	0.100	0.065	0.145	0.295
F798_189.0979_16.3_P	0.052	0.018	0.039	0.035	0.042	0.071	0.014	0.047	0.020	0.014	0.033	0.042	0.057	0.022	0.043	0.074	0.045	0.032	0.025	0.022	0.025	0.064	0.070	0.044	0.040	0.040	0.051	0.047	0.108	0.050	0.047	0.054	0.044	0.069	0.052	0.053	0.037	0.024	0.042	0.048	0.069	0.040	0.060	0.044	0.044	0.040	0.041	0.030	0.027	0.078	0.104	0.039	0.020
F799_518.0922_15.9_P	0.037	0.007	0.037	0.025	0.045	0.052	0.009	0.052	0.041	0.055	0.044	0.039	0.042	0.035	0.038	0.056	0.049	0.031	0.034	0.058	0.042	0.034	0.035	0.044	0.045	0.041	0.047	0.062	0.062	0.056	0.037	0.048	0.046	0.047	0.039	0.048	0.040	0.059	0.047	0.048	0.051	0.052	0.051	0.039	0.055	0.043	0.092	0.112	0.066	0.078	0.069	0.098	0.052
F800_236.1488_13.1_P	0.030	0.007	0.056	0.023	0.045	0.085	0.010	0.045	0.027	0.032	0.021	0.059	0.039	0.036	0.039	0.062	0.061	0.036	0.035	0.034	0.022	0.029	0.045	0.031	0.019	0.026	0.059	0.031	0.073	0.065	0.028	0.040	0.014	0.058	0.050	0.064	0.081	0.036	0.099	0.069	0.075	0.064	0.068	0.044	0.050	0.051	0.047	0.037	0.037	0.054	0.053	0.030	0.035
F801_119.0349_10.8_N	0.028	0.031	0.057	0.041	0.075	0.083	0.034	0.076	0.064	0.030	0.050	0.065	0.080	0.180	0.067	0.074	0.063	0.057	0.027	0.042	0.057	0.070	0.064	0.086	0.055	0.077	0.099	0.065	0.085	0.045	0.090	0.077	0.060	0.037	0.034	0.070	0.090	0.065	0.063	0.070	0.064	0.036	0.050	0.067	0.082	0.082	0.049	0.061	0.062	0.060	0.059	0.061	0.032
F804_360.1957_25.3_P	0.082	0.022	0.082	0.066	0.079	0.075	0.013	0.064	0.090	0.076	0.108	0.072	0.114	0.029	0.093	0.092	0.082	0.105	0.086	0.076	0.122	0.119	0.100	0.091	0.105	0.086	0.093	0.106	0.093	0.076	0.155	0.074	0.147	0.123	0.111	0.104	0.110	0.060	0.049	0.048	0.057	0.028	0.052	0.042	0.051	0.050	0.077	0.079	0.110	0.099	0.103	0.071	0.037
F805_395.1509_11.5_P	0.072	0.117	0.122	0.076	0.031	0.051	0.055	0.041	0.056	0.125	0.114	0.107	0.062	0.058	0.110	0.070	0.129	0.076	0.050	0.051	0.064	0.039	0.089	0.065	0.069	0.104	0.042	0.135	0.037	0.051	0.051	0.035	0.031	0.066	0.038	0.063	0.046	0.098	0.029	0.031	0.036	0.040	0.040	0.036	0.037	0.038	0.058	0.038	0.044	0.040	0.033	0.032	0.032
F806_306.0575_10_N	0.045	0.022	0.034	0.026	0.060	0.067	0.025	0.038	0.065	0.107	0.078	0.127	0.091	0.079	0.116	0.088	0.092	0.064	0.060	0.063	0.059	0.046	0.045	0.051	0.096	0.065	0.091	0.089	0.074	0.106	0.065	0.083	0.072	0.066	0.074	0.087	0.117	0.084	0.066	0.066	0.070	0.051	0.067	0.059	0.081	0.068	0.077	0.066	0.082	0.045	0.056	0.066	0.061
F807_162.0756_11_P	0.061	0.051	0.075	0.061	0.058	0.053	0.042	0.062	0.074	0.051	0.064	0.063	0.065	0.038	0.064	0.050	0.046	0.070	0.056	0.060	0.058	0.065	0.063	0.063	0.065	0.059	0.051	0.046	0.045	0.047	0.053	0.075	0.071	0.057	0.067	0.068	0.081	0.048	0.058	0.069	0.063	0.063	0.062	0.072	0.044	0.056	0.028	0.029	0.035	0.033	0.044	0.021	0.024
F809_454.3284_4.2_P	0.104	0.053	0.076	0.086	0.066	0.064	0.018	0.090	0.078	0.046	0.040	0.058	0.041	0.026	0.045	0.038	0.046	0.058	0.037	0.056	0.056	0.070	0.036	0.101	0.065	0.071	0.048	0.065	0.063	0.054	0.049	0.047	0.042	0.045	0.041	0.058	0.038	0.063	0.054	0.069	0.045	0.055	0.061	0.028	0.060	0.051	0.053	0.103	0.068	0.061	0.051	0.058	0.043
F810_114.0548_14.8_P	0.085	0.061	0.071	0.068	0.069	0.108	0.056	0.127	0.105	0.073	0.099	0.062	0.111	0.082	0.088	0.090	0.115	0.106	0.085	0.122	0.113	0.089	0.095	0.078	0.126	0.043	0.085	0.093	0.114	0.112	0.123	0.137	0.171	0.103	0.101	0.119	0.122	0.166	0.056	0.045	0.055	0.057	0.078	0.050	0.048	0.062	0.100	0.063	0.108	0.092	0.099	0.100	0.083
F811_288.191_11.1_P	0.122	0.124	0.118	0.127	0.090	0.072	0.059	0.126	0.142	0.168	0.075	0.127	0.121	0.048	0.125	0.118	0.130	0.139	0.084	0.145	0.079	0.101	0.093	0.127	0.137	0.114	0.129	0.150	0.120	0.143	0.118	0.149	0.113	0.128	0.105	0.129	0.142	0.144	0.069	0.084	0.074	0.121	0.094	0.081	0.097	0.077	0.205	0.136	0.134	0.112	0.087	0.122	0.122
F812_113.0244_14.8_N	0.059	0.043	0.037	0.044	0.062	0.051	0.044	0.060	0.072	0.062	0.071	0.052	0.074	0.113	0.059	0.069	0.061	0.090	0.065	0.074	0.085	0.095	0.108	0.048	0.104	0.012	0.061	0.049	0.098	0.075	0.088	0.071	0.089	0.149	0.073	0.058	0.069	0.061	0.036	0.040	0.037	0.051	0.043	0.029	0.037	0.040	0.064	0.051	0.052	0.057	0.050	0.053	0.050
F813_367.12_14.3_P	0.074	0.081	0.045	0.057	0.035	0.042	0.062	0.040	0.053	0.074	0.050	0.045	0.052	0.042	0.046	0.045	0.053	0.056	0.061	0.061	0.045	0.048	0.041	0.043	0.046	0.052	0.048	0.074	0.045	0.072	0.051	0.052	0.050	0.053	0.056	0.062	0.056	0.072	0.045	0.047	0.056	0.091	0.045	0.046	0.064	0.042	0.110	0.108	0.105	0.087	0.062	0.088	0.192
F814_549.3193_4.5_P	0.001	0.001	0.001	0.001	0.001	0.001	0.002	0.001	0.001	0.001	0.001	0.001	0.002	0.005	0.001	0.070	0.001	0.001	0.001	0.012	0.001	0.001	0.004	0.003	0.001	0.001	0.005	0.029	0.001	0.013	0.011	0.001	0.058	0.001	0.001	0.001	0.001	0.001	0.002	0.021	0.002	0.001	0.001	0.090	0.002	0.009	0.000	0.001	0.001	0.001	0.031	0.001	0.001
F815_387.1901_16.2_P	0.059	0.013	0.084	0.066	0.078	0.100	0.017	0.091	0.101	0.060	0.105	0.079	0.097	0.047	0.090	0.087	0.083	0.064	0.064	0.078	0.097	0.085	0.076	0.099	0.100	0.072	0.111	0.072	0.102	0.082	0.103	0.127	0.137	0.130	0.142	0.108	0.090	0.084	0.123	0.106	0.126	0.084	0.141	0.106	0.117	0.113	0.093	0.063	0.076	0.087	0.112	0.121	0.029
F816_728.5223_3.4_P	0.174	0.368	0.131	0.198	0.060	0.079	0.203	0.095	0.195	0.206	0.052	0.123	0.115	0.395	0.079	0.078	0.113	0.176	0.336	0.152	0.049	0.051	0.081	0.064	0.082	0.087	0.067	0.113	0.055	0.100	0.082	0.069	0.051	0.082	0.083	0.070	0.067	0.074	0.061	0.073	0.009	0.127	0.050	0.050	0.101	0.077	0.070	0.072	0.139	0.059	0.062	0.077	0.174
F817_205.0598_13.7_N	0.075	0.157	0.087	0.109	0.095	0.073	0.141	0.104	0.085	0.080	0.093	0.083	0.104	0.167	0.104	0.075	0.101	0.094	0.101	0.072	0.108	0.071	0.074	0.099	0.094	0.093	0.101	0.092	0.061	0.070	0.072	0.092	0.098	0.075	0.072	0.073	0.094	0.121	0.077	0.056	0.044	0.064	0.072	0.066	0.051	0.046	0.082	0.063	0.083	0.063	0.055	0.073	0.145
F818_576.2938_4_N	0.065	0.042	0.046	0.048	0.067	0.044	0.037	0.050	0.062	0.035	0.026	0.050	0.039	0.016	0.041	0.023	0.036	0.037	0.035	0.048	0.040	0.043	0.042	0.043	0.039	0.047	0.043	0.047	0.039	0.029	0.027	0.025	0.029	0.023	0.023	0.027	0.023	0.014	0.052	0.059	0.043	0.060	0.048	0.041	0.045	0.047	0.020	0.035	0.023	0.025	0.017	0.023	0.012
F819_372.1508_11_P	0.198	0.433	0.084	0.205	0.037	0.039	0.008	0.043	0.088	0.159	0.058	0.077	0.044	0.477	0.054	0.058	0.063	0.118	0.225	0.098	0.046	0.034	0.049	0.045	0.063	0.054	0.033	0.071	0.032	0.083	0.067	0.048	0.054	0.093	0.074	0.057	0.074	0.136	0.036	0.031	0.045	0.239	0.043	0.024	0.061	0.026	0.098	0.054	0.105	0.065	0.037	0.050	0.452
F820_150.0758_16.1_P	0.033	0.009	0.020	0.014	0.037	0.037	0.005	0.036	0.025	0.021	0.038	0.031	0.029	0.029	0.022	0.039	0.025	0.026	0.019	0.016	0.020	0.032	0.033	0.028	0.040	0.018	0.050	0.032	0.088	0.035	0.047	0.046	0.050	0.059	0.071	0.064	0.039	0.037	0.025	0.040	0.046	0.034	0.035	0.029	0.031	0.042	0.041	0.037	0.042	0.061	0.092	0.060	0.023
F821_293.1758_3.5_N	0.003	0.011	0.004	0.004	0.005	0.009	0.020	0.004	0.004	0.005	0.003	0.003	0.006	0.181	0.005	0.004	0.003	0.006	0.006	0.003	0.004	0.004	0.003	0.004	0.004	0.004	0.004	0.003	0.004	0.004	0.003	0.003	0.004	0.008	0.037	0.003	0.003	0.004	0.033	0.022	0.034	0.020	0.059	0.028	0.031	0.043	0.033	0.034	0.056	0.024	0.029	0.036	0.044
F822_168.0764_7.5_P	0.038	0.014	0.059	0.043	0.058	0.075	0.034	0.073	0.055	0.034	0.055	0.069	0.072	0.045	0.068	0.066	0.052	0.055	0.031	0.038	0.046	0.055	0.054	0.078	0.052	0.051	0.072	0.047	0.070	0.059	0.061	0.083	0.049	0.062	0.057	0.089	0.103	0.056	0.073	0.081	0.058	0.008	0.071	0.088	0.054	0.058	0.051	0.043	0.046	0.085	0.077	0.047	0.035
F823_180.0876_7.8_P	0.024	0.006	0.030	0.017	0.034	0.058	0.004	0.032	0.029	0.022	0.029	0.107	0.039	0.101	0.042	0.052	0.043	0.026	0.034	0.033	0.025	0.037	0.043	0.038	0.031	0.028	0.034	0.034	0.069	0.045	0.044	0.039	0.037	0.043	0.040	0.046	0.047	0.045	0.033	0.041	0.040	0.023	0.044	0.038	0.046	0.044	0.074	0.061	0.077	0.098	0.110	0.085	0.038
F824_229.0827_10.1_N	0.016	0.019	0.031	0.019	0.048	0.030	0.012	0.046	0.029	0.016	0.024	0.012	0.044	0.015	0.029	0.027	0.032	0.016	0.016	0.026	0.058	0.050	0.047	0.070	0.042	0.080	0.046	0.049	0.098	0.046	0.050	0.082	0.080	0.042	0.038	0.050	0.045	0.037	0.029	0.032	0.055	0.021	0.042	0.020	0.041	0.030	0.040	0.062	0.040	0.050	0.066	0.055	0.015
F825_344.1562_14.8_P	0.137	0.129	0.092	0.123	0.073	0.111	0.082	0.115	0.161	0.115	0.100	0.086	0.126	0.055	0.102	0.090	0.141	0.172	0.156	0.175	0.150	0.080	0.051	0.098	0.131	0.080	0.098	0.122	0.089	0.151	0.167	0.152	0.162	0.128	0.125	0.140	0.144	0.279	0.073	0.053	0.074	0.096	0.099	0.061	0.073	0.062	0.155	0.076	0.161	0.118	0.107	0.109	0.123
F826_841.9073_10.2_N	0.005	0.012	0.022	0.016	0.067	0.021	0.014	0.054	0.124	0.039	0.074	0.007	0.145	0.062	0.096	0.047	0.126	0.047	0.017	0.114	0.243	0.106	0.132	0.429	0.287	0.389	0.241	0.287	0.278	0.223	0.315	0.373	0.361	0.250	0.069	0.114	0.254	0.391	0.193	0.088	0.255	0.187	0.265	0.118	0.242	0.196	0.243	0.095	0.146	0.042	0.212	0.200	0.047
F827_165.0224_10.1_N	0.007	0.004	0.003	0.005	0.001	0.018	0.003	0.002	0.001	0.001	0.002	0.014	0.001	0.013	0.001	0.026	0.051	0.005	0.020	0.008	0.001	0.009	0.140	0.003	0.001	0.003	0.004	0.003	0.023	0.002	0.005	0.004	0.001	0.002	0.001	0.009	0.002	0.002	0.031	0.069	0.032	0.014	0.057	0.104	0.021	0.073	0.001	0.001	0.001	0.006	0.006	0.001	0.002
F828_466.0822_14.3_P	0.032	0.009	0.032	0.017	0.041	0.042	0.010	0.046	0.037	0.044	0.045	0.043	0.046	0.043	0.041	0.043	0.042	0.025	0.011	0.036	0.038	0.042	0.036	0.054	0.042	0.053	0.062	0.069	0.061	0.066	0.043	0.047	0.048	0.034	0.037	0.060	0.053	0.061	0.049	0.090	0.073	0.059	0.065	0.069	0.068	0.080	0.135	0.262	0.119	0.117	0.138	0.180	0.082
F829_117.0657_16.2_P	0.055	0.028	0.055	0.053	0.082	0.086	0.029	0.092	0.069	0.058	0.089	0.061	0.074	0.109	0.060	0.078	0.064	0.049	0.059	0.066	0.080	0.096	0.080	0.088	0.081	0.063	0.093	0.075	0.097	0.078	0.076	0.086	0.097	0.105	0.096	0.085	0.062	0.091	0.065	0.063	0.060	0.053	0.076	0.049	0.070	0.075	0.104	0.093	0.101	0.112	0.105	0.124	0.051
F830_289.0586_10.2_P	0.007	0.007	0.012	0.006	0.020	0.021	0.007	0.023	0.013	0.018	0.022	0.018	0.020	0.034	0.011	0.027	0.017	0.010	0.006	0.023	0.019	0.032	0.023	0.030	0.022	0.020	0.049	0.051	0.047	0.050	0.030	0.022	0.023	0.019	0.022	0.037	0.021	0.031	0.037	0.037	0.062	0.049	0.058	0.060	0.075	0.064	0.156	0.167	0.107	0.128	0.127	0.116	0.055

F831_120.0554_7.8_P	0.036	0.039	0.024	0.024	0.050	0.076	0.021	0.039	0.023	0.017	0.028	0.049	0.060	0.017	0.037	0.067	0.053	0.024	0.021	0.014	0.012	0.032	0.036	0.033	0.028	0.023	0.059	0.020	0.091	0.040	0.034	0.026	0.017	0.047	0.044	0.046	0.068	0.007	0.088	0.089	0.012	0.015	0.041	0.091	0.054	0.086	0.003	0.003	0.009	0.012	0.011	0.003	0.030
F832_144.0804_10_P	0.036	0.019	0.052	0.036	0.068	0.070	0.012	0.069	0.050	0.035	0.065	0.055	0.070	0.035	0.074	0.070	0.060	0.040	0.034	0.059	0.063	0.061	0.057	0.046	0.050	0.053	0.059	0.048	0.065	0.074	0.059	0.074	0.069	0.062	0.075	0.057	0.084	0.061	0.077	0.091	0.101	0.056	0.091	0.075	0.098	0.105	0.054	0.048	0.057	0.058	0.092	0.062	0.033
F833_310.2735_4.8_P	0.203	0.259	0.171	0.243	0.063	0.052	0.208	0.080	0.189	0.149	0.035	0.166	0.057	0.129	0.050	0.035	0.075	0.290	0.125	0.102	0.051	0.022	0.033	0.061	0.090	0.094	0.059	0.210	0.019	0.027	0.031	0.027	0.020	0.029	0.053	0.038	0.021	0.038	0.078	0.090	0.014	0.182	0.028	0.098	0.044	0.024	0.010	0.014	0.069	0.027	0.007	0.008	0.026
F835_237.1496_3.5_N	0.065	0.101	0.076	0.087	0.099	0.084	0.071	0.064	0.080	0.087	0.093	0.109	0.106	0.096	0.111	0.085	0.079	0.127	0.062	0.073	0.076	0.075	0.075	0.087	0.098	0.060	0.083	0.078	0.083	0.067	0.093	0.083	0.061	0.072	0.053	0.101	0.089	0.107	0.045	0.038	0.034	0.049	0.058	0.056	0.032	0.039	0.062	0.077	0.055	0.076	0.063	0.075	0.070
F836_298.114_14.8_N	0.167	0.446	0.072	0.158	0.038	0.048	0.481	0.042	0.100	0.140	0.054	0.088	0.084	0.905	0.067	0.078	0.048	0.200	0.197	0.047	0.107	0.081	0.065	0.065	0.077	0.068	0.079	0.133	0.049	0.032	0.118	0.090	0.082	0.046	0.057	0.097	0.066	0.054	0.048	0.081	0.060	0.103	0.048	0.088	0.073	0.096	0.061	0.040	0.082	0.064	0.048	0.048	0.268
F837_134.0611_8.7_N	0.020	0.012	0.020	0.015	0.045	0.027	0.013	0.037	0.017	0.016	0.016	0.017	0.019	0.014	0.022	0.018	0.024	0.016	0.018	0.020	0.025	0.022	0.020	0.041	0.023	0.037	0.028	0.027	0.029	0.033	0.018	0.031	0.023	0.030	0.026	0.023	0.029	0.022	0.044	0.045	0.050	0.046	0.071	0.034	0.052	0.062	0.055	0.087	0.083	0.075	0.063	0.116	0.050
F838_168.1227_7.2_P	0.010	0.013	0.044	0.008	0.024	0.043	0.012	0.075	0.077	0.006	0.033	0.046	0.043	0.140	0.049	0.008	0.057	0.008	0.004	0.005	0.053	0.047	0.052	0.034	0.061	0.035	0.015	0.021	0.036	0.046	0.037	0.125	0.027	0.070	0.065	0.047	0.165	0.101	0.038	0.015	0.023	0.007	0.024	0.010	0.033	0.018	0.077	0.113	0.073	0.042	0.009	0.037	0.017
F839_132.0301_13.2_N	0.073	0.104	0.054	0.063	0.047	0.075	0.110	0.051	0.053	0.045	0.064	0.059	0.048	0.059	0.055	0.058	0.085	0.069	0.063	0.052	0.039	0.041	0.061	0.058	0.044	0.041	0.053	0.038	0.094	0.051	0.059	0.058	0.041	0.050	0.047	0.076	0.047	0.065	0.053	0.044	0.054	0.048	0.050	0.058	0.049	0.049	0.048	0.043	0.053	0.079	0.086	0.053	0.074
F840_245.1492_10.3_P	0.040	0.053	0.074	0.040	0.073	0.074	0.058	0.055	0.048	0.036	0.043	0.079	0.071	0.257	0.058	0.072	0.059	0.051	0.032	0.044	0.045	0.057	0.081	0.055	0.036	0.058	0.074	0.045	0.080	0.063	0.052	0.073	0.075	0.068	0.056	0.087	0.077	0.032	0.093	0.082	0.071	0.064	0.093	0.083	0.049	0.067	0.060	0.046	0.039	0.101	0.084	0.043	0.033
F842_255.1334_12.7_P	0.015	0.009	0.011	0.013	0.009	0.034	0.015	0.010	0.004	0.003	0.005	0.021	0.013	0.044	0.008	0.017	0.016	0.015	0.006	0.008	0.004	0.014	0.015	0.024	0.007	0.015	0.013	0.006	0.032	0.028	0.009	0.024	0.008	0.013	0.018	0.041	0.042	0.012	0.094	0.087	0.098	0.062	0.107	0.104	0.054	0.065	0.005	0.013	0.006	0.025	0.052	0.007	0.010
F843_488.1761_13.9_P	0.101	0.040	0.062	0.070	0.041	0.069	0.030	0.062	0.071	0.041	0.051	0.090	0.081	0.043	0.068	0.104	0.069	0.109	0.057	0.058	0.037	0.079	0.096	0.057	0.056	0.050	0.059	0.079	0.046	0.090	0.061	0.070	0.054	0.066	0.093	0.075	0.091	0.051	0.046	0.050	0.060	0.066	0.057	0.069	0.035	0.034	0.050	0.045	0.049	0.084	0.044	0.039	0.052
F844_306.0801_12.9_P	0.053	0.052	0.049	0.047	0.047	0.063	0.020	0.040	0.038	0.045	0.064	0.067	0.050	0.044	0.058	0.037	0.102	0.060	0.062	0.065	0.078	0.050	0.057	0.028	0.081	0.026	0.084	0.100	0.063	0.112	0.083	0.079	0.081	0.101	0.070	0.103	0.058	0.124	0.113	0.067	0.064	0.153	0.065	0.049	0.102	0.075	0.073	0.063	0.145	0.129	0.058	0.073	0.178
F845_86.0598_7.6_P	0.046	0.010	0.036	0.027	0.034	0.019	0.016	0.026	0.071	0.045	0.068	0.025	0.045	0.186	0.030	0.038	0.113	0.051	0.129	0.104	0.090	0.051	0.167	0.017	0.026	0.037	0.051	0.033	0.060	0.025	0.107	0.024	0.063	0.033	0.058	0.044	0.033	0.088	0.038	0.033	0.051	0.036	0.062	0.026	0.062	0.070	0.060	0.034	0.070	0.056	0.056	0.042	0.027
F846_533.2925_7.6_P	0.010	0.010	0.015	0.009	0.023	0.018	0.009	0.020	0.012	0.009	0.010	0.002	0.034	0.042	0.019	0.009	0.019	0.014	0.004	0.009	0.038	0.016	0.025	0.104	0.025	0.081	0.031	0.046	0.045	0.028	0.033	0.052	0.036	0.023	0.008	0.018	0.032	0.040	0.024	0.015	0.037	0.020	0.036	0.018	0.045	0.026	0.032	0.056	0.015	0.007	0.039	0.039	0.007
F847_844.57_3.3_N	0.151	0.327	0.237	0.265	0.032	0.194	0.005	0.102	0.221	0.168	0.152	0.214	0.235	1.431	0.247	0.159	0.247	0.214	0.134	0.184	0.180	0.021	0.121	0.208	0.172	0.251	0.241	0.228	0.099	0.172	0.221	0.249	0.204	0.156	0.226	0.192	0.249	0.164	0.154	0.184	0.170	0.179	0.144	0.187	0.149	0.137	0.184	0.261	0.229	0.173	0.129	0.164	0.365
F848_279.2316_3.1_P	0.066	0.067	0.063	0.084	0.062	0.059	0.073	0.044	0.088	0.082	0.056	0.109	0.061	0.084	0.091	0.059	0.049	0.074	0.055	0.041	0.038	0.061	0.055	0.090	0.100	0.081	0.105	0.104	0.075	0.061	0.047	0.105	0.054	0.045	0.044	0.066	0.085	0.056	0.050	0.066	0.040	0.039	0.050	0.054	0.030	0.037	0.040	0.049	0.031	0.042	0.038	0.042	0.061
F849_368.2872_3.5_P	0.245	0.510	0.089	0.226	0.075	0.122	0.153	0.218	0.147	0.135	0.025	0.029	0.061	0.140	0.167	0.029	0.064	0.163	0.040	0.076	0.052	0.165	0.047	0.147	0.026	0.110	0.039	0.134	0.032	0.101	0.043	0.023	0.058	0.015	0.093	0.046	0.069	0.028	0.332	0.478	0.360	0.684	0.545	0.621	0.479	0.489	0.204	0.340	0.242	0.137	0.054	0.200	0.228
F850_160.0613_9.8_N	0.086	0.053	0.083	0.066	0.092	0.105	0.052	0.076	0.083	0.054	0.081	0.114	0.087	0.055	0.095	0.137	0.093	0.069	0.056	0.054	0.088	0.125	0.103	0.085	0.089	0.076	0.114	0.098	0.138	0.085	0.095	0.089	0.103	0.074	0.094	0.128	0.129	0.084	0.066	0.076	0.095	0.045	0.099	0.090	0.097	0.099	0.082	0.054	0.078	0.104	0.121	0.103	0.049
F851_361.1825_15.1_P	0.006	0.005	0.005	0.004	0.015	0.016	0.008	0.023	0.005	0.010	0.006	0.003	0.018	0.021	0.030	0.005	0.017	0.019	0.005	0.004	0.004	0.011	0.004	0.044	0.003	0.019	0.014	0.015	0.016	0.014	0.011	0.013	0.004	0.007	0.006	0.026	0.012	0.004	0.035	0.024	0.068	0.024	0.047	0.021	0.061	0.026	0.090	0.162	0.055	0.057	0.040	0.104	0.017
F852_482.36_4.1_P	0.148	0.104	0.151	0.105	0.078	0.083	0.082	0.103	0.147	0.075	0.074	0.103	0.074	0.023	0.074	0.061	0.098	0.060	0.103	0.096	0.127	0.084	0.074	0.169	0.120	0.102	0.068	0.119	0.119	0.062	0.090	0.065	0.080	0.065	0.056	0.096	0.061	0.092	0.055	0.063	0.061	0.115	0.078	0.041	0.108	0.066	0.138	0.158	0.153	0.101	0.097	0.092	0.110
F853_855.5696_3.2_P	0.509	0.935	0.250	0.522	0.217	0.225	0.742	0.220	0.372	0.437	0.192	0.295	0.272	1.295	0.266	0.231	0.264	0.598	0.649	0.376	0.251	0.247	0.254	0.234	0.272	0.201	0.187	0.268	0.179	0.301	0.330	0.294	0.231	0.320	0.334	0.288	0.328	0.445	0.237	0.185	0.242	0.615	0.268	0.173	0.179	0.184	0.054	0.176	0.239	0.211	0.162	0.219	0.638
F854_295.2639_3.3_N	0.074	0.131	0.188	0.117	0.207	0.203	0.083	0.147	0.145	0.113	0.192	0.163	0.227	0.039	0.208	0.161	0.134	0.079	0.072	0.133	0.134	0.159	0.141	0.223	0.179	0.167	0.141	0.128	0.163	0.096	0.133	0.103	0.157	0.096	0.062	0.126	0.072	0.092	0.049	0.052	0.028	0.018	0.058	0.060	0.037	0.047	0.070	0.054	0.091	0.111	0.108	0.091	0.039
F856_160.0966_11.6_P	0.112	0.086	0.197	0.173	0.181	0.238	0.084	0.217	0.149	0.137	0.149	0.167	0.185	0.542	0.212	0.195	0.158	0.210	0.090	0.204	0.132	0.143	0.155	0.152	0.147	0.128	0.200	0.132	0.213	0.170	0.112	0.158	0.094	0.136	0.123	0.215	0.222	0.234	0.269	0.201	0.247	0.237	0.248	0.222	0.229	0.236	0.179	0.210	0.158	0.219	0.180	0.151	0.082
F857_200.139_24.8_P	0.072	0.024	0.058	0.040	0.072	0.108	0.008	0.074	0.060	0.040	0.067	0.062	0.082	0.032	0.078	0.091	0.072	0.100	0.042	0.031	0.040	0.074	0.073	0.077	0.135	0.065	0.113	0.068	0.149	0.076	0.134	0.145	0.143	0.170	0.162	0.158	0.124	0.050	0.029	0.029	0.030	0.020	0.031	0.026	0.012	0.028	0.110	0.107	0.109	0.189	0.193	0.122	0.061
F858_148.0965_15.8_P	0.024	0.050	0.041	0.029	0.184	0.097	0.115	0.066	0.053	0.098	0.263	0.144	0.097	0.195	0.168	0.230	0.110	0.072	0.068	0.046	0.236	0.064	0.094	0.028	0.041	0.035	0.131	0.049	0.065	0.062	0.070	0.038	0.040	0.067	0.021	0.037	0.032	0.064	0.050	0.067	0.045	0.022	0.077	0.017	0.018	0.043	0.033	0.024	0.063	0.129	0.032	0.111	0.030
F859_452.3127_4.2_P	0.299	0.160	0.231	0.228	0.217	0.151	0.225	0.218	0.156	0.081	0.074	0.105	0.103	0.072	0.121	0.072	0.103	0.120	0.073	0.117	0.117	0.094	0.090	0.165	0.129	0.150	0.133	0.140	0.098	0.105	0.081	0.105	0.100	0.113	0.081	0.117	0.094	0.085	0.113	0.144	0.122	0.128	0.144	0.085	0.120	0.094	0.121	0.209	0.159	0.147	0.100	0.158	0.118
F860_480.1752_11_P	0.643	1.403	0.262	0.659	0.136	0.146	1.243	0.126	0.284	0.513	0.231	0.202	0.167	1.087	0.198	0.143	0.210	0.582	0.947	0.348	0.200	0.137	0.199	0.156	0.190	0.150	0.135	0.241	0.106	0.237	0.253	0.184	0.233	0.312	0.292	0.182	0.252	0.386	0.156	0.127	0.164	0.566	0.178	0.134	0.200	0.125	0.219	0.188	0.314	0.219	0.155	0.164	1.131
F862_134.0804_14.9_P	0.239	0.204	0.344	0.329	0.377	0.275	0.173	0.217	0.183	0.464	0.292	0.374	0.222	0.166	0.230	0.331	0.261	0.377	0.263	0.247	0.257	0.345	0.318	0.383	0.228	0.461	0.238	0.230	0.266	0.227	0.224	0.411	0.232	0.524	0.410	0.268	0.379	0.201	0.214	0.278	0.305	0.185	0.183	0.144	0.230	0.206	0.305	0.465	0.206	0.252	0.319	0.279	0.246
F863_309.0432_14.3_N	0.363	0.084	0.302	0.208	0.426	0.416	0.061	0.465	0.361	0.477	0.512	0.389	0.454	0.035	0.406	0.462	0.404	0.253	0.210	0.434	0.383	0.429	0.384	0.528	0.410	0.532	0.687	0.717	0.676	0.742	0.450	0.448	0.506	0.370	0.391	0.671	0.488	0.586	0.579	0.931	0.808	0.669	0.769	0.801	0.769	0.918	1.530	2.532	1.328	1.385	1.500	1.877	0.979
F864_416.1841_11.9_P	0.139	0.090	0.155	0.151	0.124	0.144	0.083	0.140	0.198	0.129	0.186	0.171	0.183	0.067	0.199	0.177	0.201	0.162	0.125	0.204	0.201	0.165	0.186	0.115	0.178	0.134	0.111	0.155	0.152	0.221	0.212	0.229	0.198	0.216	0.209	0.177	0.252	0.203	0.134	0.117	0.167	0.121	0.096	0.117	0.132	0.108	0.154	0.091	0.165	0.168	0.219	0.114	0.118
F865_259.9852_15.4_N	0.240	0.229	0.223	0.230	0.165	0.246	0.218	0.303	0.283	0.243	0.279	0.235	0.319	0.137	0.292	0.219	0.314	0.297	0.283	0.292	0.345	0.480	0.317	0.243	0.309	0.269	0.266	0.251	0.288	0.386	0.380	0.366	0.346	0.385	0.365	0.282	0.412	0.411	0.189	0.179	0.201	0.251	0.382	0.195	0.215	0.222	0.260	0.272	0.335	0.271	0.267	0.285	0.316
F866_224.0597_8.1_N	0.009	0.022	0.032	0.014	0.031	0.049	0.019	0.019	0.018	0.008	0.008	0.025	0.020	0.012	0.026	0.022	0.017	0.012	0.010	0.008	0.006	0.008	0.011	0.031	0.010	0.034	0.041	0.019	0.019	0.006	0.019	0.021	0.009	0.007	0.006	0.022	0.030	0.009	0.041	0.163	0.011	0.008	0.011	0.125	0.008	0.013	0.012	0.018	0.007	0.011	0.005	0.005	0.006
F867_304.2361_3.4_N	0.106	0.144	0.198	0.198	0.244	0.224	0.057	0.221	0.180	0.140	0.219	0.171	0.237	0.081	0.226	0.201	0.135	0.087	0.069	0.148	0.178	0.170	0.146	0.214	0.186	0.204	0.177	0.144	0.151	0.152	0.161	0.110	0.124	0.112	0.074	0.176	0.097	0.136	0.176	0.153	0.091	0.082	0.204	0.192	0.159	0.180	0.115	0.147	0.106	0.158	0.123	0.104	0.093
F868_329.1735_15.7_P	0.194	0.236	0.099	0.131	0.083	0.120	0.165	0.090	0.095	0.141	0.119	0.113	0.089	0.118	0.090	0.083	0.078	0.113	0.142	0.106	0.059	0.091	0.063	0.099	0.112	0.080	0.124	0.173	0.095	0.144	0.084	0.093	0.094	0.084	0.099	0.143	0.098	0.104	0.151	0.166	0.160	0.291	0.126	0.157	0.173	0.115	0.186	0.142	0.173	0.152	0.108	0.102	0.353
F869_263.1652_3.4_N	0.138	0.212	0.120	0.122	0.193	0.145	0.166	0.139	0.170	0.163	0.204	0.176	0.183	0.134	0.202	0.147	0.131	0.201	0.123	0.137	0.154	0.152	0.162	0.178	0.178	0.123	0.145	0.169	0.168	0.116	0.170	0.149	0.108	0.166	0.079	0.185	0.123	0.164	0.060	0.040	0.036	0.057	0.068	0.056	0.042	0.054	0.119	0.136	0.086	0.146	0.092	0.122	0.135
F870_280.1538_7.5_P	0.021	0.004	0.014	0.007	0.009	0.031	0.016	0.009	0.006	0.004	0.007	0.029	0.011	0.031	0.009	0.011	0.010	0.009	0.011	0.007	0.004	0.014	0.012	0.008	0.008	0.005	0.010	0.006	0.019	0.011	0.010	0.008	0.008	0.022	0.015	0.011	0.010	0.006	0.104	0.109	0.105	0.003	0.120	0.195	0.108	0.103	0.008	0.008	0.008	0.017	0.027	0.008	0.005
F871_357.2487_21.4_P	0.027	0.020	0.090	0.036	0.064	0.191	0.020	0.071	0.054	0.026	0.038	0.104	0.059	0.034	0.068	0.089	0.053	0.040	0.022	0.036	0.078	0.099	0.050	0.068	0.035	0.058	0.101	0.032	0.060	0.061	0.045	0.071	0.044	0.032	0.057	0.058	0.078	0.029	0.115	0.120	0.122	0.051	0.084	0.187	0.073	0.090	0.108	0.188	0.072	0.085	0.149	0.086	0.039
F872_508.376_4_P	0.175	0.074	0.127	0.135	0.116	0.091	0.076	0.135	0.137	0.064	0.112	0.088	0.086	0.034	0.115	0.108	0.114	0.084	0.077	0.137	0.133	0.103	0.116	0.129	0.097	0.168	0.122	0.080	0.140	0.136	0.053	0.070	0.129	0.068	0.079	0.090	0.072	0.113	0.066	0.042	0.078	0.100	0.083	0.071	0.144	0.089	0.165	0.210	0.155	0.154	0.116	0.191	0.120
F873_583.3443_3.9_N	0.289	0.168	0.148	0.199	0.209	0.126	0.217	0.172	0.237	0.091	0.111	0.090	0.160	0.098	0.100	0.083	0.164	0.166	0.203	0.151	0.195	0.176	0.123	0.130	0.147	0.210	0.225	0.170	0.201	0.148	0.147	0.107	0.101	0.125	0.161	0.122	0.119	0.112	0.202	0.166	0.190	0.265	0.230	0.121	0.208	0.172	0.169	0.189	0.213	0.181	0.226	0.171	0.136
F874_172.0793_9.3_P	0.088	0.019	0.040	0.018	0.033	0.114	0.039	0.031	0.029	0.067	0.051	0.403	0.033	0.041	0.050	0.163	0.156	0.050	0.106	0.050	0.019	0.051	0.101	0.018	0.033	0.022	0.054	0.042	0.117	0.050	0.050	0.027	0.025	0.044	0.071	0.042	0.063	0.066	0.194	0.170	0.148	0.074	0.089	0.081	0.323	0.190	0.062	0.045	0.066	0.076	0.103	0.051	0.059
F875_516.3085_3.9_P	0.297	0.367	0.410	0.355	0.017	0.259	0.073	0.273	0.199	0.248	0.149	0.277	0.242	0.096	0.258	0.060	0.237	0.264	0.205	0.295	0.200	0.225	0.229	0.222	0.247	0.300	0.147	0.203	0.225	0.166	0.095	0.134	0.093	0.161	0.142	0.128	0.130	0.115	0.331	0.214	0.261	0.385	0.337	0.204	0.310	0.258	0.147	0.211	0.195	0.122	0.049	0.142	0.017
F876_251.2018_3.4_N	0.114	0.118	0.189	0.139	0.267	0.239	0.082	0.197	0.194	0.115	0.201	0.171	0.209	0.048	0.210	0.165	0.137	0.101	0.066	0.146	0.154	0.192	0.208	0.230	0.188	0.150	0.146	0.129	0.164	0.147	0.127	0.107	0.121	0.127	0.073	0.127	0.083	0.119	0.118	0.108	0.081	0.060	0.133	0.120	0.113	0.129	0.106	0.109	0.088	0.135	0.097	0.117	0.065
F877_188.9053_12.8_N	0.137	0.189	0.188	0.171	0.155	0.228	0.004	0.160	0.171	0.135	0.114	0.126	0.165	0.091	0.162	0.162	0.176	0.192	0.108	0.165	0.127	0.147	0.179	0.121	0.137	0.206	0.125	0.165	0.194	0.144	0.142	0.191	0.130	0.174	0.161	0.158	0.200	0.161	0.114	0.149	0.118	0.128	0.125	0.109	0.122	0.100	0.108	0.121	0.102	0.157	0.118	0.090	0.116
F878_169.0579_15.2_P	0.060	0.541	0.276	0.201	0.007	0.038	0.010	0.162	0.399	0.233	0.285	0.315	0.252	0.333	0.049	0.146	0.120	0.317	0.007	0.368	0.134	0.190	0.240	0.127	0.289	0.006	0.024	0.278	0.067	0.100	0.073	0.041	0.152	0.066	0.394	0.142	0.035	0.305	0.108	0.081	0.011	0.401	0.058	0.061	0.142	0.084	0.146	0.073	0.026	0.105	0.067	0.100	0.007
F879_207.1126_4.3_P	0.126	0.027	0.131	0.150	0.062	0.075	0.108	0.115	0.379	0.119	0.138	0.225	0.153	0.051	0.153	0.074	0.130	0.192	0.155	0.226	0.123	0.085	0.112	0.079	0.132	0.113	0.055	0.083	0.075	0.075	0.110	0.115	0.132	0.127	0.224	0.137	0.190	0.121	0.054	0.028	0.067	0.136	0.056	0.063	0.086	0.030	0.173	0.097	0.172	0.102	0.050	0.031	0.120
F880_175.1075_13.5_P	0.040	0.009	0.033	0.020	0.032	0.071	0.024	0.037	0.023	0.017	0.028	0.019	0.016	0.123	0.026	0.030	0.034	0.029	0.061	0.021	0.022	0.065	0.059	0.038	0.032	0.036	0.062	0.029	0.169	0.063	0.051	0.045	0.041	0.128	0.069	0.150	0.043	0.023	0.157	0.213	0.284	0.124	0.217	0.174	0.205	0.242	0.098	0.035	0.030	0.122	0.275	0.028	0.016
F881_247.1106_8.5_P	0.064	0.037	0.092	0.033	0.042	0.045	0.032	0.032	0.064	0.030	0.077	0.117	0.065	0.035	0.067	0.061	0.057	0.046	0.120	0.119	0.055	0.080	0.096	0.025	0.042	0.053	0.105	0.071	0.096	0.036	0.103	0.114	0.169	0.047	0.061	0.092	0.060	0.016	0.127	0.132	0.089	0.096	0.111	0.216	0.075	0.170	0.028	0.008	0.030	0.040	0.060	0.013	0.006
F882_857.5176_3.1_N	0.137	0.199	0.141	0.234	0.097	0.172	0.145	0.212	0.220	0.212	0.157	0.164	0.117	0.238	0.154	0.144	0.313	0.204	0.151	0.186	0.200	0.099	0.165	0.245	0.237	0.148	0.147	0.177	0.151	0.231	0.284	0.212	0.179	0.191	0.180	0.194	0.163	0.210	0.232	0.199	0.131	0.144	0.093	0.088	0.174	0.119	0.195	0.142	0.180	0.166	0.118	0.077	0.177
F883_393.1679_13.4_P	0.171	0.123	0.105	0.149	0.100	0.119	0.068	0.088	0.119	0.150	0.165	0.106	0.129	0.060	0.156	0.133	0.148	0.142	0.103	0.154	0.108	0.105	0.092	0.111	0.111	0.108	0.088	0.107	0.135	0.175	0.131	0.165	0.126	0.144	0.188	0.113	0.161	0.137	0.150	0.123	0.215	0.287	0.267	0.090	0.206	0.162	0.170	0.121	0.184	0.174	0.201	0.168	0.149
F884_164.0915_13.5_P	0.070	0.057	0.072	0.064	0.070	0.124	0.031	0.150	0.049	0.061	0.053	0.102	0.133	0.050	0.088	0.083	0.096	0.089	0.059	0.044	0.042	0.078	0.070	0.088	0.129	0.067	0.113	0.053	0.270	0.120	0.116	0.132	0.080	0.167	0.156	0.163	0.127	0.128	0.092	0.125	0.146	0.085	0.111	0.129	0.120	0.136	0.089	0.048	0.073	0.158	0.266	0.081	0.039

F885_157.0969_7.4_P	0.104	0.004	0.148	0.180	0.210	0.062	0.080	0.044	0.136	0.069	0.102	0.143	0.182	0.117	0.158	0.169	0.159	0.152	0.107	0.124	0.104	0.133	0.130	0.194	0.115	0.104	0.152	0.093	0.261	0.148	0.060	0.202	0.096	0.230	0.102	0.198	0.202	0.125	0.167	0.123	0.157	0.110	0.038	0.145	0.102	0.117	0.103	0.110	0.095	0.098	0.155	0.101	0.069
F886_568.2677_3.6_N	0.018	0.021	0.013	0.017	0.020	0.013	0.029	0.018	0.013	0.015	0.012	0.010	0.014	0.053	0.012	0.019	0.015	0.028	0.016	0.013	0.010	0.011	0.009	0.029	0.012	0.027	0.020	0.029	0.019	0.028	0.013	0.019	0.012	0.016	0.014	0.020	0.018	0.013	0.119	0.253	0.119	0.143	0.188	0.089	0.129	0.081	0.090	0.179	0.232	0.151	0.092	0.100	0.149
F887_143.0813_12.7_P	0.082	0.068	0.098	0.082	0.071	0.169	0.102	0.109	0.074	0.054	0.055	0.115	0.132	0.036	0.089	0.094	0.111	0.119	0.130	0.097	0.027	0.047	0.080	0.121	0.060	0.069	0.133	0.084	0.147	0.132	0.060	0.136	0.034	0.068	0.053	0.147	0.149	0.104	0.252	0.177	0.260	0.260	0.232	0.157	0.270	0.199	0.089	0.087	0.068	0.125	0.106	0.063	0.041
F888_358.1635_15.4_P	0.512	0.301	0.320	0.487	0.175	0.215	0.243	0.206	0.356	0.445	0.252	0.247	0.273	0.325	0.255	0.185	0.260	0.496	0.525	0.325	0.256	0.207	0.260	0.192	0.291	0.228	0.182	0.326	0.153	0.288	0.312	0.298	0.296	0.353	0.341	0.291	0.269	0.391	0.183	0.134	0.231	0.307	0.142	0.129	0.210	0.133	0.225	0.173	0.274	0.162	0.184	0.150	0.274
F889_498.287_4.2_P	0.176	0.017	0.019	0.015	0.010	0.009	1.109	0.010	0.012	0.011	0.027	0.008	0.011	0.128	0.011	0.363	0.010	0.013	0.029	0.024	0.010	0.010	0.015	0.020	0.009	0.010	0.120	0.252	0.009	0.025	0.110	0.010	0.149	0.010	0.010	0.009	0.011	0.012	0.010	0.112	0.047	0.013	0.010	0.219	0.019	0.019	0.008	0.008	0.010	0.007	0.324	0.008	0.017
F890_381.1796_13.1_P	0.073	0.028	0.111	0.140	0.085	0.066	0.029	0.041	0.037	0.293	0.129	0.039	0.028	0.052	0.066	0.162	0.098	0.058	0.085	0.038	0.008	0.166	0.053	0.195	0.042	0.249	0.037	0.075	0.142	0.040	0.032	0.100	0.033	0.163	0.104	0.116	0.071	0.028	0.033	0.043	0.115	0.047	0.041	0.037	0.067	0.072	0.178	0.282	0.043	0.099	0.216	0.094	0.088
F891_115.0764_4.5_N	0.083	0.011	0.143	0.085	0.136	0.123	0.002	0.169	0.179	0.104	0.181	0.186	0.176	0.841	0.131	0.142	0.205	0.146	0.121	0.159	0.132	0.087	0.134	0.150	0.168	0.180	0.114	0.099	0.106	0.131	0.170	0.139	0.089	0.214	0.126	0.049	0.156	0.141	0.059	0.071	0.082	0.056	0.102	0.075	0.069	0.078	0.137	0.120	0.161	0.085	0.080	0.101	0.009
F892_303.1547_12.4_P	0.195	0.142	0.221	0.200	0.115	0.144	0.202	0.212	0.211	0.150	0.194	0.179	0.162	0.052	0.227	0.269	0.317	0.199	0.194	0.246	0.231	0.242	0.300	0.175	0.188	0.153	0.126	0.215	0.159	0.305	0.350	0.191	0.210	0.211	0.227	0.260	0.244	0.391	0.073	0.087	0.137	0.187	0.170	0.109	0.186	0.167	0.232	0.179	0.207	0.221	0.179	0.234	0.141
F893_176.0913_9.4_P	0.045	0.021	0.045	0.024	0.042	0.067	0.029	0.067	0.031	0.034	0.031	0.041	0.036	0.054	0.028	0.041	0.034	0.041	0.026	0.028	0.030	0.072	0.041	0.049	0.044	0.040	0.072	0.027	0.090	0.057	0.051	0.072	0.055	0.079	0.085	0.134	0.104	0.018	0.142	0.188	0.195	0.094	0.193	0.228	0.106	0.136	0.039	0.026	0.034	0.080	0.128	0.020	0.019
F894_195.0319_15.1_P	0.092	0.038	0.155	0.121	0.130	0.284	0.018	0.226	0.181	0.130	0.112	0.157	0.290	0.042	0.249	0.117	0.305	0.098	0.109	0.124	0.294	0.200	0.143	0.114	0.136	0.155	0.216	0.124	0.160	0.356	0.186	0.233	0.114	0.238	0.179	0.153	0.283	0.509	0.248	0.206	0.189	0.121	0.162	0.114	0.232	0.157	0.268	0.220	0.265	0.236	0.153	0.204	0.061
F895_822.5985_3.3_P	0.489	0.715	0.367	0.590	0.128	0.187	0.763	0.340	0.206	0.351	0.227	0.213	0.275	1.435	0.287	0.319	0.243	0.614	0.362	0.188	0.289	0.256	0.106	0.155	0.194	0.216	0.238	0.338	0.234	0.251	0.336	0.228	0.240	0.453	0.349	0.174	0.184	0.473	0.119	0.129	0.128	0.358	0.176	0.122	0.278	0.168	0.343	0.184	0.289	0.147	0.215	0.163	0.608
F896_301.1424_15.7_P	0.362	0.232	0.225	0.260	0.153	0.146	0.215	0.105	0.178	0.363	0.258	0.143	0.177	0.280	0.148	0.185	0.222	0.206	0.499	0.208	0.143	0.138	0.176	0.195	0.140	0.249	0.116	0.136	0.173	0.194	0.191	0.154	0.199	0.277	0.337	0.208	0.162	0.286	0.103	0.066	0.177	0.322	0.156	0.044	0.168	0.090	0.348	0.235	0.331	0.239	0.208	0.268	0.431
F897_246.1079_13.6_P	0.121	0.101	0.150	0.134	0.191	0.253	0.072	0.225	0.144	0.095	0.121	0.183	0.228	0.040	0.215	0.214	0.195	0.166	0.081	0.115	0.118	0.147	0.155	0.188	0.138	0.100	0.183	0.117	0.215	0.159	0.149	0.267	0.111	0.168	0.146	0.241	0.261	0.196	0.198	0.141	0.139	0.114	0.160	0.137	0.143	0.107	0.168	0.150	0.197	0.240	0.194	0.200	0.105
F898_146.0813_14.5_P	0.092	0.141	0.076	0.071	0.126	0.089	0.052	0.135	0.106	0.069	0.126	0.088	0.085	0.462	0.111	0.099	0.108	0.113	0.081	0.077	0.072	0.065	0.151	0.094	0.106	0.090	0.165	0.088	0.207	0.089	0.237	0.130	0.065	0.182	0.099	0.160	0.143	0.091	0.136	0.173	0.147	0.114	0.129	0.112	0.106	0.154	0.171	0.091	0.097	0.166	0.149	0.101	0.065
F899_359.2034_25.3_P	0.273	0.106	0.252	0.246	0.246	0.263	0.150	0.221	0.327	0.277	0.371	0.283	0.384	0.038	0.315	0.322	0.292	0.357	0.324	0.269	0.431	0.403	0.340	0.307	0.379	0.304	0.334	0.359	0.344	0.273	0.530	0.446	0.567	0.425	0.412	0.379	0.341	0.218	0.146	0.169	0.202	0.113	0.186	0.143	0.187	0.179	0.288	0.260	0.377	0.344	0.282	0.328	0.153
F900_531.2991_3.7_N	0.108	0.006	0.020	0.005	0.009	0.005	0.186	0.006	0.007	0.002	0.015	0.005	0.004	0.025	0.007	0.208	0.004	0.004	0.016	0.014	0.004	0.005	0.021	0.029	0.004	0.011	0.102	0.149	0.011	0.028	0.066	0.006	0.117	0.003	0.003	0.007	0.008	0.002	0.017	0.085	0.037	0.020	0.013	0.177	0.025	0.029	0.006	0.006	0.006	0.004	0.140	0.005	0.004
F901_418.2038_15.7_P	0.006	0.020	0.010	0.007	0.016	0.010	0.008	0.025	0.005	0.023	0.004	0.004	0.019	0.026	0.029	0.006	0.021	0.032	0.004	0.003	0.002	0.015	0.007	0.080	0.004	0.037	0.014	0.021	0.013	0.028	0.013	0.015	0.003	0.016	0.007	0.023	0.017	0.008	0.034	0.031	0.075	0.039	0.053	0.020	0.085	0.025	0.079	0.249	0.046	0.037	0.018	0.071	0.030
F902_225.186_3.5_N	0.136	0.132	0.216	0.154	0.303	0.234	0.088	0.275	0.256	0.159	0.217	0.243	0.271	0.103	0.265	0.236	0.216	0.133	0.093	0.193	0.230	0.213	0.188	0.298	0.252	0.230	0.259	0.192	0.234	0.246	0.201	0.184	0.190	0.153	0.125	0.222	0.178	0.199	0.206	0.183	0.159	0.119	0.271	0.248	0.170	0.245	0.223	0.223	0.246	0.210	0.237	0.327	0.119
F903_469.3112_4.2_P	0.375	0.238	0.264	0.277	0.200	0.165	0.242	0.210	0.288	0.192	0.196	0.203	0.184	0.093	0.213	0.182	0.232	0.237	0.228	0.317	0.214	0.201	0.250	0.215	0.198	0.235	0.178	0.237	0.206	0.162	0.190	0.150	0.186	0.158	0.189	0.162	0.119	0.155	0.218	0.154	0.177	0.288	0.218	0.127	0.182	0.139	0.225	0.203	0.227	0.172	0.165	0.186	0.151
F904_154.0529_10.4_P	0.074	0.069	0.131	0.095	0.133	0.186	0.061	0.163	0.104	0.068	0.097	0.150	0.151	0.052	0.147	0.146	0.149	0.102	0.062	0.104	0.095	0.096	0.116	0.161	0.114	0.137	0.143	0.100	0.145	0.131	0.106	0.162	0.076	0.104	0.079	0.155	0.203	0.113	0.154	0.122	0.153	0.128	0.191	0.141	0.160	0.132	0.088	0.092	0.080	0.113	0.101	0.065	0.053
F905_370.2943_4.2_P	0.070	0.055	0.068	0.041	0.078	0.074	0.034	0.053	0.109	0.096	0.090	0.120	0.060	0.081	0.158	0.083	0.080	0.108	0.098	0.087	0.077	0.079	0.067	0.077	0.105	0.069	0.095	0.077	0.086	0.235	0.107	0.091	0.072	0.061	0.097	0.088	0.070	0.098	0.022	0.016	0.028	0.050	0.053	0.067	0.022	0.046	0.085	0.043	0.118	0.048	0.048	0.238	0.038
F906_480.2777_4.2_P	0.113	0.014	0.013	0.012	0.008	0.008	0.704	0.009	0.013	0.009	0.009	0.007	0.008	0.046	0.008	0.205	0.008	0.010	0.018	0.015	0.008	0.008	0.007	0.014	0.007	0.008	0.063	0.119	0.007	0.010	0.052	0.008	0.051	0.008	0.008	0.007	0.009	0.010	0.007	0.038	0.026	0.009	0.008	0.136	0.008	0.019	0.006	0.007	0.008	0.006	0.181	0.007	0.014
F907_175.0013_10.4_P	0.054	0.044	0.075	0.059	0.066	0.071	0.052	0.062	0.059	0.068	0.046	0.059	0.032	0.028	0.040	0.059	0.047	0.045	0.057	0.054	0.042	0.030	0.036	0.044	0.051	0.048	0.049	0.073	0.046	0.057	0.052	0.050	0.047	0.057	0.078	0.081	0.068	0.080	0.119	0.105	0.084	0.171	0.119	0.085	0.135	0.114	0.125	0.096	0.201	0.160	0.120	0.088	0.194
F908_617.2728_3.7_N	0.017	0.030	0.027	0.014	0.042	0.021	0.039	0.019	0.013	0.009	0.004	0.020	0.015	0.025	0.033	0.017	0.015	0.017	0.008	0.011	0.011	0.009	0.015	0.021	0.006	0.035	0.029	0.015	0.012	0.010	0.010	0.010	0.006	0.005	0.007	0.018	0.018	0.006	0.023	0.064	0.022	0.020	0.018	0.108	0.017	0.029	0.002	0.003	0.006	0.009	0.004	0.005	0.009
F909_387.2097_27.1_P	0.183	0.113	0.178	0.194	0.155	0.164	0.038	0.159	0.200	0.195	0.134	0.143	0.186	0.082	0.170	0.109	0.166	0.240	0.168	0.192	0.194	0.172	0.157	0.133	0.181	0.167	0.156	0.121	0.187	0.184	0.241	0.233	0.304	0.280	0.254	0.199	0.185	0.186	0.109	0.088	0.149	0.123	0.146	0.080	0.121	0.106	0.172	0.180	0.187	0.136	0.174	0.172	0.178
F910_398.3259_4_P	0.096	0.071	0.086	0.056	0.081	0.047	0.059	0.067	0.123	0.044	0.088	0.126	0.101	0.034	0.106	0.089	0.073	0.133	0.062	0.092	0.089	0.043	0.055	0.066	0.091	0.073	0.076	0.087	0.066	0.139	0.105	0.088	0.093	0.076	0.093	0.082	0.055	0.103	0.013	0.006	0.009	0.041	0.033	0.036	0.017	0.028	0.053	0.019	0.079	0.072	0.040	0.176	0.023
F912_318.9315_12.8_N	0.073	0.033	0.229	0.103	0.099	0.223	0.011	0.143	0.171	0.063	0.122	0.149	0.124	0.033	0.105	0.239	0.237	0.145	0.062	0.211	0.199	0.123	0.202	0.087	0.099	0.175	0.103	0.119	0.090	0.136	0.088	0.215	0.123	0.142	0.144	0.128	0.213	0.234	0.131	0.139	0.112	0.101	0.200	0.114	0.129	0.121	0.133	0.068	0.189	0.186	0.166	0.133	0.033
F913_480.3106_3.8_N	0.122	0.178	0.043	0.196	0.030	0.128	0.152	0.133	0.071	0.070	0.075	0.108	0.055	0.046	0.115	0.049	0.054	0.115	0.100	0.130	0.081	0.097	0.126	0.013	0.126	0.126	0.094	0.126	0.084	0.069	0.089	0.079	0.128	0.061	0.065	0.084	0.053	0.051	0.155	0.148	0.095	0.087	0.087	0.113	0.074	0.138	0.102	0.087	0.108	0.102	0.086	0.076	0.080
F914_450.2622_4_N	0.129	0.238	0.234	0.197	0.252	0.170	0.158	0.155	0.245	0.122	0.065	0.199	0.156	0.072	0.147	0.057	0.102	0.114	0.116	0.120	0.091	0.125	0.115	0.133	0.133	0.202	0.109	0.135	0.068	0.049	0.061	0.061	0.051	0.055	0.058	0.088	0.073	0.059	0.112	0.141	0.072	0.092	0.076	0.179	0.069	0.069	0.055	0.064	0.047	0.058	0.022	0.049	0.051
F915_171.0298_13.7_N	0.107	0.229	0.132	0.166	0.148	0.116	0.208	0.165	0.126	0.119	0.145	0.131	0.165	0.257	0.160	0.121	0.143	0.139	0.145	0.113	0.168	0.106	0.121	0.144	0.134	0.147	0.160	0.142	0.092	0.114	0.121	0.141	0.142	0.117	0.112	0.121	0.152	0.183	0.121	0.085	0.071	0.098	0.110	0.105	0.082	0.074	0.111	0.097	0.125	0.096	0.084	0.109	0.218
F916_190.1181_15.3_P	0.005	0.008	0.006	0.005	0.004	0.012	0.007	0.006	0.008	0.005	0.004	0.007	0.004	0.030	0.004	0.005	0.006	0.006	0.006	0.004	0.004	0.009	0.007	0.005	0.004	0.007	0.008	0.004	0.021	0.007	0.008	0.004	0.005	0.006	0.005	0.016	0.004	0.005	0.097	0.109	0.144	0.081	0.122	0.131	0.116	0.130	0.014	0.004	0.004	0.019	0.046	0.003	0.007
F917_377.1404_13.6_P	0.004	0.008	0.010	0.005	0.012	0.014	0.008	0.013	0.009	0.004	0.009	0.009	0.012	0.041	0.012	0.010	0.010	0.008	0.006	0.010	0.011	0.012	0.010	0.014	0.012	0.016	0.019	0.009	0.019	0.016	0.012	0.013	0.013	0.012	0.012	0.015	0.018	0.006	0.108	0.135	0.163	0.081	0.126	0.135	0.133	0.147	0.006	0.011	0.006	0.008	0.011	0.007	0.007
F918_277.0889_13.9_P	0.077	0.022	0.074	0.062	0.082	0.113	0.017	0.106	0.078	0.037	0.066	0.116	0.119	0.121	0.110	0.150	0.091	0.091	0.047	0.062	0.059	0.112	0.123	0.098	0.068	0.074	0.126	0.109	0.097	0.119	0.071	0.112	0.081	0.068	0.097	0.103	0.157	0.072	0.076	0.098	0.088	0.055	0.109	0.122	0.043	0.064	0.054	0.063	0.049	0.105	0.076	0.059	0.042
F919_774.5075_3.4_N	0.177	0.257	0.106	0.143	0.112	0.102	0.175	0.158	0.121	0.149	0.110	0.152	0.126	0.539	0.143	0.077	0.167	0.185	0.264	0.192	0.115	0.154	0.136	0.148	0.160	0.177	0.201	0.165	0.072	0.125	0.121	0.155	0.113	0.122	0.089	0.143	0.182	0.132	0.137	0.138	0.091	0.199	0.126	0.089	0.094	0.094	0.076	0.085	0.072	0.079	0.042	0.082	0.194
F920_288.0582_10.2_P	0.008	0.007	0.014	0.006	0.021	0.022	0.007	0.025	0.015	0.024	0.024	0.022	0.022	0.033	0.011	0.029	0.019	0.011	0.006	0.024	0.023	0.035	0.027	0.032	0.023	0.022	0.052	0.055	0.055	0.054	0.037	0.023	0.025	0.021	0.023	0.041	0.026	0.035	0.041	0.037	0.066	0.048	0.064	0.064	0.079	0.072	0.167	0.174	0.111	0.135	0.135	0.125	0.062
F921_716.5583_3.5_P	0.346	0.801	0.188	0.179	0.130	0.170	0.706	0.183	0.383	0.292	0.173	0.163	0.156	0.418	0.135	0.101	0.208	0.577	0.311	0.240	0.245	0.110	0.131	0.213	0.220	0.193	0.175	0.261	0.100	0.246	0.087	0.252	0.163	0.070	0.166	0.171	0.233	0.287	0.098	0.110	0.158	0.291	0.133	0.111	0.114	0.069	0.154	0.222	0.275	0.233	0.149	0.132	0.426
F922_134.0692_14.8_P	0.136	0.097	0.107	0.118	0.105	0.152	0.064	0.193	0.169	0.119	0.143	0.081	0.184	0.076	0.146	0.124	0.185	0.180	0.149	0.212	0.194	0.139	0.142	0.111	0.195	0.071	0.144	0.130	0.161	0.186	0.197	0.176	0.262	0.158	0.162	0.194	0.184	0.287	0.092	0.070	0.081	0.094	0.120	0.076	0.079	0.090	0.146	0.111	0.168	0.158	0.141	0.134	0.129
F923_556.3385_3.9_P	0.107	0.072	0.116	0.111	0.075	0.065	0.155	0.091	0.081	0.074	0.050	0.099	0.068	0.035	0.081	0.060	0.055	0.067	0.058	0.067	0.072	0.112	0.068	0.047	0.057	0.089	0.060	0.079	0.051	0.045	0.045	0.041	0.054	0.050	0.064	0.066	0.035	0.048	0.092	0.090	0.063	0.104	0.080	0.094	0.085	0.088	0.072	0.038	0.079	0.082	0.055	0.045	0.044
F925_147.0761_11.4_P	0.013	0.089	0.072	0.040	0.034	0.039	0.090	0.036	0.073	0.077	0.098	0.116	0.065	0.418	0.076	0.012	0.148	0.093	0.086	0.090	0.084	0.057	0.117	0.014	0.095	0.042	0.046	0.014	0.130	0.066	0.128	0.045	0.074	0.148	0.146	0.117	0.110	0.102	0.023	0.006	0.012	0.047	0.030	0.013	0.049	0.007	0.067	0.026	0.153	0.127	0.016	0.089	0.100
F927_148.0424_15_P	0.012	0.009	0.013	0.016	0.007	0.022	0.008	0.016	0.018	0.009	0.012	0.008	0.012	0.025	0.011	0.012	0.006	0.009	0.007	0.013	0.017	0.007	0.006	0.007	0.008	0.008	0.012	0.008	0.011	0.012	0.012	0.008	0.008	0.010	0.009	0.021	0.013	0.011	0.034	0.045	0.049	0.034	0.050	0.141	0.049	0.102	0.004	0.008	0.011	0.014	0.013	0.017	0.009
F928_248.1488_11_P	0.038	0.021	0.041	0.034	0.049	0.044	0.026	0.044	0.042	0.043	0.055	0.061	0.050	0.041	0.069	0.076	0.059	0.077	0.041	0.050	0.044	0.058	0.070	0.050	0.051	0.042	0.064	0.051	0.065	0.077	0.074	0.046	0.068	0.055	0.058	0.116	0.131	0.069	0.056	0.050	0.075	0.072	0.080	0.094	0.081	0.079	0.121	0.099	0.144	0.131	0.156	0.185	0.063
F929_244.1109_10.2_P	0.020	0.007	0.020	0.014	0.023	0.030	0.007	0.028	0.017	0.026	0.027	0.025	0.024	0.031	0.013	0.031	0.025	0.017	0.011	0.030	0.024	0.036	0.033	0.031	0.028	0.024	0.056	0.058	0.058	0.059	0.034	0.027	0.029	0.028	0.024	0.043	0.021	0.039	0.040	0.043	0.080	0.053	0.064	0.053	0.080	0.068	0.175	0.139	0.111	0.143	0.134	0.125	0.057
F930_138.1024_15.3_P	0.038	0.011	0.022	0.023	0.015	0.052	0.010	0.012	0.007	0.006	0.005	0.050	0.009	0.039	0.011	0.024	0.063	0.024	0.019	0.016	0.010	0.025	0.035	0.032	0.011	0.028	0.019	0.012	0.064	0.036	0.012	0.013	0.005	0.011	0.008	0.029	0.011	0.007	0.160	0.119	0.168	0.123	0.156	0.113	0.171	0.167	0.021	0.039	0.005	0.062	0.055	0.015	0.008
F931_239.2018_3.5_N	0.071	0.085	0.140	0.091	0.154	0.147	0.046	0.139	0.128	0.086	0.123	0.111	0.157	0.056	0.135	0.099	0.093	0.069	0.053	0.095	0.121	0.111	0.108	0.169	0.139	0.112	0.119	0.096	0.110	0.096	0.094	0.076	0.087	0.076	0.045	0.097	0.065	0.078	0.075	0.074	0.061	0.041	0.093	0.092	0.061	0.079	0.077	0.079	0.067	0.080	0.069	0.100	0.038
F934_509.3301_3.1_N	0.041	0.022	0.093	0.148	0.036	0.057	0.021	0.064	0.131	0.114	0.158	0.041	0.114	0.009	0.057	0.134	0.100	0.064	0.073	0.094	0.154	0.047	0.142	0.200	0.174	0.075	0.065	0.144	0.050	0.091	0.085	0.082	0.140	0.140	0.138	0.077	0.168	0.186	0.043	0.038	0.134	0.098	0.048	0.049	0.054	0.069	0.171	0.078	0.150	0.071	0.054	0.065	0.054
F935_148.1157_25.4_P	0.245	0.119	0.285	0.227	0.403	0.334	0.143	0.311	0.332	0.247	0.478	0.283	0.441	0.079	0.405	0.469	0.325	0.334	0.285	0.265	0.629	0.562	0.472	0.409	0.448	0.403	0.475	0.432	0.530	0.339	0.774	0.575	0.781	0.499	0.468	0.484	0.406	0.255	0.203	0.219	0.235	0.112	0.223	0.183	0.217	0.255	0.314	0.327	0.478	0.465	0.490	0.464	0.143
F936_606.3413_3.9_N	0.064	0.115	0.137	0.084	0.115	0.086	0.116	0.060	0.110	0.070	0.044	0.120	0.116	0.021	0.086	0.063	0.063	0.071	0.045	0.054	0.060	0.045	0.051	0.089	0.064	0.115	0.085	0.128	0.055	0.045	0.082	0.058	0.083	0.036	0.034	0.066	0.054	0.040	0.105	0.099	0.069	0.059	0.085	0.123	0.058	0.060	0.061	0.088	0.042	0.063	0.041	0.052	0.057
F937_202.1083_4.5_N	0.000	0.001	0.047	0.029	0.060	0.054	0.002	0.046	0.054	0.031	0.063	0.072	0.052	0.005	0.057	0.087	0.062	0.055	0.001	0.073	0.054	0.059	0.061	0.055	0.110	0.096	0.109	0.100	0.098	0.086	0.155	0.143	0.109	0.164	0.165	0.098	0.185	0.082	0.150	0.141	0.101	0.000	0.081	0.157	0.107	0.141	0.106	0.081	0.137	0.153	0.160	0.096	0.001
F938_554.3098_4_N	0.213	0.208	0.248	0.224	0.249	0.163	0.181	0.203	0.252	0.146	0.180	0.193	0.169	0.089	0.183	0.137	0.199	0.168	0.183	0.228	0.209	0.187	0.187	0.205	0.180	0.220	0.186	0.212	0.185	0.147	0.165	0.119	0.164	0.122	0.131	0.137	0.113	0.121	0.202	0.163	0.161	0.204	0.189	0.157	0.157	0.141	0.150	0.185	0.185	0.150	0.133	0.148	0.121
F939_101.0596_15.6_P	0.069	0.050	0.058	0.045	0.082	0.089	0.047	0.076	0.052	0.066	0.099	0.075	0.062	0.034	0.063	0.076	0.051	0.045	0.052	0.053	0.047	0.077	0.052	0.073	0.077	0.074	0.121	0.118	0.114	0.107	0.049	0.057	0.075	0.057	0.061	0.115	0.064	0.063	0.111	0.155	0.144	0.153	0.129	0.150	0.128	0.139	0.151	0.153	0.134	0.151	0.149	0.122	0.127

F940_148.0731_15.4_P	0.285	0.147	0.249	0.254	0.213	0.222	0.151	0.267	0.350	0.246	0.271	0.220	0.320	0.170	0.287	0.251	0.267	0.313	0.271	0.294	0.326	0.294	0.328	0.229	0.310	0.235	0.250	0.292	0.262	0.261	0.331	0.333	0.354	0.334	0.335	0.277	0.298	0.318	0.187	0.187	0.189	0.180	0.191	0.134	0.188	0.189	0.186	0.160	0.241	0.189	0.245	0.204	0.152
F941_578.3093_4_N	0.115	0.120	0.129	0.119	0.132	0.091	0.113	0.122	0.133	0.083	0.074	0.105	0.091	0.043	0.063	0.053	0.089	0.092	0.093	0.119	0.089	0.116	0.097	0.112	0.097	0.121	0.105	0.113	0.095	0.073	0.071	0.061	0.070	0.059	0.068	0.073	0.056	0.049	0.116	0.130	0.111	0.137	0.127	0.108	0.105	0.113	0.062	0.087	0.081	0.067	0.058	0.061	0.045
F942_157.0365_15_N	0.084	0.007	0.017	0.016	0.015	0.024	0.026	0.016	0.012	0.005	0.007	0.016	0.016	0.015	0.015	0.211	0.017	0.015	0.020	0.017	0.009	0.009	0.017	0.039	0.010	0.012	0.034	0.087	0.016	0.026	0.029	0.014	0.023	0.006	0.010	0.020	0.024	0.009	0.018	0.035	0.026	0.017	0.020	0.023	0.018	0.023	0.016	0.006	0.011	0.014	0.018	0.008	0.007
F943_852.5737_3.4_N	0.183	0.236	0.149	0.140	0.113	0.140	0.276	0.067	0.166	0.160	0.183	0.080	0.132	0.616	0.106	0.118	0.128	0.191	0.273	0.210	0.177	0.066	0.092	0.213	0.160	0.170	0.120	0.124	0.168	0.125	0.219	0.128	0.147	0.127	0.150	0.123	0.162	0.106	0.065	0.089	0.105	0.083	0.054	0.058	0.102	0.053	0.228	0.159	0.173	0.116	0.126	0.096	0.346
F944_159.0761_13.9_P	0.099	0.061	0.092	0.090	0.108	0.107	0.047	0.131	0.092	0.088	0.112	0.082	0.114	0.065	0.085	0.136	0.122	0.120	0.082	0.101	0.115	0.137	0.171	0.109	0.110	0.111	0.115	0.127	0.172	0.140	0.166	0.132	0.132	0.166	0.146	0.161	0.147	0.139	0.069	0.111	0.119	0.093	0.113	0.088	0.096	0.144	0.104	0.091	0.125	0.145	0.146	0.121	0.080
F945_215.8371_12.8_N	0.113	0.134	0.083	0.121	0.004	0.074	0.037	0.075	0.071	0.015	0.070	0.083	0.078	0.125	0.083	0.067	0.083	0.124	0.109	0.112	0.086	0.101	0.103	0.053	0.068	0.075	0.044	0.101	0.094	0.080	0.073	0.062	0.082	0.085	0.102	0.091	0.094	0.104	0.049	0.056	0.054	0.106	0.067	0.049	0.077	0.054	0.065	0.055	0.085	0.098	0.037	0.042	0.134
F946_483.1091_14.3_P	0.024	0.011	0.027	0.015	0.033	0.034	0.012	0.040	0.030	0.034	0.038	0.034	0.040	0.094	0.035	0.036	0.033	0.019	0.011	0.028	0.031	0.035	0.028	0.039	0.032	0.044	0.048	0.058	0.046	0.057	0.034	0.037	0.041	0.027	0.028	0.049	0.045	0.052	0.039	0.072	0.059	0.044	0.052	0.057	0.055	0.067	0.112	0.212	0.098	0.095	0.114	0.144	0.063
F947_794.5993_3.5_P	0.624	0.631	0.325	0.406	0.243	0.531	0.501	0.415	0.499	0.457	0.220	0.355	0.279	1.567	0.276	0.401	0.442	0.423	0.570	0.368	0.479	0.404	0.576	0.241	0.405	0.278	0.326	0.257	0.471	0.273	0.407	0.542	0.374	0.468	0.441	0.311	0.341	0.594	0.159	0.191	0.291	0.581	0.194	0.198	0.385	0.273	0.464	0.148	0.439	0.537	0.301	0.341	1.534
F948_510.3553_4.1_P	0.268	0.152	0.223	0.184	0.160	0.128	0.167	0.150	0.142	0.107	0.108	0.105	0.078	0.027	0.094	0.093	0.122	0.125	0.113	0.174	0.136	0.087	0.115	0.129	0.103	0.135	0.086	0.141	0.147	0.116	0.109	0.083	0.129	0.078	0.122	0.073	0.078	0.090	0.140	0.094	0.107	0.153	0.132	0.074	0.128	0.071	0.149	0.167	0.107	0.108	0.096	0.111	0.058
F949_196.0283_9.7_N	0.053	0.030	0.067	0.042	0.072	0.097	0.027	0.110	0.060	0.040	0.050	0.082	0.083	0.017	0.084	0.085	0.130	0.069	0.060	0.070	0.092	0.083	0.103	0.079	0.057	0.067	0.083	0.058	0.096	0.093	0.105	0.082	0.085	0.074	0.091	0.108	0.117	0.137	0.122	0.098	0.144	0.083	0.114	0.090	0.158	0.147	0.108	0.078	0.106	0.120	0.130	0.087	0.056
F950_381.1356_9.7_P	0.071	0.023	0.050	0.032	0.059	0.041	0.028	0.056	0.059	0.066	0.092	0.040	0.076	0.030	0.047	0.039	0.041	0.042	0.041	0.081	0.049	0.062	0.047	0.068	0.078	0.060	0.067	0.092	0.046	0.104	0.062	0.063	0.093	0.061	0.074	0.077	0.062	0.076	0.017	0.017	0.012	0.050	0.016	0.018	0.027	0.019	0.161	0.199	0.163	0.141	0.114	0.174	0.113
F951_423.1819_25.2_P	0.345	0.181	0.231	0.311	0.085	0.160	0.010	0.178	0.190	0.338	0.184	0.207	0.186	0.979	0.256	0.158	0.289	0.435	0.457	0.285	0.160	0.148	0.295	0.100	0.150	0.143	0.125	0.179	0.102	0.151	0.162	0.172	0.220	0.247	0.345	0.207	0.358	0.267	0.098	0.081	0.155	0.160	0.087	0.087	0.182	0.089	0.177	0.182	0.266	0.148	0.094	0.131	0.240
F952_318.1904_9.4_P	0.035	0.038	0.069	0.024	0.046	0.112	0.041	0.071	0.059	0.034	0.042	0.116	0.047	0.044	0.073	0.104	0.087	0.035	0.044	0.052	0.063	0.055	0.062	0.074	0.047	0.049	0.089	0.051	0.056	0.104	0.084	0.073	0.045	0.059	0.047	0.104	0.081	0.111	0.102	0.066	0.098	0.053	0.064	0.097	0.147	0.127	0.150	0.042	0.121	0.155	0.172	0.126	0.047
F953_219.1336_14.3_P	0.048	0.024	0.051	0.034	0.080	0.074	0.033	0.053	0.079	0.068	0.122	0.114	0.131	0.071	0.099	0.160	0.089	0.083	0.080	0.080	0.148	0.166	0.141	0.075	0.147	0.124	0.180	0.159	0.164	0.108	0.239	0.145	0.155	0.198	0.134	0.142	0.207	0.056	0.038	0.043	0.030	0.018	0.024	0.032	0.028	0.037	0.081	0.062	0.116	0.134	0.075	0.088	0.046
F954_377.1735_10_P	0.278	0.251	0.231	0.245	0.164	0.155	0.197	0.169	0.255	0.261	0.216	0.234	0.203	0.100	0.254	0.211	0.215	0.298	0.279	0.278	0.220	0.180	0.187	0.133	0.204	0.153	0.129	0.173	0.138	0.233	0.213	0.214	0.241	0.237	0.259	0.193	0.262	0.308	0.189	0.165	0.179	0.315	0.154	0.152	0.234	0.148	0.247	0.149	0.226	0.162	0.145	0.162	0.267
F955_506.3243_3.7_N	0.179	0.269	0.197	0.236	0.105	0.118	0.112	0.192	0.231	0.156	0.180	0.200	0.169	0.118	0.090	0.099	0.115	0.104	0.159	0.234	0.177	0.219	0.115	0.209	0.157	0.137	0.152	0.198	0.077	0.131	0.115	0.125	0.100	0.119	0.128	0.157	0.135	0.127	0.231	0.129	0.107	0.215	0.134	0.134	0.120	0.125	0.100	0.115	0.163	0.094	0.100	0.102	0.096
F956_803.2343_11.1_N	0.037	0.008	0.048	0.037	0.067	0.049	0.005	0.046	0.040	0.024	0.039	0.041	0.039	0.012	0.037	0.035	0.050	0.030	0.025	0.051	0.047	0.030	0.040	0.051	0.043	0.050	0.069	0.046	0.069	0.066	0.051	0.038	0.056	0.050	0.046	0.052	0.059	0.073	0.118	0.110	0.100	0.083	0.152	0.073	0.138	0.145	0.071	0.081	0.090	0.101	0.109	0.094	0.038
F957_480.3442_4.1_P	0.121	0.129	0.096	0.139	0.111	0.083	0.120	0.113	0.119	0.083	0.054	0.081	0.049	0.021	0.080	0.052	0.059	0.093	0.046	0.088	0.077	0.037	0.034	0.137	0.058	0.091	0.070	0.078	0.057	0.061	0.036	0.045	0.048	0.040	0.040	0.063	0.045	0.047	0.048	0.062	0.044	0.074	0.070	0.067	0.033	0.036	0.064	0.141	0.080	0.079	0.031	0.075	0.075
F958_512.2683_4.3_N	0.059	0.002	0.011	0.001	0.001	0.001	0.106	0.001	0.001	0.001	0.015	0.001	0.001	0.013	0.001	0.230	0.001	0.001	0.002	0.002	0.001	0.001	0.008	0.011	0.001	0.002	0.067	0.125	0.003	0.019	0.033	0.001	0.052	0.001	0.001	0.001	0.002	0.001	0.001	0.067	0.004	0.001	0.002	0.084	0.003	0.019	0.001	0.001	0.001	0.000	0.123	0.001	0.002
